# Synthesis of 1,2-Aminoalcohols through Enantioselective Aminoallylation of Ketones by Cu-Catalyzed Reductive Coupling

**DOI:** 10.1021/acs.orglett.1c02258

**Published:** 2021-08-04

**Authors:** Raphael K. Klake, Mytia D. Edwards, Joshua D. Sieber

**Affiliations:** † Department of Chemistry, Virginia Commonwealth University, 1001 West Main Street, Richmond, Virginia 23284-3028, United States; ‡ Medicines for All Institute, VCU, Biotech 8, 737 North Fifth Street, Richmond, Virginia 23219, United States

## Abstract

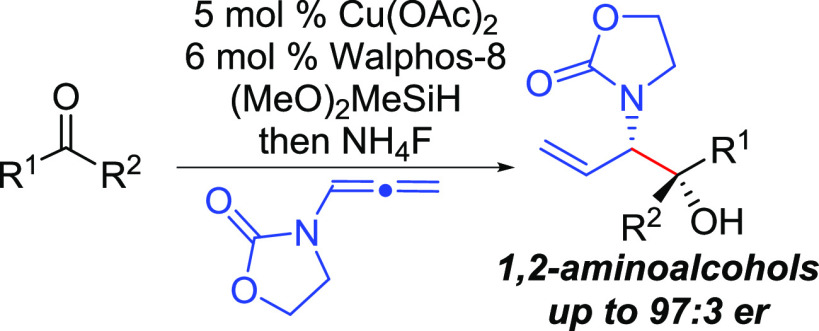

Herein, we report the development of a catalytic enantioselective addition of *N*-substituted allyl equivalents to ketone electrophiles through use of Cu-catalyzed reductive coupling to access important chiral 1,2-aminoalcohol synthons in high levels of regio-, diastereo-, and enantioselectivity. Factors affecting enantioinduction are discussed including the identification of a reversible ketone allylation step that has not been previously reported in Cu-catalyzed reductive coupling.

Chiral vicinal aminoalcohols (**4**, Figure 1) are ubiquitous in nature and represent an important class of biologically active compounds for applications in medicine and the pharmaceutical industry.^1^ A recent survey claims >300,000 compounds, > 2,000 natural products, and >80 Food and Drug Administration approved drugs contain the 1,2-aminoalcohol fragment.^2^ Therefore, asymmetric synthetic methods for the efficient preparation of the 1,2-aminoalcohol motif are needed to provide access to these valuable compounds.^1b−1d,3^ However, asymmetric preparation of **4** through the retrosynthetic disconnection highlighted in Figure 1 is challenging because the electron-withdrawing nature of the amino- and hydroxyl-substituents create a polarity profile where the reacting carbon atoms are both positively charged requiring the need for a formal cross-electrophile coupling.^4^ As a result, common polar processes prevalent in synthetic chemistry defined by the reaction between an electrophilic and nucleophilic species are not immediately amenable to the preparation of **4** since neither reacting carbon atom is nucleophilic.^4^ To circumvent this issue, strategies for reversing the polarity of common functional groups to enable these types of bond disconnection strategies are important methods for synthetic organic chemistry and have been defined as umpolung.^4^ For example, the Henry^5^ reaction between nitroalkanes and carbonyl electrophiles allows for the preparation of 1,2-aminoalcohols through a polar two-electron umpolung process. More recently, nonpolar radical based methods have emerged;^6^ however, enantioselective variants are few.^7^

**Figure 1:**
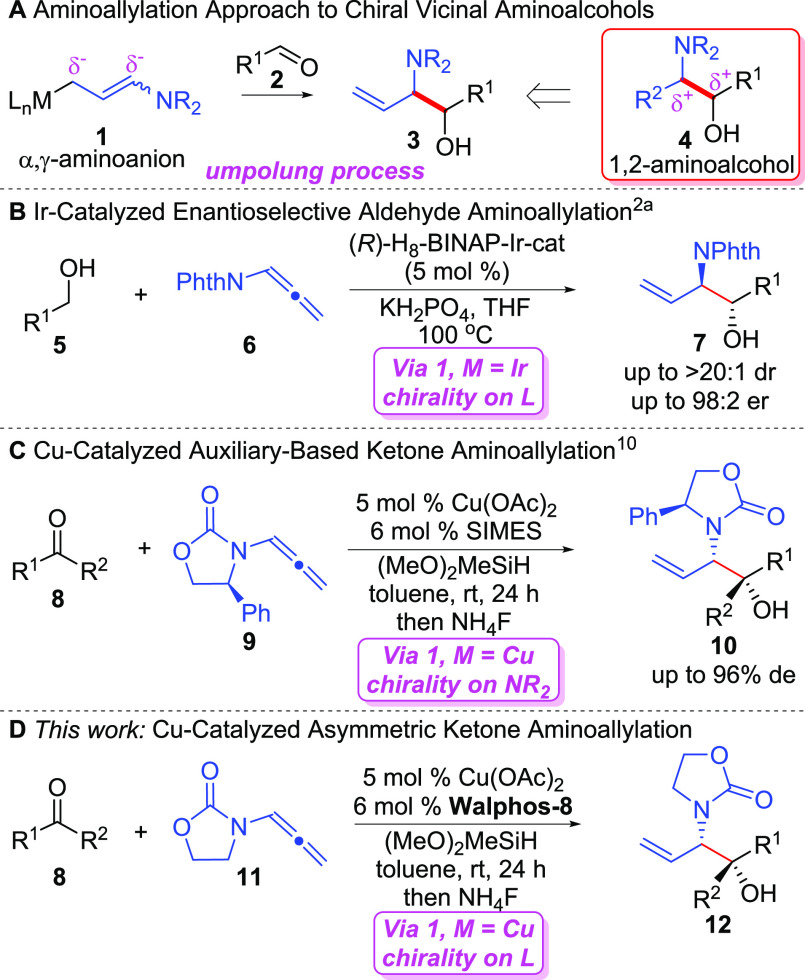
Aminoallylation strategies to 1,2-aminoalcohols.

In the context of an umpolung route toward aminoalcohols **4**, aminoallylation of a carbonyl electrophile **2** by a nucleophilic amino-substituted allymetal reagent **1** represents a powerful technique for the generation of 1,2-aminoalcohol **3** containing alkene functionality that may be utilized in further synthetic manipulations. Indeed, carbonyl allylation chemistries have been extensively developed for the preparation of chiral homoallylic alcohols;^8^ however, the application of amino-substituted organometallic reagent **1** in analogous chemistry has been underdeveloped.^2,9−11^ While Barrett^9^ originally reported an asymmetric process using the stoichiometric preparation of chiral boron reagents of **1** (M = B), only recently have catalytic asymmetric variants emerged to promote the catalytic generation of **1**.^2,10,11^ For example, Krische^2a^ (Figure 1B) recently disclosed an enantioselective Ir-catalyzed aminoallylation of aldehydes through hydrogen transfer from alcohol **5** to allenamide **6** generating the necessary α,γ-aminoanion nucleophile (**1**, M = Ir) and the aldehyde electrophile. Additionally, our group^10a^ recently reported an orthogonal method for the aminoallylation of ketone electrophiles enabled by Cu-catalyzed reductive coupling^12,13^ (Figure 1C). Here, the readily available Evans-auxiliary derived chiral allenamide **9** was employed for stereochemical control affording high diastereoselectivies.^10a,11^ While this method is practical due to the low cost of the Evans auxiliary, we appreciated the fact that absolute stereochemical control by a chiral Cu-catalyst with an achiral allenamide would increase atom efficiency (Figure 1D). Significantly, enantioselective metal catalyzed aminoallylation of ketone electrophiles is unknown and can be more challenging than aldehydes due to the decreased reactivity and steric differentiation of ketones versus aldehydes. Herein we report the development of the first Cu-catalyzed enantioselective aminoallylation of ketone electrophiles.

Initial studies focused on identifying an appropriate chiral ligand scaffold to afford high diastereo- and enantioselectivity in the reductive coupling of ketones and achiral allenamide **11** (Table 1, entries 1–7).^14^ Importantly, in all cases, variable amounts of carbonate migration product **13a** were formed from internal trapping of the Cu-alkoxide^11,14^ intermediate with only single diastereomers of **12a** and **13a** observed. Of the ligands studied, **W8** was identified as the best candidate providing the desired branched reaction product **12a** in good enantioselectivity as a single diastereomer (entry 5). Interestingly, (*S,S*)-Ph-BPE (entry 1) and **J11** (entry 7) were inferior to **W8** despite their widespread use in Cu-catalyzed reductive coupling reactions.^12,13^

**Table 1:**
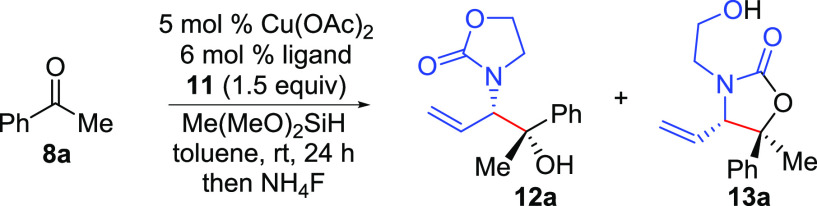
Chiral Ligand Survey^a^

entry	ligand	% **12a**^b^	**12a**:**13a**^b^	er **12a**^c^
1	(*S,S*)-Ph-BPE	64	89:11	20:80
2	(*R*)-BINAP	82	83:17	18:82
3	(*R*)-Segphos	51	86:14	30:70
4	**W3**	64	90:10	57:43
5	**W8**	77	81:19	93:7^d^
6	**J6**	58	91:9	15:85
7	**J11**	60	78:22	28:72
8^e^	**W8**	71	87:13	87:13
9^f^	**W8**	58	>99:1	88:12
10^f^^,^^g^	**W8**	61	90:10	91:9
11^h^	**W8**	45^i^	52:48^j^	85:15^i^^,^^k^
12^f^^,^^h^	**W8**	50^i^	>99:1^j^	97:3
13^f^^,^^g^^,^^h^	**W8**	77^i^	>99:1^j^	96:4

a **1a** (0.25 mmol), **11** (0.375 mmol), and 0.50 mmol Me(MeO)_2_SiH in 0.5 mL of toluene. In all cases, a single diastereomer of **12a** and **13a** was obtained (^1^H NMR spectroscopic analysis). See the Supporting Information for additional details.

b Determined by ^1^H NMR spectroscopy on the unpurified reaction mixture using dimethylfumarate as standard.

c Value determined by chiral HPLC analysis.

d Er of **13a** was 50:50.

e Using 10 equiv of silane.

f 2 equiv of *t*-BuOH added.

g PhCF_3_ used as solvent.

h Propiophenone (**8b**) used in place of **8a**.

i Value for **12b**.

j Ratio of **12b**:**13b**.

k Er fo **13b** was 60:40.
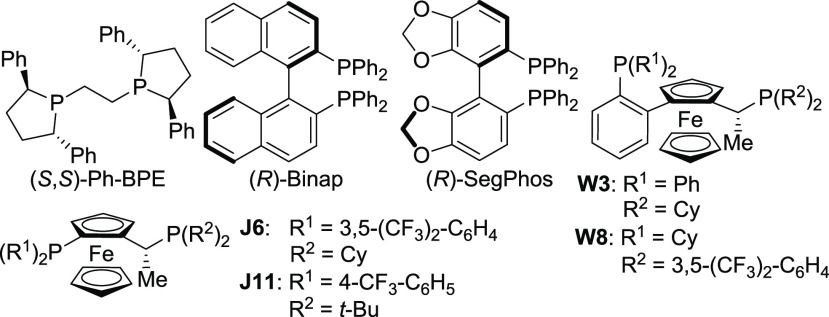

Compounds **12** and **13** were produced in differing enantiopurities (Table 1, entries 5 and 11); one explanation consistent with this outcome is reversibility in the allylcupration.^14^ Reversible allylation^15^ in metal catalyzed reductive coupling reactions has not been identified prior to our work,^11,16^ and this issue would have significant ramifications on catalyst stereocontrol. For instance, the enantiopurity of product **12** would be dependent on the subsequent rate of silylation vs carbonate migration of the intermediate Cu-alkoxide formed after allylcupration leading to catalyst turnover if the allylcupration step was reversible.^14^ Along these lines, and in an effort to improve enantioselection, we reasoned that enantioselectivities may be improved if the rate of trapping of the Cu-alkoxide intermediate formed after allylcupration could be increased relative to carbonate migration. This may be achieved either through an increased silylation rate or by use of protic additives capable of quenching the Cu-alkoxide by protonolysis^17^ (e.g., *t*-BuOH). In this regard, examination of alternate silane reducing agents or reaction solvents led to no improvements.^14^ Use of excess silane (Table 1, entry 8) reduced the amount of **13a** formed but also reduced the enantiopurity of **12a**. Addition of *t*-BuOH as a proton donor generally mitigated the formation of **13** but afforded reduced yields presumably due to competitive protonation of the *N*-allyl(Cu) nucleophile (entries 5 vs 9/10 and 11 vs 12/13). This effect was more pronounced when a more sterically demanding ketone was used (propiophenone (**8b**), entries 11–13), and use of PhCF_3_ as solvent led to improved yields (entry 12 vs 13). The large amounts of **13** formed in the absence of *t*-BuOH with **8b** are consistent with an increased rate of carbonate migration due to an enhanced Thorpe–Ingold effect.

With optimized conditions in hand (Table 1, entries 5 and 13), the ketone scope was examined (Scheme 1). Notably, the Me-group of **8** could be replaced with increased substitution providing products in high enantioselectivities (**12a**–**12c**), which can often be challenging due to the decreased steric bias of the two ketone substituents. *Para*-substitution of the ketone Ph-group generally led to a decrease in enantioselectivities (**12d**–**12j**); however, enantiopurity could be improved through the use of *t*-BuOH as an additive (Method B). Here, electron-poor ketones (**8d**–**e**) afforded good yields whereas electron-rich ketones (**8f**–**i**) provided poor yields with Method B due to incomplete conversion from competitive protonation of the allenamide, likely due to the reduced rate of addition to these less electrophilic ketones. Interestingly, ketones with *meta*-substitution (**8k**–**o**) returned to typical enantioselection levels as was obtained with **8a**; however, addition of *t*-BuOH led to reduced er with the exception of the bromo derivative **12m**. For ketones containing both *meta-* and *para*-substitution, the *para*-trend was stronger affording products with moderate enantioselectivities (**12i,j**). Smaller ketones bearing 5-membered heterocycles (**12p**–**r**) generally afforded good yields and enantioselectivities without the formation of **13** presumably due to increased silylation rates with these less hindered ketones. A cyclic ketone (**8s**) afforded a moderate yield due to large amounts of the linear product being formed. A ketone lacking prochirality (**8t**) afforded reduced enantioselection while **8u** afforded only linear product *l*-**12u**. Finally, *ortho*-substitution was not tolerated providing large amounts of **13** in poor enantioselectivity along with the linear isomer when 2′-methoxyacetophenone was used.

**Scheme 1:**
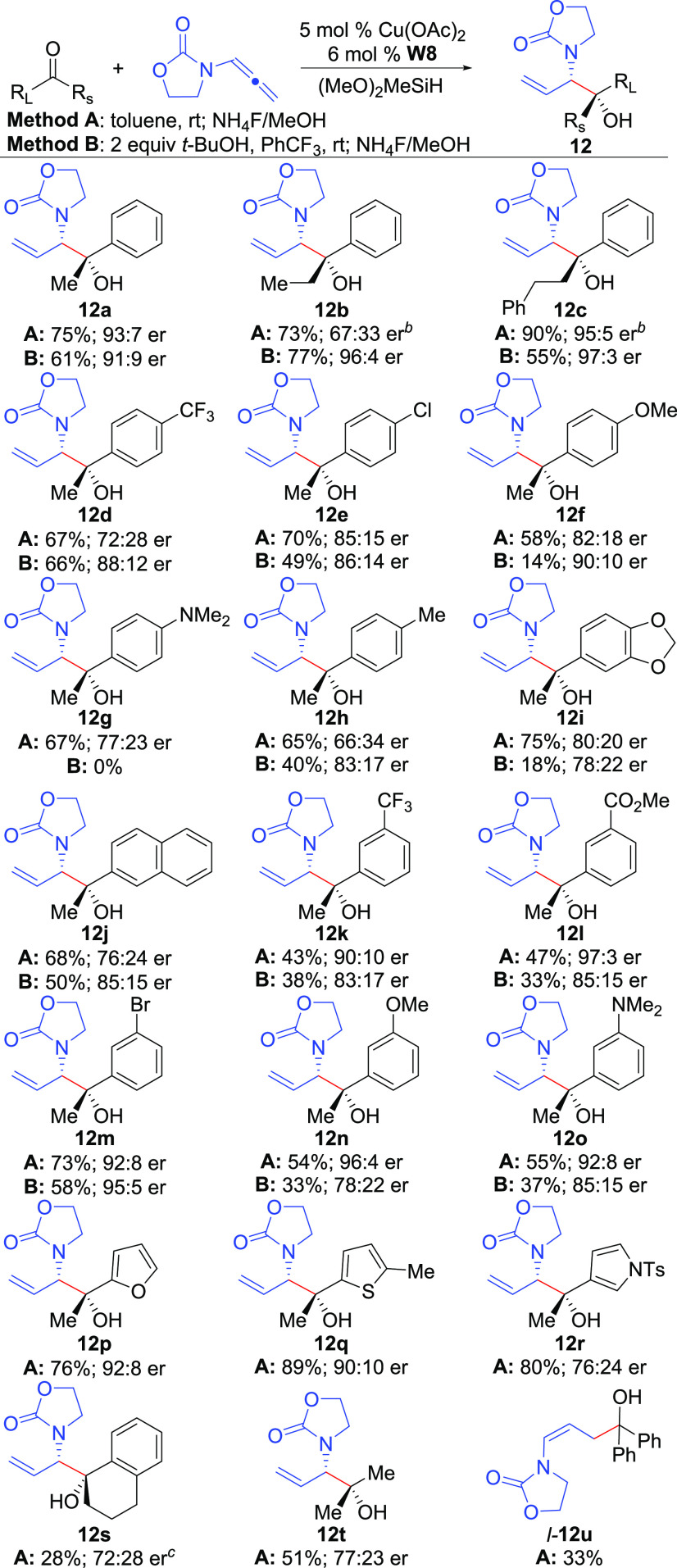
Enantioselective Cu-Catalyzed Reductive Coupling Reaction utilizes 0.25 mmol of ketone; see the Supporting Information. Yield and er of **13** after converting the mixture of **12**/**13** to **13** with NaH. 58% yield of liner isomer also isolated.

A working model to rationalize the observed absolute and relative stereochemistry obtained in these reactions is highlighted in Scheme 2. The quadrant diagram given for the (**W8**)Cu-catalyst (**14**) is proposed based off of a (**W1**)PdCl_2_ crystal structure.^18^ Analysis of the X-ray structure suggests that the northern hemisphere of the complex is sterically more encumbered over the southern hemisphere due to the axial-like orientation of the two Ph-groups. When this information is applied to **14**, a tetrahedral geometry is expected.^11−13^ Furthermore, replacement of the PPh_2_-group of **W1** with the PCy_2_ moiety would result in the eastern hemisphere of **14** having increased steric hindrance relative to the western hemisphere containing the P(Ar)_2_ group. These effects create the quadrant diagram shown for **14** suggesting the southwest quadrant as the most “open.” Mechanistically,^10,11^ hydrocupration of allenamide **11** is expected to provide linear oxazolidinone-substituted allyl ligands that may afford an equilibrating mixture of *E* and *Z* isomers due to inhibition of oxazolidinone complexation to Cu by the chelating nature of **W8**.^19^ However, the *Z*-isomer, as in **15**, may lead to an energetically lower pathway since the oxazolidinone group resides in the most open quadrant. Additionally, hydrocupration of **11** from (**W8**)Cu–H complex **14** would be expected to have the smaller hydride ligand in the northern hemisphere of the catalyst (L_a_ = H) with the allenamide approaching from the south (i.e., L_b_) to minimize steric strain. From **15**, subsequent complexation of the ketone electrophile and nucleophilic addition to the *re*-face occurs preferentially since the large substituent (R_L_) resides in the less sterically hindered northwestern quadrant over the P(Ar)_2_ group and the small substituent (R_s_) in the more sterically hindered northeast quadrant over the P-Cy-substituent (**TS-1** quadrant-view). A side-view of **TS-1** shows this pathway to be chairlike^10,11,13^ having the R_L_-group of the ketone pseudoequatorial. This analysis correctly predicts the observed stereochemical outcome. These arguments assume that stereoinduction is controlled by ketone allylcupration. However, the overall enantioselectivity obtained will be a function of the relative rates of stereoisomers in the allylcupration event and the subsequent silylation or carbonate migration steps of **16** for catalyst turnover due to the reversibility of the allylcupration step. This phenomenon accounts for the variability in enantioselectivity obtained when utilizing diverse ketone electrophiles (Scheme 1). Furthermore, when *t*-BuOH is added, an additional catalyst turnover event through protonolysis of **16**(17,20) is possible that can affect the stereoconvergence of **16** resulting in modulation of the enantioinduction in these processes.

**Scheme 2:**
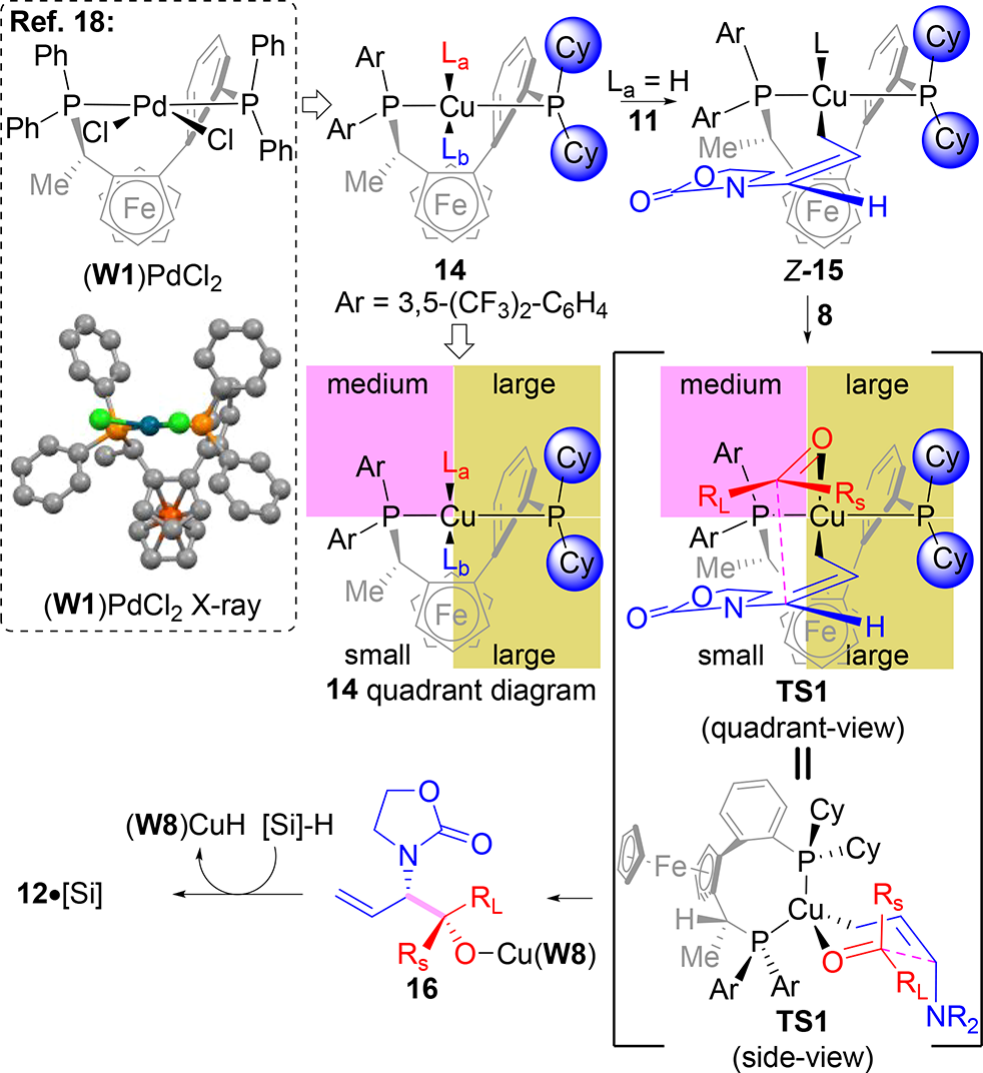
Working Mechanistic Stereochemical Model

To gain experimental evidence for the reversibility of ketone allylcupration, we attempted to regenerate intermediate **16** from **12a** by treatment with an LCuO^*t*^Bu catalyst, but only rearrangement product **13a** was observed.^14^ Therefore, *b*-**17** was prepared using the described methodology and an analogous experiment was performed utilizing (**W8**)CuO^*t*^Bu prepared *in situ* from CuI, KO^*t*^Bu, and **W8** (Scheme 3a). Gratifyingly, ketone **8a** was observed in addition to the linear allylation product (*l*-**17**) and protonation products **18** and **19**. Additionally, recovered *b*-**17** had reduced enantiopurity. Together, these results offer strong evidence in support of a reversible ketone allylcupration in Cu-catalyzed reductive coupling reactions of allenamides.^10,11,14^

**Scheme 3:**
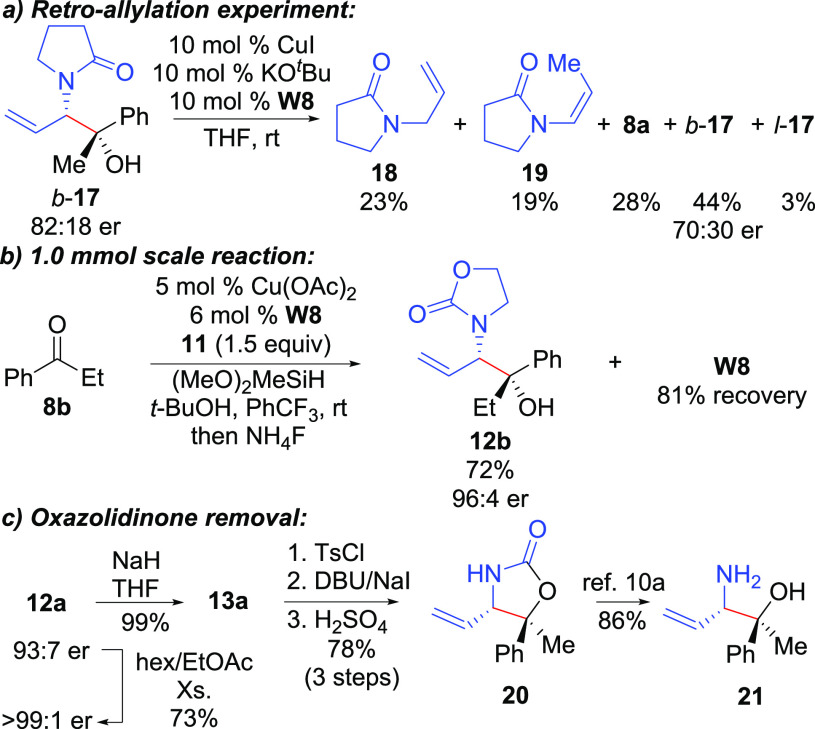
Mechanistic Experiments and Applications

In regards to the synthetic utility of the present methodology, the process was scaled to 1.0 mmol scale providing **12b** in good yield with high enantiopurity as a single diastereomer, and **W8** could be recovered and recycled with identical results (Scheme 3b). Reaction products **12** were generally highly crystalline and could be recrystallized to single enantiomers.^14^ Unmasking of the amino group of **12a** was achieved by carbonate migration to **13a** followed by a three-step telescoped sequence performed without purification of intermediates through tosylation, elimination with DBU/NaI,^21^ and hydrolysis of the resultant enamide to afford **20** (Scheme 3c). Aminoalcohol **21** is then obtained from hydrolysis of **20**.^10a^

In conclusion, we have disclosed an enantio- and diastereoselective aminoallylation of ketones by Cu-catalyzed reductive coupling to access useful chiral protected 1,2-aminoalcohols. Identification of an unprecedented reversible ketone addition step that appears to be unique to *N*-carbamoyl substituted aminoallylation intermediates derived from the hydrometalation of allenamides having important implications on asymmetric induction was reported. Future investigation into the unique reactivity of these systems for asymmetric synthesis is ongoing.

## Supporting Information Available

Detailed experimental procedures, compound characterization data, chiral HPLC traces, and copies of NMR spectra (PDF)
