# Supplementary material for: Synthesis of 1,2-Aminoalcohols through Enantioselective Aminoallylation of Ketones by Cu-Catalyzed Reductive Coupling
Source: Org Lett. 2021 Aug 4;23(16):6444–9. doi: 10.1021/acs.orglett.1c02258 (PMC8384047; doi:10.1021/acs.orglett.1c02258)
Supplement: Supplementary file 1 — ol1c02258_si_001.pdf [file ol1c02258_si_001.pdf]

# Supporting Information

## Synthesis of 1,2-Aminoalcohols Through Enantioselective Aminoallylation of Ketones by Cu-Catalyzed Reductive Coupling

Raphael K. Klake,[a] Mytia D. Edwards,[a] and Joshua D. Sieber*,[a,b]

[a]*Department of Chemistry, Virginia Commonwealth University, 1001 West Main St., Richmond, VA 23284-3028, USA*

[b]*Medicines for All Institute, VCU, 737 N 5th Street, Richmond, VA 23219, USA*

*Correspondence to: jdsieber@vcu.edu

**Table of Contents:**

General……………………………………………………………………………………S1

Optimization of reaction parameters………………………………………………………….. S2 – S4

General Procedures……………………………………………………………………………S5

Reductive coupling product characterization data....…………………………………………S6 – S13

Reaction performed on 1.0 mmol scale and crystallization of **12a**…………………………….... S14

Oxazolidinone removal........................................................................................................... S15

Determination of absolute stereochemistry……………………………………………………S16 – S17

Chiral HPLC traces of the reductive coupling products…………………………..…………….. S18 – S39

Mechanistic Studies……………………………………………………………………………S40 – S45

References............................................................................................................................ S46

$^{1}$H and $^{13}$C NMR data……………………………………………………………………………S47 – S74

**General.** $^{1}$H NMR spectra were recorded on Bruker 600 MHz spectrometers. Chemical shifts are reported in ppm from tetramethylsilane with the solvent resonance as an internal standard ($CDCl_3$: 7.26 ppm). Data are reported as follows: chemical shift, integration, multiplicity (s = singlet, d = doublet, t = triplet, q = quartet, p = pentet, h = hextet, hept = heptet, br = broad, m = multiplet), and coupling constants (Hz). $^{13}$C NMR was recorded on a Bruker 600 MHz (151 MHz) instrument with complete proton decoupling. Chemical shifts are reported in ppm from tetramethylsilane with the solvent as the internal standard ($CDCl_3$: 77.0 ppm). Chiral HPLC analyses were performed on a Shimadzu Prominence i-series LC-2030C using chiral Daicel columns purchased from Chiral Technologies, Inc. Liquid chromatography was performed using forced flow (flash chromatography) on silica gel purchased from Silicycle. Thin layer chromatography (TLC) was performed on glass-backed 250 μm silica gel $F_{254}$ plates purchased from Silicycle. Visualization was achieved using UV light, a 10% solution of phosphomolybdic acid in EtOH, or potassium permanganate in water followed by heating. HRMS was collected using a Jeol AccuTOF-DART™ mass spectrometer using DART source ionization. All reactions were conducted in oven or flame dried glassware under an inert atmosphere of nitrogen or argon with magnetic stirring unless otherwise noted. Solvents were obtained from VWR as HPLC grade and transferred to septa sealed bottles, degased by Ar sparge, and analyzed by Karl-Fischer titration to ensure water content was ≤ 600 ppm. $Me(MeO)_2SiH$ was purchased from Alfa Aesar and used as received. Allenamides **11** and **23** were prepared in one step as described in the literature.[1] Ketones were purchased from Sigma Aldrich, TCI America, Alfa Aesar,

or Oakwood Chemicals and used as received. Ligands were obtained from the Strem Chemical Company. All other materials were purchased from VWR, Sigma Aldrich, Combi-Blocks, Alfa-Aesar, or Strem Chemical Company and used as received.

**Table SI-1: Ligand Survey in the Cu-Catalyzed Reductive Coupling**[a]

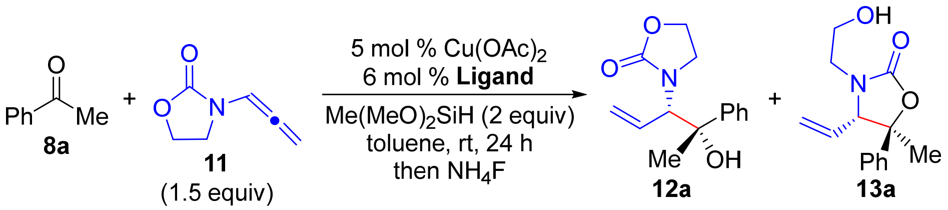


| Entry | Ligand | % y **12a**[b] | dr **12a**[c] | er **12a**[d] | **12a**:**13a**[c] | *b*:*l*[c,e] |
|---|---|---|---|---|---|---|
| 1 | **L1** | 20 | >99:1 | 46:53 | >99:1 | >99:1 |
| 2 | **L2** | 55 | >99:1 | 51:42 | 87:13 | >99:1 |
| 3 | **L3** | 69 | >99:1 | 24:76 | >99:1 | 90:10 |
| 4 | (*S,S,S*)-Feringa | 38 | >99:1 | 57:43 | >99:1 | 76:24 |
| 5 | (*S,S*)-Phenyl-BPE | 64 | >99:1 | 20:80 | 89:11 | >99:1 |
| 6 | (*S,S*)-Ethyl-BPE | 67 | >99:1 | 25:75 | 90:10 | 87:11 |
| 7 | (*R,R*)-QuinoxP* | 63 | >99:1 | 82:18 | 81:19 | 93:7 |
| 8 | (*R,R*)-DuPhos | 81 | >99:1 | 81:19 | 89:11 | >99:1 |
| 9 | (*R,R,S,S*)-DuanPhos | 82 | >99:1 | 18:82 | 84:16 | >99:1 |
| 10 | (*R*)-BINAP | 82 | >99:1 | 18:82 | 83:17 | >99:1 |
| 11 | (*R*)-Tol-BINAP | 57 | >99:1 | 77:23 | >99:1 | 81:19 |
| 12 | (*R*)-DM-BINAP | 45 | >99:1 | 69:31 | >99:1 | >99:1 |
| 13 | (*R*)-SEGPHOS | 51 | >99:1 | 30:70 | 86:14 | >99:1 |
| 14 | (*R*)-DTBM-SEGPHOS | 26 | >99:1 | 68:32 | 82:18 | >99:1 |
| 15 | (*R*)-DM-SEGPHOS | 50 | >99:1 | 69:31 | 86:14 | >99:1 |
| 16 | **J-6** | 58 | >99:1 | 15:85 | 91:9 | >99:1 |
| 17 | **J-7** | 70 | >99:1 | 18:82 | >99:1 | >99:1 |
| 18 | **J-9** | 51 | >99:1 | 39:61 | 81:19 | >99:1 |
| 19 | **J-11** | 60 | >99:1 | 28:72 | 78:22 | >99:1 |
| 20 | **M-1** | 62 | >99:1 | 58:42 | >99:1 | >99:1 |
| 21 | **M-2** | 68 | >99:1 | 37:63 | 87:13 | >99:1 |
| 22 | **M-9** | 55 | >99:1 | 60:40 | >99:1 | >99:1 |
| 23 | **W-1** | 13 | >99:1 | 70:30 | 86:14 | >99:1 |
| 24 | **W-2** | 61 | >99:1 | 67:33 | 73:24 | >99:1 |
| 25 | **W-3** | 64 | >99:1 | 57:43 | 90:10 | >99:1 |
| 26 | **W-5** | 15 | >99:1 | 75:25 | 25:75 | >99:1 |
| 27 | **W-6** | 59 | >99:1 | 60:40 | 84:16 | >99:1 |
| 28 | **W-8** | 77 | >99:1 | 93:7 | 81:19 | >99:1 |
| 29 | **W-9** | 52 | >99:1 | 62:38 | 55:45 | >99:1 |

[a]Reaction performed according to the general procedure employing 0.250 mmol of **8a**, 0.375 mmol of **11**, 0.50 mmol of $Me(OMe)_2SiH$ in 0.5 mL of toluene at rt for 24 h. [b]Yield of **12a** determined by quantitative $^1H$ NMR spectroscopy on the unpurified reaction mixture using dimethyl fumarate as the analytical standard. [c]The ratio was determined by $^1H$ NMR spectroscopic analysis on the unpurified reaction mixture. [d]Enantiomeric ratios were determined by chiral HPLC analysis. [e]*b*:*l* refers to the ratio of branched to linear isomers as defined by: (**12a** + **13a**) : *l*-**12a**.

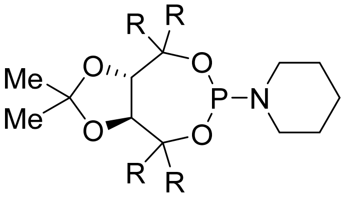

**L1**
R = 3,5-($^t$Bu)$_2$-Ph

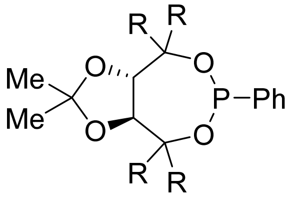

**L2**
R = 3,5-($^t$Bu)$_2$-Ph

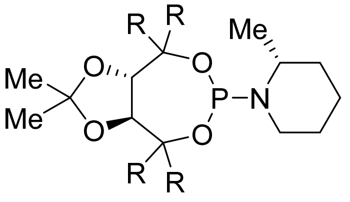

**L3**
R = 3,5-($^t$Bu)$_2$-Ph

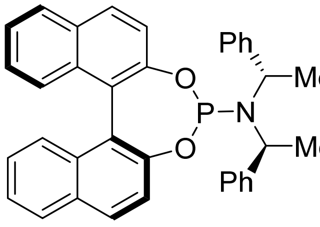

**(*S,S,S*)-Feringa**

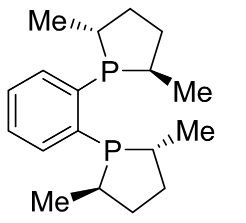

**(*R,R*)-DuPhos**

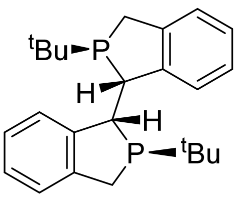

**(*R,R,S,S*)-DuanPhos**

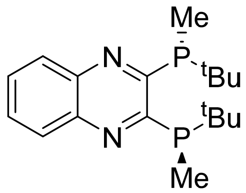

**(*R,R*)-QuinoxP***

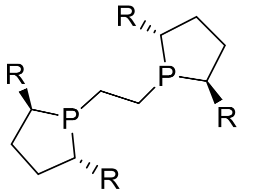

**(*S,S*)-Ph-BPE** R = Ph
**(*S,S*)-Et-BPE** R = Et

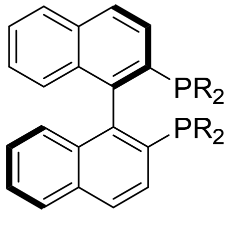

**(*R*)-BINAP** R = Ph
**(*R*)-Tol-BINAP** R = 4-Me-Ph
**(*R*)-DM-BINAP** R = 3,5-(Me)$_2$-Ph

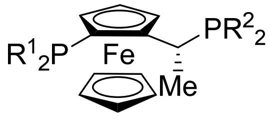

**J-6** $R^1$ = 3,5-($CF_3$)$_2$-Ph, $R^2$ = Cy
**J-7** $R^1$ = 3,5-(Me)$_2$-4-OMe-Ph, $R^2$ = Cy
**J-9** $R^1$ = Cy, $R^2$ = $^t$Bu
**J-11** $R^1$ = 4-$CF_3$-Ph, $R^2$ = $^t$Bu

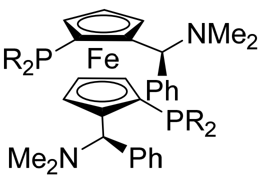

**M-1** R = Ph
**M-2** R = Cy
**M-9** R = 3,5-(Me)$_2$-Ph

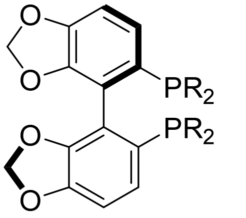

**(*R*)-SEGPHOS** R = Ph
**(*R*)-DM-SEGPHOS** R = 3,5-(Me)$_2$-Ph
**(*R*)-DTBM-SEGPHOS** R = 3,5-($^t$Bu)$_2$-4-OMe-Ph

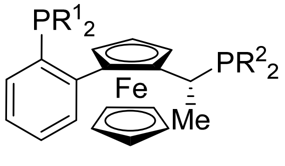

**W-1** $R^1$ = Ph, $R^2$ = 3,5-($CF_3$)$_2$-Ph
**W-2** $R^1$ = Ph, $R^2$ = Ph
**W-3** $R^1$ = Ph, $R^2$ = Cy
**W-5** $R^1$ = 3,5-($CF_3$)$_2$-Ph, $R^2$ = 3,5-(Me)$_2$-4-OMe-Ph
**W-6** $R^1$ = Ph, $R^2$ = 3,5-(Me)$_2$-Ph
**W-8** $R^1$ = 3,5-($CF_3$)$_2$-Ph, $R^2$ = Cy
**W-9** $R^1$ = $R^2$ = 3,5-(Me)$_2$-Ph

**Table SI-2: Reducing Agent Survey in the Cu-Catalyzed Reductive Coupling**[a]

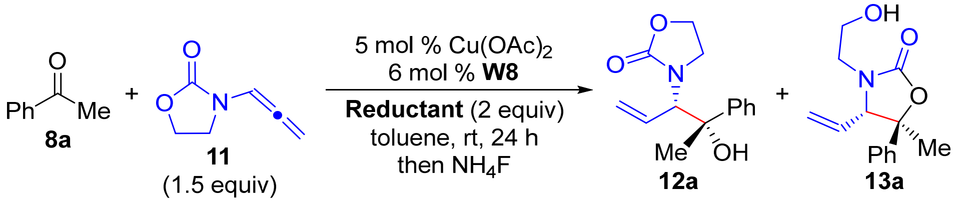


| Entry | Reductant | % y **12a**[b] | dr **12a**[c] | er **12a**[d] | **12a**:**13a**[c] | *b*:*l*[c,e] |
|---|---|---|---|---|---|---|
| 1 | $Me(MeO)_2SiH$ | 77 | >99:1 | 92:8 | 81:19 | >99:1 |
| 2 | $PhSiH_3$ | 55 | >99:1 | 86:14 | >99:1 | 87:13 |
| 3 | PMHS | 0 | - | - | - | - |
| 4 | $Ph_2SiH_2$ | 66 | >99:1 | 80:20 | 94:6 | 86:14 |
| 5 | $(EtO)_3SiH$ | 28 | >99:1 | 84:16 | 77:23 | >99:1 |
| 6 | $PhMe_2SiH$ | 0 | - | - | - | - |
| 7 | (pin)BH | 16 | >99:1 | 70:30 | >99:1 | 63:37 |

[a]Reaction performed according to the general procedure employing 0.250 mmol of **8a**, 0.375 mmol of **11**, 0.50 mmol of reductant in 0.5 mL of toluene at rt for 24 h. [b]Yield of **12a** determined by quantitative $^1H$ NMR spectroscopy on the unpurified reaction mixture using dimethyl fumarate as the analytical standard. [c]The ratio was determined by $^1H$ NMR spectroscopic analysis on the unpurified reaction mixture. [d]Enantiomeric ratios were determined by chiral HPLC analysis. [e]*b*:*l* refers to the ratio of branched to linear isomers as defined by: (**12a** + **13a**) : *l*-**12a**.

**Table SI-3: Solvent Survey in the Cu-Catalyzed Reductive Coupling**[a]

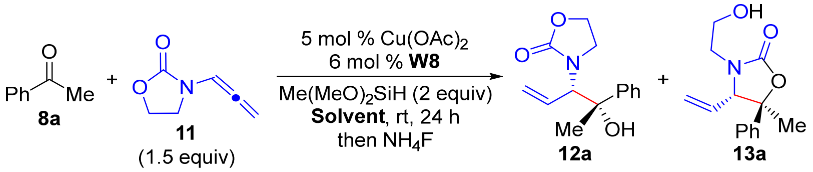


| Entry | Solvent | % y **12a**[b] | dr **12a**[c] | er **12a**[d] | **12a**:**13a**[c] | *b*:*l*[c,e] |
|---|---|---|---|---|---|---|
| 1 | THF | 60 | >99:1 | 89:11 | 79:21 | 96:4 |
| 2 | MTBE | 45 | >99:1 | 90:10 | 64:36 | 90:10 |
| 3 | $CH_2Cl_2$ | 35 | >99:1 | 60:40 | >99:1 | 96:5 |
| 4 | Toluene | 77 | >99:1 | 92:8 | 81:19 | >99:1 |
| 5 | $PhCF_3$ | 62 | >99:1 | 96:4 | 71:29 | >99:1 |
| 6 | Fluorobenzene | 50 | >99:1 | 91:8 | 70:30 | >99:1 |
| 7 | DMF | 77 | >99:1 | 70:30 | >99:1 | 98:2 |
| 8 | 1:1 DMF:Tol. | 73 | >99:1 | 85:15 | 86:13 | >99:1 |
| 9 | 3:7 DMF:Tol. | 33 | >99:1 | 80:20 | 90:10 | >99:1 |
| 10 | 3:7 DMF:$PhCF_3$ | 63 | >99:1 | 86:14 | 90:10 | >99:1 |
| 11 | 3:7 NMP:$PhCF_3$ | 59 | >99:1 | 86:14 | 94:6 | >99:1 |
| 12 | 3:7 DMAc:$PhCF_3$ | 68 | >99:1 | 84:16 | 98:2 | >99:1 |
| 13 | 3:7 tetramethylurea:$PhCF_3$ | 35 | >99:1 | 93:7 | 58:42 | >99:1 |
| 14 | 3:7 *N*,*N*-diisopropylformamide:$PhCF_3$ | 44 | >99:1 | 87:16 | 84:16 | >99:1 |
| 15 | 3:7 *N*-formylpiperidine:$PhCF_3$ | 0 | - | - | - | - |
| 16 | 3:7 DMPU:$PhCF_3$ | 74 | >99:1 | 86:14 | 88:11 | >99:1 |
| 17 | 3:7 propylene carbonate:$PhCF_3$ | 32 | >99:1 | 81:19 | >99:1 | >99:1 |

[a]Reaction performed according to the general procedure employing 0.250 mmol of **8a**, 0.375 mmol of **11**, 0.50 mmol of $Me(OMe)_2SiH$ in 0.5 mL of solvent at rt for 24 h. [b]Yield of **12a** determined by quantitative $^1H$ NMR spectroscopy on the unpurified reaction mixture using dimethyl fumarate as the analytical standard. [c]The ratio was determined by $^1H$ NMR spectroscopic analysis on the unpurified reaction mixture. [d]Enantiomeric ratios were determined by chiral HPLC analysis. [e]*b*:*l* refers to the ratio of branched to linear isomers as defined by: (**12a** + **13a**) : *l*-**12a**.

**General procedure for the branched-selective Cu(W8) catalyzed reductive coupling by Method A.**

To a 20 mL crimp-cap vial with stir-bar in an Ar-filled glove-box was charged 2.3 mg (0.0125 mmol) of $Cu(OAc)_2$ and 14.0 mg (0.0150 mmol) of Walphos-8. Toluene (0.5 mL) was then added, and the mixture was allowed to stir for 10 min. Allenamide **11** (47.0 mg, 0.375 mmol) followed by the ketone (0.250 mmol) was then charged, and the vial was sealed with a crimp-cap septum and removed from the glove-box. The reaction was then cooled in an ice bath, and dimethoxymethylsilane (62 μL, 0.5 mmol) was then charged by syringe *(caution: dimethoxymethylsilane should be handled in a well-ventilated fume hood because it is known to cause blindness. Syringes were quenched with 2M NaOH, gas evolution!, prior to disposal)* The mixture was then allowed to warm to rt and stirred for 24 h. The reaction was then quenched by the addition of 95 mg of NH4F and 1.5 mL of MeOH followed by agitation at rt for 30 min – 1 h. To the mixture was then charged 5 mL of 5% NaHCO3 followed by extraction with $CH_2Cl_2$ (2x4mL). The combined organics were dried with $Na_2SO_4$ and concentrated *in vacuo*. An aliquot of the crude mixture was analyzed by $^1$HNMR spectroscopy to determine the dr and b/l ratio. The crude residue was then purified by flash chromatography on silica gel to afford the desired product. Enantioselectivity was determined by chiral HPLC analysis relative to authentic racemate prepared by the same method using $PCy_3$ as ligand.

**General procedure for the branched-selective Cu(W8) catalyzed reductive coupling by Method B.**

To a 20 mL crimp-cap vial with stir-bar in an Ar-filled glove-box was charged 2.3 mg (0.0125 mmol) of $Cu(OAc)_2$ and 14.0 mg (0.0150 mmol) of Walphos-8. α,α,α-Trifluorotoluene (0.5 mL) was then added, and the mixture was allowed to stir for 10 min. Allenamide **11** (47.0 mg, 0.375 mmol) followed by the ketone (0.250 mmol) and $^t$BuOH (48 μL, 0.500 mmol) was then charged. The vial was then sealed with a crimp-cap septum and removed from the glove-box. The reaction was then cooled in an ice bath, and dimethoxymethylsilane (62 μL, 0.5 mmol) was then charged by syringe *(caution: dimethoxymethylsilane should be handled in a well-ventilated fume hood because it is known to cause blindness. Syringes were quenched with 2M NaOH, gas evolution!, prior to disposal)* The mixture was then allowed to warm to rt and stirred for 24 h. The reaction was then quenched by the addition of 95 mg of NH4F and 1.5 mL of MeOH followed by agitation at rt for 30 min – 1 h. To the mixture was then charged 5 mL of 5% NaHCO3 followed by extraction with $CH_2Cl_2$ (2x4mL). The combined organics were dried with $Na_2SO_4$ and concentrated *in vacuo*. An aliquot of the crude mixture was analyzed by $^1$HNMR spectroscopy to determine the dr and b/l ratio. The crude residue was then purified by flash chromatography on silica gel to afford the desired product. Enantioselectivity was determined by chiral HPLC analysis relative to authentic racemate prepared by the same method using $PCy_3$ as ligand.

**Analytical data for the reductive coupling products**

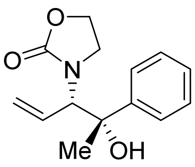

**12a**

**3-((3S,4S)-4-hydroxy-4-phenylpent-1-en-3-yl)oxazolidin-2-one** (**12a**): According to the Method A general procedure, a crude mixture of 80:20 **12a**:**13b** was obtained and purified by silica gel chromatography (eluent: 10 – 50% EtOAc in hexanes) to provide 46.3 mg (75%) of **12a** in 91 wt% purity by quantitative $^{1}$H NMR spectroscopy using dimethylfumarate as analytical standard as an off-white solid as a single diastereomer as a 93:7 mixture of enantiomers (allene rearrangement products *N*-allyl and *N*-propenyl oxazolidin-2-one could not be removed by chromatography). Analytically pure material could be obtained by slurrying the material in 10 vol. of hot 30% EtOAc in hexanes followed by cooling to rt in 76% recovery with >99 er. Absolute and relative stereochemistry was determined by conversion to authentic material (see page S16). R$_f$ = 0.12 (30% EtOAc/hexanes). $^{1}$HNMR ($CDCl_3$, 600 MHz) δ: 7.47 (d, *J* = 7.90 Hz, 2H), 7.34 (t, *J* = 7.30 Hz, 2H), 7.23 – 7.28 (m, 1H), 6.29 (ddd, *J* = 16.40 Hz, *J* = 9.98 Hz, 8.50 Hz, 1H), 5.43 (d, *J* = 10.6 Hz, 1H), 5.36 (dt, *J* = 17.46 Hz, *J* = 1Hz, 1H), 4.29 (br s, 1H), 4.15 (d, *J* = 8.65 Hz, 1H), 4.02 (dtd, *J* = 21.44 Hz, *J* = 8.81 Hz *J* = 7.11 Hz, 2H), 3.53 (td, *J* = 15.61 Hz, *J* = 7.11 Hz, 1H), 3.23 (td, *J* = *1*7.62 Hz, *J* = 7.01 Hz, 1H), 1.53 (s, 3H) ppm. $^{13}$C NMR (151 MHz, $CDCl_3$): δ 158.8, 145.8, 130.7, 128.1, 126.9, 124.5, 120.6, 67.0, 62.6, 45.0, 29.1 ppm. HRMS (DART) *m/z* calcd for $C_{14}H_{18}NO_3$ $[M + H]^+$: 248.1287; Found $[M + H]^+$: 248.1264.

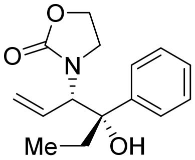

**12b**

**3-((3S,4S)-4-hydroxy-4-phenylhex-1-en-3-yl)oxazolidin-2-one** (**12b**): According to the Method B general procedure, a crude mixture of >99:1 **12b**:**13b** was obtained and purified by silica gel chromatography (eluent: 10 – 50% EtOAc in hexanes) to provide 50.3 mg (77%) of **12b** in 85 wt% purity by quantitative $^{1}$H NMR spectroscopy using dimethylfumarate as analytical standard as an off-white solid as a single diastereomer as a 96:4 mixture of enantiomers (allene rearrangement products *N*-allyl and *N*-propenyl oxazolidin-2-one could not be removed by chromatography). Analytically pure material could be obtained by slurrying the material in 10 vol. of hot 30% EtOAc in hexanes followed by cooling to rt in 81% recovery with >99 er. The stereochemistry was assigned by analogy to that of **12a**. R$_f$ = 0.18 (30% EtOAc/hexanes). $^{1}$HNMR ($CDCl_3$, 600 MHz) δ: 7.43 (d, *J* = 7.70 Hz, 2H), 7.34 (t, *J* = 7.08 Hz, 2H), 7.21-7.27 (m, 1H), 6.27 (ddd, *J* = 17.8 Hz, *J* = 10.3 Hz, *J* = 8.5 Hz, 1H), 5.41 (d, *J* = 10.3 Hz, 1H), 5.36 (d, *J* = 17.3 Hz, 1H), 4.27 (br s, 1H), 4.18 (d, *J* = 8.9 Hz, 1H), 3.98 (t, *J* = 8.6 Hz, 2H), 3.52 (q, 8.0 Hz, 1H), 3.25 (q, *J* = 8.0 Hz, 1.87 – 1.97 (m, 1H), 1.73 – 1.81 (m, 1H), 0.66 (t, *J* = 6.9 Hz, 3H) ppm. $^{13}$C NMR (151 MHz, $CDCl_3$): δ 158.7, 143.2, 130.9, 128.0, 126.7, 125.2, 120.5, 79.5, 66.8, 62.6, 45.0, 33.4, 7.3 ppm. HRMS (DART) *m/z* calcd for $C_{15}H_{19}NO_3$ $[M]^+$: 261.1365; Found $[M]^+$: 261.1332.

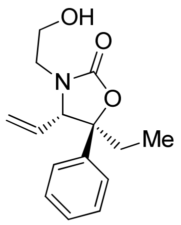

**13b**

**(4S,5S)-5-ethyl-3-(2-hydroxyethyl)-5-phenyl-4-vinyloxazolidin-2-one** (**13b**): According to the Method A general procedure, a crude mixture of 12:88 **12b**:**13b** was obtained and purified by silica gel chromatography (eluent: 0 – 30% EtOAc in $CH_2Cl_2$) to provide 45.8 mg (70%) of **13b** as a thick glass as a single diastereomer as a 67:33 mixture of enantiomers. The stereochemistry was assigned by analogy to that of **12a**. R$_f$ = 0.31 (30% EtOAc/ $CH_2Cl_2$). $^{1}$HNMR ($CDCl_3$, 600 MHz) δ: 7.33 – 7.41 (m, 4H), 7.31 (t, *J* = 6.6 Hz, 1H), 5.95 (dt, *J* = 17.1 Hz, 9.5 Hz, 1H, 5.53 (d, *J* = 10.3 Hz, 1H), 5.44 (d, *J* = 17.2 Hz, 1H), 4.21 (d, *J* = 9.5 Hz, 1H), 3.62 – 3.74 (m, 2H), 3.47 (dt, *J* = 14.8 Hz, 4.8 Hz, 1H), 3.17 (dt, 15.1 Hz, 5.2 Hz, 1H), 2.22 (br s, 1H), 2.07 (dq, *J* = 14.4 Hz, 6.1 Hz, 1H), 1.87 (dq, 15.4 Hz, 7.0 Hz, 1H), 0.76 (t, *J* = 7.2 Hz, 3H) ppm. $^{13}$C NMR (151 MHz, $CDCl_3$): δ 158.2, 142.3, 132.3, 128.7, 127.8, 124.3, 123.2, 86.3, 71.5, 61.0, 45.1, 29.5, 7.6 ppm. HRMS (DART) *m/z* calcd for $C_{15}H_{19}NO_3$ $[M]^+$: 261.1365; Found $[M]^+$: 261.1336.

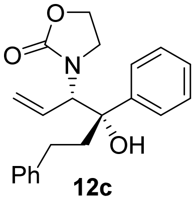
12c

**3-((3S,4S)-4-hydroxy-4,6-diphenylhex-1-en-3-yl)oxazolidin-2-one** (**12c**): According to the Method B general procedure, a crude mixture of >99:1 **12c**:**13c** was obtained and purified by silica gel chromatography (eluent: 10 – 50% EtOAc in hexanes) to provide 42.1 mg (55%) of **12c** in 90 wt% purity by quantitative $^{1}$H NMR spectroscopy using dimethylfumarate as analytical standard as an off-white solid as a single diastereomer as a 97:3 mixture of (allene rearrangement products *N*-allyl and *N*-propenyl 4-phenyloxazolidin-2-one could not be removed by chromatography). Analytically and diastereomerically pure material could be obtained by slurrying the material in 10 vol. of hot 30% EtOAc in hexanes followed by cooling to rt in 76% recovery with >99 er. The stereochemistry was assigned by analogy to that of **12a**. $R_f$ = 0.21 (30% EtOAc/hexanes). $^{1}$HNMR ($CDCl_3$, 600 MHz) δ: 7.48 (d, *J* = 7.9 Hz, 2H), 7.38 (t, *J* = 7.42 Hz, 2H), 7.27 (t, *J* = 6.7 Hz, 1H), 7.21 (t, *J* = 7.7 Hz, 2H), 7.12 (t, *J* = 7.7 Hz, 1H), 7.03 (d, *J* = 7.7 Hz, 2H), 6.29 (ddd, *J* = 17.4 Hz, *J* = 10.6 Hz, *J* = 9.1 Hz, 1H), 5.39 (d, *J* = 9.31 Hz, 1H), 5.31 (d, *J* = 17.2 Hz, 1H), 4.80 (br s, 1H), 4.05 ( d, *J* = 9.3 Hz, 1H), 4.00 (q, *J* = 7.0 Hz, 1H), 3.94 (q, *J* = 8.8 Hz, 1H), 3.50 (q, J = 8.5 Hz, 1H), 3.16 (q, *J* = 6.8 Hz, 1H), 2.60 (td, *J* = 12.9 Hz, *J* = 4.9 Hz, 1H), 2.21 (td, *J* = 12.3 Hz, *J* = 5.0 Hz, 1H), 2.13 ( td, *J* = 12.7 Hz, *J* = 3.6 Hz, 1H), 1.99 (td, *J* = 12.7 Hz, *J* = 4.3 Hz, 1H) ppm. $^{13}$C NMR (151 MHz, $CDCl_3$): δ 158.9, 143.5, 142.1, 130.6, 128.3, 128.2, 126.9, 125.7, 125.1, 120.8, 79.4, 67.8, 62.8, 45.6, 42.7, 29.5 ppm. HRMS (DART) *m/z* calcd for $C_{21}H_{24}NO_3$ $[M + H]^+$: 338.1756; Found $[M + H]^+$: 338.1735.

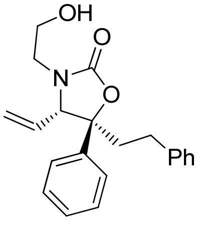
13c

**(4S,5S)-3-(2-hydroxyethyl)-5-phenethyl-5-phenyl-4-vinyloxazolidin-2-one** (**13c**): According to the Method A general procedure, a crude mixture of 14:86 **12c**:**13c** was obtained and purified by silica gel chromatography (eluent: 0 – 30% EtOAc in $CH_2Cl_2$) to provide 66.7 mg (78%) of **13c** as a thick glass as a single diastereomer as a 95:5 mixture of enantiomers. The stereochemistry was assigned by analogy to that of **12a**. $R_f$ = 0.35 (30% EtOAc/$CH_2Cl_2$). $^{1}$HNMR ($CDCl_3$, 600 MHz) δ: 7.40 – 7.46 (m, 4H), 7.33 – 7.39 (m, 1H), 7.23 (t, *J* = 7.8 Hz, 2H), 7.16 (t, *J* = 7.30 Hz, 1H), 7.06 (d, *J* = 7.30 Hz, 2H), 5.92 (dt, *J* = 16.6 Hz, 10.3 Hz, 1H), 5.50 (d, *J* = 10.3 Hz, 1H), 5.44 (d, *J* = 17.5 Hz, 1H), 4.23 (d, *J* = 9.7 Hz, 1H), 3.63 – 3.75 (m, 2H) 3.44 (ddd, *J* = 16.9 Hz, *J* = 7.2 Hz, 4.16 Hz, 1H), 3.18 (ddd, *J* = 17.2, *J* = 7.2 Hz, 3.8 Hz, 1H), 2.7 (td, *J* = 12.8 Hz, *J* = 5.1 Hz, 1H), 2.30 (dt, *J* = 12.6 Hz, 4.6 Hz, 1H), 2.08 – 2.22 (m, 3H) ppm. $^{13}$C NMR (151 MHz, $CDCl_3$): δ 158.0, 142.3, 141.5, 132.2, 128.9, 128.5, 128.3, 128.0, 126.0, 126.1, 123.4, 85.6, 71.7, 60.9, 45.1, 39.0, 29.6 ppm. HRMS (DART) *m/z* calcd for $C_{21}H_{24}NO_3$ $[M + H]^+$: 338.1756; Found $[M + H]^+$: 338.1735.

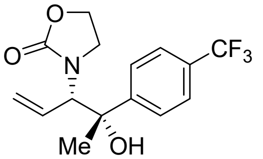
12d

**3-((3S,4S)-4-hydroxy-4-(4-(trifluoromethyl)phenyl)pent-1-en-3-yl)oxazolidin-2-one** (**12d**): According to the Method B general procedure, the crude mixture of >99:1 **12d**:**13d** was obtained and purified by silica gel chromatography (eluent: 0 – 20% EtOAc in $CH_2Cl_2$) to provide 52.0 mg (66%) of **12d** as a thick clear oil as a single diastereomer as a 88:12 mixture of enantiomers. The stereochemistry was assigned by analogy to that of **12a**. $R_f$ = 0.49 (10% EtOAc/$CH_2Cl_2$). $^{1}$HNMR ($CDCl_3$, 600 MHz) δ: 7.58 – 7.63 (m, 4H), 6.26 (ddd, *J* = 17.7 Hz, *J* = 9.9 Hz, *J* = 8.7 Hz, 1H), 5.46 (d, *J* = 10.2 Hz, 1H), 5. 38 (d, *J* = 17.4 Hz, 1H), 4.64 (s, 1H), 4.17 (d, *J* = 8.6 Hz, 1H), 4.0 – 4.1 (m, 2H), 3.56 (q, *J* = 8.4 Hz, 1H), 3.28 (q, *J* = 7.5 Hz, 1H), 1.51 (s, 3H) ppm. $^{13}$C NMR (151 MHz, $CDCl_3$): δ 158.9, 150.1, 130.1, 129.3, 129.0, 125 (q, $^{3}J_{CF}$ = 3.5), 125.0, 121.2, 76.8, 66.9, 62.7, 40.1, 29.3 ppm. $^{19}$F NMR (565 MHz, $CDCl_3$): – 62.4 ppm. HRMS (DART) *m/z* calcd for $C_{18}H_{24}F_3NO_5Si$ $[M]^+$: 419.1376; Found $[M]^+$: 419.1376.

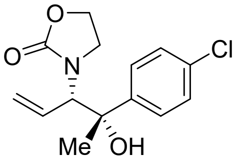


**12e**

**3-((3S,4S)-4-(4-chlorophenyl)-4-hydroxypent-1-en-3-yl)oxazolidin-2-one (12e)**: According to the Method A general procedure, a crude mixture of 76:24 **12e**:**13e** was obtained and purified by silica gel chromatography (eluent: 10 – 50% EtOAc in hexanes) to provide 49.2 mg (70%) of **12e** in 89 wt% purity by quantitative $^{1}$H NMR spectroscopy using dimethylfumarate as analytical standard as a white solid as a single diastereomer as a 85:15 mixture of enantiomers (allene rearrangement products *N*-allyl and *N*-propenyl oxazolidin-2-one could not be removed by chromatography). Analytically pure material could be obtained by slurrying the material in 10 vol. of hot 30% EtOAc in hexanes followed by cooling to rt in 65% recovery with 95:5 er. The stereochemistry was assigned by analogy to that of **12a**. $R_f$ = 0.14 (30% EtOAc/hexanes) $^{1}$HNMR ($CDCl_3$, 600 MHz) δ: 7.42 (d, *J* = 8.7 2H), 7.32 (d, *J* = 8.7 Hz, 1H), 6.25 (ddd, *J* = 18.4 Hz, *J* = 17.4 Hz, *J* = 9.0 Hz 1H), 5.44 (d, *J* = 10.4 Hz, 1H), 5.36 (d, *J* = 17.3 Hz, 1H), 4.43 (s, 1H), 4.05 - 4.15 (m, 3H), 3.55 (dt, *J* = 7.6 Hz, *J* = 8.6 Hz, 1H), 3.27 (dt, *J* = 7.9 Hz, *J* = 7.0 Hz, 1H), 1.50 (s, 3H) ppm. $^{13}$C NMR (151 MHz, $CDCl_3$): δ 158.9, 144.5, 132.8, 130.4, 128.4, 126.1, 121.1, 76.8, 67.0, 62.8, 45.2, 29.3 ppm. HRMS (DART) *m/z* calcd for $C_{14}H_{17}ClNO_3$ [M + H]$^+$: 282.0897; Found [M + H]$^+$: 282.0883.

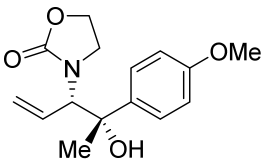


**12f**

**3-((3S,4S)-4-hydroxy-4-(4-methoxyphenyl)pent-1-en-3-yl)oxazolidin-2-one (12f)**: According to the Method A general procedure, a crude mixture of 88:12 **12f**:**13f** was obtained and purified by silica gel chromatography (eluent: 10 – 50% EtOAc in hexanes) to provide 40.2 mg (58%) of **12f** in 91 wt% purity by quantitative $^{1}$H NMR spectroscopy using dimethylfumarate as analytical standard as a white solid as a single diastereomer as a 82:18 mixture of enantiomers (allene rearrangement products *N*-allyl and *N*-propenyl oxazolidin-2-one could not be removed by chromatography). Analytically and diastereomerically pure material could be obtained by slurrying the material in 10 vol. of hot 30% EtOAc in hexanes followed by cooling to rt in 62% recovery with >99:1 er. The stereochemistry was assigned by analogy to that of **12a**. $R_f$ = 0.10 (30% EtOAc/hexanes). $^{1}$HNMR ($CDCl_3$, 600 MHz) δ: 7.38 (d, *J* = 7.7 Hz, 2H), 6.88 (d, *J* = 9.0 Hz, 2H), 6.26 (ddd, J =. 17.5 Hz, *J* = 10.5, *J* = 8.9 Hz, 1H), 5.42 (d, *J* = 10.1 Hz, 1H), 5.35 (d, *J* = 17.1 Hz, 1H), 4.10 – 4.17 (m, 2H), 4.05 (t, *J* = 8.2 Hz, 2H), 3.52 (dt, *J* = 8.4 Hz, *J* = 7.8 Hz, 1H), 3.27 (dt, *J* = 8.2 Hz, *J* = 8.4 Hz, 1H), 1.50 (s, 3H) ppm. $^{13}$C NMR (151 MHz, $CDCl_3$): δ 158.8, 158.4, 137.9, 130.8, 125.8, 120.6, 114.5, 76.8, 67.0, 62.7, 55.2, 45.0, 29.4 ppm. HRMS (DART) *m/z* calcd for $C_{15}H_{20}NO_4$ [M + H]$^+$: 278.1392; Found [M + H]$^+$: 278.1379.

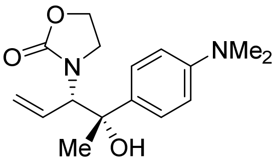


**12g**

**3-((3S,4S)-4-(4-(dimethylamino)phenyl)-4-hydroxypent-1-en-3-yl)oxazolidin-2-one (12g)**: According to the Method A general procedure, a crude mixture of >99:1 **12g**:**13g** was obtained and purified by silica gel chromatography (eluent: 0 – 30% EtOAc in $CH_2Cl_2$) to provide 48.6 mg (67%) of **12g** as thick glass as a single diastereomer as a 77:23 mixture of enantiomers and >99 mixture of **12g**:**13g**. The stereochemistry was assigned by analogy to that of **12a**. $R_f$ = 021 (10% EtOAc/$CH_2Cl_2$). $^{1}$HNMR ($CDCl_3$, 600 MHz) δ: 7.32 (d, *J* = 8.9 Hz, 2H), 6.72 (d, *J* = 7.7, 2H), 6.26 (ddd, *J* = 17.6 Hz, *J* = 10.5 Hz, *J* = 8.4 Hz, 1H), 5.41 (d, *J* = 10.4 Hz, 1H), 5.34 (d, *J* = 17.3 Hz, 1H), 4.17 (d, *J* = 8.4 Hz, 1H), 4.05 (t, *J* = 7.7 Hz, 1H), 3.51 (dt, *J* = 8.0 Hz, *J* = 8.4 Hz, 1H), 3.28 (dt, *J* = 8.2 Hz, *J* = 8.6 Hz, 1H), 2.95 (s, 6H), 1.51 (s, 3H) ppm. $^{13}$C NMR (151 MHz, $CDCl_3$): δ 158.7, 143.2, 130.9, 128.0, 126.7, 125.2, 120.5, 79.5, 66.8, 62.6, 45.0, 33.4, 7.3 ppm. HRMS (DART) *m/z* calcd for $C_{16}H_{23}N_2O_3$ [M + H]$^+$: 291.1709; Found [M + H]$^+$: 291.1714.

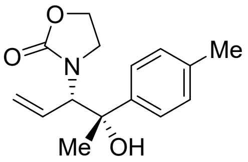
12h

**3-((3S,4S)-4-hydroxy-4-(p-tolyl)pent-1-en-3-yl)oxazolidin-2-one** (**12h**): According to the Method B general procedure, a crude mixture of >99:1 **12h**:**13h** was obtained and purified by silica gel chromatography (eluent: 10 – 50% EtOAc in hexanes) to provide 26.1 mg (40%) of **12h** in 88 wt% purity by quantitative $^1$H NMR spectroscopy using dimethylfumarate as analytical standard as a white solid as a single diastereomer as a 83:17 mixture of enantiomers (allene rearrangement products *N*-allyl and *N*-propenyl oxazolidin-2-one could not be removed by chromatography). Analytically pure material could be obtained by slurrying the material in 10 vol. of hot 30% EtOAc in hexanes followed by cooling to rt in 77% recovery with 91:9 er. The stereochemistry was assigned by analogy to that of **12a**. $R_f$ = 0.16 (30% EtOAc/hexanes). $^1$HNMR ($CDCl_3$, 600 MHz) δ: 7.32 (d, *J* = 8.9 Hz, 2H), 6.72 (d, *J* = 7.7, 2H), 6.26 (ddd, *J* = 17.6 Hz, *J* = 10.5 Hz, *J* = 8.4 Hz, 1H), 5.41 (d, *J* = 10.4 Hz, 1H), 5.34 (d, *J* = 17.3 Hz, 1H), 4.17 (d, *J* = 8.4 Hz, 1H), 4.05 (t, *J* = 7.7 Hz, 1H), 3.51 (dt, *J* = 8.0 Hz, *J* = 8.4 Hz, 1H), 3.28 (dt, *J* = 8.2 Hz, *J* = 8.6 Hz, 1H), 2.95 (s, 6H), 1.51 (s, 3H) ppm. $^{13}$C NMR (151 MHz, $CDCl_3$): δ 158.7, 142.7, 136.4, 130.8, 128.8, 124.4, 120.5, 76.8, 66.8, 62.6, 44.9, 29.2, 20.9 ppm. HRMS (DART) *m/z* calcd for $C_{16}H_{23}N_2O_3$ $[M + H]^+$: 291.1709; Found $[M + H]^+$: 291.1714.

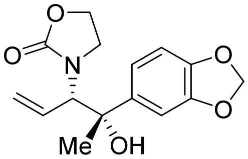
12i

**3-((3S,4S)-4-(benzo[d][1,3]dioxol-5-yl)-4-hydroxypent-1-en-3-yl)oxazolidin-2-one** (**12i**): According to the Method A general procedure, a crude mixture of 88:12 **12i**:**13i** was obtained and purified by silica gel chromatography (eluent: 10 – 50% EtOAc in hexanes) to provide 54.6 mg (75%) of **12i** in 87 wt% purity by quantitative $^1$H NMR spectroscopy using dimethylfumarate as analytical standard as a white solid as a single diastereomer as a 80:20 mixture of enantiomers (allene rearrangement products *N*-allyl and *N*-propenyl oxazolidin-2-one could not be removed by chromatography). Analytically and diastereomerically pure material could be obtained by slurrying the material in 10 vol. of hot 30% EtOAc in hexanes followed by cooling to rt in 75% recovery with 90:10 er. The stereochemistry was assigned by analogy to that of **12a**. $R_f$ = 0.12 (30% EtOAc/hexanes). $^1$HNMR ($CDCl_3$, 600 MHz) δ: 6.99 (s, 1H), 6.93 (dd, *J* = 8.18 Hz, *J* = 1.1 Hz, 1H), 6.78 (d, *J* = 8.30 Hz, 1H), 6.25 (ddd, *J* = 17.3 Hz, J =. 10.8 Hz, *J* = 9.3 Hz, 1H), 5.96 (s, 2H), 5.42 (d, *J* = 10.3 Hz, 1H), 5.35 (d, *J* = 17.3 Hz, 1H), 4.21 (br, s, 1H), 4.05 - 4.14 (m, 3H), 3.6 (q, *J* = 8.4, 1H), 3.32 (q, *J* = 8.4 Hz, 3H), 1.49 (s, 3H) ppm. $^{13}$C NMR (151 MHz, $CDCl_3$): δ 158.9, 147.6, 146.4, 140.1, 130.4, 120.8, 117.8, 107.9, 105.6, 101.1, 76.9, 67.1, 62.7, 45.1, 29.5 ppm. HRMS (DART) *m/z* calcd for $C_{15}H_{17}NO_5$ $[M + H]^+$: 291.1107; Found $[M]^+$: 291.1123.

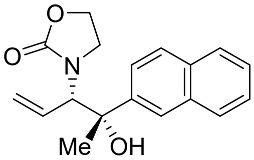
12j

**3-((3S,4S)-4-hydroxy-4-(naphthalen-2-yl)pent-1-en-3-yl)oxazolidin-2-one** (**12j**): According to the Method B procedure, a crude mixture of >99:1 **12j**:**13j** was obtained and purified by silica gel chromatography (eluent: 10 – 50% EtOAc in hexanes) to provide 37.1 mg (50%) of **12j** in 88 wt% purity by quantitative $^1$H NMR spectroscopy using dimethylfumarate as analytical standard as a white solid as a single diastereomer as a 85/15 mixture of enantiomers and >99:1 mixture of **12j**:**13j** (allene rearrangement products *N*-allyl and *N*-propenyl oxazolidin-2-one could not be removed by chromatography). Analytically and diastereomerically pure material could be obtained by slurrying the material in 10 vol. of hot 30% EtOAc in hexanes followed by cooling to rt in 80% recovery with 82:17 er. The stereochemistry was assigned by analogy to that of **12a**. $R_f$ = 0.16 (30% EtOAc/hexanes). $^1$HNMR ($CDCl_3$, 600 MHz) δ: 8.00 (s, 1H), 7.75 -8.86 (m, 3H), 7.4 – 7.50 (m, 3H), 6.31 (ddd, *J* = 17.4 Hz, *J* = 10.4 Hz, *J* = 9.19 Hz, 1H), 5.43 (d, *J* = 10.2 Hz, 1H), 5.37 (d, *J* = 17.27 Hz, 1H), 4.59 (s, 1H), 4.26 (d, *J* = 8.5 Hz, 1H), 3.94 (q, J= 9.0 Hz, 1H), 3.89 (q, *J* = 8.4 Hz, 1H), 3.59 (q, *J* = 8.81 Hz, 1H), 3.22 (q, *J* = 8.81 Hz, 1H) 1.56 (s, 3H) ppm. $^{13}$C NMR (151 MHz, $CDCl_3$): δ 158.9, 143.3, 133.1, 132.3, 128.2, 127.8, 127.4,

126.1, 125.8, 123.3, 122.9, 120.7, 76.9, 66.9, 62.6, 45.1, 29.3 ppm. HRMS (DART) *m/z* calcd for $C_{18}H_{20}NO_3$ [M + H]$^+$: 298.1443; Found [M + H]$^+$: 298.1435.

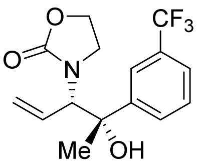

**12k**

**3-((3S,4S)-4-hydroxy-4-(3-(trifluoromethyl)phenyl)pent-1-en-3-yl)oxazolidin-2-one** (**12k**): According to the Method A general procedure, a crude mixture of 60:40 **12k**:**13k** was obtained and purified by silica gel chromatography (eluent: 0 – 20% EtOAc in $CH_2Cl_2$) to provide 33.9 mg (43%) of **12k** as an oil as a single diastereomer as a 90:10 mixture of enantiomers. The stereochemistry was assigned by analogy to that of **12a**. $R_f$ = 0.19 (10% EtOAc/ $CH_2Cl_2$). $^1$HNMR ($CDCl_3$, 600 MHz) δ: 7.74 (s, 1H), 7.69 (d, *J* = 8.00 Hz, 1H), 7.51 (d, *J* = 8.24 Hz, 1H), 7.47 (t, *J* = 7.6 Hz, 1H), 6.25 (ddd, *J* = 17.3 Hz, *J* = 10.8 Hz, *J* = 9.7 Hz, 1H), 5.45 (d, *J* = 9.9 Hz, 1H), 5.38 (d, *J* = 17.0 Hz, 1H), 4.55 (br s, 1H), 4.18 (d, *J* = 8.4 Hz, 1H), 4.00 – 4.07 (m, 2H), 3.60 (q, 8.4 Hz, 1H), 3.28 (q, *J* = 7.5 Hz, 1H), 1.52 (s, 3H) ppm. $^{13}$C NMR (151 MHz, $CDCl_3$): δ 158.8, 147.1, 130.3 (q, $^2J_{CF}$ = 33 Hz), 129.4 (q, $^1J_{CF}$ = 274 Hz), 128.8, 128.3, 123.7 (q, $^3J_{CF}$ = 4.2 Hz), 123.7 (q, $^3J_{CF}$ = 4.6 Hz), 121.3, 118.6, 66.7, 62.7, 61.7, 44.9, 29.1 ppm. $^{19}$F NMR (565 MHz, $CDCl_3$): – 62.5 ppm. HRMS (DART) *m/z* calcd for $C_{18}H_{24}F_3NO_5Si$ [M]$^+$: 419.1376; Found [M]$^+$: 419.1376.

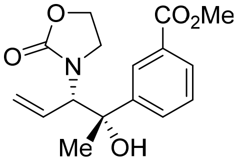

**12l**

**methyl 3-((2S,3S)-2-hydroxy-3-(2-oxooxazolidin-3-yl)pent-4-en-2-yl)benzoate** (**12l**): According to the Method A general procedure, a crude mixture of 73:27 **12l**:**13l** was obtained and purified by silica gel chromatography (eluent: 0 – 20% EtOAc in $CH_2Cl_2$) to provide 35.9 mg (47%) of **12l** as a thick glass as a single diastereomer as a 97:3 mixture of enantiomers. The stereochemistry was assigned by analogy to that of **12a**. $R_f$ = 0.38 (10% EtOAc/ $CH_2Cl_2$). $^1$HNMR ($CDCl_3$, 600 MHz) δ: 8.11 (s, 1H), 7.93 ( d, *J* = 8.6 Hz, 1H), 7.76 (d, *J* = 8.0 Hz, 1H), 7.44 (t, *J* = 8.60 Hz, 1H), 6.27 (ddd, *J* = 17.5 Hz, *J* = 9.5 Hz, *J* = 9.0 Hz, 1H), 5.45 (d, *J* = 10.0 Hz, 1H), 5.37 (d, *J* = 17.6 Hz, 1H), 4.63 (s, 1H), 4.15 (d, *J* = 9.0 Hz, 1H), 4.07 (q, *J* = 8.2, 1H), 4.01(q, *J* = 8.5 Hz, 1H), 3.92 (s, 3H), 3.58 (q, *J* = 8.5 Hz, 1H), 3.27 (q, *J* = 7.4 Hz, 1H), 1.52 (s, 3H) ppm. $^{13}$C NMR (151 MHz, $CDCl_3$): δ 167.1, 158.8, 146.5, 130.0, 129.9, 129.5, 128.4, 128.2, 125.5, 121.0, 76.8, 67.1, 62.7, 52.1, 45.3, 29.2 ppm. HRMS (DART) *m/z* calcd for $C_{16}H_{20}NO_5$ [M + H]$^+$: 306.1341; Found [M]$^+$: 306.1315.

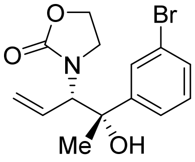

**12m**

**3-((3S,4S)-4-(3-bromophenyl)-4-hydroxypent-1-en-3-yl)oxazolidin-2-one** (**12m**): According to the Method A general procedure, a crude mixture of 74:26 **12m**:**13m** was obtained and purified by silica gel chromatography (eluent: 10 – 50% EtOAc in hexanes) to provide 67.0 mg (73%) of **12m** in 89 wt% purity by quantitative $^1$H NMR spectroscopy using dimethylfumarate as analytical standard as a white solid as a single diastereomer as a 92:8 mixture of enantiomers (allene rearrangement products *N*-allyl and *N*-propenyl oxazolidin-2-one could not be removed by chromatography). Analytically and diastereomerically pure material could be obtained by slurrying the material in 10 vol. of hot 30% EtOAc in hexanes followed by cooling to rt in 80% recovery with 98:2 er. The stereochemistry was assigned by analogy to that of **12a**. $R_f$ = 0.18 (30% EtOAc/hexanes). $^1$HNMR ($CDCl_3$, 600 MHz) δ: 7.64 (s, 1H), 7.40 (t, *J* = 8.7 Hz, 2H), 7.23 (t, *J* = 7.6 Hz, 1H), 6.26 (ddd, *J* = 16.91 Hz, *J* = 9.8 Hz, *J* = 8.6 Hz, 1H), 5.45 (d, *J* = 10.6 Hz, 1H), 5.37 (d, *J* = 17.6 Hz, 1H), 4.50 (br s, 1H), 4.05 – 4.15 (m, 3H), 3.57 (q, *J* = 7.6 Hz, 1H), 3.29 (q, *J* = 7.55 Hz, 1H) 1.50 (s, 3H) ppm. $^{13}$C NMR (151 MHz, $CDCl_3$): δ 148.5, 130.3, 130.1, 129.9, 129.8, 127.8, 123.5, 122.5, 121.2, 76.8, 67.1, 62.8, 45.3, 29.2 ppm. HRMS (DART) *m/z* calcd for $C_{14}H_{17}BrNO_3$ [M + H]$^+$: 326.0392; Found [M + H]$^+$: 326.0380.

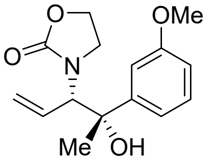

**12n**

**3-((3S,4S)-4-hydroxy-4-(3-methoxyphenyl)pent-1-en-3-yl)oxazolidin-2-one** (**12n**): According to the Method A general procedure, a crude mixture of 70:30 **12n**:**13n** was obtained and purified by silica gel chromatography (eluent: 0 – 20% EtOAc in $CH_2Cl_2$) to provide 37.4 mg (54%) of **12n** as a thick glass as a single diastereomer as a 96:4 mixture of enantiomers. The stereochemistry was assigned by analogy to that of **12a**. $R_f$ = 0.41 (10% EtOAc/ $CH_2Cl_2$). $^1$HNMR ($CDCl_3$, 600 MHz) δ: 7.27 (t, *J* = 8.5 Hz, 1H), 7.09 (s, 1H), 7.02 (d, *J* = 7.8 Hz, 1H), 6.80 (dd, *J* = 8.3 Hz, *J* = 2.1 Hz, 1H), 6.29 (ddd, *J* = 18.0 Hz, *J* = 9.8 Hz, *J* = 9.0 Hz, 1H), 5.43 (d, *J* = 10.6 Hz, 1H), 5.36 (d, *J* = 17.3 Hz, 1H), 4.43 (s, 1H), 4.17 (d, *J* = 8.4 Hz, 1H), 4.00 – 4.07 (m, 2H), 3.82 (s, 3H), 3.56 (q, *J* = 9.9 Hz, 1H), 3.28 (q, *J* = 8.5 Hz, 1H), 1.51 (s, 3H) ppm. $^{13}$C NMR (151 MHz, $CDCl_3$): δ 159.5, 158.8, 147.7, 130.7, 129.1, 120.6, 120.2, 116.9, 112.2, 111.3, 110.5, 76.9, 66.8, 62.7, 61.8, 55.2, 45.1, 44.1, 29.2 ppm. HRMS (DART) *m/z* calcd for $C_{15}H_{20}NO_4$ $[M + H]^+$: 278.1392; Found $[M + H]^+$: 278.1379.

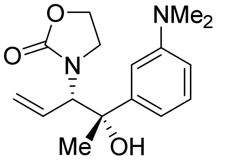

**12o**

**3-((3S,4S)-4-(3-(dimethylamino)phenyl)-4-hydroxypent-1-en-3-yl)oxazolidin-2-one** (**12o**): According to the Method A general procedure, a crude mixture of 72:28 **12o**:**13o** was obtained and purified by silica gel chromatography (eluent: 0 – 30% EtOAc in $CH_2Cl_2$) to provide 39.9 mg (55%) of **12o** as a thick yellow glass as a single diastereomer as a 92:8 mixture of enantiomers. The stereochemistry was assigned by analogy to that of **12a**. $R_f$ = 0.19 (10% EtOAc/ $CH_2Cl_2$). $^1$HNMR ($CDCl_3$, 600 MHz) δ: 7.19 (t, *J* = 8.2 Hz, 1H), 6.90 (br s, 1H), 6.73 (d, *J* = 9.4 Hz, 1H), 6.61 (d, *J* = 8.3 Hz, 1H), 6.29 (ddd, *J* = 17.8 Hz, *J* = 9.1 Hz, *J* = 9.3 Hz, 1H), 5.40 (d, *J* = 9.93 Hz, 1H), 5.34 (d, *J* = 16.6 Hz, 1H), 4.33 (s, 1H), 4.16 (d, *J* = 8.8 Hz, 1H), 3.98 – 4.04 (m, 2H), 3.50 (q, *J* = 7.9 Hz, 1H), 3.24 (q, *J* = 8.3 Hz, 1H), 2.94 (s, 6H), 1.50 (s, 3H) ppm. $^{13}$C NMR (151 MHz, $CDCl_3$): δ 158.8, 150.5, 146.6, 131.0, 130.8, 128.7, 120.3, 112.7, 111.0, 109.1, 66.8, 62.7, 45.1, 40.7, 29.3 ppm. HRMS (DART) *m/z* calcd for $C_{16}H_{23}N_2O_3$ $[M + H]^+$: 291.1709; Found $[M + H]^+$: 291.1714.

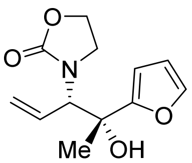

**12p**

**3-((3S,4R)-4-(furan-2-yl)-4-hydroxypent-1-en-3-yl)oxazolidin-2-one** (**12p**): According to the Method A general procedure, a crude mixture of >99:1 **12p**:**13p** was obtained and purified by silica gel chromatography (eluent: 10 – 50% EtOAc in hexanes) to provide 45.1 mg (76%) of **12p** in 90 wt% purity by quantitative $^1$H NMR spectroscopy using dimethylfumarate as analytical standard as a white solid as a single diastereomer as a 92:8 mixture of enantiomers (allene rearrangement products *N*-allyl and *N*-propenyl oxazolidin-2-one could not be removed by chromatography). Analytically and diastereomerically pure material could be obtained by slurrying the material in 10 vol. of hot 30% EtOAc in hexanes followed by cooling to rt in 73% recovery with >99 er. The stereochemistry was assigned by analogy to that of **12a**. $R_f$ = 0.10 (30% EtOAc/hexanes) $^1$HNMR ($CDCl_3$, 600 MHz) δ: 7.35 (s, 1H), 6.3 ( d, *J* = 2 Hz, 2H), 6.23 (ddd, *J* = 19.0, 17.2, 9.4, 1H), 5.42 (d, *J* = 10.2 Hz, 1H), 5.33 (d, *J* = 17.1 Hz, 1H), 4.63 (br, s, 1H), 4.21 (dd, *J* = 17.1 Hz, *J* = 8.9 Hz, 1H), 4.10 - 4.18 (m, 2H), 3.55 (dt, *J* = 8.4 Hz, *J* = 8.4, 1H), 3.34 (dt, *J* = 7.9 Hz, *J* = 8.9 Hz, 1H), 1.53 (s, 3H) ppm. $^{13}$C NMR (151 MHz, $CDCl_3$): δ 159.1, 158.3, 141.5, 130.1, 121.2, 110.6, 105.8, 74.5, 66.1, 62.9, 45.1, 25.9 ppm. HRMS (DART) *m/z* calcd for $C_{12}H_{16}NO_4$ $[M + H]^+$: 238.1079; Found $[M + H]^+$: 238.1079.

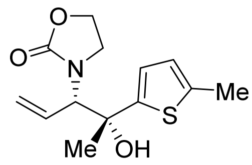
12q

**3-((3S,4R)-4-hydroxy-4-(5-methylthiophen-2-yl)pent-1-en-3-yl)oxazolidin-2-one** (**12q**): According to the Method A general procedure, a crude mixture of >99:1 **12q**:**13q** was obtained and purified by silica gel chromatography (eluent: 10 – 50% EtOAc in hexanes) to provide 59.5 mg (89%) of **12q** in 91 wt% purity by quantitative $^{1}$H NMR spectroscopy using dimethylfumarate as analytical standard as a yellow solid as a single diastereomer as a 90:10 mixture of enantiomers (allene rearrangement products *N*-allyl and *N*-propenyl oxazolidin-2-one could not be removed by chromatography). Analytically and diastereomerically pure material could be obtained by slurrying the material in 10 vol. of hot 30% EtOAc in hexanes followed by cooling to rt in 70 recovery with 75:25 er. The stereochemistry was assigned by analogy to that of **12a**. $R_f$ = 0.20 (30% EtOAc/hexanes). $^{1}$HNMR ($CDCl_3$, 600 MHz) δ: 6.76 (d, *J* = 3.3 Hz, 1H), 6.60 (d, *J* = 3.3 Hz, 1H), 6.22 (ddd, *J* = 17.3 Hz, *J* = 10.0 Hz, *J* = 8.6 Hz, 1H), 5.41 (d, *J* = 10.3 Hz, 1H), 5.35 (d, *J* = 17.1 Hz, 1H), 4.40 (br s, 1H), 4.14 – 4.22 (m, *2*H), 4.06 (d, *J* = 8.8 Hz, 1H), 3.65 (dt, *J* = 6.9 Hz, *J* = 8.8 Hz, 1H), 3.41 (dt, *J* = 8.2 Hz, *J* = 8.6 Hz, 1H), 2.44 (s, *3*H), 1.57 (s, *3*H) ppm. $^{13}$C NMR (151 MHz, $CDCl_3$): δ 158.9, 148.0, 138.4, 130.6, 127.9, 124.9, 122.7, 121.1, 76.1, 67.5, 62.8, 44.9, 29.7, 15.2 ppm. HRMS (DART) *m/z* calcd for $C_{13}H_{18}NO_3S$ $[M + H]^+$: 268.1007; Found $[M + H]^+$: 268.0996.

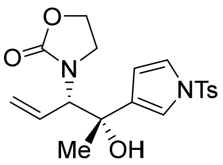
12r

**3-((3S,4R)-4-hydroxy-4-(1-tosyl-1H-pyrrol-2-yl)pent-1-en-3-yl)oxazolidin-2-one** (**12r**): According to the Method A general procedure, a crude mixture of >99:1 **12r**:**13r** was obtained and purified by silica gel chromatography (eluent: 20 – 70% EtOAc in hexanes) to provide 78.1 mg (80%) of **12r** as a thick glass as a single diastereomer as a 76:24 mixture of enantiomers. The stereochemistry was assigned by analogy to that of **12a**. $R_f$ = 0.07 (30% EtOAc/hexanes). $^{1}$HNMR ($CDCl_3$, 600 MHz) δ: 7.70 (d, *J* = 8.6 Hz, 2H), 7.9 (d, *J* = 8.6 Hz, 2H), 7.13 (t, *J* = 2.0 Hz, 1H), 7.10 – 7.12 (m, 1H), 6.22 – 6.25 (m, 1H), 6.15 (ddd, *J* = 17.2 Hz, *J* = 10.3 Hz, *J* = 8.8 Hz, 1H), 5.39 (dd, *J* = 10.1 Hz, *J* = 0.7, 1H), 5.30 (dd, *J* = 17.0 Hz, *J* = 0.8 Hz, 1H), 3.97 (dd, *J* = 9.2 Hz, *J* = 1.1 Hz, 1H), 3.92 – 3.96 (m, 1H), 3.87 (br s, 1H), 3.49 (dt, *J* = 6.6 Hz, *J* = 8.9 Hz, 1H), 3.26 (t, *J* = 7.7 Hz, *J* = 8.4 Hz, 1H), 2.40 (s, 3H), 1.44 (s, 3H) ppm. $^{13}$C NMR (151 MHz, $CDCl_3$): δ 158.7, 144.8, 135.9, 135.3, 130.3, 129.9, 126.6, 121.3, 120.9, 116.9, 111.6, 74.2, 66.1, 62.4, 44.3, 28.3, 21.5 ppm. HRMS (DART) *m/z* calcd for $C_{19}H_{23}N_2O_5S$ $[M + H]^+$: 391.1328; Found $[M + H]^+$: 391.1352.

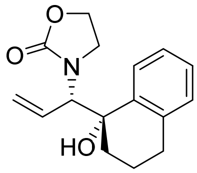
12s

**3-((S)-1-((S)-1-hydroxy-1,2,3,4-tetrahydronaphthalen-1-yl)allyl)oxazolidin-2-one** (**12s**): According to the Method A general procedure, a crude mixture of >99:1 **12s**:**13s** and 1:2 *b:l* was obtained and purified by silica gel chromatography (eluent: 10 – 50% EtOAc in hexanes) to provide 29.1 mg (28%) of **12s** in 88 wt% purity by quantitative $^{1}$H NMR spectroscopy using dimethylfumarate as analytical standard as a white solid as a single diastereomer as a 72:28 mixture of enantiomers (allene rearrangement products *N*-allyl and *N*-propenyl oxazolidin-2-one could not be removed by chromatography). Analytically and diastereomerically pure material could be obtained by slurrying the material in 10 vol. of hot 30% EtOAc in hexanes followed by cooling to rt in 66% recovery with 70:30 er. The stereochemistry was assigned by analogy to that of **12a**. $R_f$ = 0.11 (30% EtOAc/hexanes). $^{1}$HNMR ($CDCl_3$, 600 MHz) δ: 7.48 – 7.52 (m, 1H), 7.15 – 7.23 (m, 2H), 7.07 – 7.10 (m, 1H), 6.17 (ddd, *J* = 17.6 Hz, *J* = 10.5 Hz, *J* = 8.8 Hz, 1H), 5.35 (d, *J* = 10.0 Hz, 1H), 5.28 (d, *J* = 17.6 Hz, 1H), 4.34 (d, *J* = 8.5 Hz, 1H), 4.28 (dt, *J* = 8.8 Hz, *J* = 8.2 Hz, 1H), 4.22 (dt, *J* = 6.2 Hz, 9.1 Hz, 1H), 3.75 (dt, *J* = 6.4 Hz, *J* = 8.8 Hz, 1H), 3.40 (br, s, 1H), 3.30 (q, *J* = 8.8 Hz, 1H), 2.84 (t, *J* = 7.0 Hz, 2H), 2.14 (dt, *J* = 13.2 Hz, *J* = 5.3 Hz, 1H), 1.85 – 1.92 (m, 2H), 1.77 – 1.83 (m, 1H), 1.55 (s, 3H) ppm. $CDCl_3$: δ 159.3, 140.0, 136.6, 131.1, 129.0,

127.6, 126.2, 125.8, 120.9, 75.4, 64.6, 62.7, 44.6, 44.6, 34.5, 28.6, 19.0. HRMS (DART) *m/z* calcd for $C_{16}H_{20}NO_3$ $[M + H]^+$: 274.1444; Found $[M + H]^+$: 274.1444.

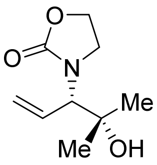

**12t**

**(*S*)-3-(4-hydroxy-4-methylpent-1-en-3-yl)oxaxolidin-2-one** (**12t**): According to the general procedure, the product was purified by silica gel chromatography (eluent: 0 – 40% EtOAc in $CH_2Cl_2$) to provide 23.6 mg (51%) of **12t** as a white solid as a single diasteromer. The stereochemistry was assigned by analogy to that of **12a**. $R_f$ = 0.24 (40% EtOAc/$CH_2Cl_2$). $^1$HNMR ($CDCl_3$, 600 MHz) δ: 6.07 (ddd, *J* = 17 Hz, *J* = 10Hz, *J* = 8.8 Hz, 1H), 5.38 (d, *J* = 10 Hz, 1H), 5.32 (d, *J* = 17 Hz, 1H), 4.35 (t, *J* = 8.5 Hz, 2H), 3.94 (d, *J* = 8.8 Hz, 1H), 3.82 (dd, *J* = 16 Hz, *J* = 8.5 Hz, 1H), 3.62 (dd, *J* = 16 Hz, *J* = 8.5 Hz, 1H), 2.51 (s, 1H), 1.31 (s, 3H), 1.24 (s, 3H) ppm. $^{13}$C NMR (151 MHz, $CDCl_3$): δ 159.1, 131.1, 121.0, 72.9, 65.6, 62.6, 43.7, 27.8, 27.5. HRMS (DART) *m/z* calcd for $C_9H_{16}NO_3$ $[M + H]^+$: 186.1130; Found $[M + H]^+$: 186.1131.

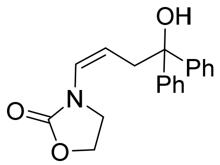

*l*-**12u**

**(*Z*)-3-(4-hydroxy-4,4-diphenylbut-1-en-1-yl)oxaxolidin-2-one** (*l*-**12u**): According to the general procedure, the product was purified by silica gel chromatography (eluent: 0 – 10% EtOAc in $CH_2Cl_2$) to provide 24.2 mg (33%) of *l*-**12u** as a white solid. $R_f$ = 0.27 (10% EtOAc/$CH_2Cl_2$). $^1$HNMR ($CDCl_3$, 600 MHz) δ: 7.35 (d, *J* = 8.0 Hz, 4H), 7.21 (t, *J* = 8.0 Hz, 4H), 7.12 (t, *J* = 8.0 Hz, 2H), 6.11 (d, *J* = 9.4 Hz, 1H), 4.77 (q, *J* = 9.1 Hz, 1H), 4.26 (t, *J* = 8.3 Hz, 2H), 3.79 (t, *J* = 7.8 Hz, 2H), 3.26 (s, 1H), 3.07 (d, *J* = 7.8 Hz, 2H) ppm. $^{13}$C NMR (151 MHz, $CDCl_3$): δ 156.8, 146.4, 128.2, 126.9, 125.8, 125.8, 112.1, 77.1, 62.2, 45.9, 39.2. HRMS (DART) *m/z* calcd for $C_{19}H_{20}NO_3$ $[M + H]^+$: 310.1443; Found $[M + H]^+$: 310.1435.

**Reaction Performed on 1.0 mmol scale:**

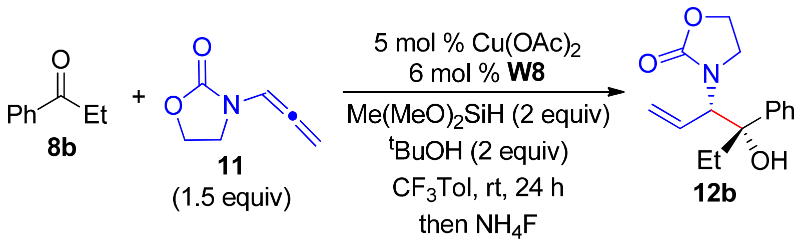


To a 20 mL crimp-cap vial with stir-bar in an Ar-filled glove-box was charged 9.2 mg (0.05 mmol) of $Cu(OAc)_2$ and 56.0 mg (0.06 mmol) of Walphos-8. α,α,α-Trifluorotoluene (2.0 mL) was then added, and the mixture was allowed to stir for 10 min. Allenamide **11** (188.0 mg, 1.5 mmol) followed by ketone **8b** (1.0 mmol) and $^t$BuOH ( 0.192 mL, 2.00 mmol) was then charged. The vial was then sealed with a crimp-cap septum and removed from the glove-box. The reaction was then cooled in an ice bath, and dimethoxymethylsilane (0.248 µL, 2.0 mmol) was then charged by syringe *(caution: dimethoxymethylsilane should be handled in a well-ventilated fume hood because it is known to cause blindness. Syringes were quenched with 2M NaOH, gas evolution!, prior to disposal)* The mixture was then allowed to warm to rt and stirred for 24 h. The reaction was then quenched by the addition of 380 mg of NH4F and 6.0 mL of MeOH followed by agitation at rt for 30 min – 1 h. The crude mixture was then transferred to a separatory funnel to which 20 mL of 5% NaHCO3 was then charged and agitated. The mixture was then extracted with $CH_2Cl_2$ (2x10mL). The combined organics were dried with $Na_2SO_4$ and concentrated *in vacuo*. An aliquot of the crude mixture was analyzed by $^1$HNMR spectroscopy to determine the dr (>99) and b/l (>99) ratio. The crude residue was then purified by flash chromatography on silica gel (gradient, 0 – 20% EtOAc in $CH_2Cl_2$). The first spot to elute was isolated as a (**W8**)Cu complex that was then de-complexed for recovery of **W8** (see below). The product spot ($R_f$ = 0.31, 10% EtOAc in $CH_2Cl_2$) was then collected and concentrated *in vacuo* to afford 188 mg (72%) of **12b**. Enantioselectivity was determined by chiral HPLC analysis to be 96:4.

**Recovery of W8:** The (**W8**)Cu complex obtained from the above reaction was further purified by flash chromatography on silica gel (gradient, 0 – 15% EtOAc in hexanes) to afford 71.0 mg of an orange solid. This material was then dissolved in 3.0 mL of 2:1 pentane:MTBE and then 1 mL of 50% $NH_4OH$ solution was added. The mixture was then agitated vigorously for 5 minutes upon which the lower blue aqueous layer was removed. The organic layer was then washed twice with 1 mL of 50% $NH_4OH$, dried with $Na_2SO_4$ and concentrated *in vacuo* to yield 45.1 mg (81% recovery) of **W8** as an orange solid. Use of this recovered ligand in the Cu-catalyzed reductive coupling with propiophenone provided identical results to that obtained with the commercially obtained **W8**.

**Large Scale recrystallization of 12a:**

To a 20 mL crimp-cap vial with a stir-bar was charged 357.0 mg of **12a** of 91 wt% with 90/10 er. To the vial was then added 2.0 mL of 30% EtOAc/Hexanes solution and heated to 40 °C while stirring vigorously. After 30 min of stirring, the mixture was allowed to cool to rt and stir for and additional 30 min and then filtered to yield 237 mg (73%) of analytically pure **12a** with >99:1 er.

**Oxazolidinone Removal:**

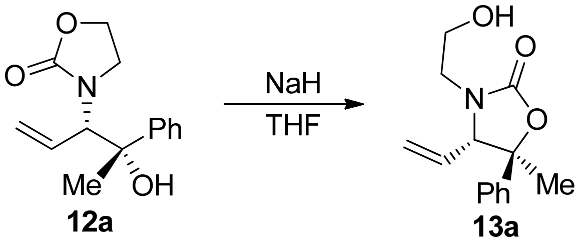


To a solution of 210 mg (0.850 mmol) of **12a** in 2.0 mL of THF at 0 °C was charged 34 mg (0.850 mmol) of NaH. The reaction was then allowed to warm to room temperature and stirred for 1 h. The reaction was then quenched by the addition of 5 mL of 1M HCl and extracted with $CH_2Cl_2$ (3 X 10 mL). The combined organics were dried with $Na_2SO_4$ and concentrated *in vacuo*. The crude residue was purified by flash chromatography (silica gel, gradient, 20 – 50% EtOAc/hexanes) to afford 209.1 mg (99%) of **13a** as a thick waxy oil. $R_f$ = 0.18 (10% EtOAc/$CH_2Cl_2$). $^1$HNMR (CDCl3, 600 MHz) δ: 7.35 – 7.41 (m, 4H), 7.32 (t, $J$ = 6.7 Hz, 1H), 5.91 (dt, $J$ = 16.6 Hz, $J$ = 10.1 Hz, 1H), 5.53 (d, $J$ = 10.2 Hz, 1H), 5.43 (d, $J$ = 16.2 Hz, 1H), 5.22 (d, $J$ = 9.5 Hz, 1H), 3.67 – 3.75 (m, 2H), 3.45 (ddd, $J$ = 14.8 Hz, $J$ = 6.3 Hz, $J$ = 4.2 Hz, 1H), 3.19 (ddd, $J$ = 14.6 Hz, $J$ = 6.9 Hz, $J$ = 3.7 Hz, 1H), 1.63 (s, 3H) ppm. $^{13}$C NMR (151 MHz, $CDCl_3$): δ 158.0, 144.1, 132.3, 128.7, 127.9, 123.8, 123.1, 83.4, 70.8, 60.8, 45.0, 23.9 ppm. HRMS (DART) *m/z* calcd for $C_{14}H_{18}NO_3$ $[M + H]^+$: 248.1287; Found $[M + H]^+$: 248.1287.

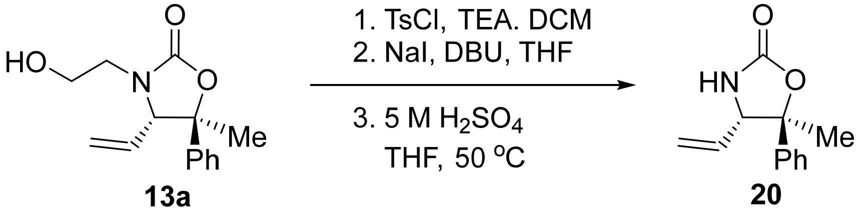


To a solution of 209.1 mg (0.846 mmol) of **13a** in 4.2 mL of $CH_2Cl_2$ at 0 °C was charged 141 µL (1.02 mmol) of triethylamine followed by 177 mg (0.931 mmol) of TsCl. The mixture was stirred for 30 min at 0 °C, allowed to warm to rt and stirred for 8 h. To the mixture was charged 2 mL of 10% $NH_4Cl$ followed by extraction with $CH_2Cl_2$ (3x2mL). The combined organics were dried with anhydrous $Na_2SO_4$, and volatile material was removed *in vacuo*. The crude residue was then dissolved in 8.0 mL of glyme and charged with 360 mg (2.54 mmol) of NaI and 383 µL (2.54 mmol) of DBU and refluxed for 8 h. The mixture was diluted with 30 mL of 1:1 mixture of $Et_2O$ and $H_2O$ and stirred for 10 min upon which organics were extracted with $Et_2O$ (2x10 mL). The combined organic layers were washed with brine, dried with anhydrous $Na_2SO_4$, filtered, and volatiles removed *in vacuo*. The crude residue was then dissolved in 5.0 mL of THF in a 20 mL scintillation vial. To the solution was added 1.7 mL (8.46 mmol) of 5.0 M aqueous $H_2SO_4$. The vial was purged with argon, sealed, and immersed in an oil bath at 50 °C. After 2.5 h, the reaction was cooled to rt and 10 mL of saturated aqueous $NaHCO_3$ was charged. The mixture was extracted with $CH_2Cl_2$ (3x5mL), dried with anhydrous $Na_2SO_4$, and concentrated *in vacuo*. The crude residue was purified by flash chromatography (silica gel, gradient, hexanes to 60% EtOAc/hexanes) to afford 134.2 mg (78%) of **20** as a White solid. $R_f$ = 0.19 (40% EtOAc/hexanes). Spectral data was identical to that made previously.[2]

**Absolute and relative stereochemistry determination:**

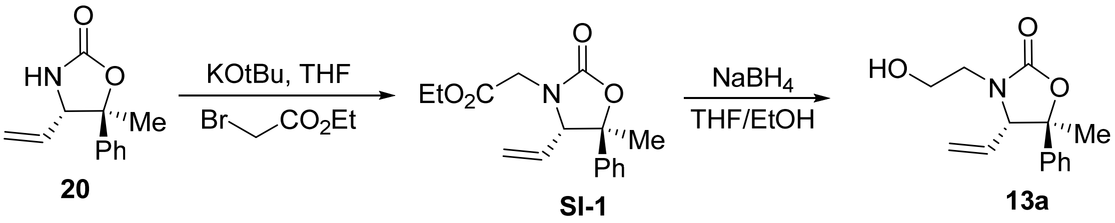

Diastereo- and enantiopure **20** prepared by our previous work[2] was converted to **13a** by the given two-step procedure[3] and then compared to **13a** prepared using the current Cu(OAc)2/**W8** catalysts system followed by carbonate rearrangement. The major diastereomer and enantiomer formed from the Cu(OAc)2/**W8** catalysts system was the same as that of authentic **13a** prepared by the given procedure by comparison of the authentic material by $^{1}$HNMR spectropscopy and chiral HPLC analysis.

**Alkylation:** To a solution of **20** (20.0 mg, 0.0984 mmol) in 0.25 mL of THF at 0 °C was charged 0.11 mL of a 1.0 M (0.11 mmol) solution of KO$^t$Bu in THF. The mixture was then allowed to stir for 10 min before the addition of 12 µL (11 mg, 0.11 mmol) of ethyl bromoacetate. The mixture was warmed to rt and allowed to stir for 3 h. To the reaction was added 2 mL of 10% aqueous $NH_4Cl$, and the mixture was extracted with $CH_2Cl_2$ (3x2mL). The combined organics were dried with anhydrous $Na_2SO_4$, filtered, and concentrated *in vacuo* to afford **SI-1**. This material was used directly in the next step without further purification.

**Reduction:** To a solution of **SI-1** in 0.25 mL of 7:1 THF:EtOH was charged 11.2 mg (0.295 mmol) of $NaBH_4$, and the resultant mixture was allowed to stir at rt for 3 h. The reaction was then cooled to 0 °C, and 2 mL of 10% aqueous $NH_4Cl$ was carefully added (gas evolution!). The mixture was then warmed to rt and extracted with EtOAc (3x2mL). The combined organics were dried with anhydrous $Na_2SO_4$, filtered, and concentrated *in vacuo* to afford 17.1 mg (70%, 2 steps) of **13a** as a colorless oil. $^{1}$HNMR spectroscopy matched that of the material prepared using the current Cu(OAc)2/**W-8** catalysts system followed by carbonate rearrangement (Scheme 3). Chiral HPLC analysis of this material relative to the material prepared from the Cu(OAc)2/**W8** catalysts system followed by carbonate rearrangement is given below:

*Chiral HPLC analysis (*Chiralpak *AD-3 x 250 mm, heptane/ethanol = 85/15, flow rate = 1.2 mL/min, λ = 254 nm) $t_R$ = 5.2 min (major), 5.7 min (minor):*

**Racemic 13a:**

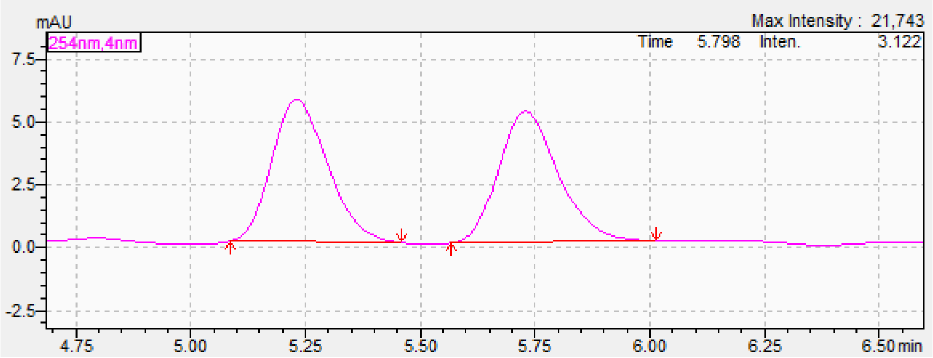


| Peak | Ret. Time | Area% | Height | Mark | Conc. |
|---|---|---|---|---|---|
| 1 | 5.232 | 50.094 | 72081 | M | 0.000 |
| 2 | 5.729 | 49.906 | 67571 | M | 0.000 |
| Total | | 100.000 | 139652 | | 0.000 |

**Authentic 13a:**

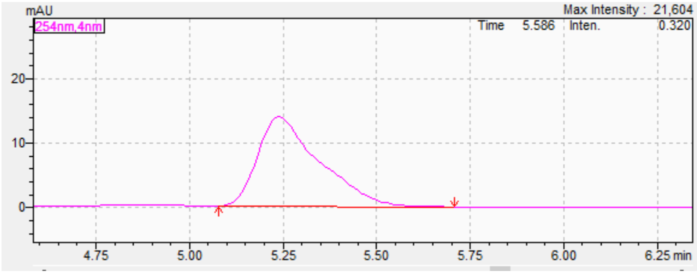


**Authentic 13a + racemic spike:**

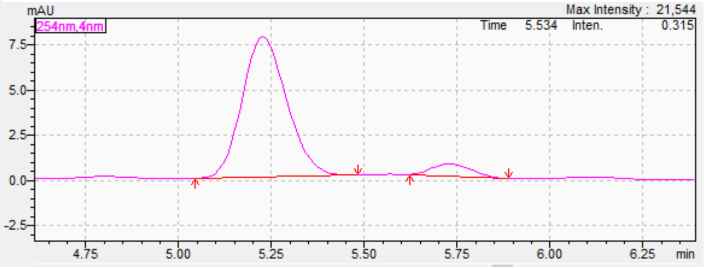


**13a from $Cu(OAc)_2$/W8 reaction followed by carbonate rearrangement using NaH:**

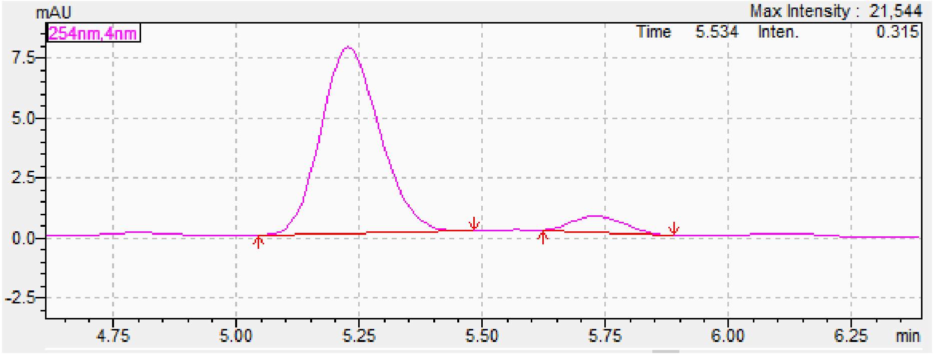


| Peak | Ret. Time | Area% | Height | Mark | Conc. |
|---|---|---|---|---|---|
| 1 | 5.226 | 92.794 | 7797 | M | 0.000 |
| 2 | 5.726 | 7.206 | 685 | M | 0.000 |
| Total | | 100.000 | 8482 | | 0.000 |

**Chiral HPLC analysis of the reductive coupling products:**

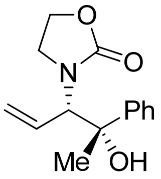


*Chiral HPLC analysis (Chiralcel OD-3 x 250 mm, heptane/isopropanol = 90/10, flow rate = 1.0 mL/min, λ = 190 nm) $t_R$ = 18.9 min (minor), 23.6 min (major):*

**Racemic 12a:**

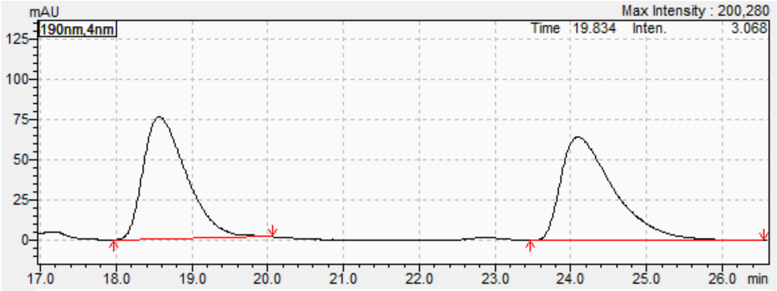


| Peak | Ret. Time | Area% | Height | Mark | Conc. |
|---|---|---|---|---|---|
| 1 | 18.570 | 48.921 | 75785 | M | 48.921 |
| 2 | 24.093 | 51.079 | 64405 | M | 51.079 |
| Total | | 100.000 | 140191 | | 100.000 |

**12a from the $Cu(OAc)_2$/W8 reaction:**

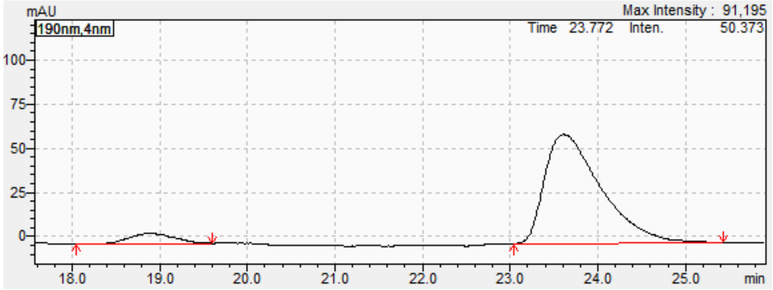


| Peak | Ret. Time | Area% | Height | Mark | Conc. |
|---|---|---|---|---|---|
| 1 | 18.852 | 6.965 | 5744 | M | 6.965 |
| 2 | 23.611 | 93.035 | 61653 | M | 93.035 |
| Total | | 100.000 | 67397 | | 100.000 |

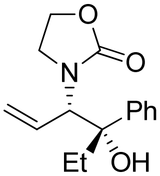

*Chiral HPLC analysis (Chiralpak AD-3 x 250 mm, heptane/isopropanol = 90/10, flow rate = 1.0 mL/min, λ = 213 nm) $t_R$ = 12.1 min (major), 13.9 min (minor):*

**Racemic 12b:**

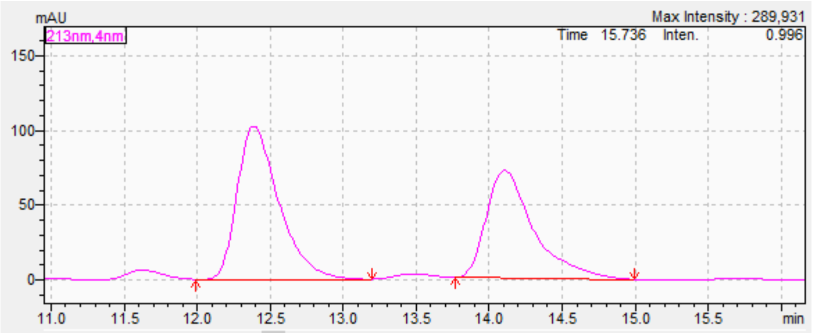


| Peak | Ret. Time | Area% | Height | Mark | Conc. |
|---|---|---|---|---|---|
| 1 | 12.388 | 55.520 | 79465 | M | 0.000 |
| 2 | 14.103 | 44.480 | 56263 | M | 0.000 |
| Total | | 100.000 | 135727 | | 0.000 |

**12b from the $Cu(OAc)_2$ /W8 reaction:**

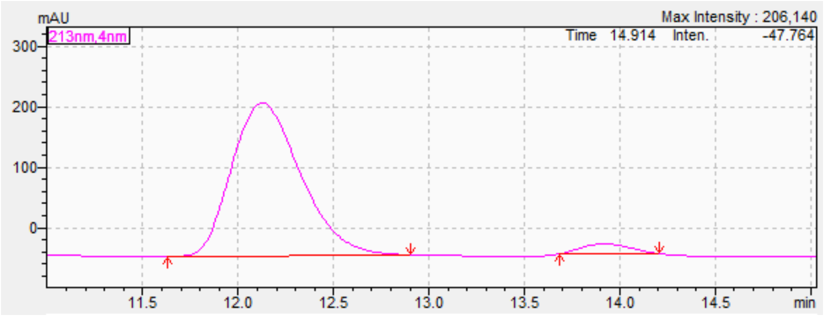


| Peak | Ret. Time | Area% | Height | Mark | Conc. |
|---|---|---|---|---|---|
| 1 | 12.126 | 95.743 | 253938 | M | 0.000 |
| 2 | 13.915 | 4.257 | 16458 | M | 0.000 |
| Total | | 100.000 | 270396 | | 0.000 |

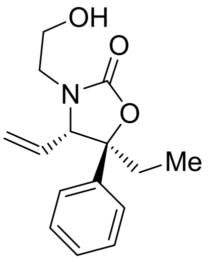


*Chiral HPLC analysis (Chiralcel OD-3 x 250 mm, heptane/isopropanol = 90/10, flow rate = 1.0 mL/min, λ = 205 nm) $t_R$ = 7.3 min (minor), 8.0 min (major):*

**Racemic 13b:**

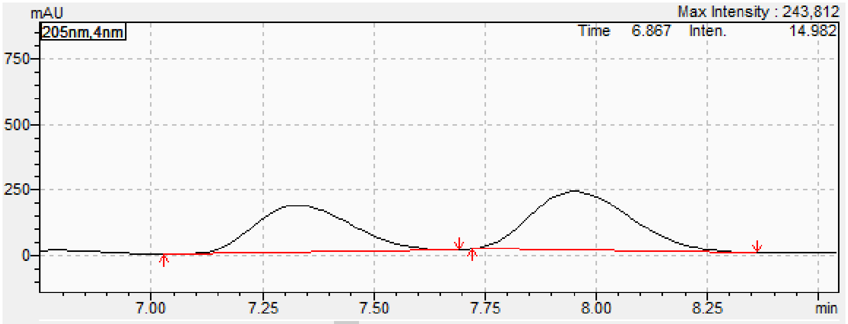


| Peak | Ret. Time | Area% | Height | Mark | Conc. |
|---|---|---|---|---|---|
| 1 | 7.330 | 47.091 | 180354 | M | 47.091 |
| 2 | 7.952 | 52.909 | 210977 | M | 52.909 |
| Total | | 100.000 | 391331 | | 100.000 |

**13b from the Cu(OAc)$_2$/W8 reaction:**

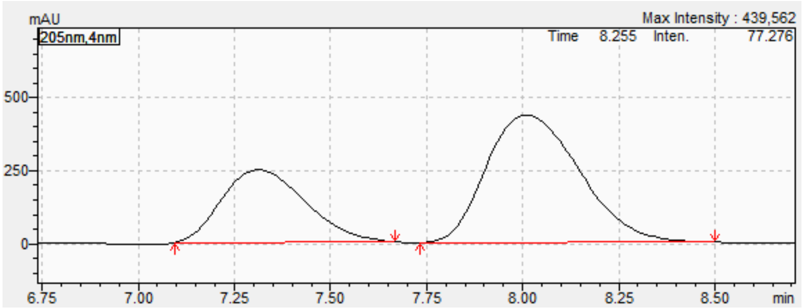


| Peak | Ret. Time | Area% | Height | Mark | Conc. |
|---|---|---|---|---|---|
| 1 | 7.312 | 32.865 | 134980 | M | 32.865 |
| 2 | 8.010 | 67.135 | 232609 | M | 67.135 |
| Total | | 100.000 | 367590 | | 100.000 |

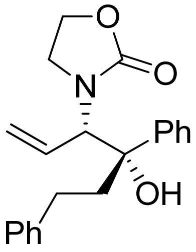

*Chiral HPLC analysis (Chiralpak AD-3 x 250 mm, heptane/isopropanol = 90/10, flow rate = 1.0 mL/min, λ = 206 nm) $t_R$ = 11.8 min (minor), 12.8 min (major):*

**Racemic 12c:**

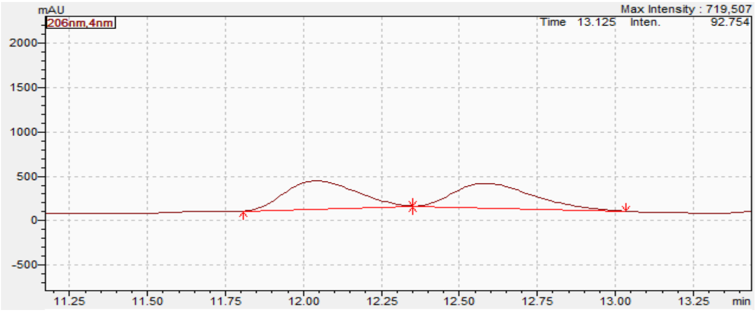


| Peak | Ret. Time | Area% | Height | Mark | Conc. |
|---|---|---|---|---|---|
| 1 | 12.042 | 49.914 | 101377 | M | 0.000 |
| 2 | 12.582 | 50.086 | 90034 | M | 0.000 |
| Total | | 100.000 | 191411 | | 0.000 |

**12c from the Cu(OAc)$_2$/W8 reaction:**

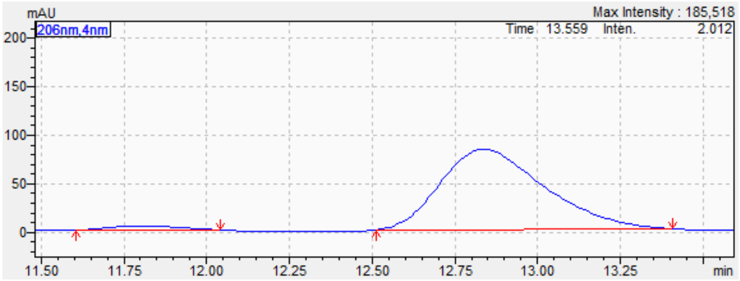


| Peak | Ret. Time | Area% | Height | Mark | Conc. |
|---|---|---|---|---|---|
| 1 | 11.798 | 3.306 | 3431 | M | 0.000 |
| 2 | 12.836 | 96.694 | 62430 | M | 0.000 |
| Total | | 100.000 | 65861 | | 0.000 |

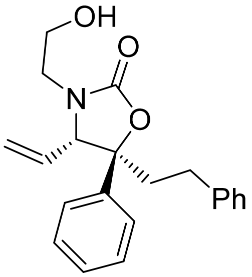

*Chiral HPLC analysis (Chiralpak AD-3 x 250 mm, heptane/isopropanol = 90/10, flow rate = 1.0 mL/min, λ = 206 nm) $t_R$ = 12.1 min (minor), 13.1 min (major):*

**Racemic 13c:**

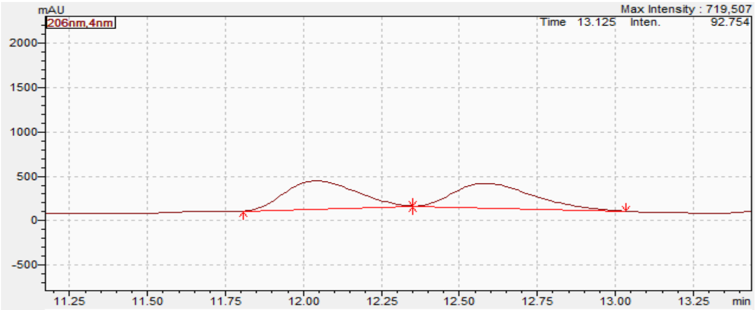


| Peak | Ret. Time | Area% | Height | Mark | Conc. |
|---|---|---|---|---|---|
| 1 | 12.042 | 49.914 | 101377 | M | 0.000 |
| 2 | 12.582 | 50.086 | 90034 | M | 0.000 |
| Total | | 100.000 | 191411 | | 0.000 |

**13c from the Cu(OAc)$_2$/W8 reaction:**

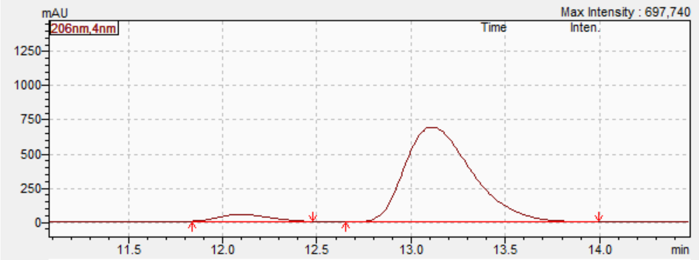


| Peak | Ret. Time | Area% | Height | Mark | Conc. |
|---|---|---|---|---|---|
| 1 | 12.096 | 5.145 | 51976 | M | 0.000 |
| 2 | 13.111 | 94.855 | 694801 | M | 0.000 |
| Total | | 100.000 | 746777 | | 0.000 |

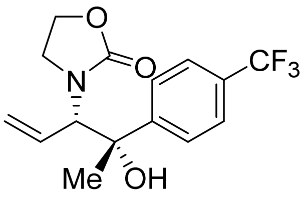


*Chiral HPLC analysis (Chiralpak AD-3 x 250 mm, heptane/isopropanol = 90/10, flow rate = 1.0 mL/min, λ = 190 nm) $t_R$ = 8.7 min (major), 10.3 min (minor):*

**Racemic 12d:**

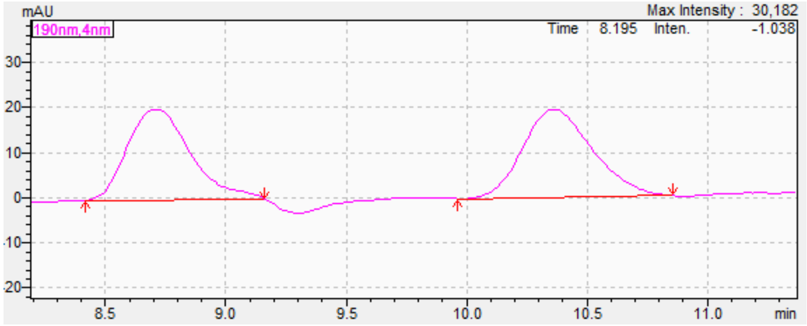


| Peak | Ret. Time | Area% | Height | Mark | Conc. |
|---|---|---|---|---|---|
| 1 | 8.707 | 48.368 | 20117 | M | 48.368 |
| 2 | 10.364 | 51.632 | 19433 | M | 51.632 |
| Total | | 100.000 | 39550 | | 100.000 |

**12d from the Cu(OAc)$_2$/W8 reaction:**

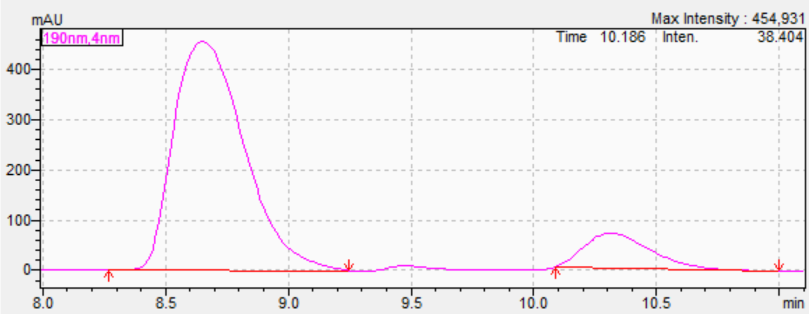


| Peak | Ret. Time | Area% | Height | Mark | Conc. |
|---|---|---|---|---|---|
| 1 | 8.651 | 87.737 | 454671 | M | 0.000 |
| 2 | 10.315 | 12.263 | 68870 | M | 0.000 |
| Total | | 100.000 | 523541 | | 0.000 |

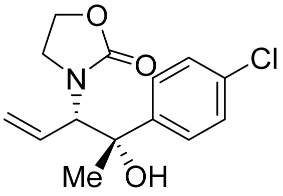


*Chiral HPLC analysis (Chiralcel OD-3 x 250 mm, heptane/isopropanol = 90/10, flow rate = 1.0 mL/min, λ = 209 nm) $t_R$ = 11.3 min (major), 12.6 min (minor):*

**Racemic 12e:**

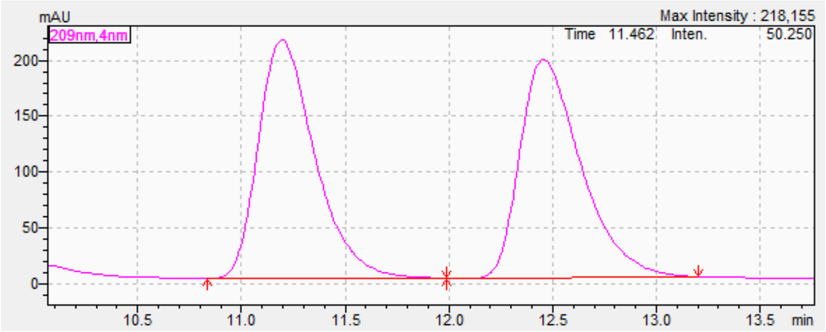


| Peak | Ret. Time | Area% | Height | Mark | Conc. |
|---|---|---|---|---|---|
| 1 | 11.195 | 50.396 | 213816 | M | 0.000 |
| 2 | 12.457 | 49.604 | 195616 | M | 0.000 |
| Total | | 100.000 | 409433 | | 0.000 |

**12e from the Cu(OAc)$_2$/W8 reaction:**

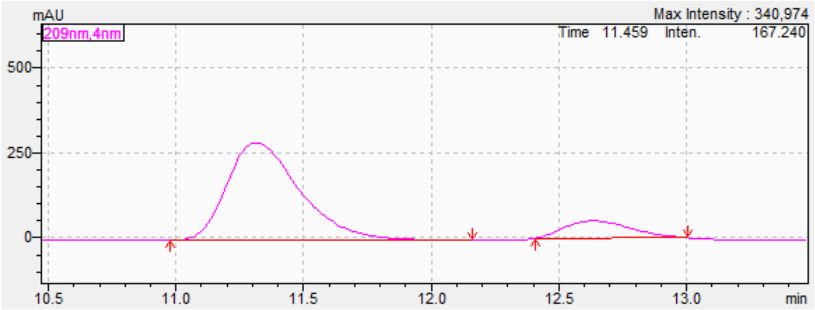


| Peak | Ret. Time | Area% | Height | Mark | Conc. |
|---|---|---|---|---|---|
| 1 | 11.314 | 86.267 | 288084 | M | 86.267 |
| 2 | 12.633 | 13.733 | 51138 | M | 13.733 |
| Total | | 100.000 | 339222 | | 100.000 |

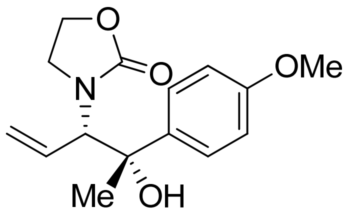


*Chiral HPLC analysis (Chiralpak AD-3 x 250 mm, heptane/isopropanol = 90/10, flow rate = 1.0 mL/min, λ = 190 nm)* $t_R$ = *11.1 min (major), 11.7 min (minor):*

**Racemic 12f:**

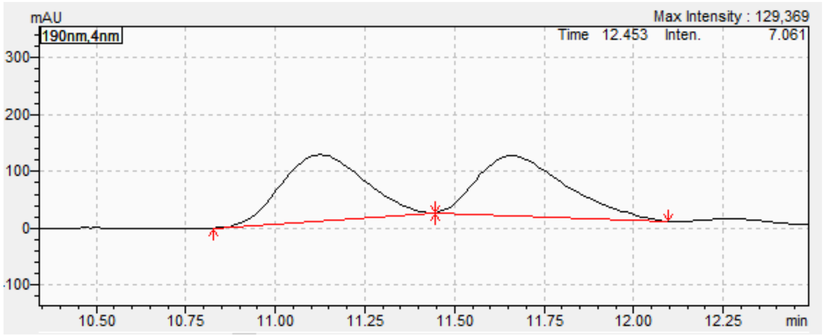


| Peak | Ret. Time | Area% | Height | Mark | Conc. |
|---|---|---|---|---|---|
| 1 | 11.123 | 50.752 | 115905 | M | 50.752 |
| 2 | 11.659 | 49.248 | 104736 | M | 49.248 |
| Total | | 100.000 | 220641 | | 100.000 |

**12f from the Cu(OAc)** $_2$**/W8 reaction:**

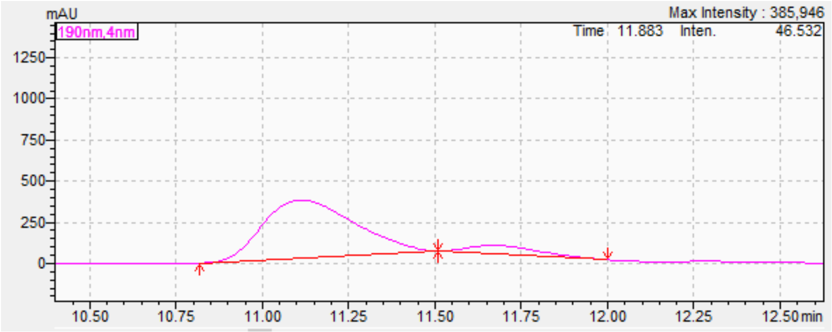


| Peak | Ret. Time | Area% | Height | Mark | Conc. |
|---|---|---|---|---|---|
| 1 | 11.111 | 89.668 | 353191 | M | 0.000 |
| 2 | 11.669 | 10.332 | 53741 | M | 0.000 |
| Total | | 100.000 | 406932 | | 0.000 |

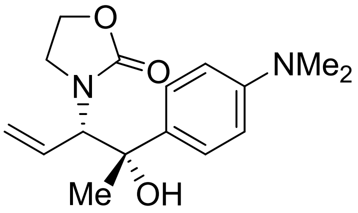

*Chiral HPLC analysis (Chiralcel OD-3 x 250 mm, heptane/isopropanol = 90/10, flow rate = 1.0 mL/min, λ = 254 nm) $t_R$ = 9.0 min (minor), 9.6 min (major):*

**Racemic 12g:**

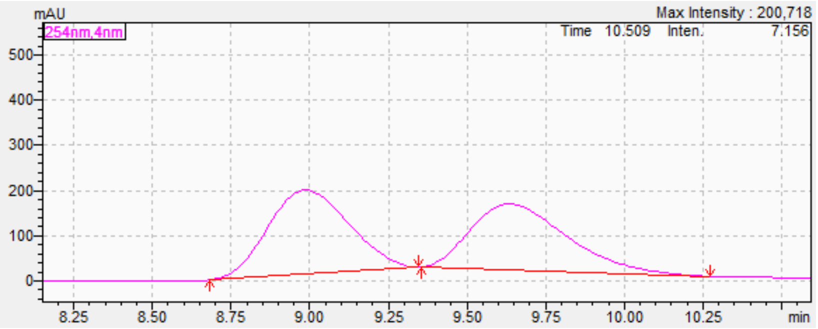


| Peak | Ret. Time | Area% | Height | Mark | Conc. |
|---|---|---|---|---|---|
| 1 | 8.986 | 51.740 | 185880 | M | 51.740 |
| 2 | 9.634 | 48.260 | 145818 | M | 48.260 |
| Total | | 100.000 | 331699 | | 100.000 |

**12g from the Cu(OAc)$_2$/W8 reaction:**

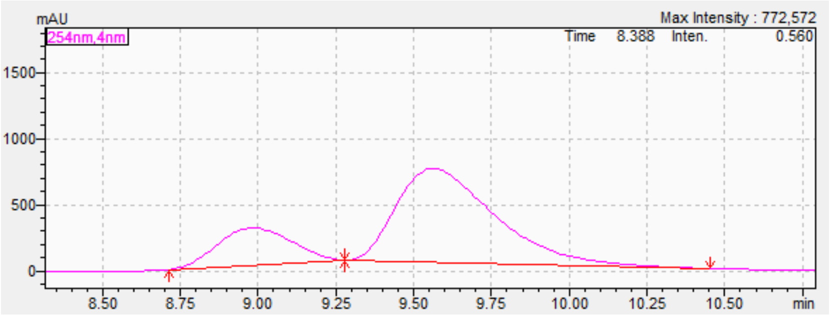


| Peak | Ret. Time | Area% | Height | Mark | Conc. |
|---|---|---|---|---|---|
| 1 | 8.982 | 23.067 | 282239 | M | 23.067 |
| 2 | 9.562 | 76.933 | 708798 | M | 76.933 |
| Total | | 100.000 | 991036 | | 100.000 |

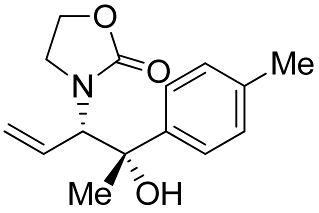


*Chiral HPLC analysis (Chiralcel OD-3 x 250 mm, heptane/isopropanol = 90/10, flow rate = 1.0 mL/min, λ = 258 nm) $t_R$ = 14.3 min (minor), 18.1 min (major):*

**Racemic 12h:**

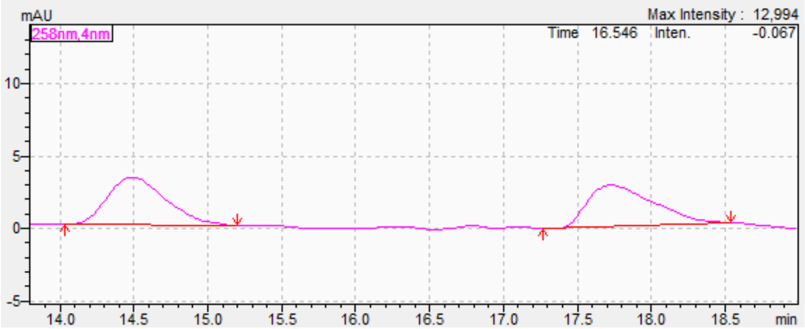


| Peak | Ret. Time | Area% | Height | Mark | Conc. |
|---|---|---|---|---|---|
| 1 | 14.489 | 50.943 | 3306 | M | 0.000 |
| 2 | 17.727 | 49.057 | 2849 | M | 0.000 |
| Total | | 100.000 | 6155 | | 0.000 |

**12h from the $Cu(OAc)_2$/W8 reaction:**

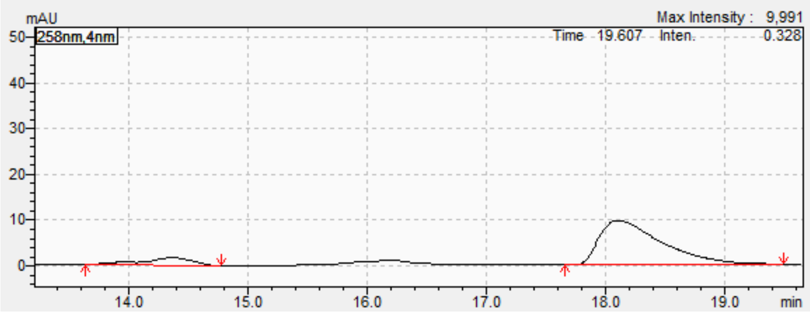


| Peak | Ret. Time | Area% | Height | Mark | Conc. |
|---|---|---|---|---|---|
| 1 | 14.364 | 12.610 | 1734 | M | 12.610 |
| 2 | 18.102 | 87.390 | 9733 | M | 87.390 |
| Total | | 100.000 | 11467 | | 100.000 |

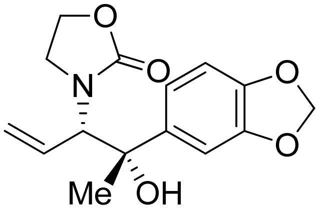

*Chiral HPLC analysis (Chiralcel OD-3 x 250 mm, heptane/isopropanol = 90/10, flow rate = 1.0 mL/min, λ = 190 nm) $t_R$ = 26.9 min (minor), 29.8 min (major):*

**Racemic 12i:**

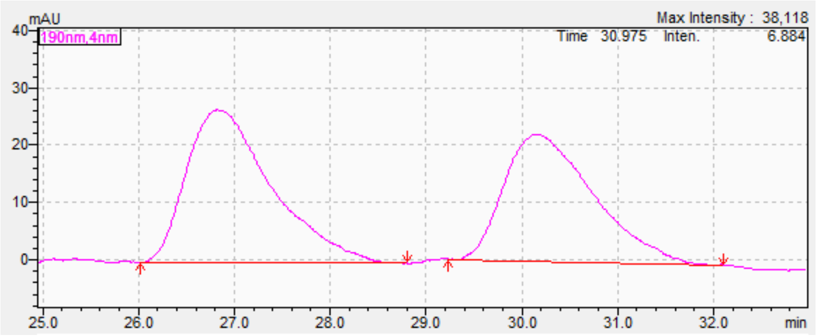

| Peak | Ret. Time | Area% | Height | Mark | Conc. |
|---|---|---|---|---|---|
| 1 | 26.819 | 53.696 | 26762 | M | 0.000 |
| 2 | 30.140 | 46.304 | 22111 | M | 0.000 |
| Total | | 100.000 | 48873 | | 0.000 |

**12i from the Cu(OAc)$_2$/W8 reaction:**

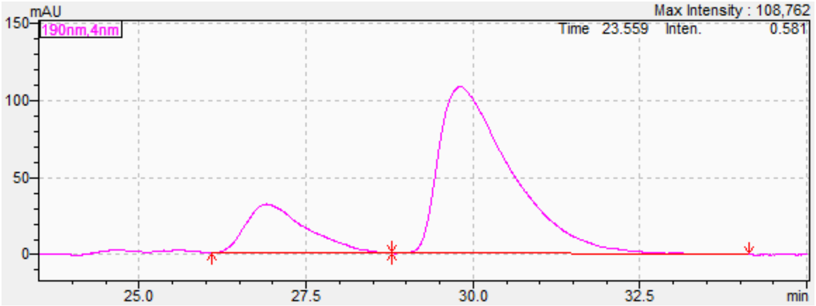

| Peak | Ret. Time | Area% | Height | Mark | Conc. |
|---|---|---|---|---|---|
| 1 | 26.910 | 19.715 | 31608 | M | 0.000 |
| 2 | 29.821 | 80.285 | 107955 | M | 0.000 |
| Total | | 100.000 | 139563 | | 0.000 |

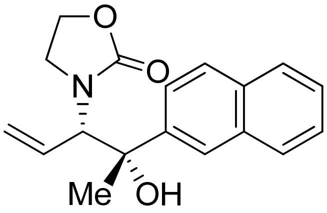


*Chiral HPLC analysis (Chiralpak AD-3 x 250 mm, heptane/isopropanol = 90/10, flow rate = 1.0 mL/min, λ = 254 nm) $t_R$ = 18.4 min (minor), 20.5 min (major):*

**Racemic 12j:**

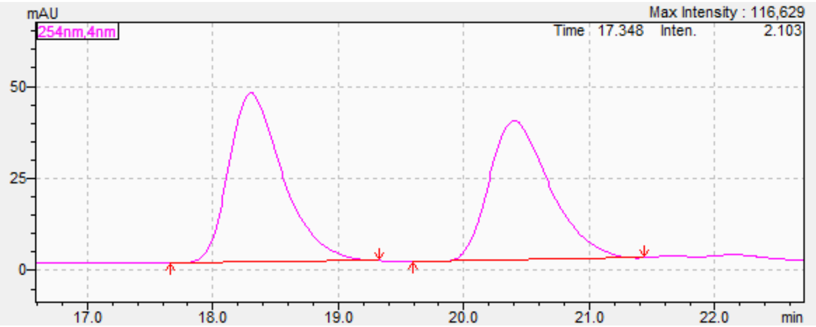


| Peak | Ret. Time | Area% | Height | Mark | Conc. |
|---|---|---|---|---|---|
| 1 | 18.304 | 51.801 | 45920 | M | 0.000 |
| 2 | 20.402 | 48.199 | 37807 | M | 0.000 |
| Total | | 100.000 | 83727 | | 0.000 |

**12j from the Cu(OAc)$_2$/W8 reaction:**

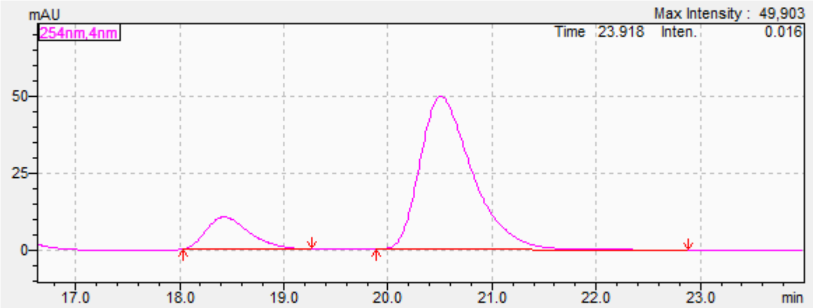


| Peak | Ret. Time | Area% | Height | Mark |
|---|---|---|---|---|
| 1 | 18.426 | 14.644 | 10430 | M |
| 2 | 20.510 | 85.356 | 49691 | M |
| Total | | 100.000 | 60121 | |

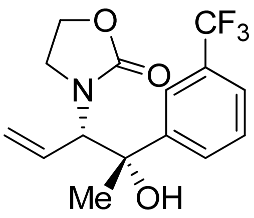


*Chiral HPLC analysis (Chiralcel OD-3 x 250 mm, heptane/isopropanol = 90/10, flow rate = 1.0 mL/min, λ = 254 nm) $t_R$ = 10.0 min (minor), 11.4 min (major):*

**Racemic 12k:**

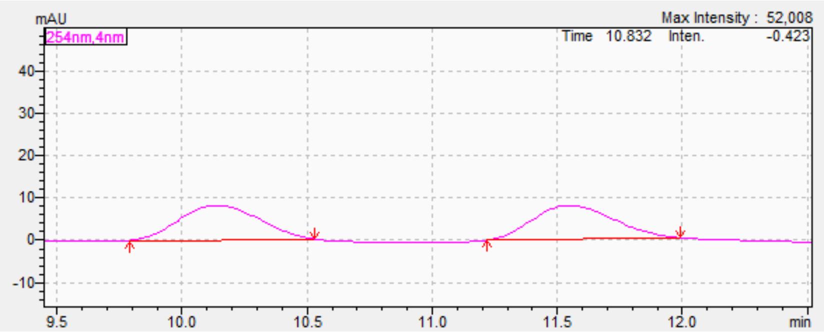


| Peak | Ret. Time | Area% | Height | Mark | Conc. |
|---|---|---|---|---|---|
| 1 | 10.140 | 50.030 | 8151 | M | 0.000 |
| 2 | 11.549 | 49.970 | 7961 | M | 0.000 |
| Total | | 100.000 | 16112 | | 0.000 |

**12k from the Cu(OAc)$_2$/W8 reaction:**

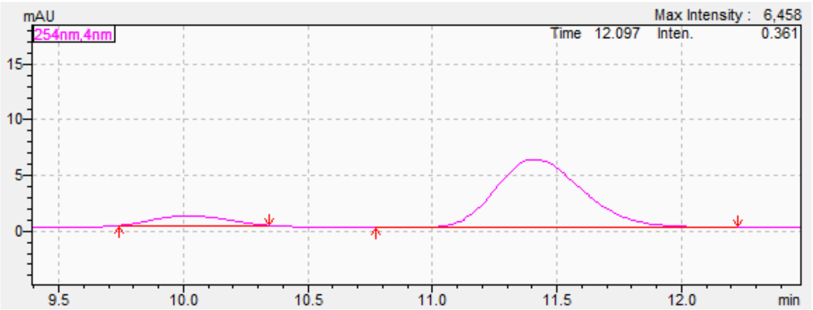


| Peak | Ret. Time | Area% | Height | Mark | Conc. |
|---|---|---|---|---|---|
| 1 | 10.024 | 10.401 | 868 | M | 0.000 |
| 2 | 11.412 | 89.599 | 6144 | M | 0.000 |
| Total | | 100.000 | 7012 | | 0.000 |

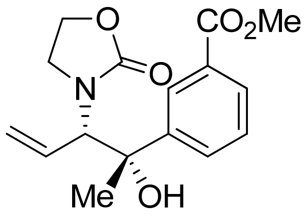

*Chiral HPLC analysis (Chiralcel OD-3 x 250 mm, heptane/isopropanol = 90/10, flow rate = 1.0 mL/min, λ = 254 nm) $t_R$ = 11.9 min (minor), 15.1 min (major):*

**Racemic 12l:**

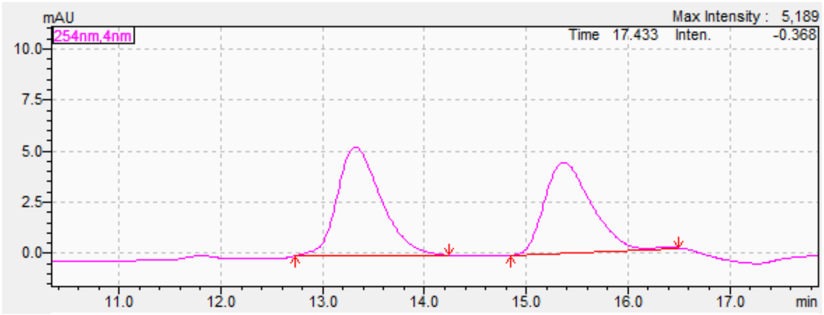


| Peak | Ret. Time | Area% | Height | Mark | Conc. |
|---|---|---|---|---|---|
| 1 | 13.320 | 53.086 | 5302 | M | 0.000 |
| 2 | 15.368 | 46.914 | 4429 | M | 0.000 |
| Total | | 100.000 | 9731 | | 0.000 |

**12l from the Cu(OAc)$_2$/W8 reaction:**

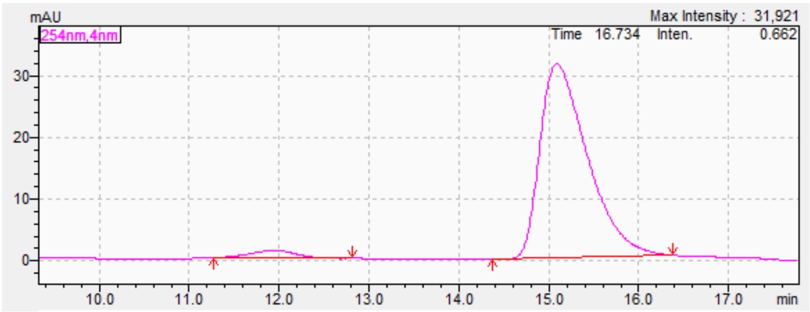


| Peak | Ret. Time | Area% | Height | Mark | Conc. |
|---|---|---|---|---|---|
| 1 | 11.947 | 3.445 | 1254 | M | 0.000 |
| 2 | 15.090 | 96.555 | 31417 | M | 0.000 |
| Total | | 100.000 | 32671 | | 0.000 |

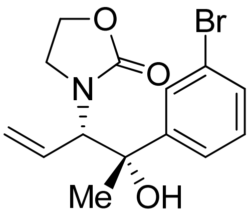


*Chiral HPLC analysis (Chiralpak AD-3 x 250 mm, heptane/isopropanol = 90/10, flow rate = 1.0 mL/min, λ = 220 nm)* $t_R$ *= 10.1 min (major), 10.7 min (minor):*

**Racemic 12m:**

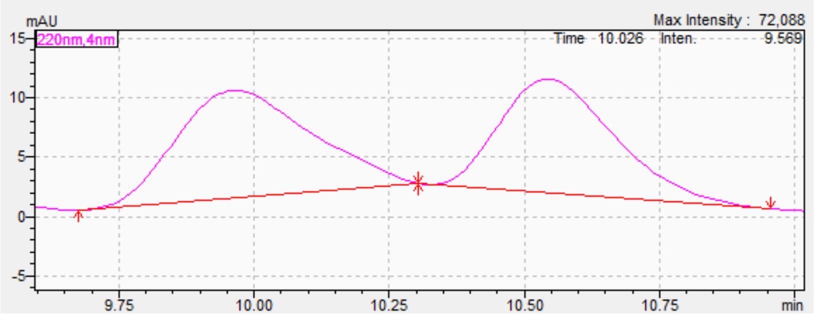


| Peak | Ret. Time | Area% | Height | Mark | Conc. |
|---|---|---|---|---|---|
| 1 | 9.966 | 52.848 | 9083 | M | 0.000 |
| 2 | 10.543 | 47.152 | 9589 | M | 0.000 |
| Total | | 100.000 | 18672 | | 0.000 |

**12m from the Cu(OAc)$_2$/W8 reaction (Method B):**

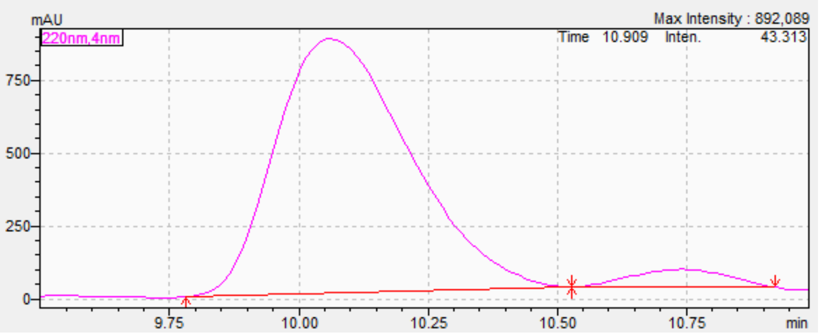


| Peak | Ret. Time | Area% | Height | Mark | Conc. |
|---|---|---|---|---|---|
| 1 | 10.062 | 95.273 | 872763 | M | 95.273 |
| 2 | 10.741 | 4.727 | 61775 | M | 4.727 |
| Total | | 100.000 | 934538 | | 100.000 |

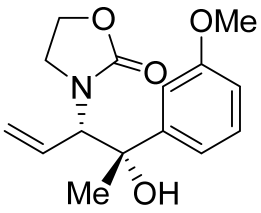


*Chiral HPLC analysis (Chiralcel OD-3 x 250 mm, heptane/isopropanol = 90/10, flow rate = 1.0 mL/min, λ = 285 nm) $t_R$ = 24.0 min (minor), 32.6 min (major):*

**Racemic 12n:**

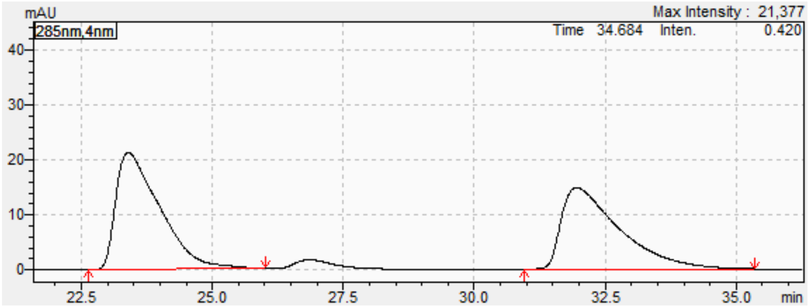


| Peak | Ret. Time | Area% | Height | Mark | Conc. |
|---|---|---|---|---|---|
| 1 | 23.396 | 52.108 | 21289 | M | 52.108 |
| 2 | 31.954 | 47.892 | 14863 | M | 47.892 |
| Total | | 100.000 | 36152 | | 100.000 |

**12n from the Cu(OAc)$_2$/W8 reaction:**

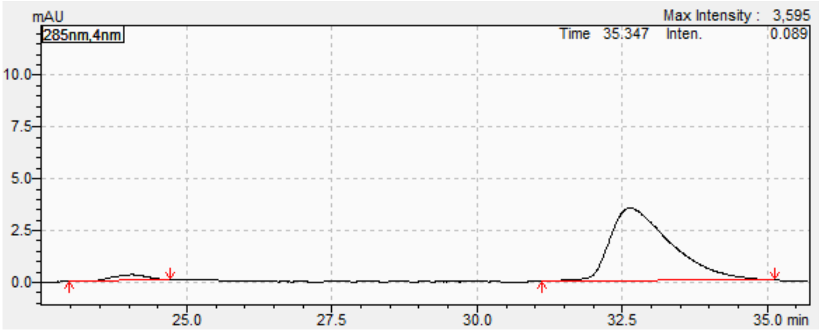


| Peak | Ret. Time | Area% | Height | Mark | Conc. |
|---|---|---|---|---|---|
| 1 | 24.045 | 4.292 | 288 | M | 4.292 |
| 2 | 32.647 | 95.708 | 3525 | M | 95.708 |
| Total | | 100.000 | 3813 | | 100.000 |

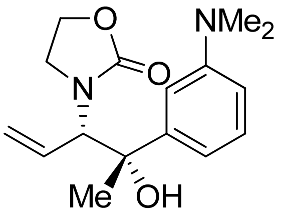

*Chiral HPLC analysis (Chiralpak AD-3 x 250 mm, heptane/isopropanol = 90/10, flow rate = 1.0 mL/min, λ = 190 nm) $t_R$ = 13.4 min (major), 14.7 min (minor):*

**Racemic 12o:**

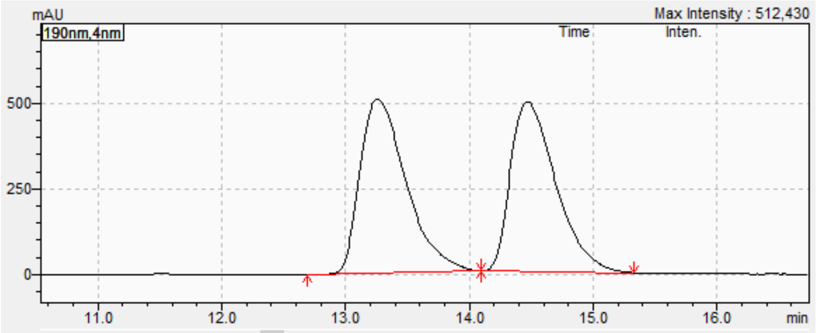


| Peak | Ret. Time | Area% | Height | Mark | Conc. |
|---|---|---|---|---|---|
| 1 | 13.259 | 50.931 | 508325 | M | 50.931 |
| 2 | 14.469 | 49.069 | 496699 | M | 49.069 |
| Total | | 100.000 | 1005023 | | 100.000 |

**12o from the Cu(OAc)$_2$/W8 reaction::**

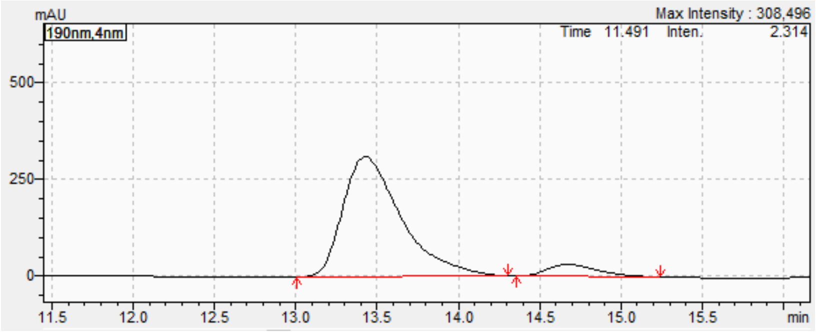


| Peak | Ret. Time | Area% | Height | Mark | Conc. |
|---|---|---|---|---|---|
| 1 | 13.426 | 91.997 | 309634 | M | 91.997 |
| 2 | 14.672 | 8.003 | 31327 | M | 8.003 |
| Total | | 100.000 | 340961 | | 100.000 |

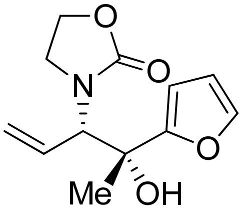

*Chiral HPLC analysis (Chiralcel OD-3 x 190 mm, heptane/isopropanol = 90/10, flow rate = 1.0 mL/min, λ = 190 nm) $t_R$ = 13.6 min (major), 16.4 min (minor):*

**Racemic 12p:**

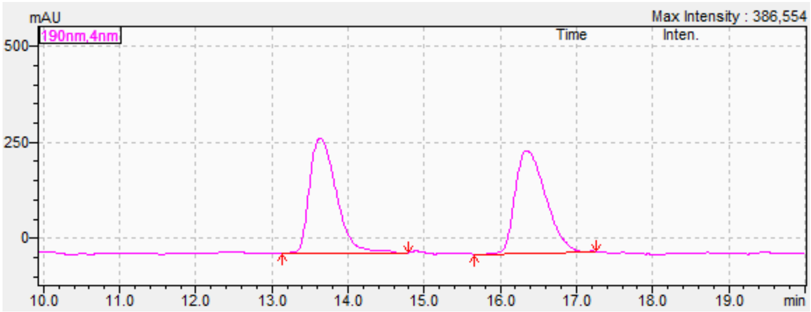


| Peak | Ret. Time | Area% | Height | Mark | Conc. |
|---|---|---|---|---|---|
| 1 | 13.633 | 49.549 | 299112 | M | 0.000 |
| 2 | 16.347 | 50.451 | 266935 | M | 0.000 |
| Total | | 100.000 | 566047 | | 0.000 |

**12p from the Cu(OAc)$_2$/W8 reaction:**

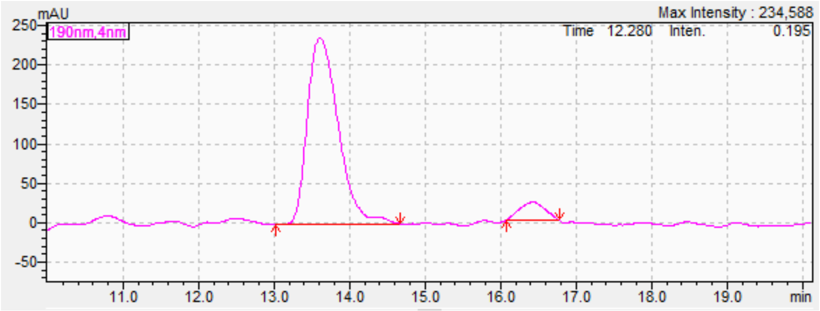


| Peak | Ret. Time | Area% | Height | Mark | Conc. |
|---|---|---|---|---|---|
| 1 | 13.605 | 92.354 | 236268 | M | 0.000 |
| 2 | 16.426 | 7.646 | 23189 | M | 0.000 |
| Total | | 100.000 | 259458 | | 0.000 |

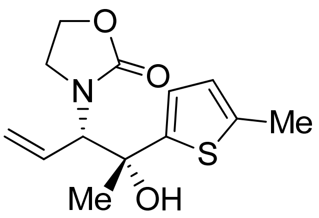

*Chiral HPLC analysis (Chiralpak AD-3 x 254 mm, heptane/isopropanol = 95/5, flow rate = 1.0 mL/min, λ = 254 nm) $t_R$ = 20.3 min (minor), 22.1 min (major):*

**Racemic 12q:**

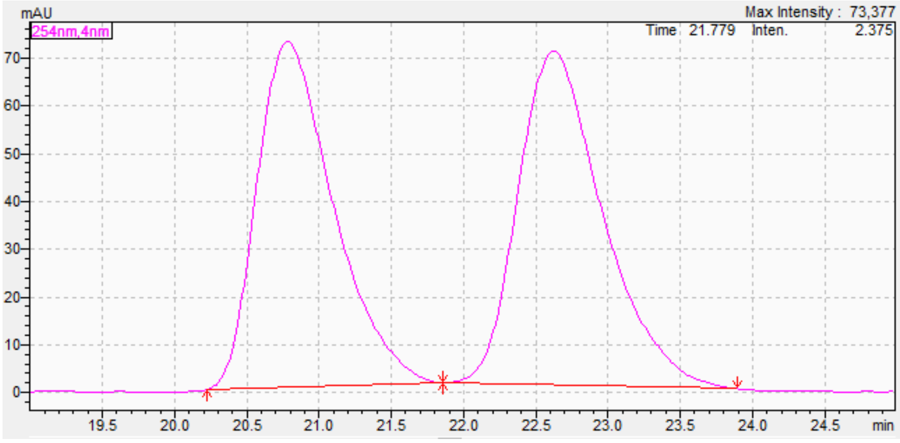


**12q from the Cu(OAc)$_2$/W8 reaction:**

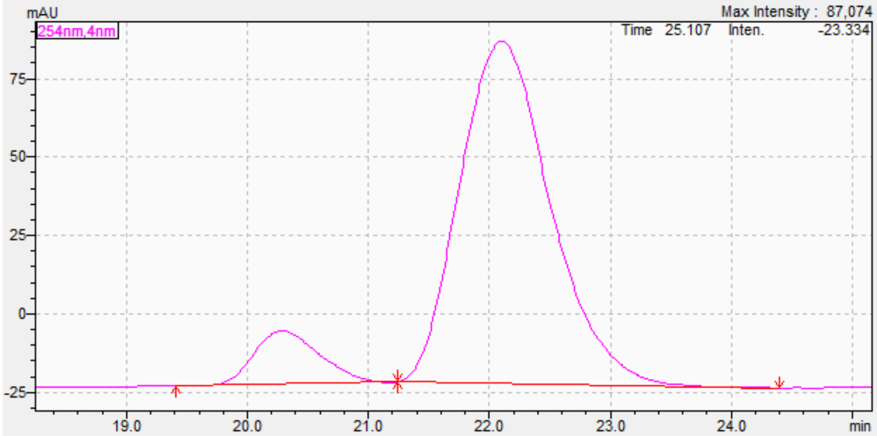


| Peak# | Ret. Time | Area% | Height | Mark |
|---|---|---|---|---|
| 1 | 20.287 | 9.962 | 16965 | M |
| 2 | 22.103 | 90.038 | 109340 | M |
| Total | | 100.000 | 126305 | |

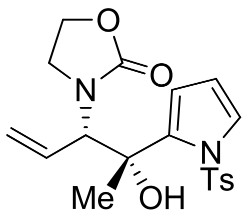

*Chiral HPLC analysis (ChiralpakAD-3 x 220 mm, heptane/isopropanol = 90/10, flow rate = 1.0 mL/min, λ = 220 nm) $t_R$ = 17.7 min (minor), 19.2 min (major):*

**Racemic 12r:**

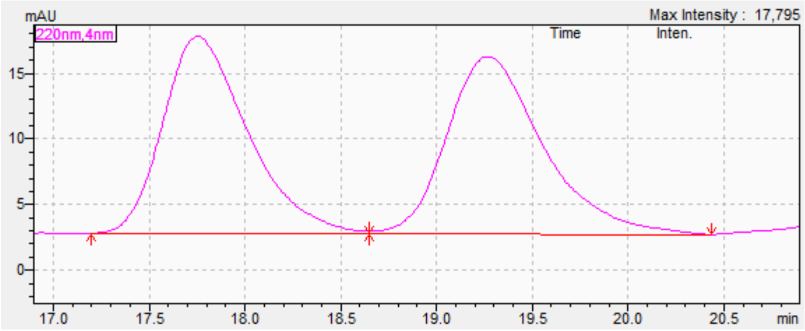


| Peak | Ret. Time | Area% | Height | Mark | Conc. |
|---|---|---|---|---|---|
| 1 | 17.754 | 49.440 | 14981 | | 0.000 |
| 2 | 19.269 | 50.560 | 13508 | V | 0.000 |
| Total | | 100.000 | 28489 | | 0.000 |

**12r from the $Cu(OAc)_2$/W8 reaction:**

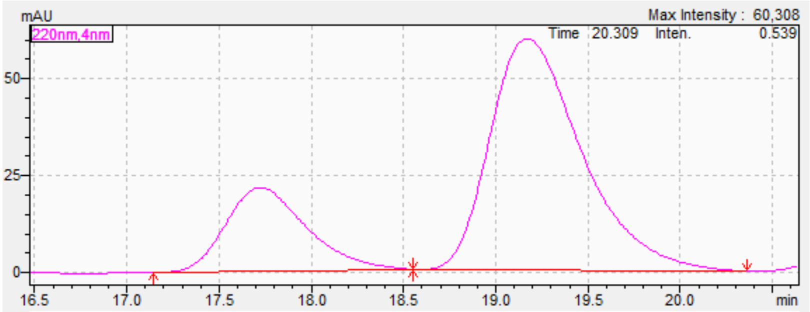


| Peak | Ret. Time | Area% | Height | Mark | Conc. |
|---|---|---|---|---|---|
| 1 | 17.724 | 24.010 | 21470 | M | 0.000 |
| 2 | 19.169 | 75.990 | 59608 | M | 0.000 |
| Total | | 100.000 | 81078 | | 0.000 |

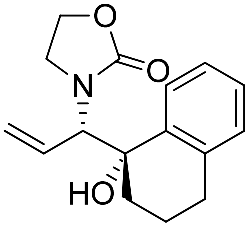

*Chiral HPLC analysis (Chiralpak AD-3 x 210 mm, heptane/isopropanol = 95/5, flow rate = 1.0 mL/min, λ = 210 nm) $t_R$ = 26.6 min (major), 29.3 min (minor):*

**Racemic 12s:**

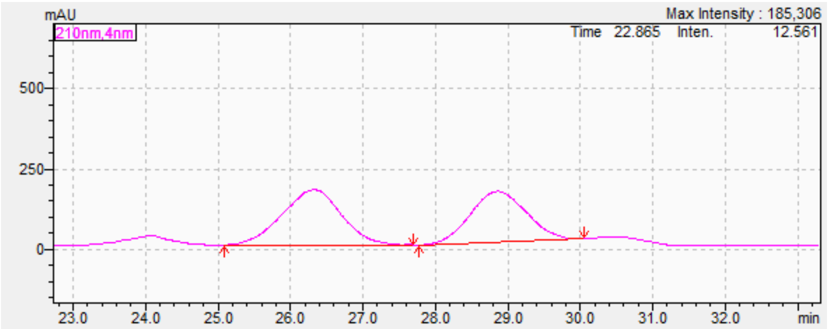


| Peak | Ret. Time | Area% | Height | Mark | Conc. |
|---|---|---|---|---|---|
| 1 | 26.333 | 53.326 | 171682 | M | 0.000 |
| 2 | 28.864 | 46.674 | 158237 | M | 0.000 |
| Total | | 100.000 | 329919 | | 0.000 |

**12s from the $Cu(OAc)_2$/W8 reaction:**

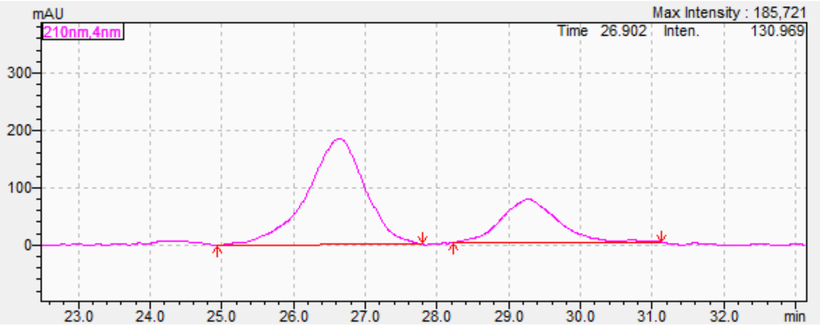


| Peak | Ret. Time | Area% | Height | Mark | Conc. |
|---|---|---|---|---|---|
| 1 | 26.638 | 71.600 | 185354 | M | 0.000 |
| 2 | 29.276 | 28.400 | 75477 | M | 0.000 |
| Total | | 100.000 | 260830 | | 0.000 |

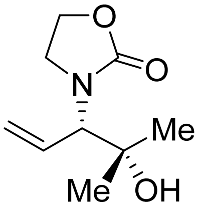

*Chiral HPLC analysis (Chiralcel OD-3 x 250 mm, heptane/isopropanol = 90/10, flow rate = 1.0 mL/min, λ = 205 nm)* $t_R$ *= 10.4 min (minor), 11.1 min (major):*

**Racemic 12t:**

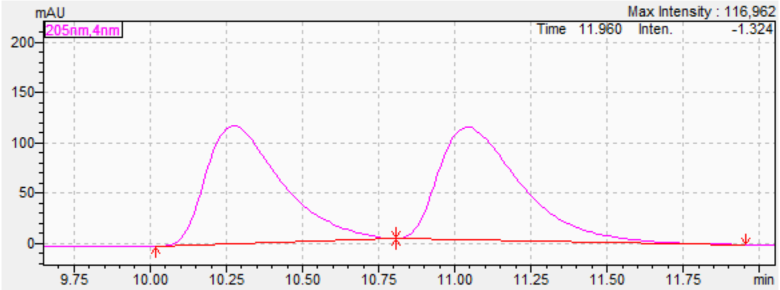


| Peak | Ret. Time | Area% | Height | Mark | Conc. |
|---|---|---|---|---|---|
| 1 | 10.273 | 48.961 | 117290 | M | 48.961 |
| 2 | 11.042 | 51.039 | 111953 | M | 51.039 |
| Total | | 100.000 | 229243 | | 100.000 |

**12t from the $Cu(OAc)_2$/W8 reaction:**

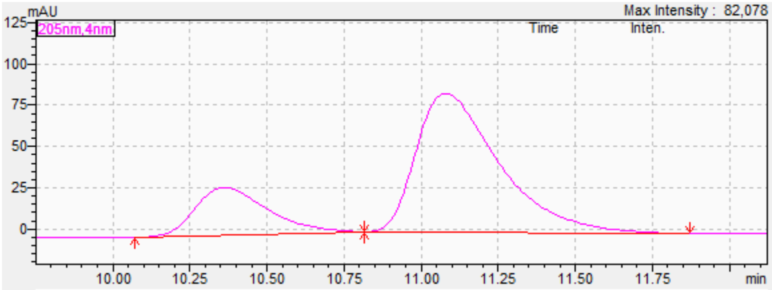


| Peak | Ret. Time | Area% | Height | Mark | Conc. |
|---|---|---|---|---|---|
| 1 | 10.362 | 22.955 | 28738 | M | 0.000 |
| 2 | 11.080 | 77.045 | 83956 | M | 0.000 |
| Total | | 100.000 | 112694 | | 0.000 |

## Mechanistic Experiments (Scheme 3):

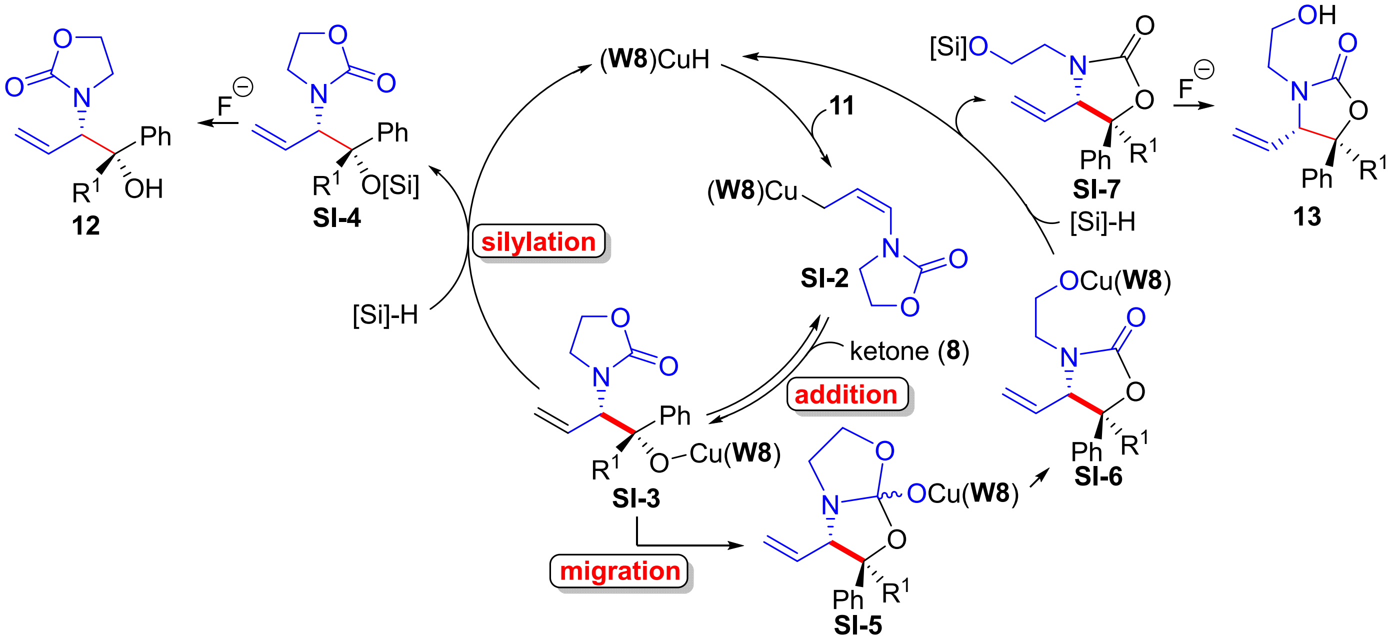


**Figure SI-1. Proposed catalytic cycle**

Hydrometalation of **11** by a **W8**CuH catalyst affords **SI-2**[8] that adds to ketone **8** through a closed chair-like transition state[2,6,9,10] providing branched intermediate **SI-3**. The addition step (**SI-2** + **8** → **SI-3**) is likely stereodetermining, however, the enantiopurity of product **12** derived from this intermediate will be affected if this addition step were reversible. For instance, the subsequent silylation or migration steps of **SI-3** for catalytic turnover affording products **12** or **13**, respectively may enhance or erode the initial stereoselectivity set in the addition step since turnover of the initially formed diastereomeric mixture of **SI-3** will proceed at different rates through diastereomeric transition states. Under this scenario, the addition step could be highly stereoselective providing **SI-3** in high d.r., but if the minor diastereomer undergoes migration to **SI-5** faster than the major one, overall poor enantioselectivity of **13** would be obtained, as was observed. This effect is exacerbated when using more sterically demanding ketones (*e.g.* propiophenone: $R^1$ = Et) whereby the migration rate would increase due to the enhanced Thorpe-Ingold effect while the silylation rate may decrease due to the increased steric demand. This can account for the increased amounts of **13** obtained when utilizing propiophenone.

### Attempted retroallylation of 12a:

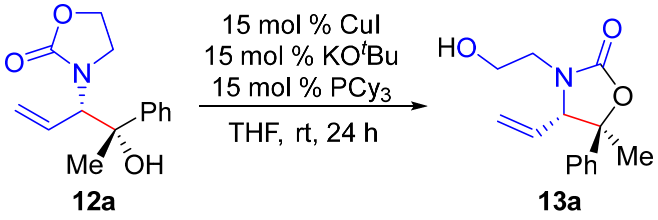


A crimp-cap vial with magnetic stir-bar was charged with 1.7 mg (0.0091 mmol) of CuI, 1.0 mg (0.0091 mmol) of KO$^t$Bu, 2.6 mg (0.0091 mmol) of $PCy_3$, and 0.20 mL of THF in the glove-box, and the resultant mixture

was allowed to stir for 30 min. Next, **12a** (15.0 mg, 0.0607 mmol) was charged and the vial was sealed, removed from the glove-box, and allowed to stir at rt for 24 h. To the mixture was then added 0.2 mL of 50% aqueous $NH_4OH$ and 1.0 mL of water followed by extraction with $CH_2Cl_2$ (2x1mL). The combined organics were dried with anhydrous $Na_2SO_4$ and concentrated *in vacuo*. The crude mixture was then analyzed by quantitative $^1$H NMR spectroscopy using dimethyl fumarate as analytical standard.

**Synthesis of *b*-17:**

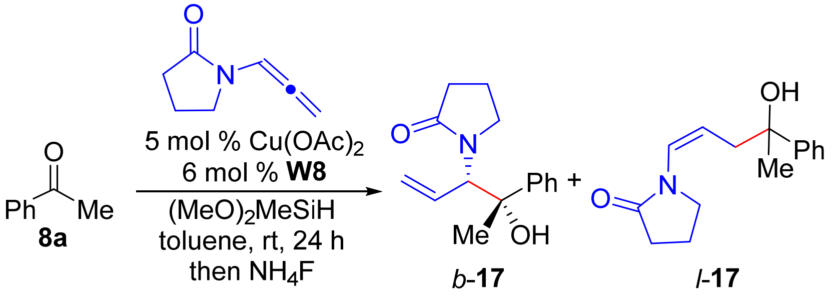


The Cu-catalyzed reductive coupling of *N*-allenyl pyrrolidinone and acetophenone (**8a**) was performed according to the Method A general procedure. The products were purified by silica gel chromatography (eluent: 0 – 30% EtOAc in $CH_2Cl_2$) to provide 35.5 mg (58%) of *b*-**17** as a white solid as a single diastereomer and 22.0 mg (36%) of *l*-**17** as a thick oil. The enantiopurity of *b*-**17** was determined by chiral HPLC analysis relative to authentic racemic material prepared using $PCy_3$ as ligand in the reductive coupling reaction. The stereochemistry of *b*-**17** was assigned by analogy to that of **12a**

**1-((3S,4S)-4-hydroxy-4-phenylpent-1-en-3-yl)pyrrolidin-2-one** (*b*-**17**): $R_f$ = 0.40 (30% EtOAc/$CH_2Cl_2$). $^1$HNMR ($CDCl_3$, 600 MHz) δ: 7.46 (d, *J* = 7.5 Hz, 2H), 7.32 (t, *J* = *8.2* Hz, 2H), 7.22 (t, *J* = *7.4* Hz , 1H), 6.37 (ddd, *J* = 18.1 Hz, *J* = 9.6 Hz, 8.50 Hz, 1H), 6.33 (s, 1H), 5.36 (d, *J* = 9.6 Hz, 1H), 5.24 (d, *J* = 17.4 Hz, 1H), 3.92 (d, *J* = *8.4* Hz, 1H), 3.32 (dt, *J* = *10*.8 Hz, 6.9 Hz, 1H), 2.88 (dt, *J* = *12.4 Hz,* 5.5 Hz, 1H), 2.17 (ddd, *J* = 17.6 Hz, *J* = *10.0* Hz, *J* = 7.8 Hz, 1H), 2.02 (ddd, *J* = *1*7.3 Hz, *J* = 10.0 Hz, *J* = 6.5 Hz, 1H), 1.72 – 1.82 (m, 1H), 1.53 – 1.61 (m, 1H), 1.50 (s, 3H) ppm. $^{13}$C NMR (151 MHz, $CDCl_3$): δ 176.4, 146.7, 131.3, 127.9, 126.6, 124.6, 119.4, 76.75, 70.3, 50.6, 31.6, 28.5, 18.9 ppm. HRMS (DART) *m/z* calcd for $C_{15}H_{20}NO_2$ $[M + H]^+$: 246.1494; Found $[M + H]^+$: 246.1485.

**(*Z*)-1-(4-hydroxy-4-phenylpent-1-en-1-yl)pyrrolidin-2-one** (*l*-**17**): $R_f$ = 0.21 (30% EtOAc/ $CH_2Cl_2$). $R_f$ = 0.12 (30% EtOAc/hexanes). $^1$HNMR ($CDCl_3$, 600 MHz) δ: 7.47 (d, *J* = 7.6 Hz, 2H), 7.34 (t, *J* = 8.0 Hz, 2H), 7.23 (t, *J* = 7.6 Hz, 1H), 6.14 (d, *J* = 9.8 Hz, 1H), 4.94 (q, *J* = *8.4* Hz, 1H), 3.63 (t, *J* = 6.9 Hz, 2H), 3.57 (br s, 1H), 2.67 (p, *J* = 8.6 Hz, 2H), 2.43 (td, *J* =*8* .4 Hz, , *J* = 2.6 Hz, 2H), 2.06 (p, *J* = *7*.6 Hz, 2H), 1.58 (s, 3H) ppm. $^{13}$C NMR (151 MHz, $CDCl_3$): δ 174.6, 148.1, 128.2, 126.5, 125.2, 124.8, 115.4, 73.4, 49.3, 41.8, 30.8, 30.3, 18.3 ppm. HRMS (DART) *m/z* calcd for $C_{15}H_{20}NO_2$ $[M + H]^+$: 246.1494; Found $[M + H]^+$: 246.1484.

*Chiral HPLC analysis (Chiralcel OD-3 x 250 mm, heptane/isopropanol = 90/10, flow rate = 1.0 mL/min, λ = 190 nm) $t_R$ = 14.9 min (minor), 16.0 min (major):*

**Racemic *b*-17:**

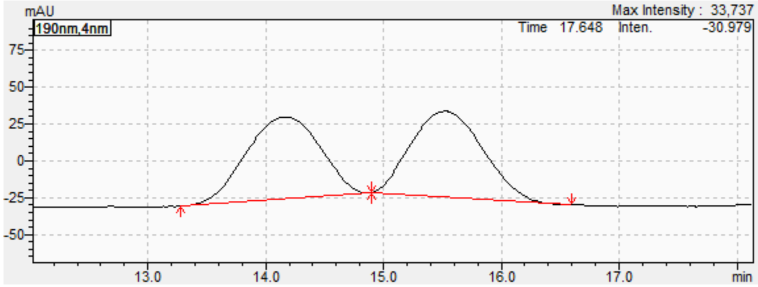


| Peak | Ret. Time | Area% | Height | Mark | Conc. |
|---|---|---|---|---|---|
| 1 | 14.153 | 49.760 | 55507 | M | 49.760 |
| 2 | 15.529 | 50.240 | 58213 | M | 50.240 |
| Total | | 100.000 | 113720 | | 100.000 |

***b*-17 from the Cu(OAc)$_2$/W8 reaction:**

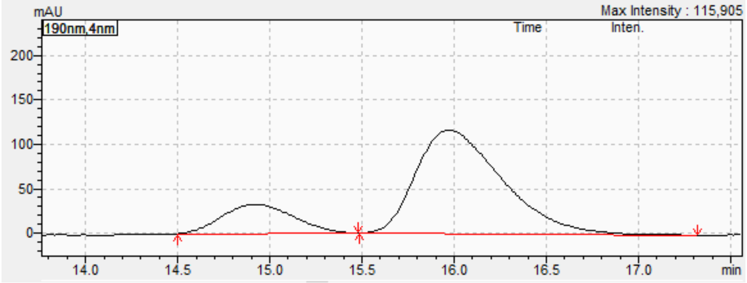


| Peak | Ret. Time | Area% | Height | Mark | Conc. |
|---|---|---|---|---|---|
| 1 | 14.920 | 18.336 | 49041 | M | 18.336 |
| 2 | 15.972 | 81.664 | 182395 | M | 81.664 |
| Total | | 100.000 | 231436 | | 100.000 |

**Retroallylation experiments employing *b*-17:**

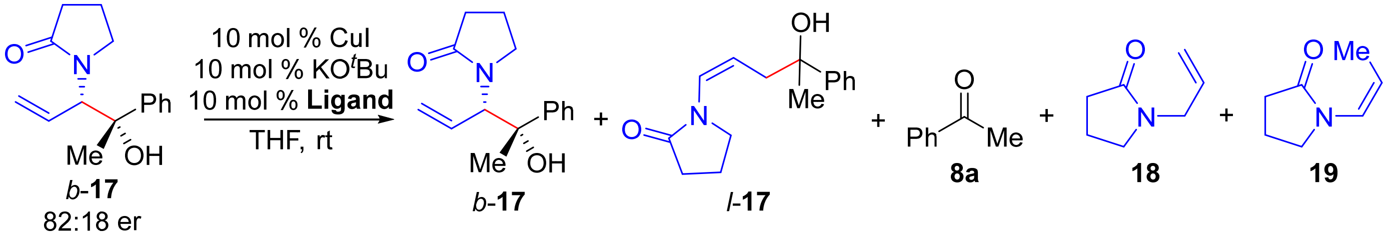


A crimp-cap vial with magnetic stir-bar was charged with 2.3 mg (0.012 mmol) of CuI, 1.4 mg (0.012 mmol) of KO$^t$Bu, 0.012 mmol of ligand (**W8** or PCy$_3$), and 0.50 mL of THF in the glove-box, and the resultant mixture was allowed to stir for 30 min. Next, *b*-**17** (30.0 mg, 0.122 mmol) was charged and the vial was sealed, removed from the glove-box, and allowed to stir at rt for 24 h. To the mixture was then added 0.5 mL of 50% aqueous $NH_4OH$ and 1.0 mL of water followed by extraction with EtOAc (3x2mL). The combined organics were dried with anhydrous $Na_2SO_4$ and concentrated *in vacuo*. The crude mixture was then analyzed by quantitative $^1$H NMR spectroscopy using dimethyl fumarate as analytical standard to determine the amounts of *b*-**17**, *l*-**17**, **8a**, **18**, and **19**. The identity of *N*-allyl pyrrolidine-2-one (**18**)[4] and (*Z*)-*N*-propenyl pyrrolidine-2-one (**19**)[5] were confirmed in relation to authentic material. The enantiopurity of recovered *b*-**17** (silica gel chromatography) was determined by chiral HPLC analysis.

**Reaction using W8 as ligand:**

44% *b*-**17** (70:30 er); 3% *l*-**17**; 28% acetophenone (**8a**); 23% *N*-allyl pyrrolidine-2-one (**18**); and 19% (*Z*)-*N*-propenyl pyrrolidine-2-one (**19**)

**$^1$H NMR ($CDCl_3$, 600 MHz) of the unpurified reaction mixture:**

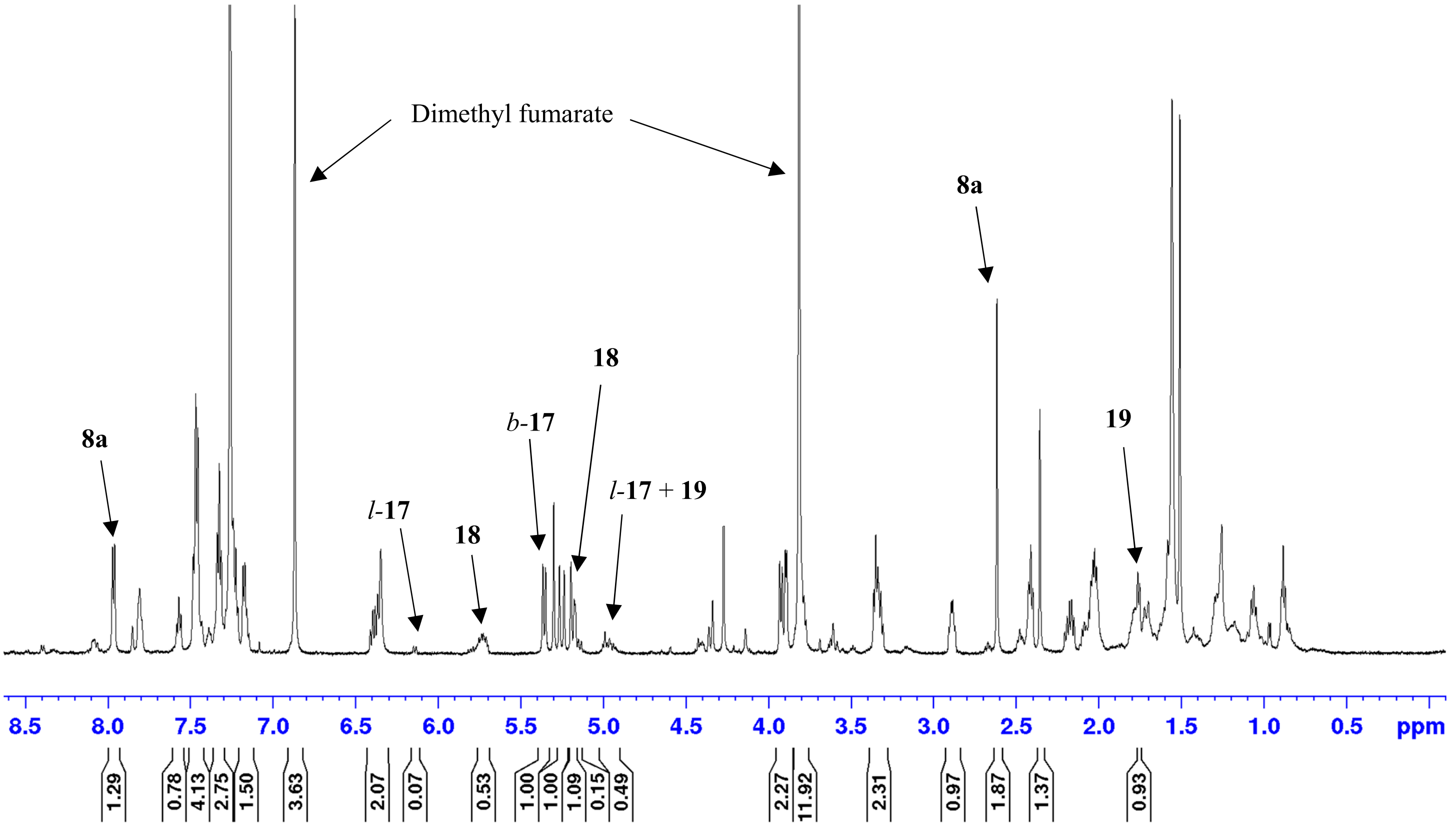


Re-isolated *b*-**17 from the CuI/W8/KO$^t$Bu reaction:**

*Chiral HPLC analysis (Chiralcel OD-3 x 250 mm, heptane/isopropanol = 90/10, flow rate = 1.0 mL/min, λ = 190 nm)*

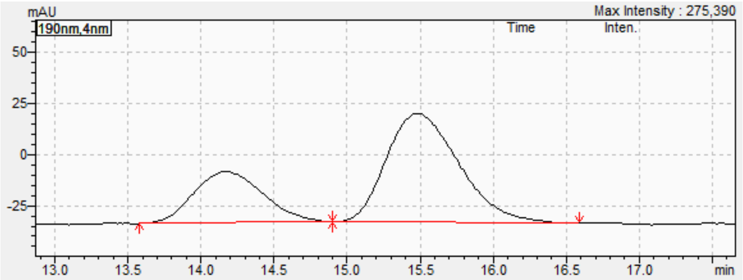


| Peak | Ret. Time | Area% | Height | Mark | Conc. |
|---|---|---|---|---|---|
| 1 | 14.158 | 30.963 | 24809 | M | 30.963 |
| 2 | 15.486 | 69.037 | 53117 | M | 69.037 |
| Total | | 100.000 | 77926 | | 100.000 |

**Reaction using $PCy_3$ as ligand:**

0% *b*-**17**; 43% *l*-**17**; 32% acetophenone (**8a**); 19% *N*-allyl pyrrolidine-2-one (**18**); and 15% (*Z*)-*N*-propenyl pyrrolidine-2-one (**19**)

**$^1$H NMR ($CDCl_3$, 600 MHz) of the unpurified reaction mixture:**

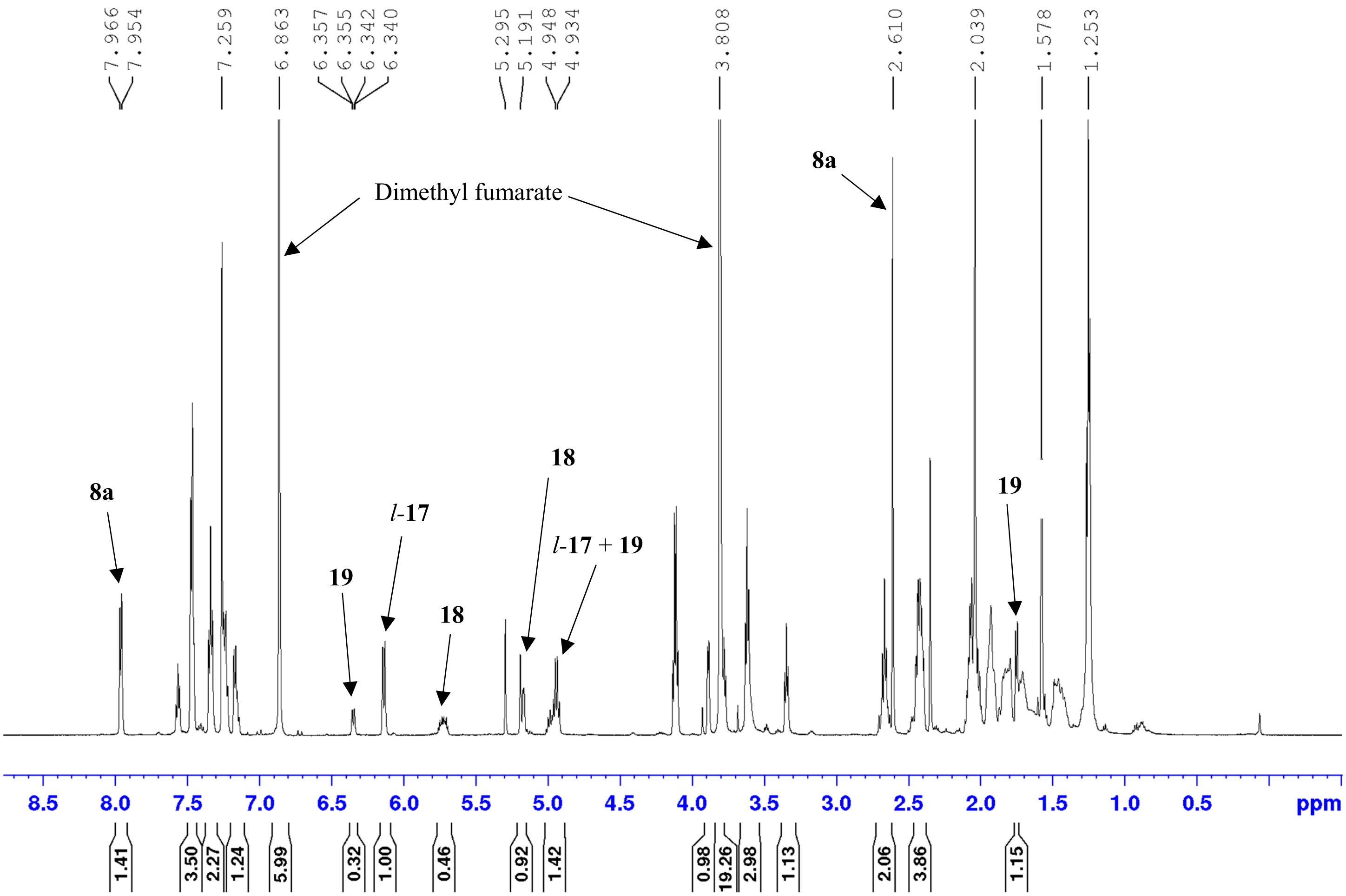


A proposed mechanism is outlined in **Figure SI-2**. Alcohol exchange leads to **SI-8** that upon retroallylation to release acetophenone (**8a**) affords *N*-allyl Cu-complex *l*-**SI-9** that can equilibrate to *b*-**SI-9**. Reaction of *b*-**SI-9** with acetophenone leads to formation of linear isomer *l*-**17** whereas reaction of *l*-**SI-9** with acetophenone regenerates *b*-**17** rationalizing the change in er for recovered *b*-**17** observed in this experiment. Protonolysis of *l*-**SI-9** and *b*-**SI-9** is proposed to occur through chair-like transition states **SI-TS1** and **SI-TS2**, respectively. The exclusive formation of the *Z*-stereoisomer of **19** in these experiments strongly suggests this type of protonolysis pathway that is similar to that for the addition of related analogues of *b*-**SI-9** to ketone electrophiles providing *Z*-enamides.[6,7] However, direct protonolysis of *l*-**SI-9** and *b*-**SI-9** cannot be ruled out.

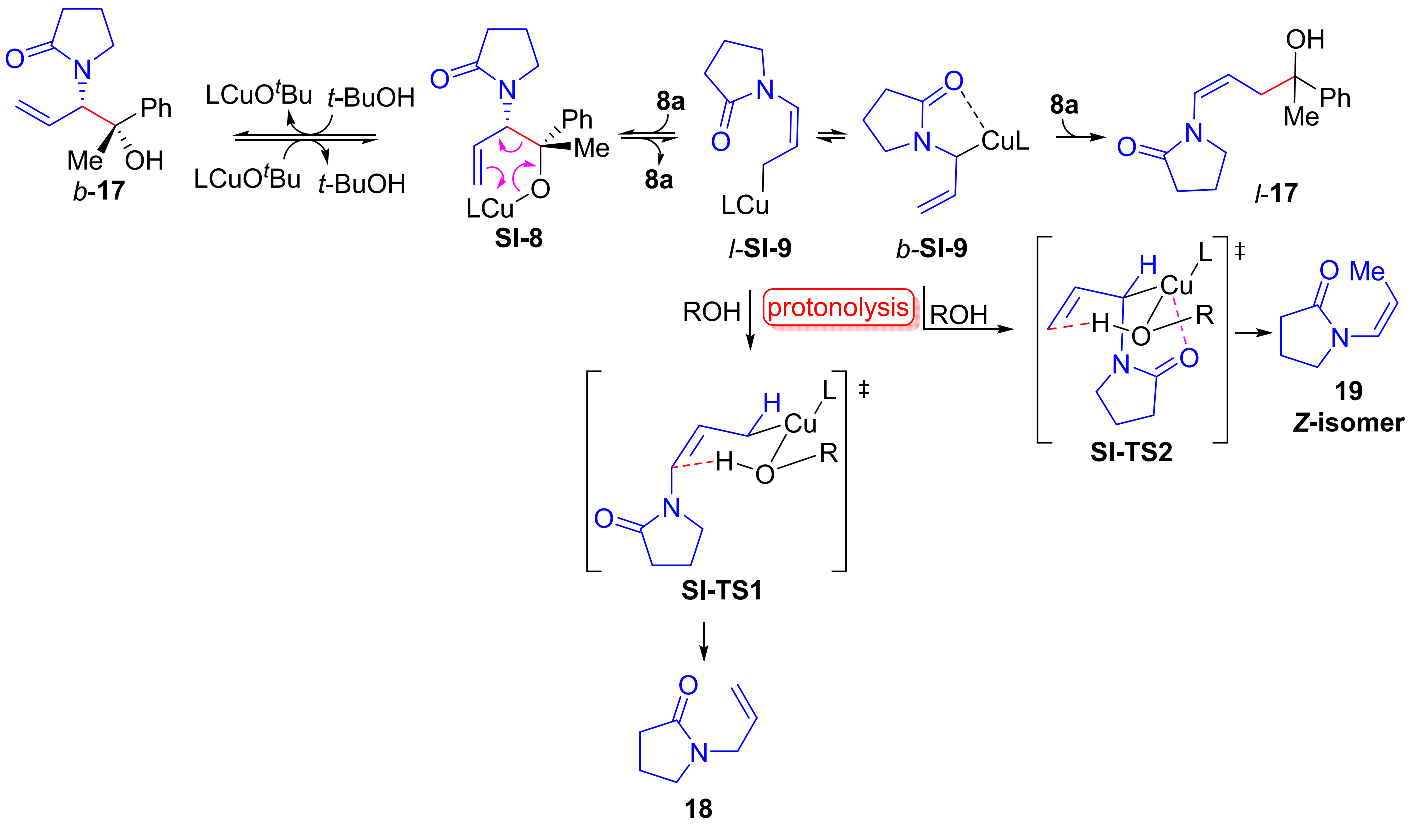


**Figure SI-2. Proposed Retroallylation Mechanism.**

**Analysis of product 12a enantiopurity throughout the progress of the reaction:**

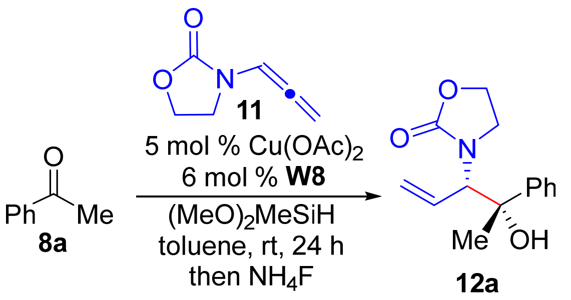


Three reactions were setup at the same time according to the Method A general procedure utilizing 9.7 μL (10 mg, 0.083 mmol) of acetophenone. Each reaction was quenched and worked up at different time points and **12a** was isolated and the enantiopurity determined (Table SI-4). The enantiopurity did not vary over time.

**Table SI-4: Enantiopurity of 12a *vs* Time.**

| Entry | Reaction time (h) | %**12a**[a] | **12a**:**13a**[b] | Er of **12a**[c] |
|---|---|---|---|---|
| 1 | 6 | 17 | 67:33 | 93:7 |
| 2 | 12 | 30 | 68:32 | 93:7 |
| 3 | 19 | 53 | 70:30 | 93:7 |

[a]Yield of **12a** determined by quantitative $^1$H NMR spectroscopy on the unpurified reaction mixture using dimethyl fumarate as the analytical standard. [b]The ratio was determined by $^1$H NMR spectroscopic analysis on the unpurified reaction mixture. [c]Enantiomeric ratios were determined by chiral HPLC analysis on purified material (flash chromatography).

**References:**


1. Bousfield, T. W.; Kimber, M. C. A simple one-pot preparation of *N*-allenyl amides, ureas, carbamates, and sulfonamides using a DMSO/$^t$BuOK protocol. *Tetrahedron Lett.* **2015**, *56*, 350 – 352.
2. Gargaro, S. L.; Klake, R. K.; Burns, K. L.; Elele, S. O.; Gentry, S. L.; Sieber, J. D. Access to a Catalytically Generated Umpolung Reagent through the Use of Cu-Catalyzed Reductive Coupling of Ketones and Allenes for the Synthesis of Chiral Vicinal Aminoalcohol Synthons. *Org. Lett.* **2019**, *21*, 9753–9758.
3. Kinoshita, A.; Higashino, M.; Yoshida, K.; Artani, Y.; Kakuuchi, A.; Hanada, D.; Takeda, H.; Naganawa, A.; Matsuya, H.; Ohmoto, K. Synthesis and evaluation of a potent, well-balanced $EP_2/EP_3$ dual agonist. *Biorg. Med. Chem.* **2018**, *26*, 200 – 214.
4. Mukherjee, P.; Widenhoefer, R. A. Gold(I)-Catalyzed Amination of Allylic Alcohols with Cyclic Ureas and Related Nucleophiles. *Org. Lett.* **2010**, *12*, 1184 – 1187.
5. Alcaide, B.; Almendros, P.; Alonso, J. M. A Practical Ruthenium-Catalyzed Cleavage of the Allyl Protecting Group in Amides, Lactams, Imides, and Congeners. *Chem. Eur. J.* **2006**, *12*, 2874 – 2879.
6. Klake, R. K.; Gargaro, S. L.; Gentry, S. L.; Elele, S. O.; Sieber, J. D. Development of a Strategy for Linear-Selective Cu-Catalyzed Reductive Coupling of Ketones and Allenes for the Synthesis of Chiral γ-Hydroxyaldehyde Equivalents. *Org. Lett.* **2019**, *21*, 7992 – 7998.
7. (a) Jang, H.; Jung, B.; Hoveyda, A. H. Catalytic Enantioselective Protoboration of Disubstituted Allenes. Access to Alkenylboron Compounds in High Enantiomeric Purity. *Org. Lett.* **2014**, *16*, 4658 – 4661. (b) Meng, F.; Jung, B.; Haeffner, F.; Hoveyda, A. H. NHC-Cu-Catalyzed Protoboration of Monosubstituted Allens. Ligand-Controlled Site Selectivity, Application to Synthesis and Mechanism. *Org. Lett.* **2013**, *15*, 1414 – 1417.
8. The allylcopper complex is represented as $\eta^1$ rather than $\eta^3$ (*i.e.* π-allyl) based on a number of computational studies which predict the $\eta^1$ form to be more stable than the $\eta^3$ form in Cu(allyl) complexes. See, ref. 9, 10c-e
9. Agrawal, T.; Martin, R. T.; Collins, S.; Wilhelm, Z.; Edwards, M. D.; Gutierrez, O.; Sieber, J. D. Access to Chiral Diamine Derivatives through Stereoselective Cu-Catalyzed Reductive Coupling of Imines and Allenamides. *J. Org. Chem.* **2021**, *86*, 5026 – 5046.
10. (a) Tsai, E. Y.; Liu, R. Y.; Yang, Y.; Buchwald, S. L. A Regio- and Enantioselective CuH-Catalyzed Ketone Allylation with Terminal Allenes. *J. Am. Chem. Soc.* **2018**, *140*, 2007 – 2011. (b) Liu, R. Y.; Zhou, Y.; Yang, Y.; Buchwald, S. L. Enantioselective Allylation Using Allene, a Petroleum Cracking Byproduct. *J. Am. Chem. Soc.* **2019**, *141*, 2251 – 2256. (c) Liu, T. Y.; Yang, Y.; Buchwald, S. L. Regiodivergent and Diastereoselective CuH-Catalyzed Allylation of Imines with Terminal Allenes. *Angew. Chem. Int. Ed.* **2016**, *55*, 14077 – 14080. (d) Li, C.; Liu, R. Y.; Jesikiewicz, L. T.; Yang, Y.; Liu, P.; Buchwald, S. L. CuH-Catalyzed Enantioselective Ketone Allylation with 1,3-Dienes: Scope, Mechanism, and Applications. *J. Am. Chem. Soc.* **2019**, *141*, 5062 – 5070. (e) Li, C.; Shin, K. Liu, R. Y.; Buchwald, S. L. Engaging Aldehydes in CuH-Catalyzed Reductive Coupling Reactions: Stereoselective Allylation with Unactivated 1,3-Diene Pronucleophiles. *Angew. Chem. Int. Ed.* **2019**, *58*, 17074 – 17080.

# NMR Data:

$^{1}H$ NMR ($CDCl_3$), 600 MHz

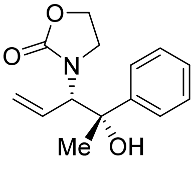

**12a**

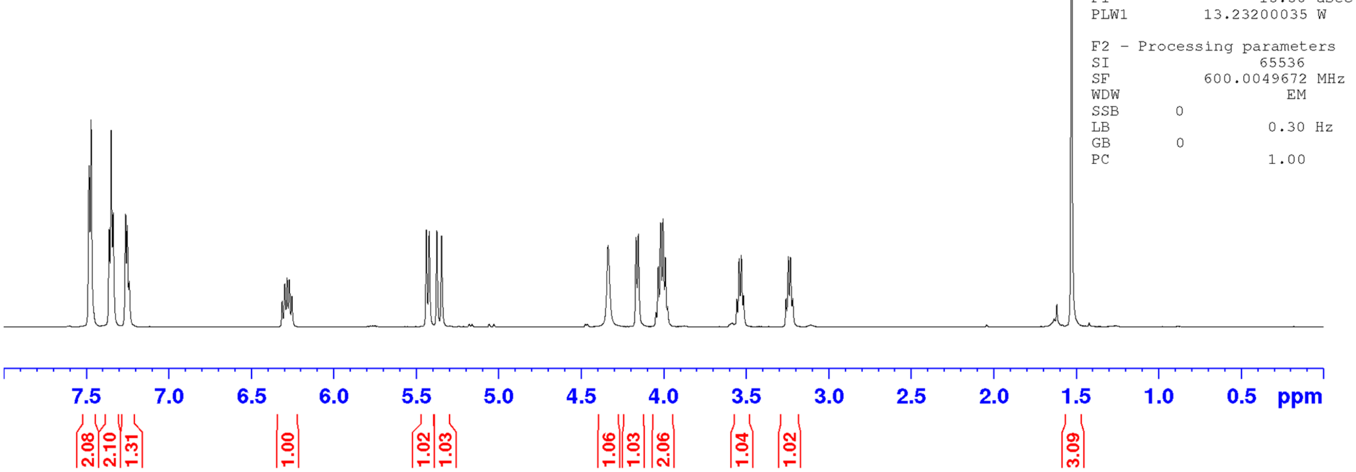


$^{13}C$ NMR ($CDCl_3$), 151 MHz

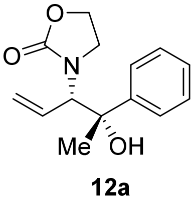

**12a**

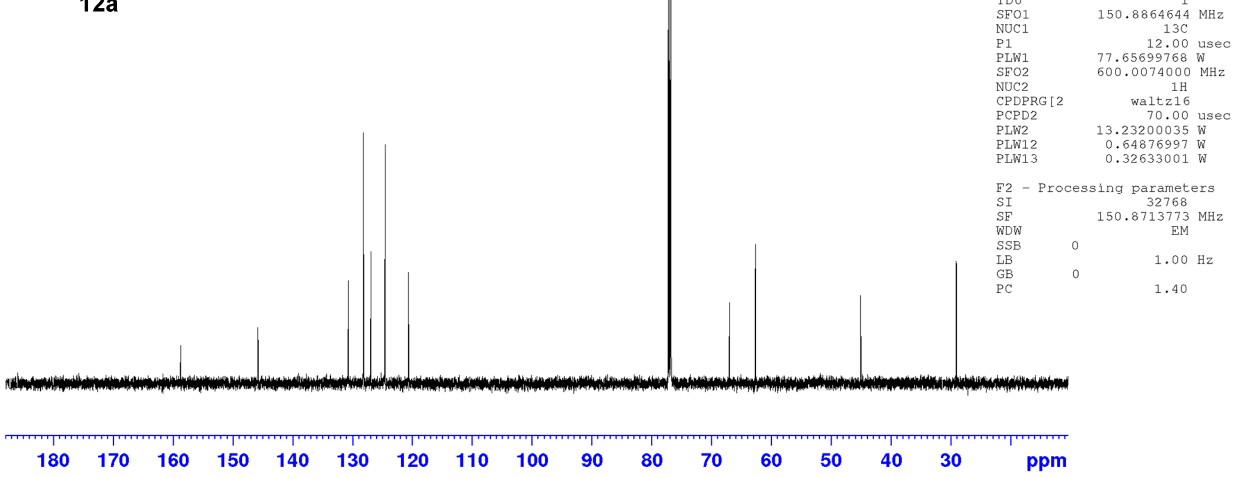

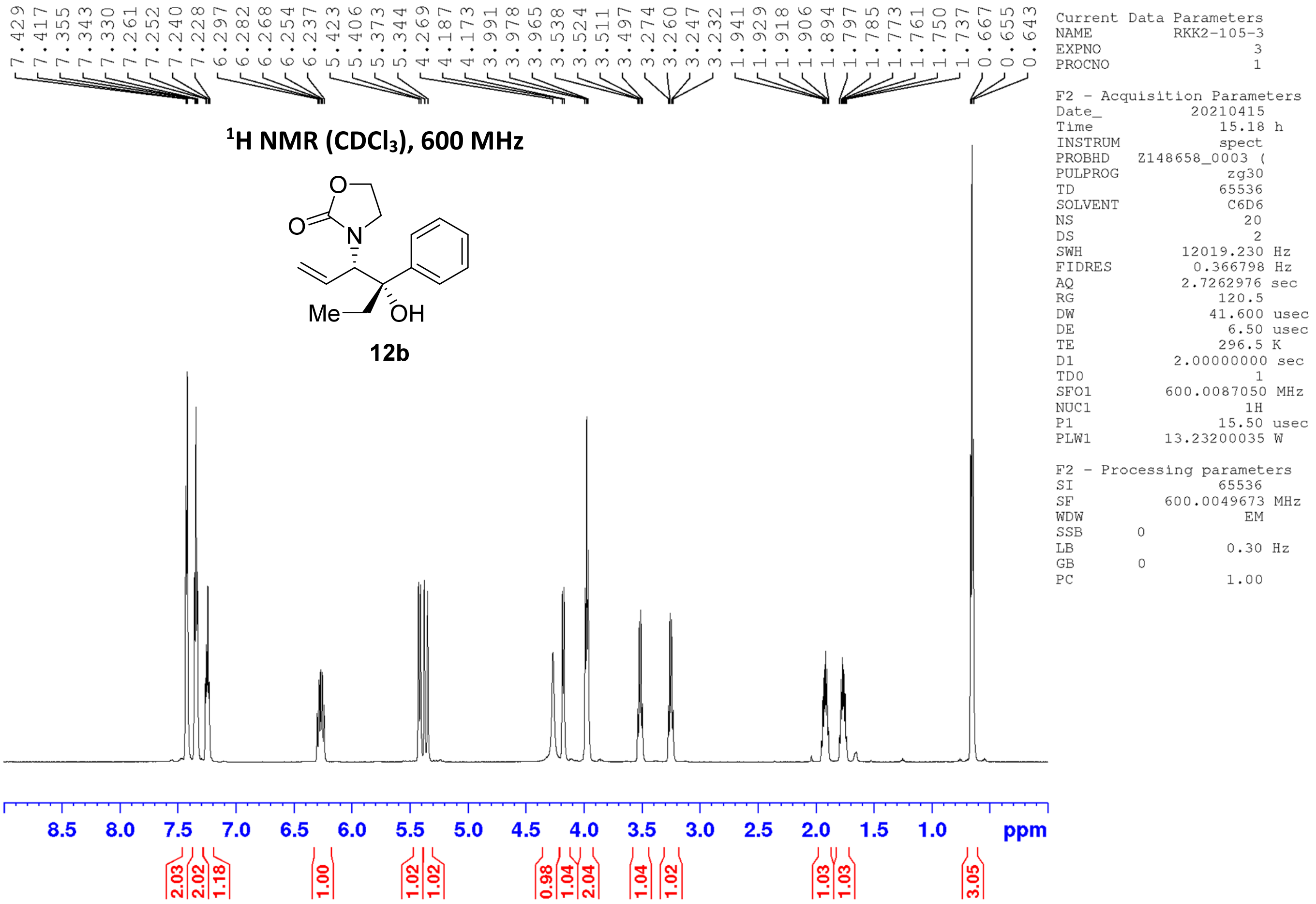

7.429 7.417 7.355 7.343 7.330 7.261 7.252 7.240 7.228 6.297 6.282 6.268 6.254 6.237 5.423 5.406 5.373 5.344 4.269 4.187 4.173 3.991 3.978 3.965 3.538 3.524 3.511 3.497 3.274 3.260 3.247 3.232 1.941 1.929 1.918 1.906 1.894 1.797 1.785 1.773 1.761 1.750 1.737 0.667 0.655 0.643
1H NMR (CDCl3), 600 MHz
O
N
O
Me
OH
12b
8.5 8.0 7.5 7.0 6.5 6.0 5.5 5.0 4.5 4.0 3.5 3.0 2.5 2.0 1.5 1.0 ppm
2.03 2.02 1.18 1.00 1.02 1.02 0.98 1.04 2.04 1.04 1.02 1.03 1.03 3.05
Current Data Parameters
NAME RKK2-105-3
EXPNO 3
PROCNO 1
F2 - Acquisition Parameters
Date_ 20210415
Time 15.18 h
INSTRUM spect
PROBHD Z148658_0003 (
PULPROG zg30
TD 65536
SOLVENT C6D6
NS 20
DS 2
SWH 12019.230 Hz
FIDRES 0.366798 Hz
AQ 2.7262976 sec
RG 120.5
DW 41.600 usec
DE 6.50 usec
TE 296.5 K
D1 2.00000000 sec
TD0 1
SFO1 600.0087050 MHz
NUC1 1H
P1 15.50 usec
PLW1 13.23200035 W
F2 - Processing parameters
SI 65536
SF 600.0049673 MHz
WDW EM
SSB 0
LB 0.30 Hz
GB 0
PC 1.00


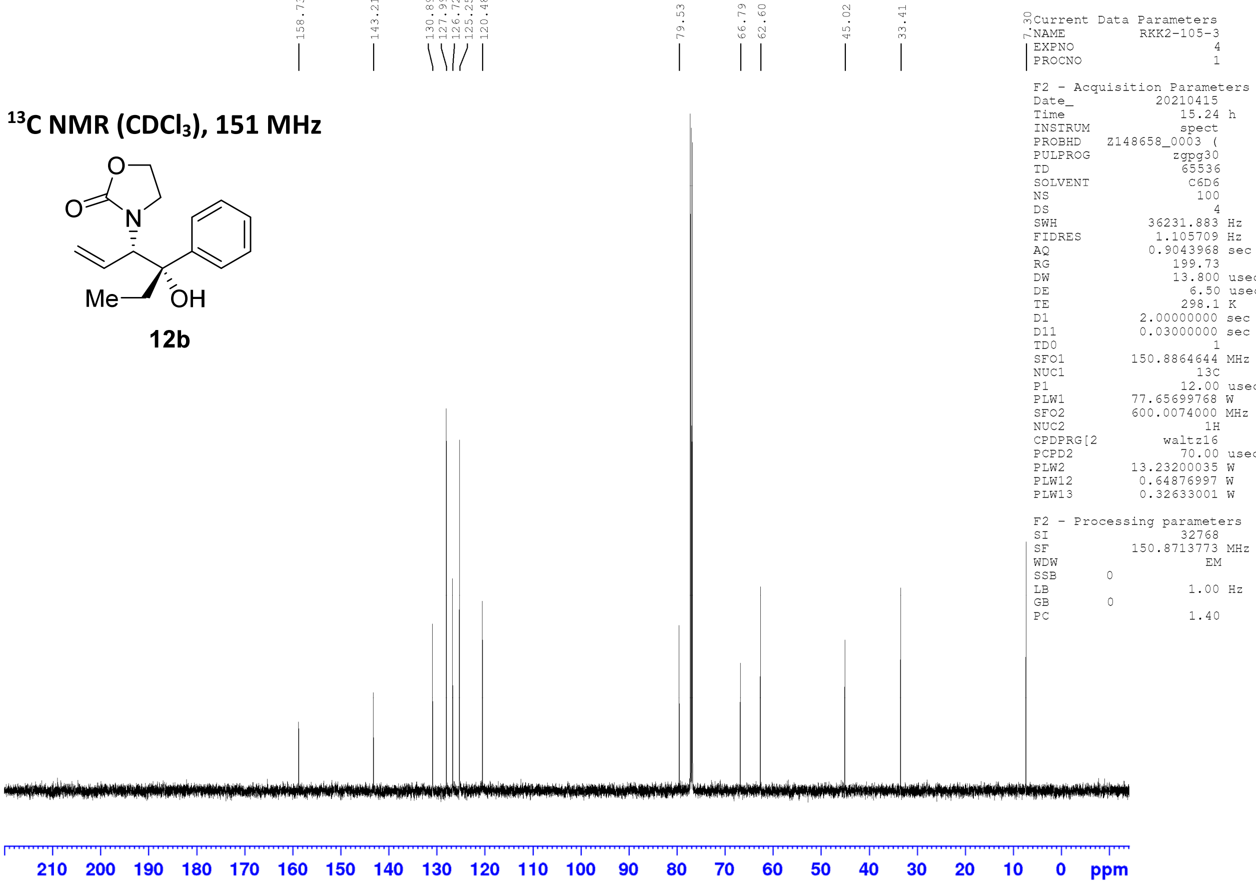

158.73 143.21 130.89 127.99 126.72 125.25 120.48 79.53 66.79 62.60 45.02 33.41 7.30
13C NMR (CDCl3), 151 MHz
O
N
O
Me
OH
12b
210 200 190 180 170 160 150 140 130 120 110 100 90 80 70 60 50 40 30 20 10 0 ppm
Current Data Parameters
NAME RKK2-105-3
EXPNO 4
PROCNO 1
F2 - Acquisition Parameters
Date_ 20210415
Time 15.24 h
INSTRUM spect
PROBHD Z148658_0003 (
PULPROG zgpg30
TD 65536
SOLVENT C6D6
NS 100
DS 4
SWH 36231.883 Hz
FIDRES 1.105709 Hz
AQ 0.9043968 sec
RG 199.73
DW 13.800 usec
DE 6.50 usec
TE 298.1 K
D1 2.00000000 sec
D11 0.03000000 sec
TD0 1
SFO1 150.8864644 MHz
NUC1 13C
P1 12.00 usec
PLW1 77.65699768 W
SFO2 600.0074000 MHz
NUC2 1H
CPDPRG[2 waltz16
PCPD2 70.00 usec
PLW2 13.23200035 W
PLW12 0.64876997 W
PLW13 0.32633001 W
F2 - Processing parameters
SI 32768
SF 150.8713773 MHz
WDW EM
SSB 0
LB 1.00 Hz
GB 0
PC 1.40

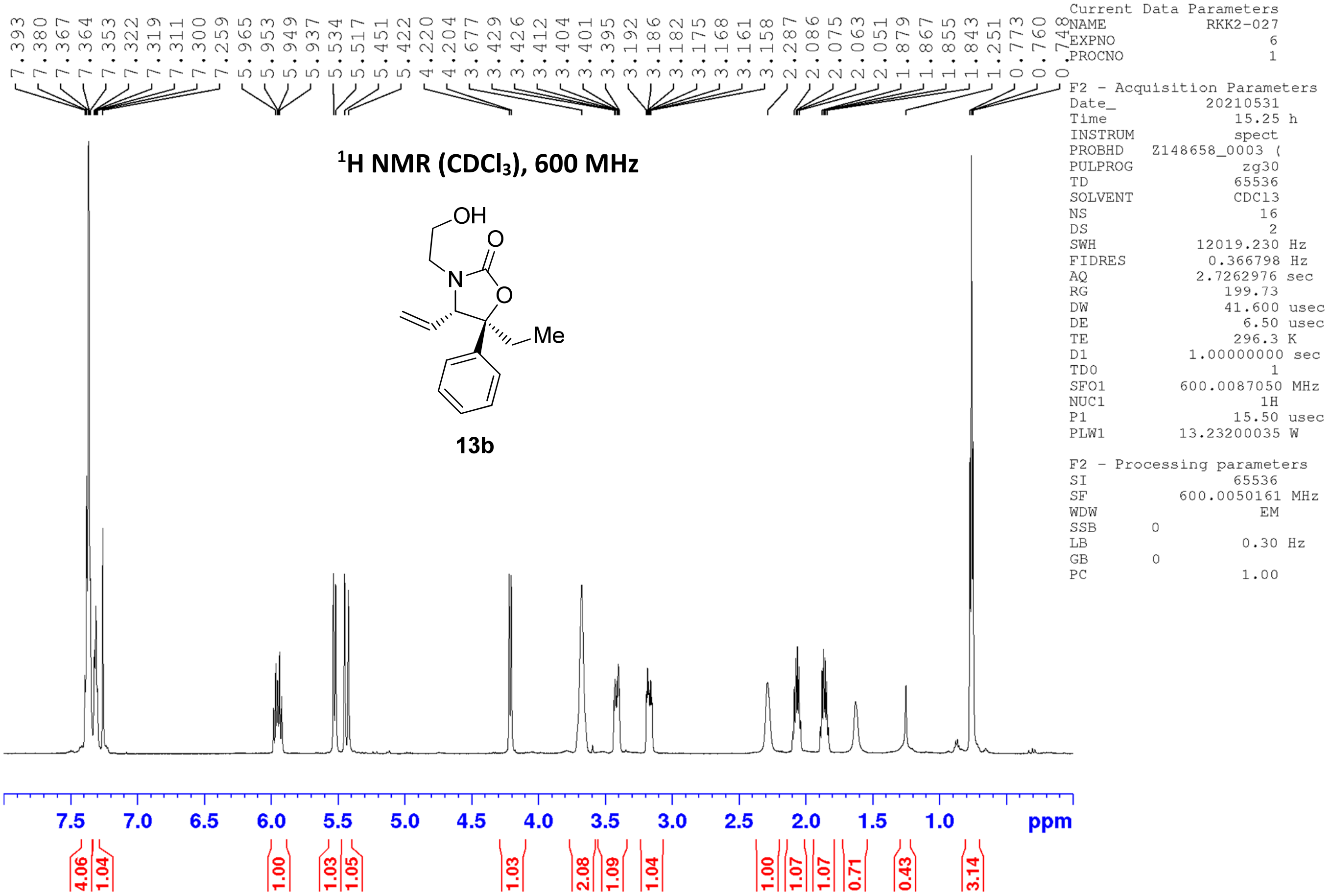

7.393 7.380 7.367 7.364 7.353 7.322 7.319 7.311 7.300 7.259 5.965 5.953 5.949 5.937 5.534 5.517 5.451 5.422 4.220 4.204 3.677 3.429 3.426 3.412 3.404 3.401 3.395 3.192 3.186 3.182 3.175 3.168 3.161 3.158 2.287 2.086 2.075 2.063 2.051 1.879 1.867 1.855 1.843 1.251 0.773 0.760 0.748
$^{1}$H NMR (CDCl$_3$), 600 MHz
OH
O
N
O
Me
13b
ppm
4.06 1.04 1.00 1.03 1.05 1.03 2.08 1.09 1.04 1.00 1.07 1.07 0.71 0.43 3.14
Current Data Parameters
NAME RKK2-027
EXPNO 6
PROCNO 1
F2 - Acquisition Parameters
Date_ 20210531
Time 15.25 h
INSTRUM spect
PROBHD Z148658_0003 (
PULPROG zg30
TD 65536
SOLVENT CDCl3
NS 16
DS 2
SWH 12019.230 Hz
FIDRES 0.366798 Hz
AQ 2.7262976 sec
RG 199.73
DW 41.600 usec
DE 6.50 usec
TE 296.3 K
D1 1.00000000 sec
TD0 1
SFO1 600.0087050 MHz
NUC1 1H
P1 15.50 usec
PLW1 13.23200035 W
F2 - Processing parameters
SI 65536
SF 600.0050161 MHz
WDW EM
SSB 0
LB 0.30 Hz
GB 0
PC 1.00


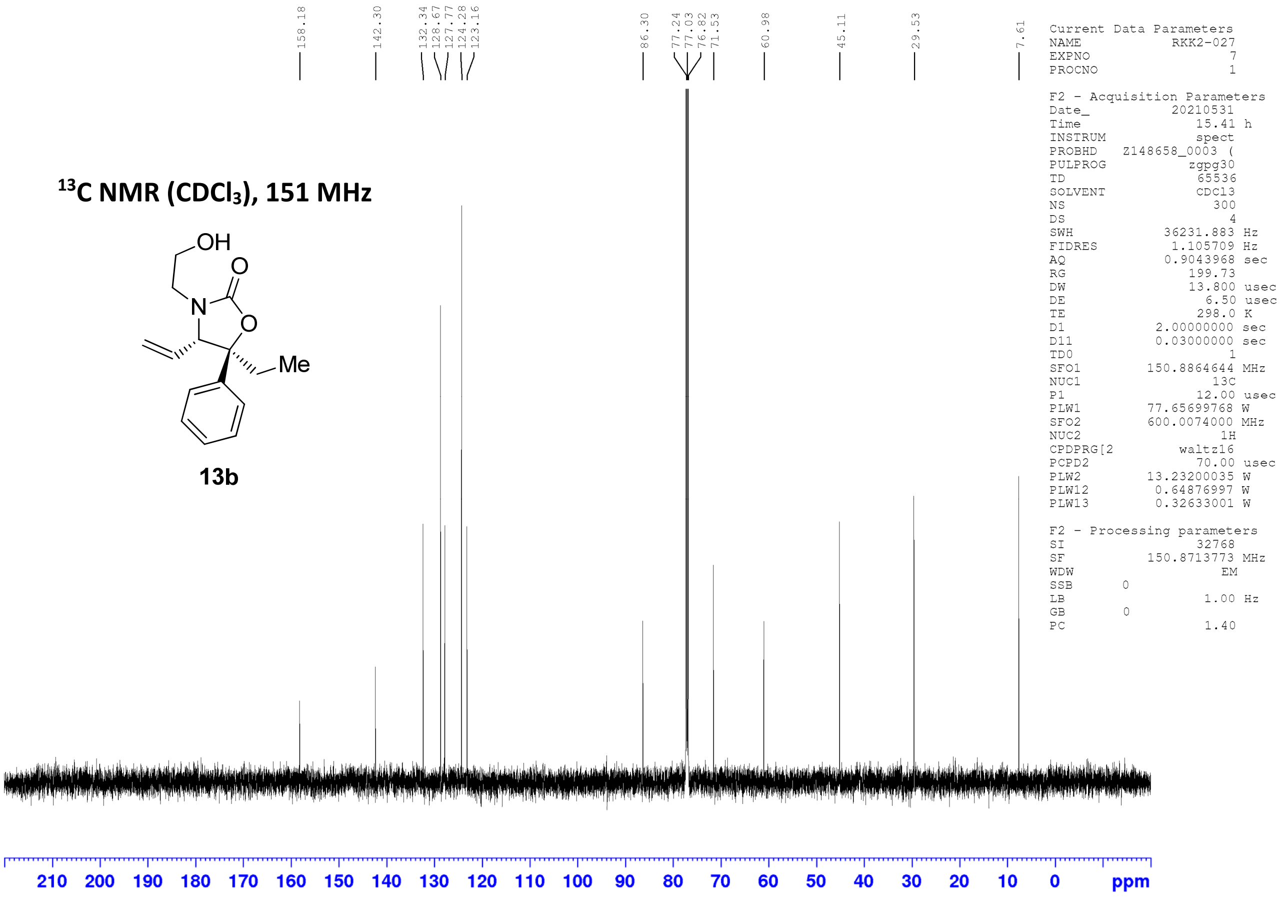

158.18 142.30 132.34 128.67 127.77 124.28 123.16 86.30 77.24 77.03 76.82 71.53 60.98 45.11 29.53 7.61
$^{13}$C NMR (CDCl$_3$), 151 MHz
OH
O
N
O
Me
13b
ppm
Current Data Parameters
NAME RKK2-027
EXPNO 7
PROCNO 1
F2 - Acquisition Parameters
Date_ 20210531
Time 15.41 h
INSTRUM spect
PROBHD Z148658_0003 (
PULPROG zgpg30
TD 65536
SOLVENT CDCl3
NS 300
DS 4
SWH 36231.883 Hz
FIDRES 1.105709 Hz
AQ 0.9043968 sec
RG 199.73
DW 13.800 usec
DE 6.50 usec
TE 298.0 K
D1 2.00000000 sec
D11 0.03000000 sec
TD0 1
SFO1 150.8864644 MHz
NUC1 13C
P1 12.00 usec
PLW1 77.65699768 W
SFO2 600.0074000 MHz
NUC2 1H
CPDPRG[2 waltz16
PCPD2 70.00 usec
PLW2 13.23200035 W
PLW12 0.64876997 W
PLW13 0.32633001 W
F2 - Processing parameters
SI 32768
SF 150.8713773 MHz
WDW EM
SSB 0
LB 1.00 Hz
GB 0
PC 1.40

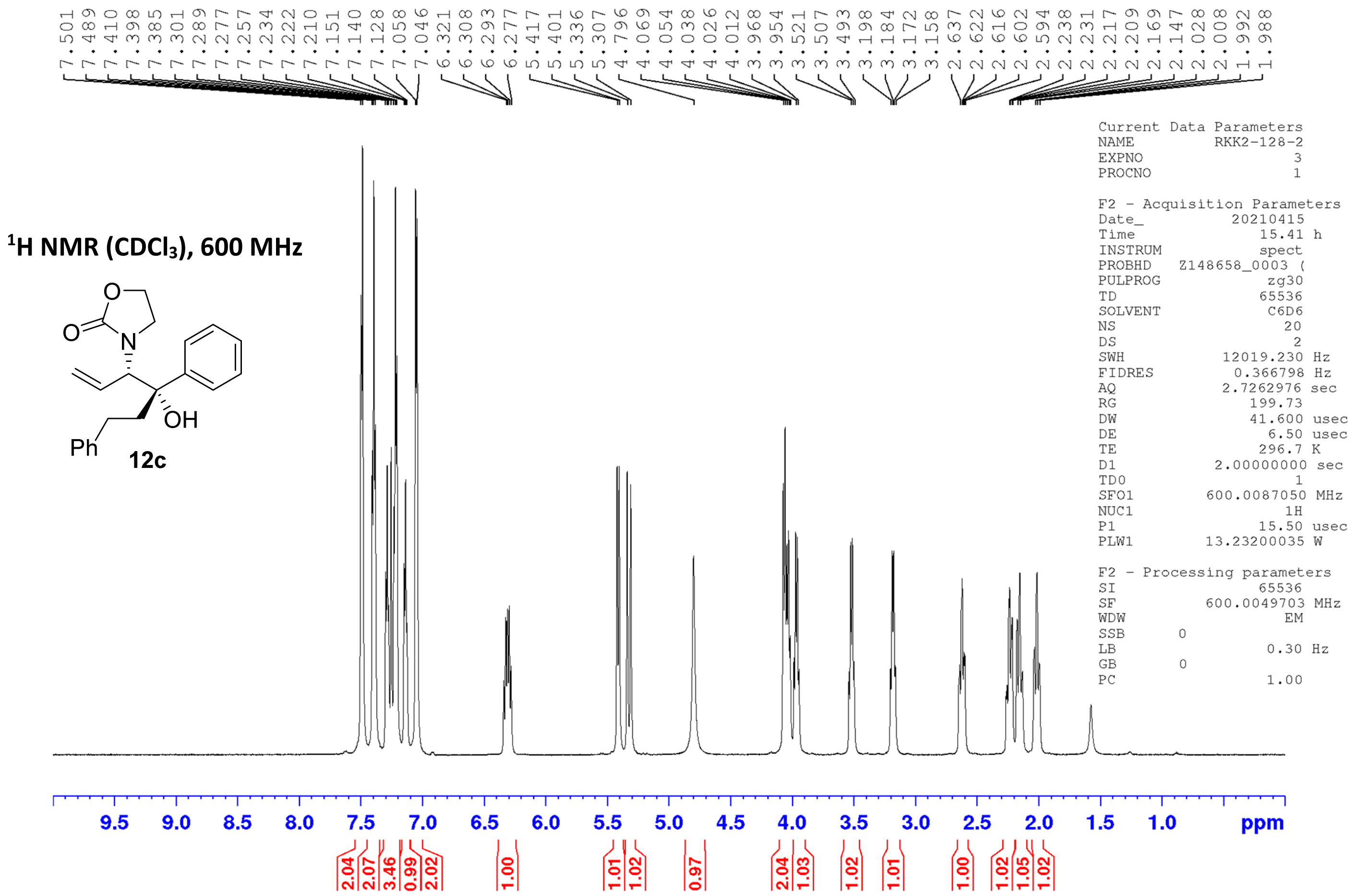

7.501 7.489 7.410 7.398 7.385 7.301 7.289 7.277 7.257 7.234 7.222 7.210 7.151 7.140 7.128 7.058 7.046 6.321 6.308 6.293 6.277 5.417 5.401 5.336 5.307 4.796 4.069 4.054 4.038 4.026 4.012 3.968 3.954 3.521 3.507 3.493 3.198 3.184 3.172 3.158 2.637 2.622 2.616 2.602 2.594 2.238 2.231 2.217 2.209 2.169 2.147 2.028 2.008 1.992 1.988
$^1$H NMR (CDCl$_3$), 600 MHz
O
O
N
OH
Ph
12c
Current Data Parameters
NAME RKK2-128-2
EXPNO 3
PROCNO 1
F2 - Acquisition Parameters
Date_ 20210415
Time 15.41 h
INSTRUM spect
PROBHD Z148658_0003 (
PULPROG zg30
TD 65536
SOLVENT C6D6
NS 20
DS 2
SWH 12019.230 Hz
FIDRES 0.366798 Hz
AQ 2.7262976 sec
RG 199.73
DW 41.600 usec
DE 6.50 usec
TE 296.7 K
D1 2.00000000 sec
TD0 1
SFO1 600.0087050 MHz
NUC1 1H
P1 15.50 usec
PLW1 13.23200035 W
F2 - Processing parameters
SI 65536
SF 600.0049703 MHz
WDW EM
SSB 0
LB 0.30 Hz
GB 0
PC 1.00
9.5 9.0 8.5 8.0 7.5 7.0 6.5 6.0 5.5 5.0 4.5 4.0 3.5 3.0 2.5 2.0 1.5 1.0 ppm
2.04 2.07 3.46 0.99 2.02 1.00 1.01 1.02 0.97 2.04 1.03 1.02 1.01 1.00 1.02 1.05 1.02


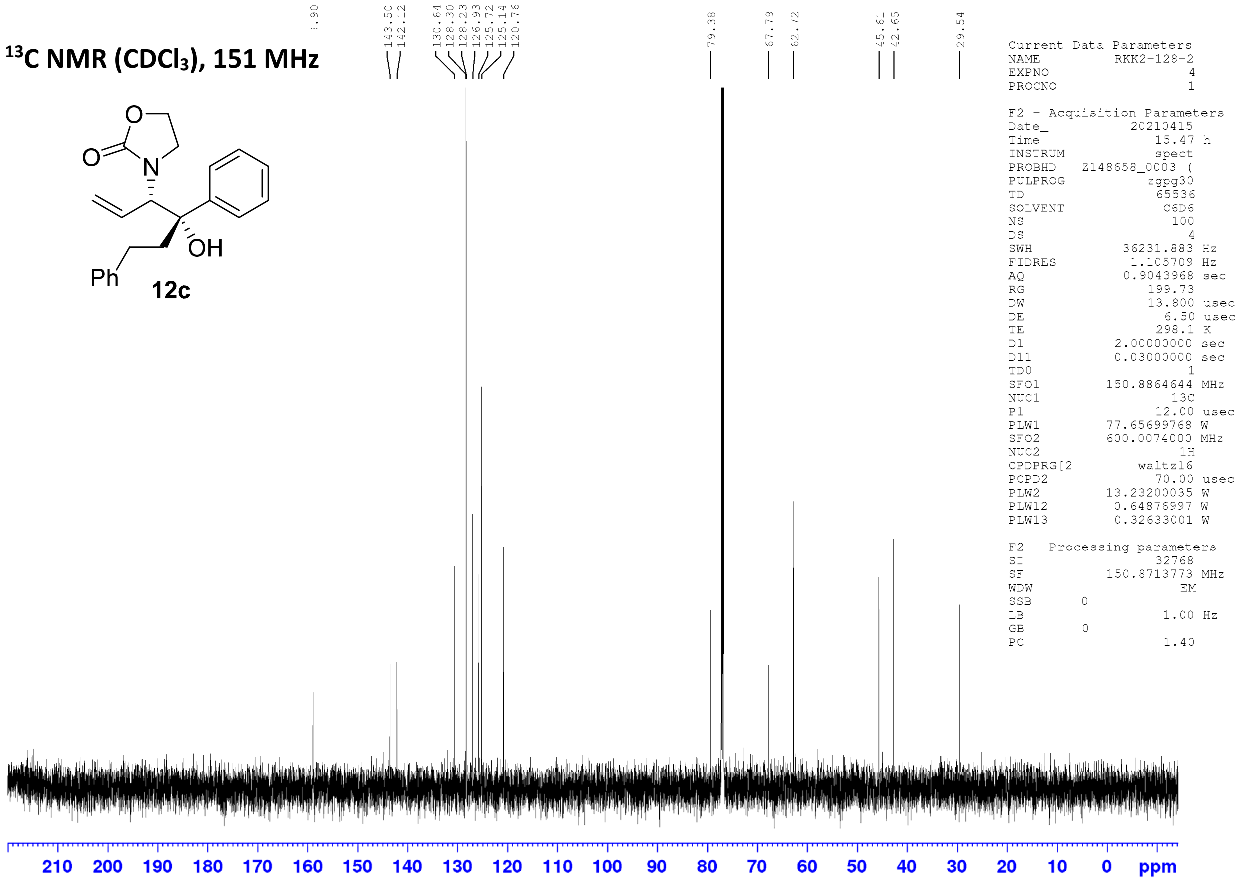

143.50 142.12 130.64 128.30 128.23 126.93 125.72 125.14 120.76 79.38 67.79 62.72 45.61 42.65 29.54
$^{13}$C NMR (CDCl$_3$), 151 MHz
O
O
N
OH
Ph
12c
Current Data Parameters
NAME RKK2-128-2
EXPNO 4
PROCNO 1
F2 - Acquisition Parameters
Date_ 20210415
Time 15.47 h
INSTRUM spect
PROBHD Z148658_0003 (
PULPROG zgpg30
TD 65536
SOLVENT C6D6
NS 100
DS 4
SWH 36231.883 Hz
FIDRES 1.105709 Hz
AQ 0.9043968 sec
RG 199.73
DW 13.800 usec
DE 6.50 usec
TE 298.1 K
D1 2.00000000 sec
D11 0.03000000 sec
TD0 1
SFO1 150.8864644 MHz
NUC1 13C
P1 12.00 usec
PLW1 77.65699768 W
SFO2 600.0074000 MHz
NUC2 1H
CPDPRG[2 waltz16
PCPD2 70.00 usec
PLW2 13.23200035 W
PLW12 0.64876997 W
PLW13 0.32633001 W
F2 - Processing parameters
SI 32768
SF 150.8713773 MHz
WDW EM
SSB 0
LB 1.00 Hz
GB 0
PC 1.40
210 200 190 180 170 160 150 140 130 120 110 100 90 80 70 60 50 40 30 20 10 0 ppm

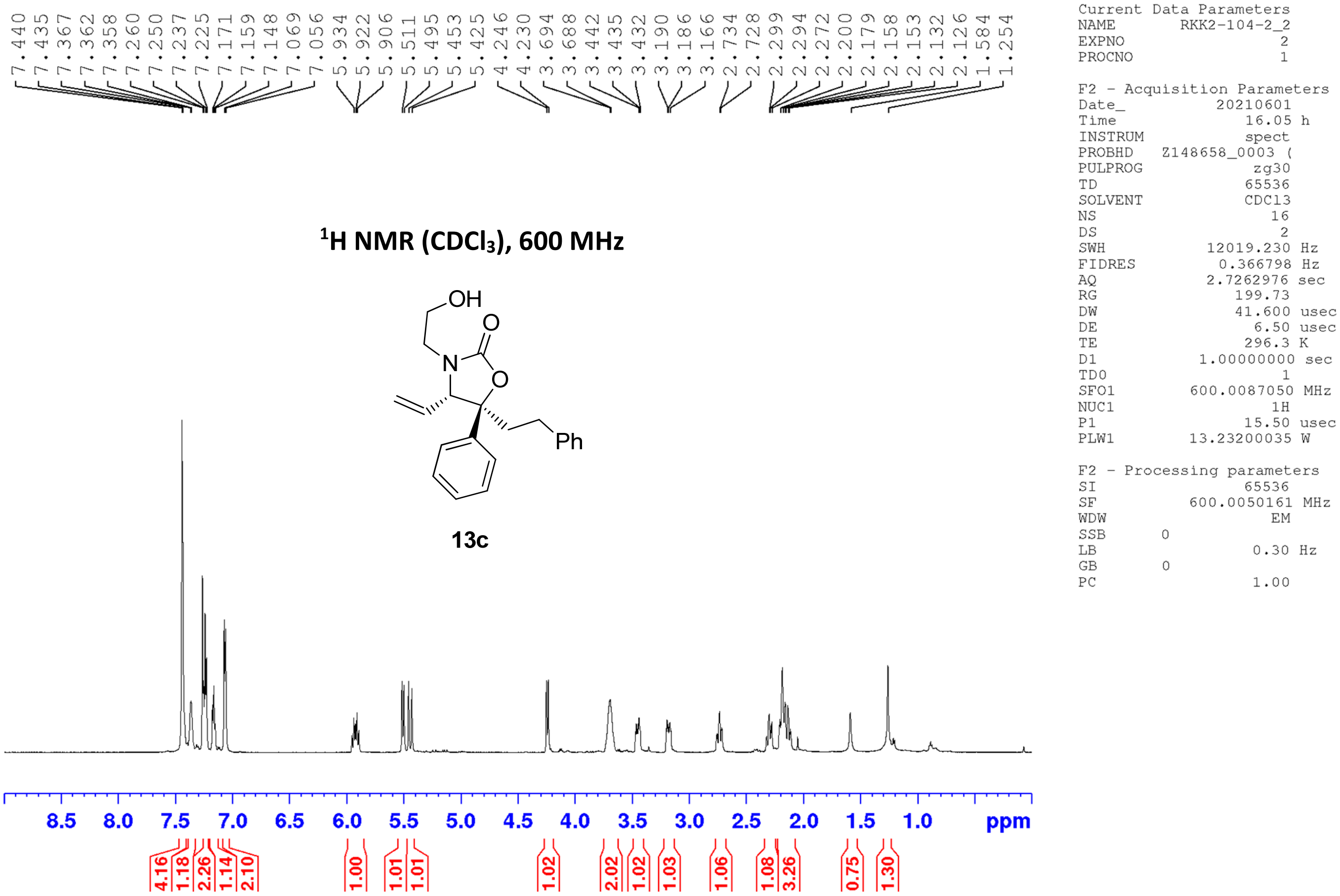
7.440
7.435
7.367
7.362
7.358
7.260
7.250
7.237
7.225
7.171
7.159
7.148
7.069
7.056
5.934
5.922
5.906
5.511
5.495
5.453
5.425
4.246
4.230
3.694
3.688
3.442
3.435
3.432
3.190
3.186
3.166
2.734
2.728
2.299
2.294
2.272
2.200
2.179
2.158
2.153
2.132
2.126
1.584
1.254
1H NMR (CDCl3), 600 MHz
OH
O
N
O
Ph
13c
8.5 8.0 7.5 7.0 6.5 6.0 5.5 5.0 4.5 4.0 3.5 3.0 2.5 2.0 1.5 1.0 ppm
4.16 1.18 2.26 1.14 2.10 1.00 1.01 1.01 1.02 2.02 1.02 1.03 1.06 1.08 3.26 0.75 1.30
Current Data Parameters
NAME RKK2-104-2_2
EXPNO 2
PROCNO 1
F2 - Acquisition Parameters
Date_ 20210601
Time 16.05 h
INSTRUM spect
PROBHD Z148658_0003 (
PULPROG zg30
TD 65536
SOLVENT CDCl3
NS 16
DS 2
SWH 12019.230 Hz
FIDRES 0.366798 Hz
AQ 2.7262976 sec
RG 199.73
DW 41.600 usec
DE 6.50 usec
TE 296.3 K
D1 1.00000000 sec
TD0 1
SFO1 600.0087050 MHz
NUC1 1H
P1 15.50 usec
PLW1 13.23200035 W
F2 - Processing parameters
SI 65536
SF 600.0050161 MHz
WDW EM
SSB 0
LB 0.30 Hz
GB 0
PC 1.00

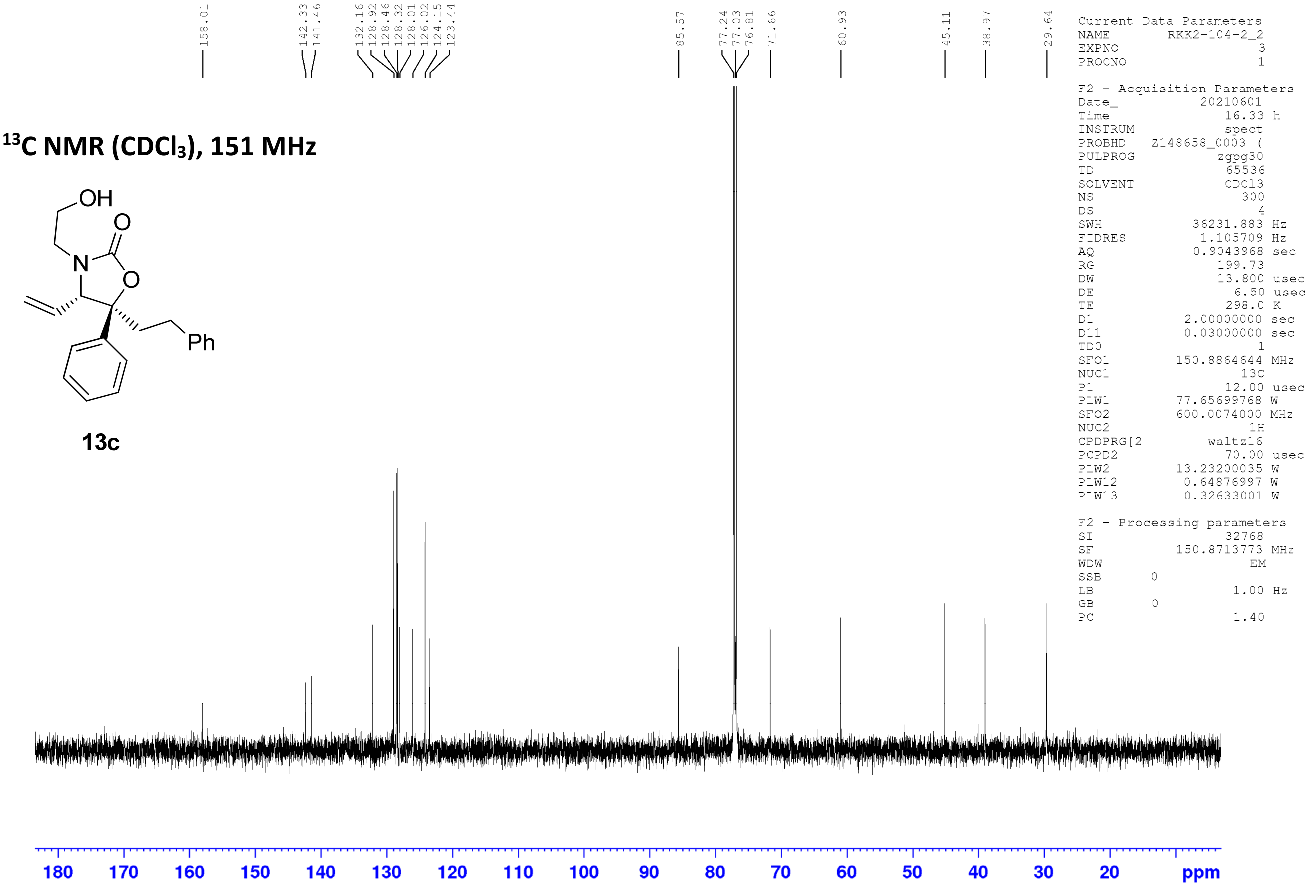
158.01
142.33
141.46
132.16
128.92
128.46
128.32
128.01
126.02
124.15
123.44
85.57
77.24
77.03
76.81
71.66
60.93
45.11
38.97
29.64
13C NMR (CDCl3), 151 MHz
OH
O
N
O
Ph
13c
180 170 160 150 140 130 120 110 100 90 80 70 60 50 40 30 20 ppm
Current Data Parameters
NAME RKK2-104-2_2
EXPNO 3
PROCNO 1
F2 - Acquisition Parameters
Date_ 20210601
Time 16.33 h
INSTRUM spect
PROBHD Z148658_0003 (
PULPROG zgpg30
TD 65536
SOLVENT CDCl3
NS 300
DS 4
SWH 36231.883 Hz
FIDRES 1.105709 Hz
AQ 0.9043968 sec
RG 199.73
DW 13.800 usec
DE 6.50 usec
TE 298.0 K
D1 2.00000000 sec
D11 0.03000000 sec
TD0 1
SFO1 150.8864644 MHz
NUC1 13C
P1 12.00 usec
PLW1 77.65699768 W
SFO2 600.0074000 MHz
NUC2 1H
CPDPRG[2 waltz16
PCPD2 70.00 usec
PLW2 13.23200035 W
PLW12 0.64876997 W
PLW13 0.32633001 W
F2 - Processing parameters
SI 32768
SF 150.8713773 MHz
WDW EM
SSB 0
LB 1.00 Hz
GB 0
PC 1.40

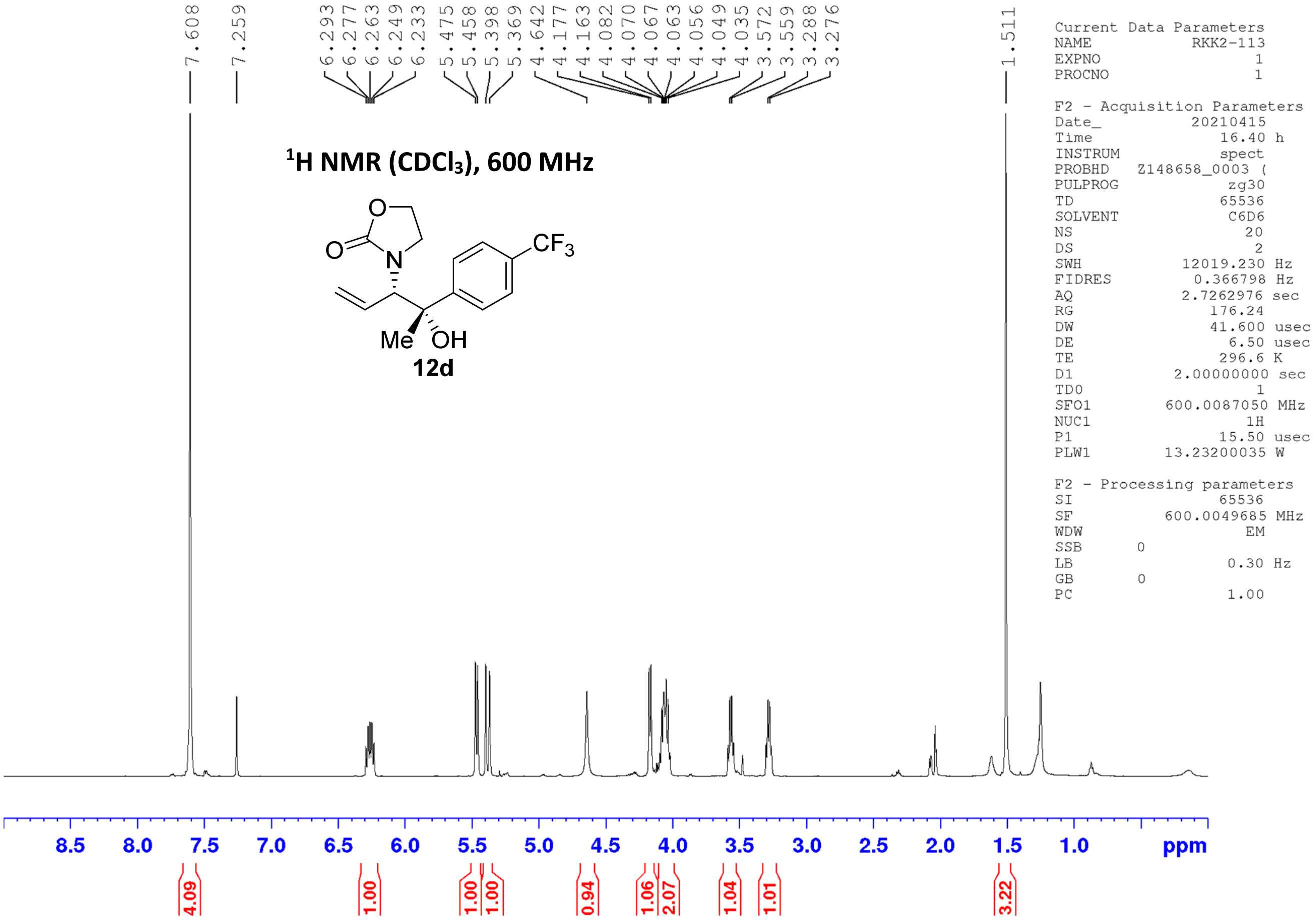
7.608
7.259
6.293
6.277
6.263
6.249
6.233
5.475
5.458
5.398
5.369
4.642
4.177
4.163
4.082
4.070
4.067
4.063
4.056
4.049
4.035
3.572
3.559
3.288
3.276
1.511
1H NMR (CDCl3), 600 MHz
O
N
O
CF3
Me
OH
12d
Current Data Parameters
NAME RKK2-113
EXPNO 1
PROCNO 1
F2 - Acquisition Parameters
Date_ 20210415
Time 16.40 h
INSTRUM spect
PROBHD Z148658_0003 (
PULPROG zg30
TD 65536
SOLVENT C6D6
NS 20
DS 2
SWH 12019.230 Hz
FIDRES 0.366798 Hz
AQ 2.7262976 sec
RG 176.24
DW 41.600 usec
DE 6.50 usec
TE 296.6 K
D1 2.00000000 sec
TD0 1
SFO1 600.0087050 MHz
NUC1 1H
P1 15.50 usec
PLW1 13.23200035 W
F2 - Processing parameters
SI 65536
SF 600.0049685 MHz
WDW EM
SSB 0
LB 0.30 Hz
GB 0
PC 1.00
8.5
8.0
7.5
7.0
6.5
6.0
5.5
5.0
4.5
4.0
3.5
3.0
2.5
2.0
1.5
1.0
ppm
4.09
1.00
1.00
1.00
0.94
1.06
2.07
1.04
1.01
3.22

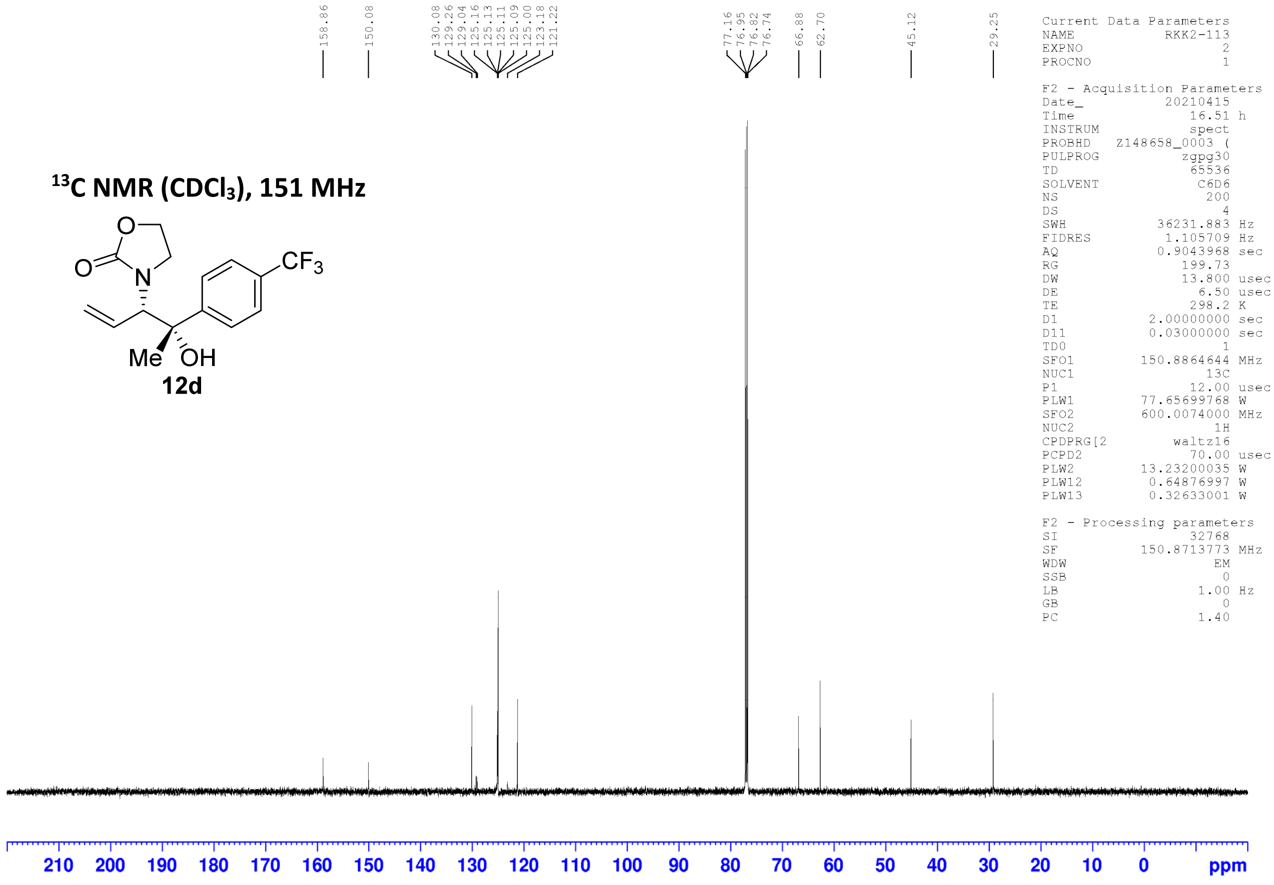
158.86
150.08
130.08
129.26
129.04
125.16
125.13
125.11
125.09
125.00
123.18
121.22
77.16
76.95
76.82
76.74
66.88
62.70
45.12
29.25
13C NMR (CDCl3), 151 MHz
O
N
O
CF3
Me
OH
12d
Current Data Parameters
NAME RKK2-113
EXPNO 2
PROCNO 1
F2 - Acquisition Parameters
Date_ 20210415
Time 16.51 h
INSTRUM spect
PROBHD Z148658_0003 (
PULPROG zgpg30
TD 65536
SOLVENT C6D6
NS 200
DS 4
SWH 36231.883 Hz
FIDRES 1.105709 Hz
AQ 0.9043968 sec
RG 199.73
DW 13.800 usec
DE 6.50 usec
TE 298.2 K
D1 2.00000000 sec
D11 0.03000000 sec
TD0 1
SFO1 150.8864644 MHz
NUC1 13C
P1 12.00 usec
PLW1 77.65699768 W
SFO2 600.0074000 MHz
NUC2 1H
CPDPRG[2 waltz16
PCPD2 70.00 usec
PLW2 13.23200035 W
PLW12 0.64876997 W
PLW13 0.32633001 W
F2 - Processing parameters
SI 32768
SF 150.8713773 MHz
WDW EM
SSB 0
LB 1.00 Hz
GB 0
PC 1.40
210
200
190
180
170
160
150
140
130
120
110
100
90
80
70
60
50
40
30
20
10
0
ppm

**$^{19}$F NMR ($CDCl_3$) 565 MHz**

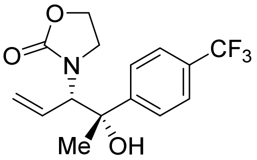

**12d**

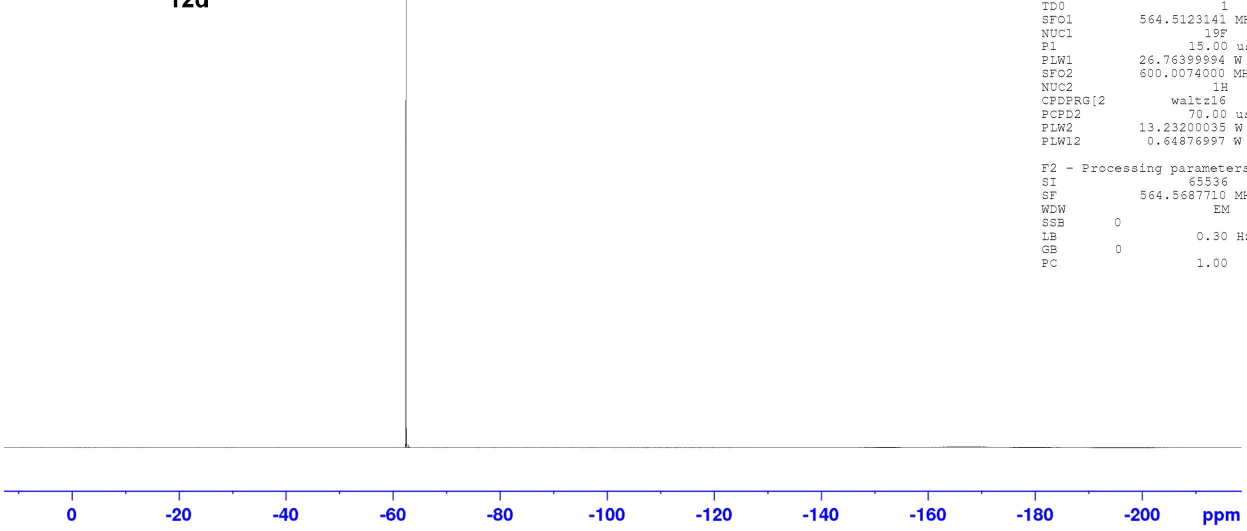


Current Data Parameters
NAME RKK2-103
EXPNO 4
PROCNO 1

F2 - Acquisition Parameters
Date_ 20210415
Time 12.11 h
INSTRUM spect
PROBHD Z148658_0003 (
PULPROG zgfhigqn.2
TD 131072
SOLVENT C6D6
NS 16
DS 4
SWH 133928.578 Hz
FIDRES 2.043588 Hz
AQ 0.4893355 sec
RG 199.73
DW 3.733 usec
DE 6.50 usec
TE 297.2 K
D1 1.00000000 sec
D11 0.03000000 sec
D12 0.00002000 sec
TD0 1
SFO1 564.5123141 MHz
NUC1 19F
P1 15.00 usec
PLW1 26.76399994 W
SFO2 600.0074000 MHz
NUC2 1H
CPDPRG[2 waltz16
PCPD2 70.00 usec
PLW2 13.23200035 W
PLW12 0.64876997 W

F2 - Processing parameters
SI 65536
SF 564.5687710 MHz
WDW EM
SSB 0
LB 0.30 Hz
GB 0
PC 1.00

**$^{1}$H NMR ($CDCl_3$), 600 MHz**

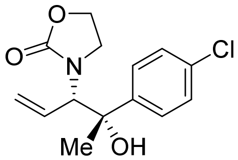

**12e**

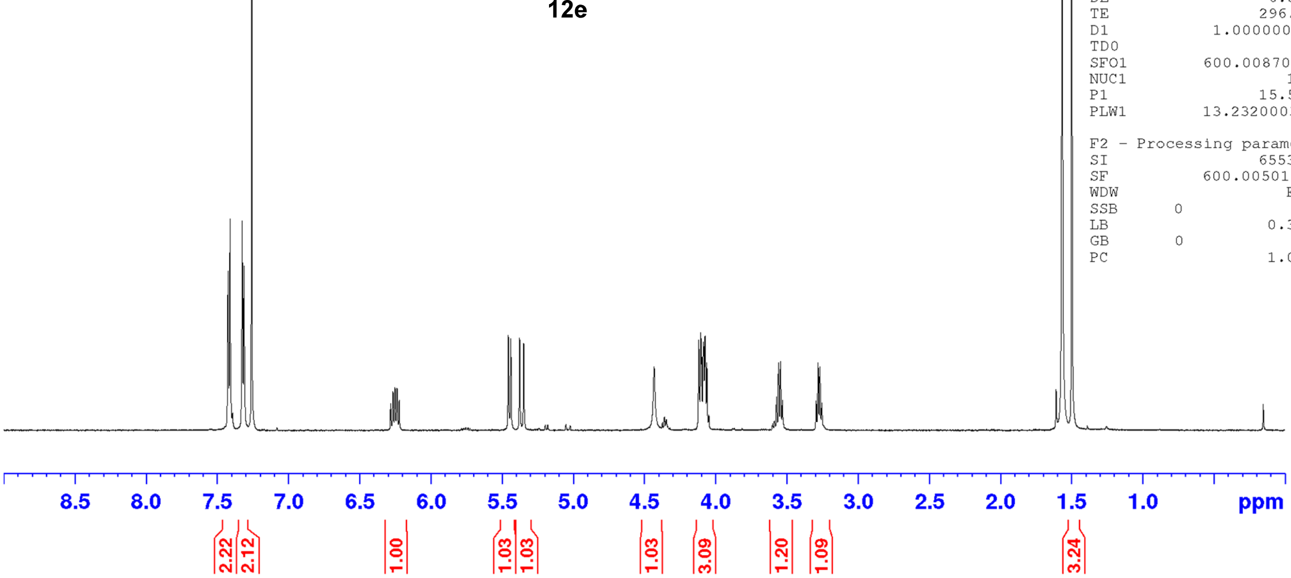


Current Data Parameters
NAME RKK2-W008
EXPNO 12
PROCNO 1

F2 - Acquisition Parameters
Date_ 20200904
Time 15.29 h
INSTRUM spect
PROBHD Z148658_0003 (
PULPROG zg30
TD 65536
SOLVENT CDCl3
NS 20
DS 2
SWH 12019.230 Hz
FIDRES 0.366798 Hz
AQ 2.7262976 sec
RG 199.73
DW 41.600 usec
DE 6.50 usec
TE 296.1 K
D1 1.00000000 sec
TD0 1
SFO1 600.0087050 MHz
NUC1 1H
P1 15.50 usec
PLW1 13.23200035 W

F2 - Processing parameters
SI 65536
SF 600.0050158 MHz
WDW EM
SSB 0
LB 0.30 Hz
GB 0
PC 1.00

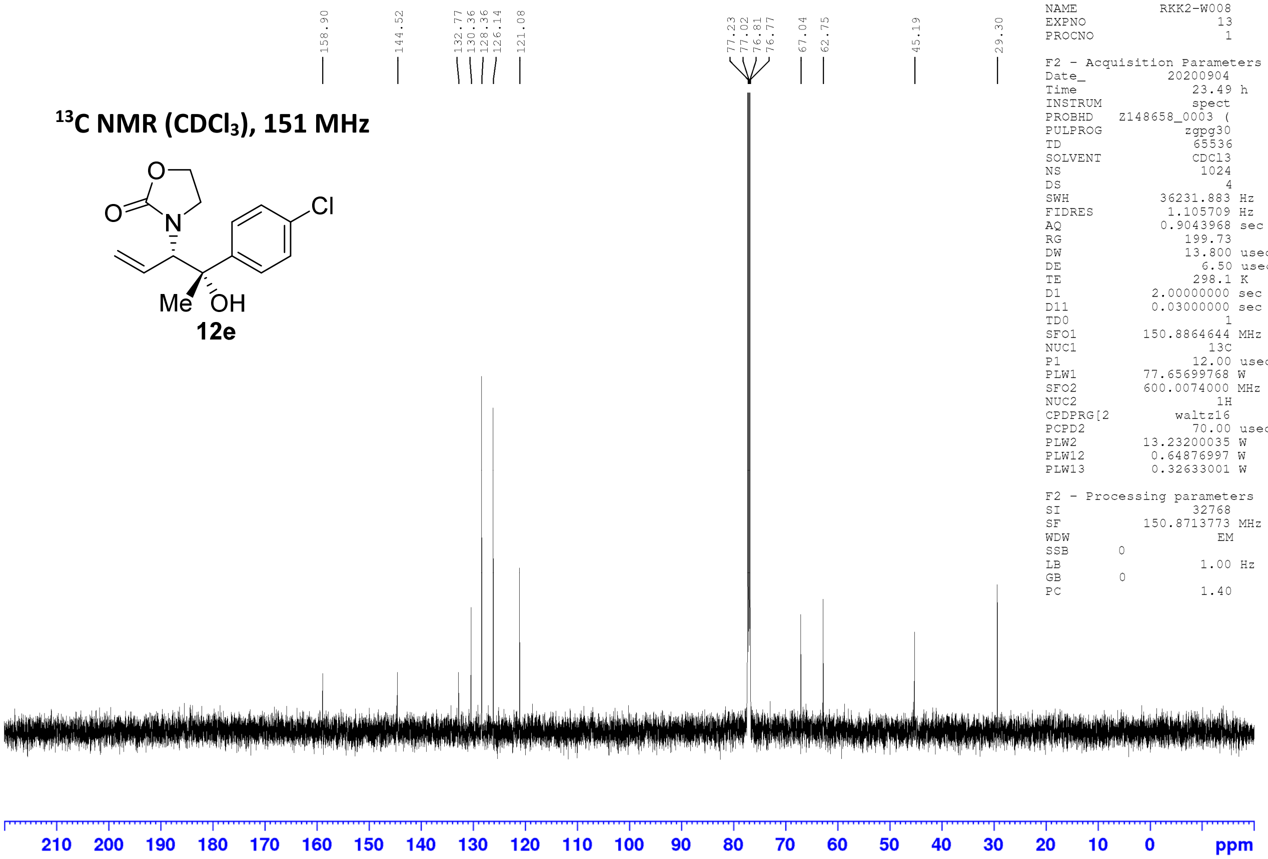


$^{13}$C NMR ($CDCl_3$), 151 MHz

**12e**

Current Data Parameters
NAME RKK2-W008
EXPNO 13
PROCNO 1

F2 - Acquisition Parameters
Date_ 20200904
Time 23.49 h
INSTRUM spect
PROBHD Z148658_0003 (
PULPROG zgpg30
TD 65536
SOLVENT CDCl3
NS 1024
DS 4
SWH 36231.883 Hz
FIDRES 1.105709 Hz
AQ 0.9043968 sec
RG 199.73
DW 13.800 usec
DE 6.50 usec
TE 298.1 K
D1 2.00000000 sec
D11 0.03000000 sec
TD0 1
SFO1 150.8864644 MHz
NUC1 13C
P1 12.00 usec
PLW1 77.65699768 W
SFO2 600.0074000 MHz
NUC2 1H
CPDPRG[2 waltz16
PCPD2 70.00 usec
PLW2 13.23200035 W
PLW12 0.64876997 W
PLW13 0.32633001 W

F2 - Processing parameters
SI 32768
SF 150.8713773 MHz
WDW EM
SSB 0
LB 1.00 Hz
GB 0
PC 1.40

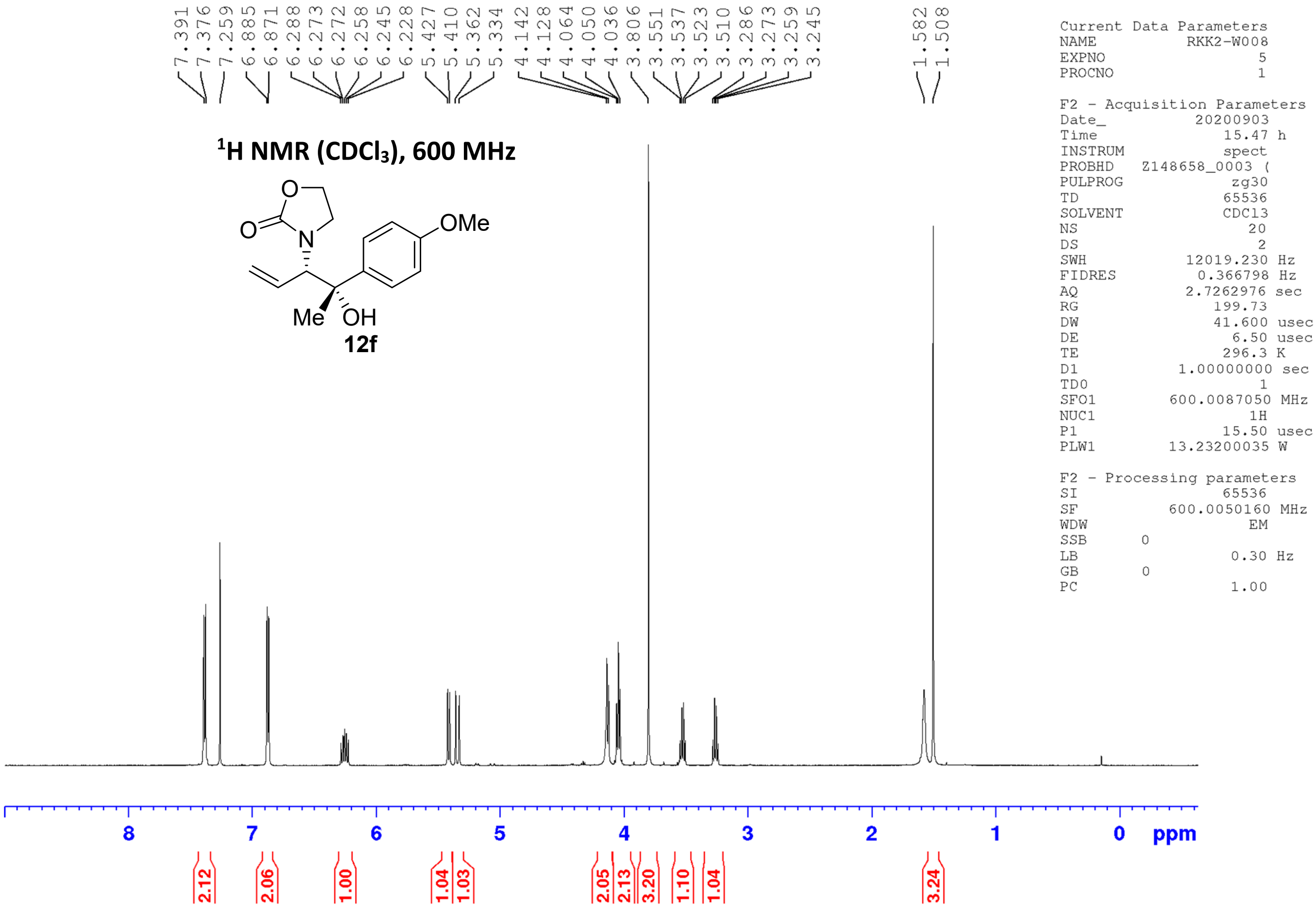


$^{1}$H NMR ($CDCl_3$), 600 MHz

**12f**

Current Data Parameters
NAME RKK2-W008
EXPNO 5
PROCNO 1

F2 - Acquisition Parameters
Date_ 20200903
Time 15.47 h
INSTRUM spect
PROBHD Z148658_0003 (
PULPROG zg30
TD 65536
SOLVENT CDCl3
NS 20
DS 2
SWH 12019.230 Hz
FIDRES 0.366798 Hz
AQ 2.7262976 sec
RG 199.73
DW 41.600 usec
DE 6.50 usec
TE 296.3 K
D1 1.00000000 sec
TD0 1
SFO1 600.0087050 MHz
NUC1 1H
P1 15.50 usec
PLW1 13.23200035 W

F2 - Processing parameters
SI 65536
SF 600.0050160 MHz
WDW EM
SSB 0
LB 0.30 Hz
GB 0
PC 1.00

$^{13}$C NMR (CDCl$_3$), 151 MHz

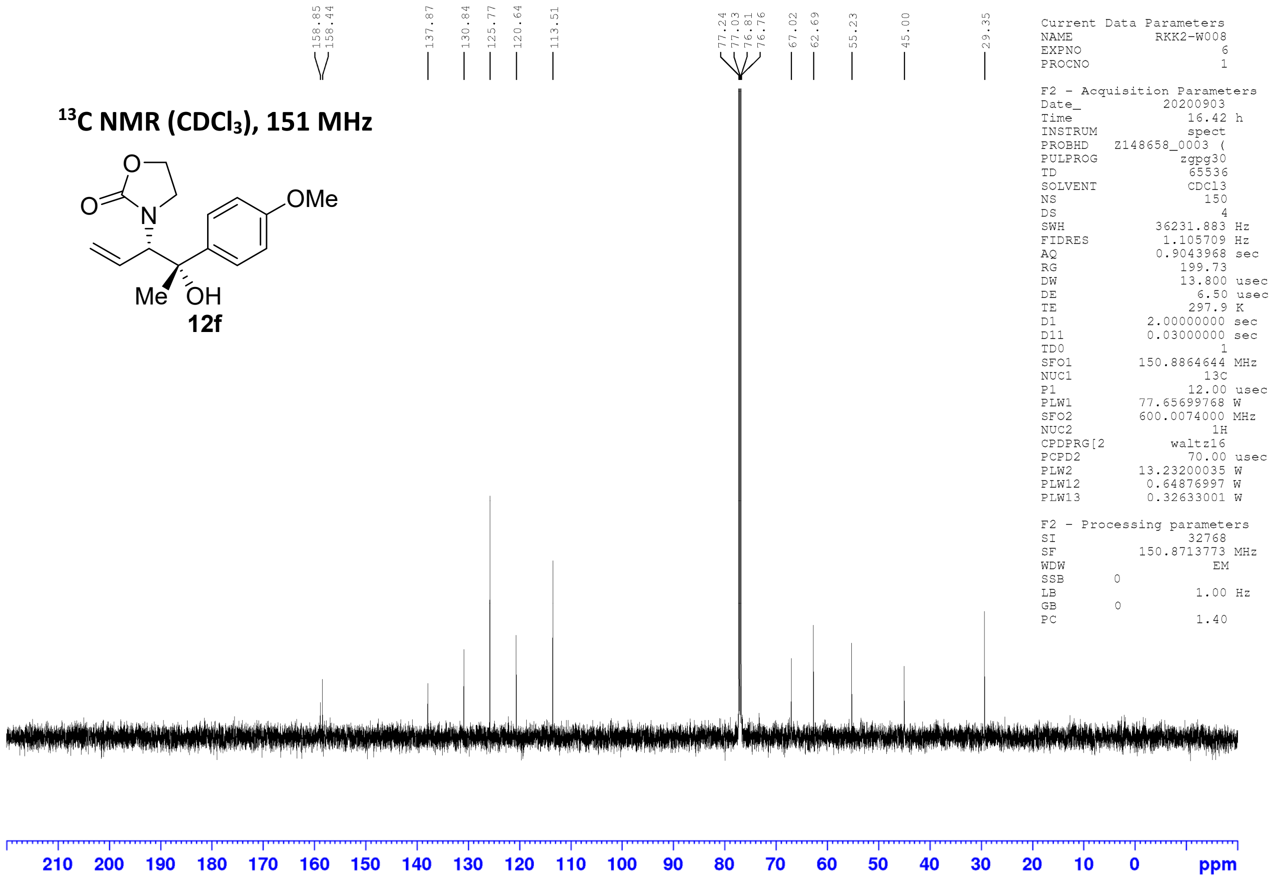


**12f**

$^{1}$H NMR (CDCl$_3$), 600 MHz

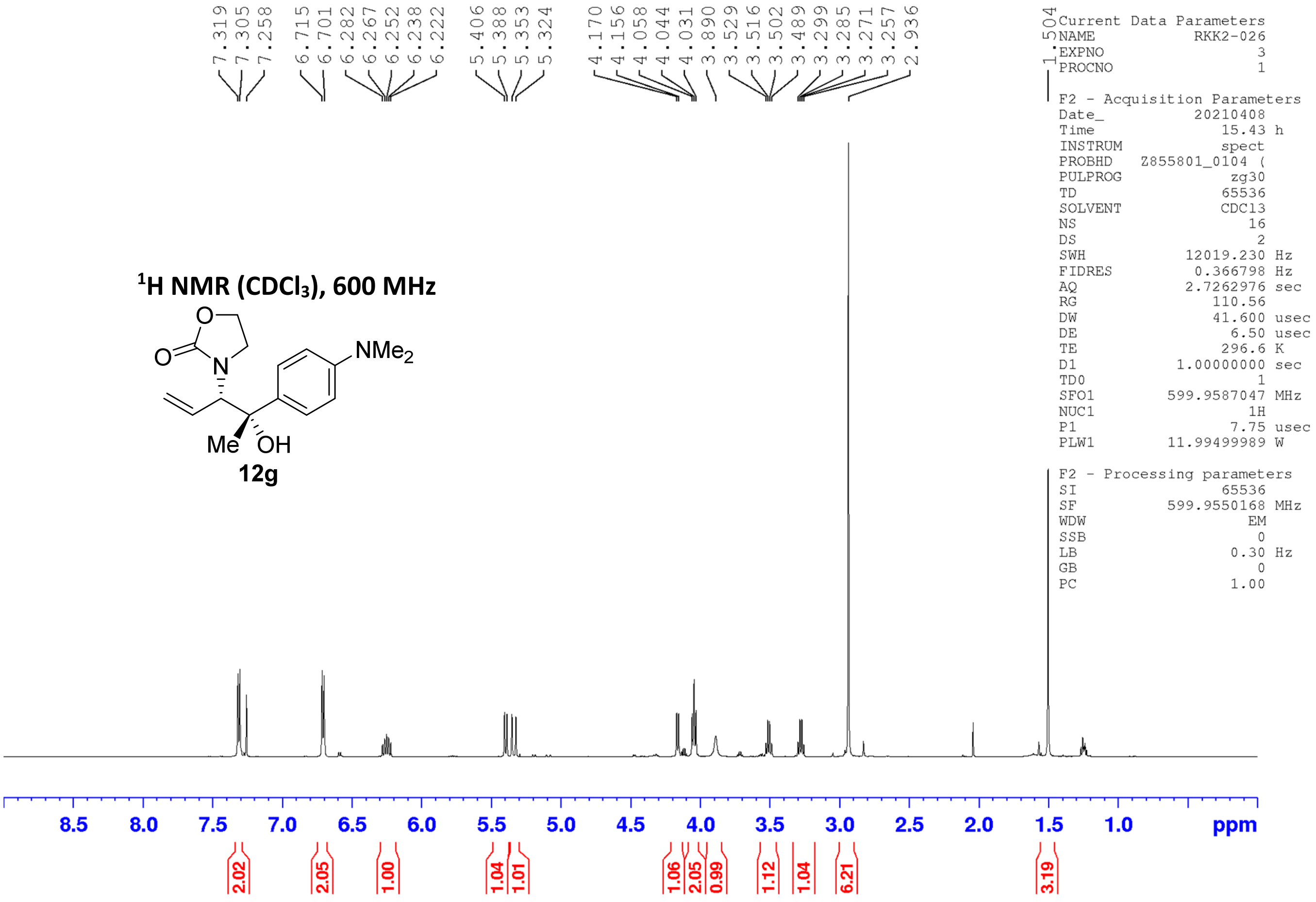


**12g**

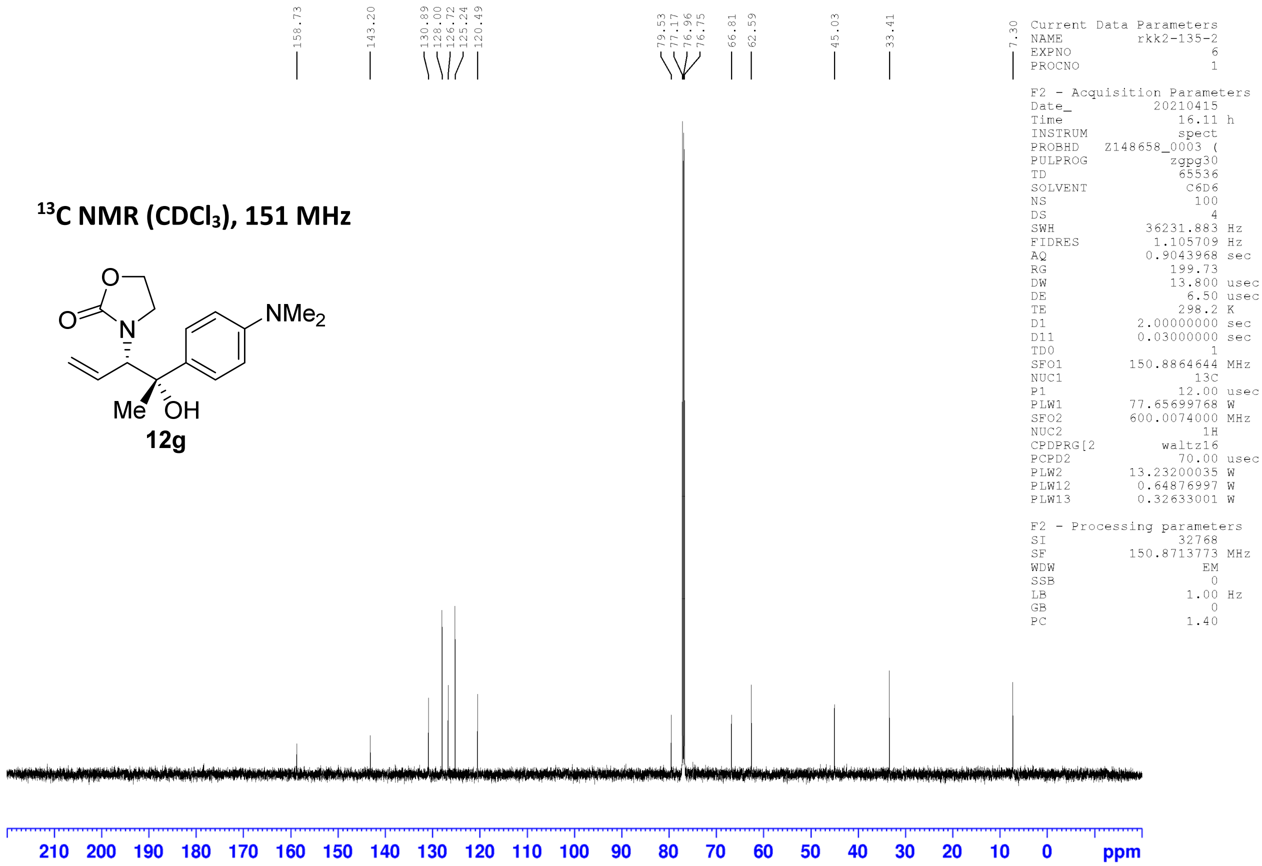

158.73
143.20
130.89
128.00
126.72
125.24
120.49
79.53
77.17
76.96
76.75
66.81
62.59
45.03
33.41
7.30
Current Data Parameters
NAME rkk2-135-2
EXPNO 6
PROCNO 1
F2 - Acquisition Parameters
Date_ 20210415
Time 16.11 h
INSTRUM spect
PROBHD Z148658_0003 (
PULPROG zgpg30
TD 65536
SOLVENT C6D6
NS 100
DS 4
SWH 36231.883 Hz
FIDRES 1.105709 Hz
AQ 0.9043968 sec
RG 199.73
DW 13.800 usec
DE 6.50 usec
TE 298.2 K
D1 2.00000000 sec
D11 0.03000000 sec
TD0 1
SFO1 150.8864644 MHz
NUC1 13C
P1 12.00 usec
PLW1 77.65699768 W
SFO2 600.0074000 MHz
NUC2 1H
CPDPRG[2 waltz16
PCPD2 70.00 usec
PLW2 13.23200035 W
PLW12 0.64876997 W
PLW13 0.32633001 W
F2 - Processing parameters
SI 32768
SF 150.8713773 MHz
WDW EM
SSB 0
LB 1.00 Hz
GB 0
PC 1.40
13C NMR (CDCl3), 151 MHz
O
O
N
NMe2
Me
OH
12g
210 200 190 180 170 160 150 140 130 120 110 100 90 80 70 60 50 40 30 20 10 0 ppm


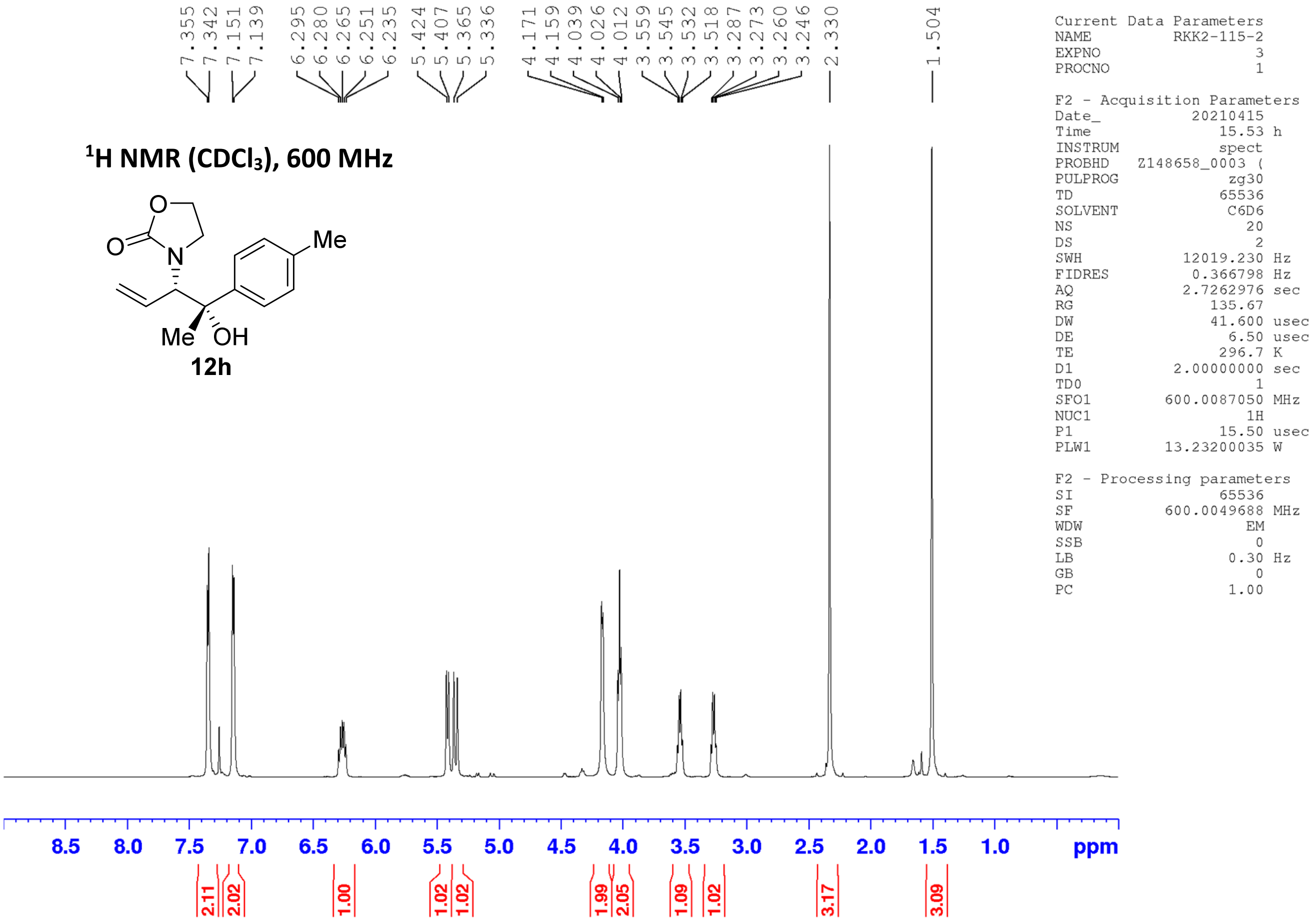

7.355
7.342
7.151
7.139
6.295
6.280
6.265
6.251
6.235
5.424
5.407
5.365
5.336
4.171
4.159
4.039
4.026
4.012
3.559
3.545
3.532
3.518
3.287
3.273
3.260
3.246
2.330
1.504
Current Data Parameters
NAME RKK2-115-2
EXPNO 3
PROCNO 1
F2 - Acquisition Parameters
Date_ 20210415
Time 15.53 h
INSTRUM spect
PROBHD Z148658_0003 (
PULPROG zg30
TD 65536
SOLVENT C6D6
NS 20
DS 2
SWH 12019.230 Hz
FIDRES 0.366798 Hz
AQ 2.7262976 sec
RG 135.67
DW 41.600 usec
DE 6.50 usec
TE 296.7 K
D1 2.00000000 sec
TD0 1
SFO1 600.0087050 MHz
NUC1 1H
P1 15.50 usec
PLW1 13.23200035 W
F2 - Processing parameters
SI 65536
SF 600.0049688 MHz
WDW EM
SSB 0
LB 0.30 Hz
GB 0
PC 1.00
1H NMR (CDCl3), 600 MHz
O
O
N
Me
Me
OH
12h
8.5 8.0 7.5 7.0 6.5 6.0 5.5 5.0 4.5 4.0 3.5 3.0 2.5 2.0 1.5 1.0 ppm
2.11
2.02
1.00
1.02
1.02
1.99
2.05
1.09
1.02
3.17
3.09

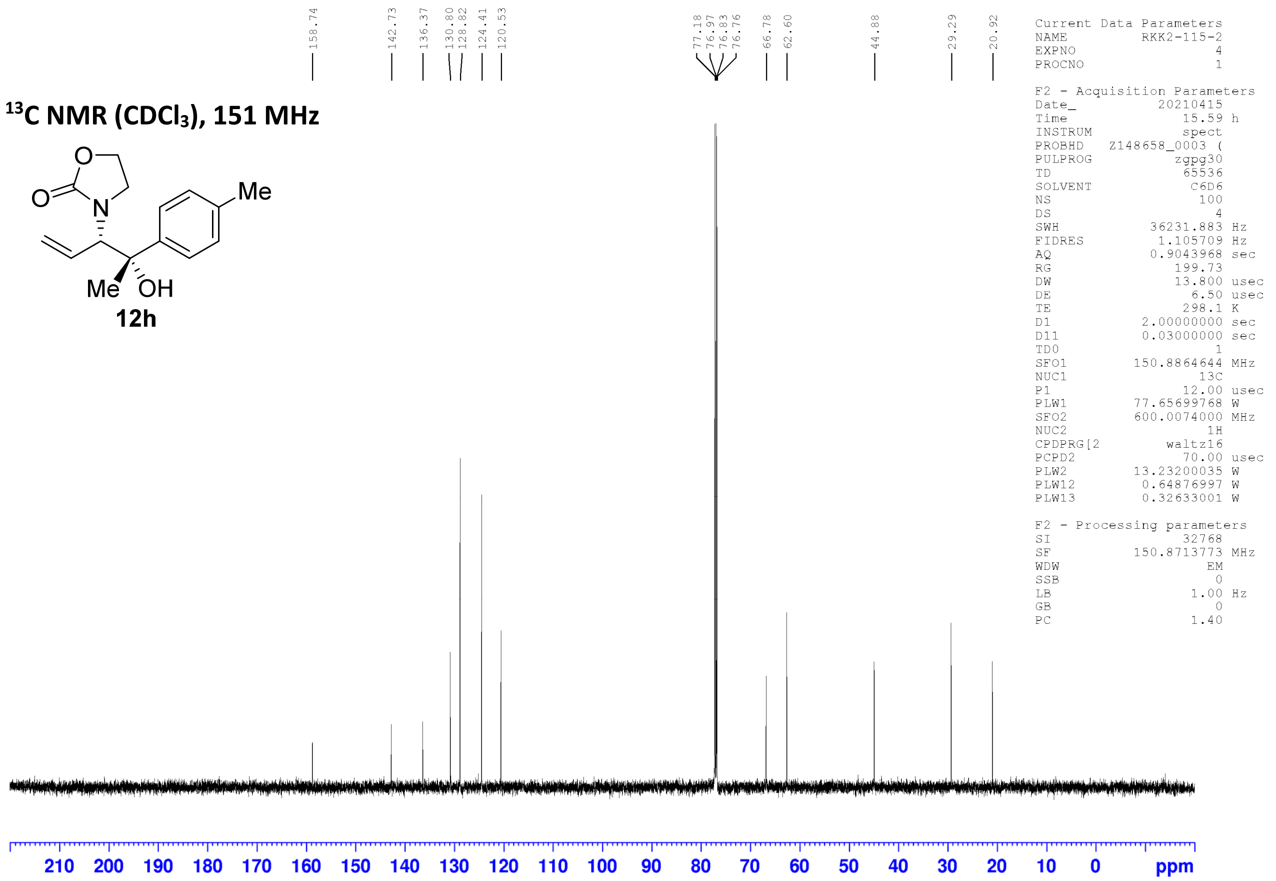
158.74
142.73
136.37
130.80
128.82
124.41
120.53
77.18
76.97
76.83
76.76
66.78
62.60
44.88
29.29
20.92
$^{13}$C NMR (CDCl$_3$), 151 MHz
O
N
O
Me
Me
OH
12h
Current Data Parameters
NAME RKK2-115-2
EXPNO 4
PROCNO 1
F2 - Acquisition Parameters
Date_ 20210415
Time 15.59 h
INSTRUM spect
PROBHD Z148658_0003 (
PULPROG zgpg30
TD 65536
SOLVENT C6D6
NS 100
DS 4
SWH 36231.883 Hz
FIDRES 1.105709 Hz
AQ 0.9043968 sec
RG 199.73
DW 13.800 usec
DE 6.50 usec
TE 298.1 K
D1 2.00000000 sec
D11 0.03000000 sec
TD0 1
SFO1 150.8864644 MHz
NUC1 13C
P1 12.00 usec
PLW1 77.65699768 W
SFO2 600.0074000 MHz
NUC2 1H
CPDPRG[2 waltz16
PCPD2 70.00 usec
PLW2 13.23200035 W
PLW12 0.64876997 W
PLW13 0.32633001 W
F2 - Processing parameters
SI 32768
SF 150.8713773 MHz
WDW EM
SSB 0
LB 1.00 Hz
GB 0
PC 1.40
210 200 190 180 170 160 150 140 130 120 110 100 90 80 70 60 50 40 30 20 10 0 ppm

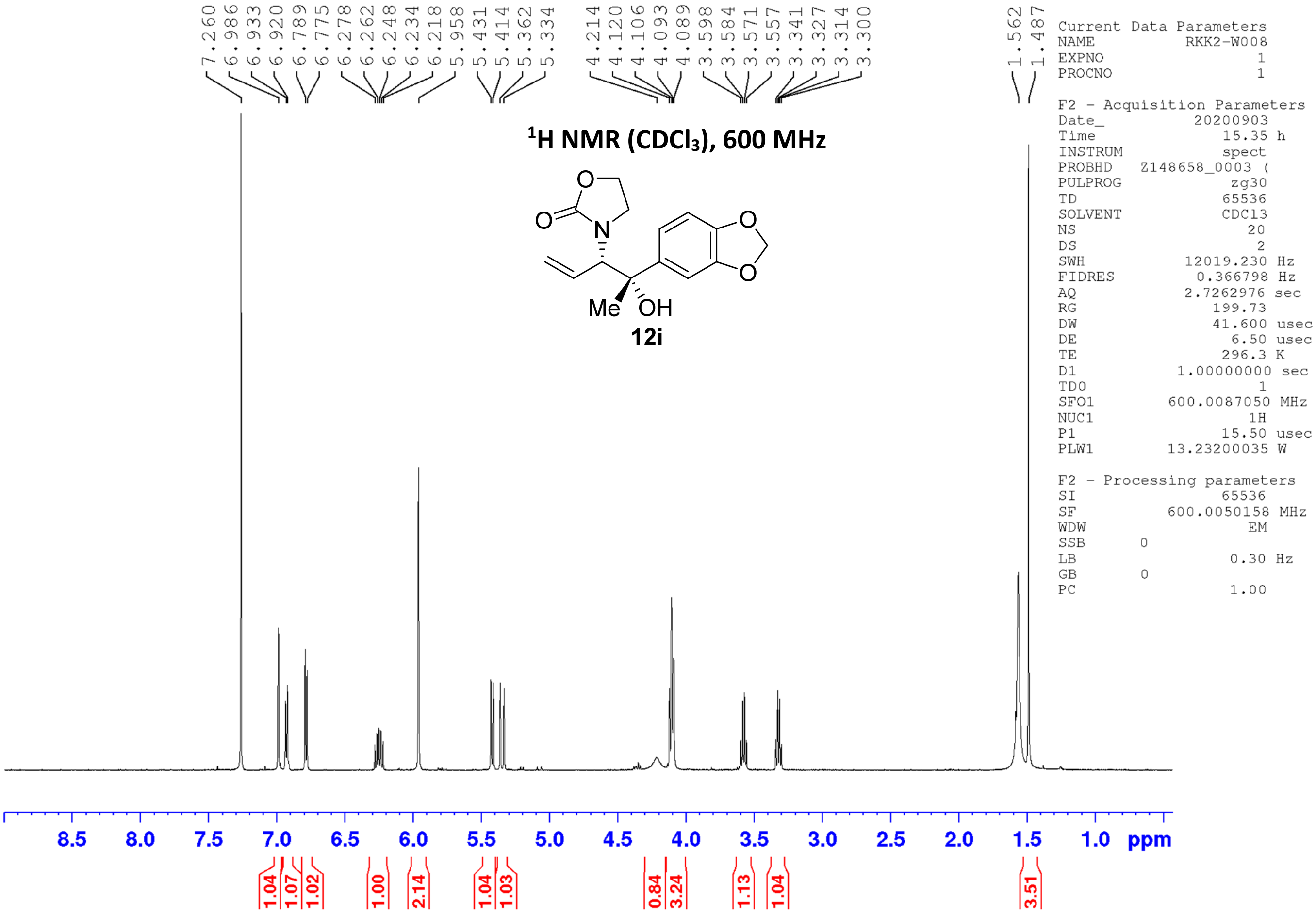
7.260
6.986
6.933
6.920
6.789
6.775
6.278
6.262
6.248
6.234
6.218
5.958
5.431
5.414
5.362
5.334
4.214
4.120
4.106
4.093
4.089
3.598
3.584
3.571
3.557
3.341
3.327
3.314
3.300
1.562
1.487
$^{1}$H NMR (CDCl$_3$), 600 MHz
O
N
O
O
O
Me
OH
12i
Current Data Parameters
NAME RKK2-W008
EXPNO 1
PROCNO 1
F2 - Acquisition Parameters
Date_ 20200903
Time 15.35 h
INSTRUM spect
PROBHD Z148658_0003 (
PULPROG zg30
TD 65536
SOLVENT CDCl3
NS 20
DS 2
SWH 12019.230 Hz
FIDRES 0.366798 Hz
AQ 2.7262976 sec
RG 199.73
DW 41.600 usec
DE 6.50 usec
TE 296.3 K
D1 1.00000000 sec
TD0 1
SFO1 600.0087050 MHz
NUC1 1H
P1 15.50 usec
PLW1 13.23200035 W
F2 - Processing parameters
SI 65536
SF 600.0050158 MHz
WDW EM
SSB 0
LB 0.30 Hz
GB 0
PC 1.00
8.5 8.0 7.5 7.0 6.5 6.0 5.5 5.0 4.5 4.0 3.5 3.0 2.5 2.0 1.5 1.0 ppm
1.04
1.07
1.02
1.00
2.14
1.04
1.03
0.84
3.24
1.13
1.04
3.51

**$^{13}C$ NMR ($CDCl_3$), 151 MHz**

**12i**

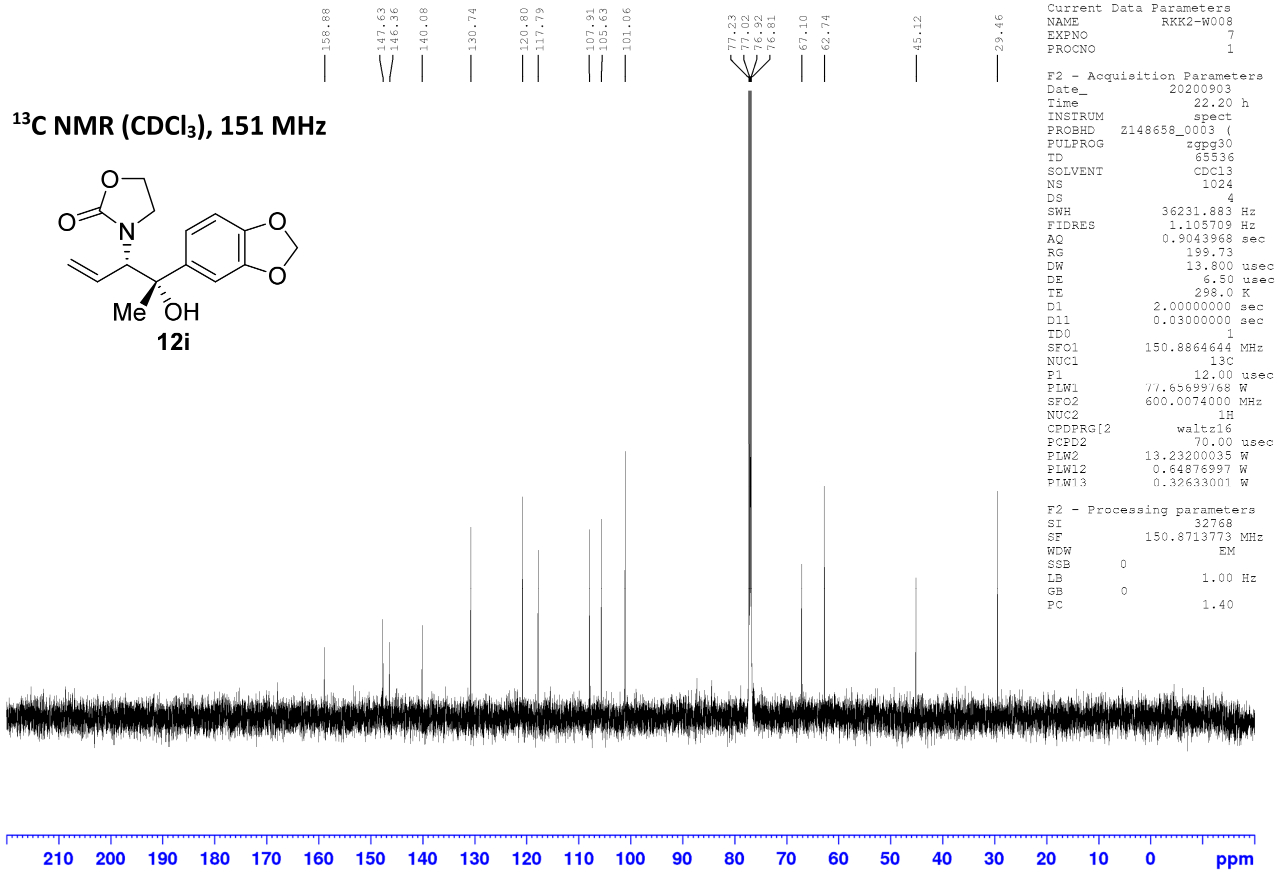


**$^{1}H$ NMR ($CDCl_3$), 600 MHz**

**12j**

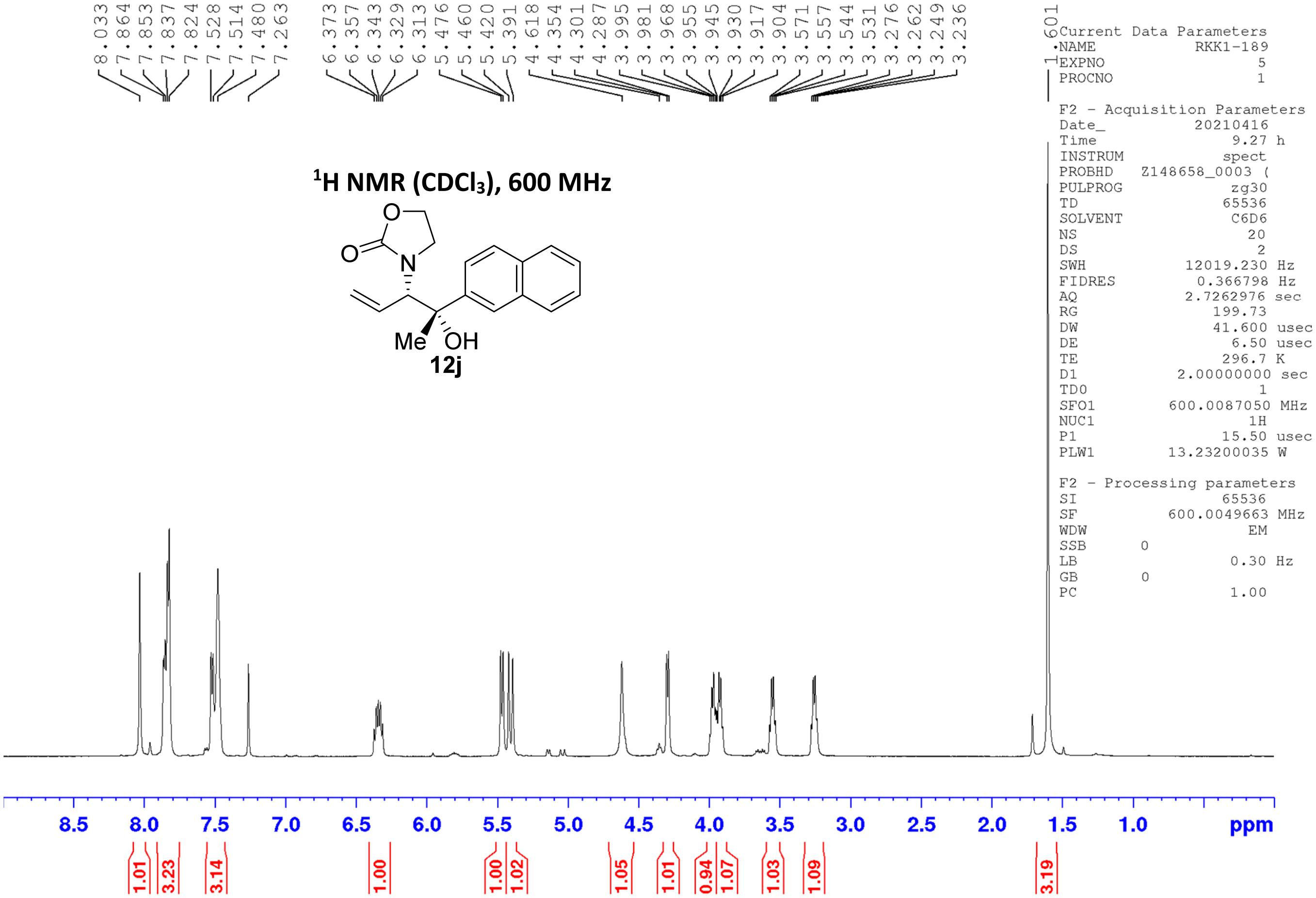

## $^{13}C$ NMR ($CDCl_3$), 151 MHz

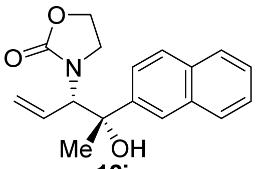

12j

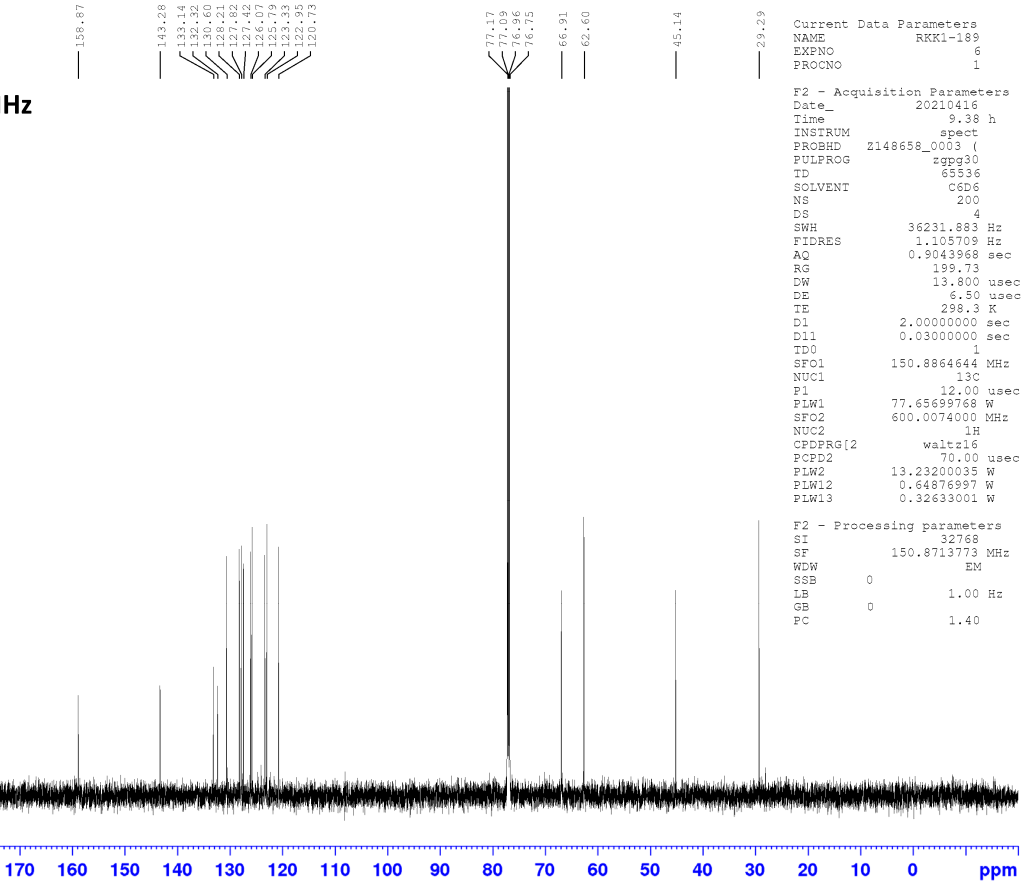


## $^{1}H$ NMR ($CDCl_3$), 600 MHz

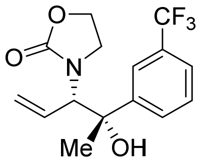

12k

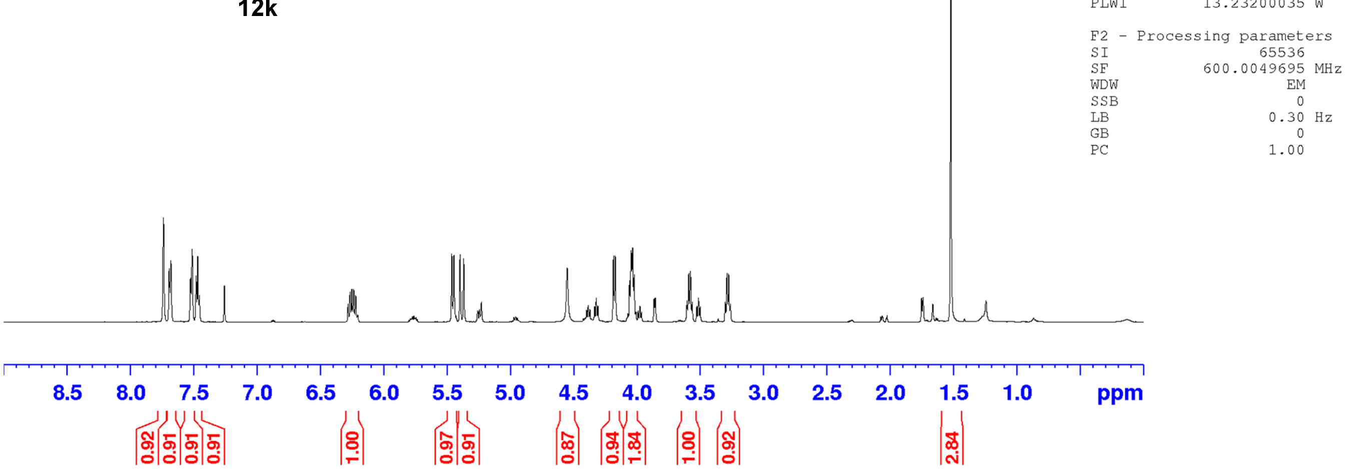

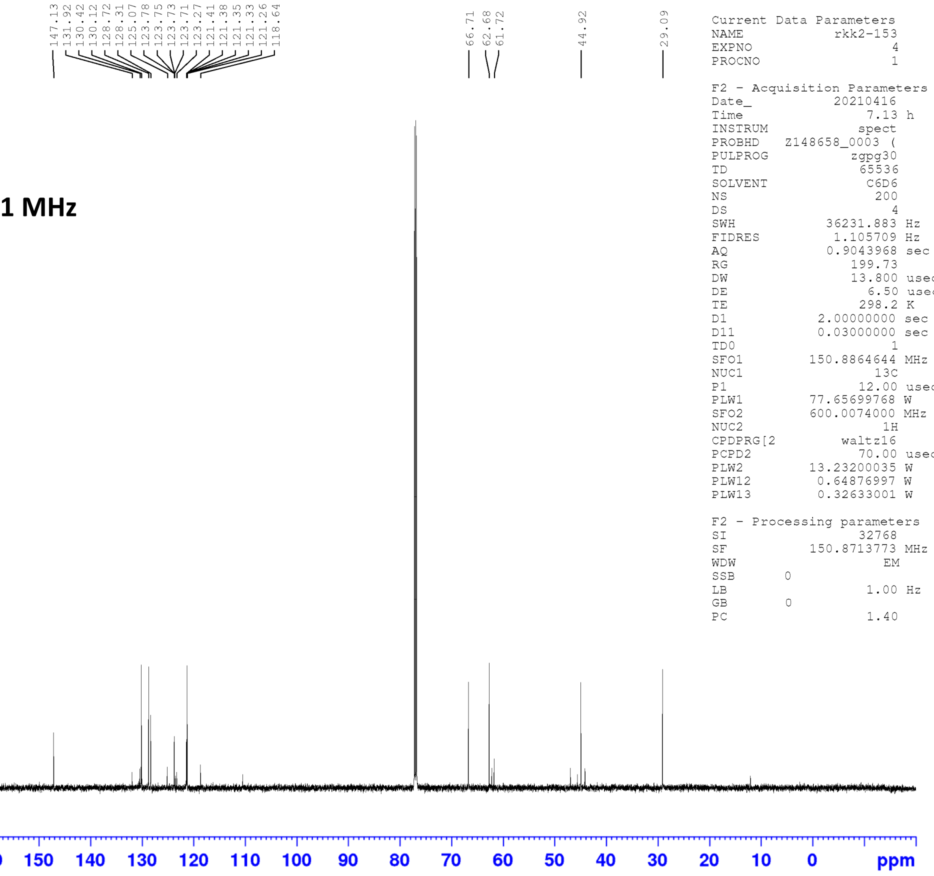


## $^{13}C$ NMR ($CDCl_3$), 151 MHz

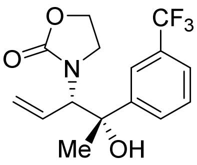

**12k**

```
Current Data Parameters
NAME            rkk2-153
EXPNO                  4
PROCNO                 1

F2 - Acquisition Parameters
Date_           20210416
Time                7.13 h
INSTRUM            spect
PROBHD   Z148658_0003 (
PULPROG           zgpg30
TD                 65536
SOLVENT             C6D6
NS                   200
DS                     4
SWH            36231.883 Hz
FIDRES          1.105709 Hz
AQ             0.9043968 sec
RG                199.73
DW                13.800 usec
DE                  6.50 usec
TE                 298.2 K
D1            2.00000000 sec
D11           0.03000000 sec
TD0                    1
SFO1         150.8864644 MHz
NUC1                 13C
P1                 12.00 usec
PLW1         77.65699768 W
SFO2         600.0074000 MHz
NUC2                  1H
CPDPRG[2         waltz16
PCPD2              70.00 usec
PLW2         13.23200035 W
PLW12         0.64876997 W
PLW13         0.32633001 W

F2 - Processing parameters
SI                 32768
SF           150.8713773 MHz
WDW                   EM
SSB      0
LB                  1.00 Hz
GB       0
PC                  1.40
```

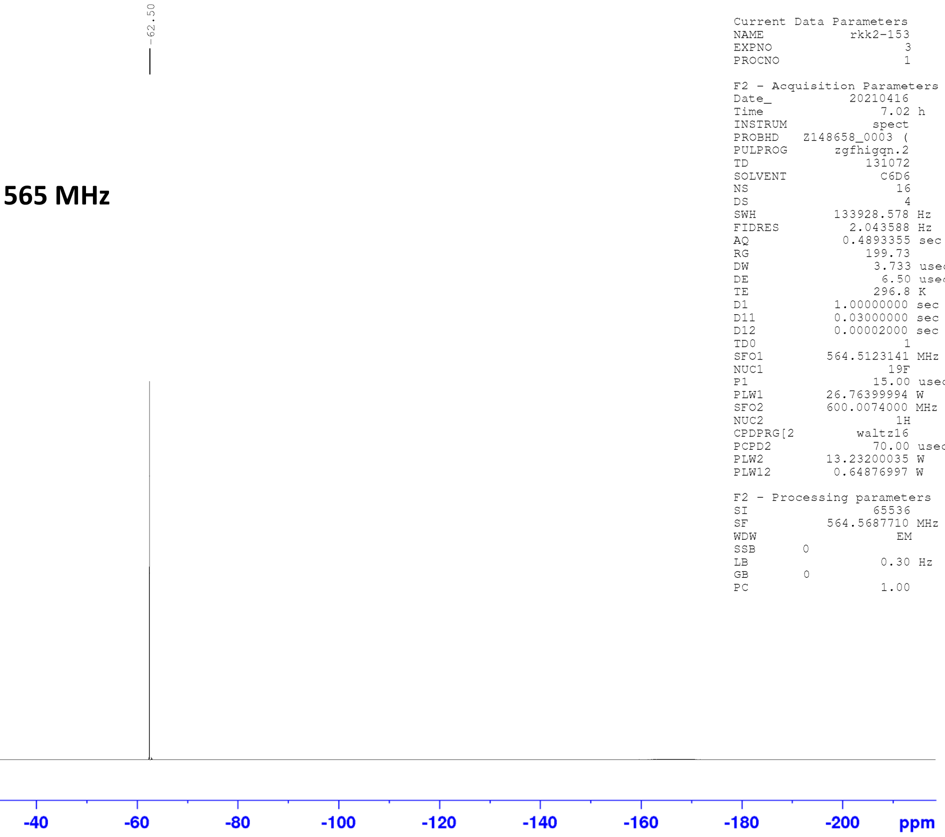


## $^{19}F$ NMR ($CDCl_3$) 565 MHz

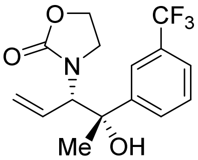

**12k**

```
Current Data Parameters
NAME            rkk2-153
EXPNO                  3
PROCNO                 1

F2 - Acquisition Parameters
Date_           20210416
Time                7.02 h
INSTRUM            spect
PROBHD   Z148658_0003 (
PULPROG      zgfhigqn.2
TD                131072
SOLVENT             C6D6
NS                    16
DS                     4
SWH           133928.578 Hz
FIDRES          2.043588 Hz
AQ             0.4893355 sec
RG                199.73
DW                 3.733 usec
DE                  6.50 usec
TE                 296.8 K
D1            1.00000000 sec
D11           0.03000000 sec
D12           0.00002000 sec
TD0                    1
SFO1         564.5123141 MHz
NUC1                 19F
P1                 15.00 usec
PLW1         26.76399994 W
SFO2         600.0074000 MHz
NUC2                  1H
CPDPRG[2         waltz16
PCPD2              70.00 usec
PLW2         13.23200035 W
PLW12         0.64876997 W

F2 - Processing parameters
SI                 65536
SF           564.5687710 MHz
WDW                   EM
SSB      0
LB                  0.30 Hz
GB       0
PC                  1.00
```

**$^{1}$H NMR ($CDCl_3$), 600 MHz**

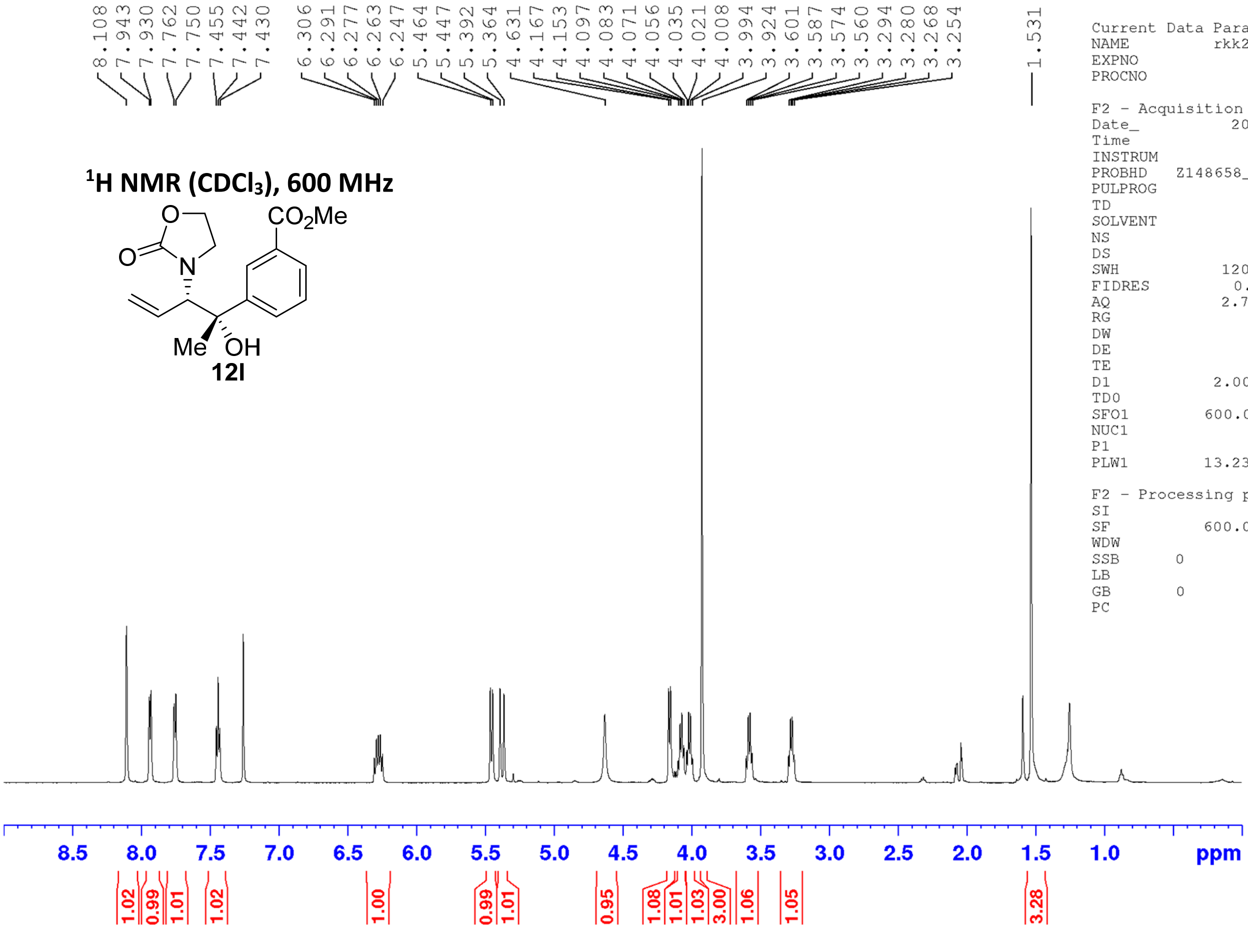


**12l**

```
Current Data Parameters
NAME          rkk2-155_2
EXPNO                  1
PROCNO                 1

F2 - Acquisition Parameters
Date_           20210415
Time              17.13 h
INSTRUM            spect
PROBHD   Z148658_0003 (
PULPROG             zg30
TD                 65536
SOLVENT             C6D6
NS                    20
DS                     2
SWH           12019.230 Hz
FIDRES         0.366798 Hz
AQ            2.7262976 sec
RG               199.73
DW               41.600 usec
DE                 6.50 usec
TE                296.8 K
D1           2.00000000 sec
TD0                    1
SFO1        600.0087050 MHz
NUC1                  1H
P1                15.50 usec
PLW1        13.23200035 W

F2 - Processing parameters
SI                 65536
SF           600.0049684 MHz
WDW                   EM
SSB      0
LB                  0.30 Hz
GB       0
PC                  1.00
```

**$^{13}$C NMR ($CDCl_3$), 151 MHz**

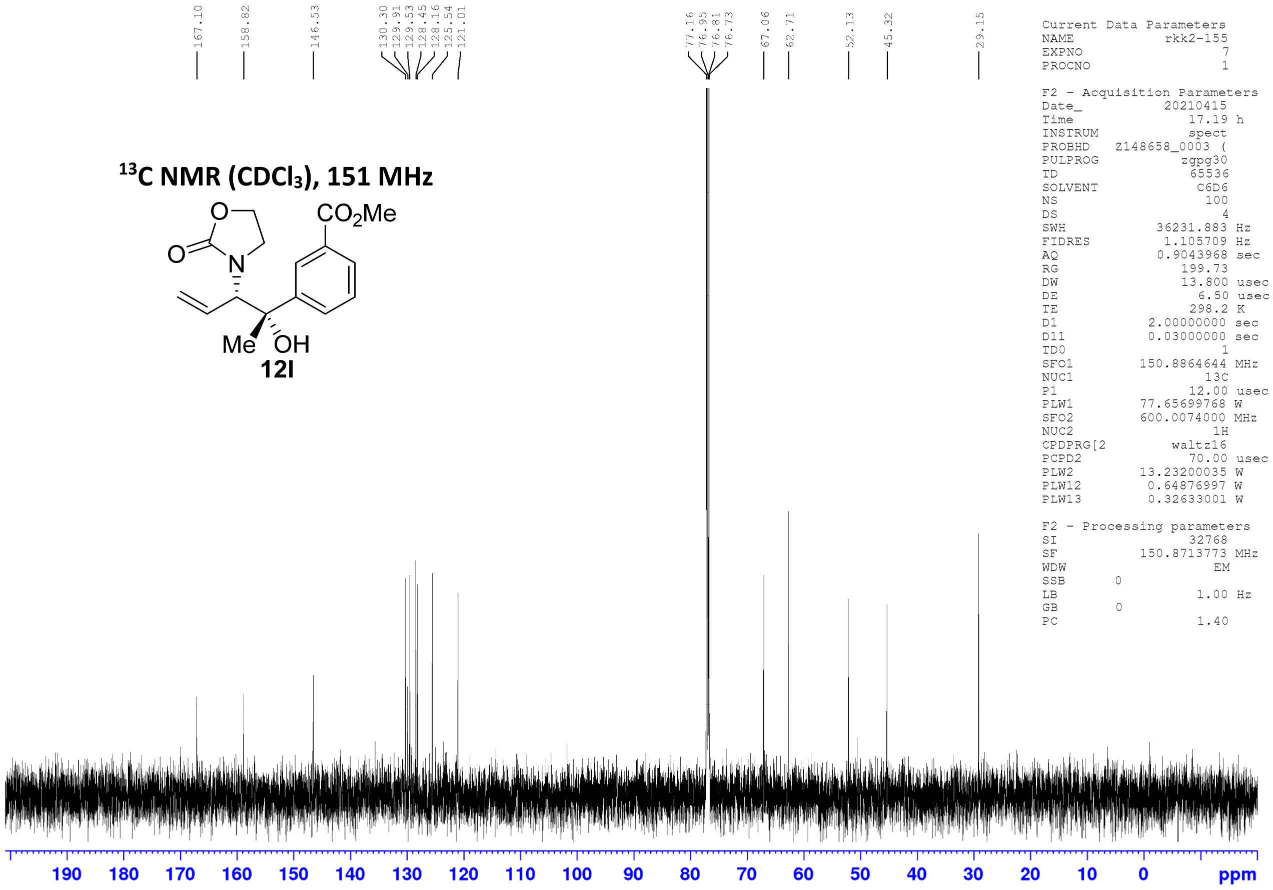


**12l**

```
Current Data Parameters
NAME            rkk2-155
EXPNO                  7
PROCNO                 1

F2 - Acquisition Parameters
Date_           20210415
Time              17.19 h
INSTRUM            spect
PROBHD   Z148658_0003 (
PULPROG           zgpg30
TD                 65536
SOLVENT             C6D6
NS                   100
DS                     4
SWH           36231.883 Hz
FIDRES         1.105709 Hz
AQ            0.9043968 sec
RG               199.73
DW               13.800 usec
DE                 6.50 usec
TE                298.2 K
D1           2.00000000 sec
D11          0.03000000 sec
TD0                    1
SFO1        150.8864644 MHz
NUC1                 13C
P1                12.00 usec
PLW1        77.65699768 W
SFO2        600.0074000 MHz
NUC2                  1H
CPDPRG[2         waltz16
PCPD2             70.00 usec
PLW2        13.23200035 W
PLW12        0.64876997 W
PLW13        0.32633001 W

F2 - Processing parameters
SI                 32768
SF           150.8713773 MHz
WDW                   EM
SSB      0
LB                  1.00 Hz
GB       0
PC                  1.40
```

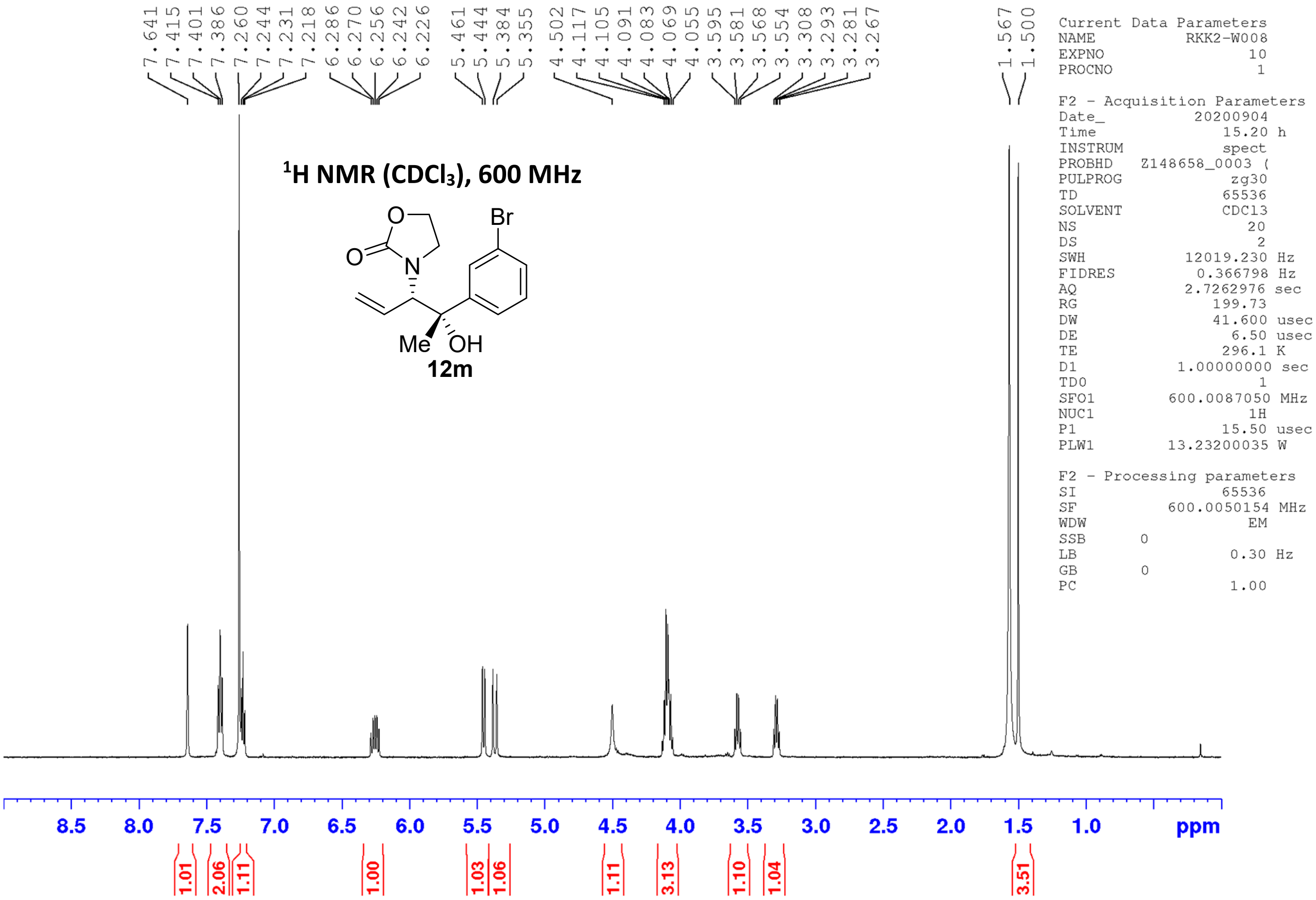

7.641
7.415
7.401
7.386
7.260
7.244
7.231
7.218
6.286
6.270
6.256
6.242
6.226
5.461
5.444
5.384
5.355
4.502
4.117
4.105
4.091
4.083
4.069
4.055
3.595
3.581
3.568
3.554
3.308
3.293
3.281
3.267
1.567
1.500
1H NMR (CDCl3), 600 MHz
O
N
O
Br
Me
OH
12m
Current Data Parameters
NAME RKK2-W008
EXPNO 10
PROCNO 1
F2 - Acquisition Parameters
Date_ 20200904
Time 15.20 h
INSTRUM spect
PROBHD Z148658_0003 (
PULPROG zg30
TD 65536
SOLVENT CDCl3
NS 20
DS 2
SWH 12019.230 Hz
FIDRES 0.366798 Hz
AQ 2.7262976 sec
RG 199.73
DW 41.600 usec
DE 6.50 usec
TE 296.1 K
D1 1.00000000 sec
TD0 1
SFO1 600.0087050 MHz
NUC1 1H
P1 15.50 usec
PLW1 13.23200035 W
F2 - Processing parameters
SI 65536
SF 600.0050154 MHz
WDW EM
SSB 0
LB 0.30 Hz
GB 0
PC 1.00
8.5
8.0
7.5
7.0
6.5
6.0
5.5
5.0
4.5
4.0
3.5
3.0
2.5
2.0
1.5
1.0
ppm
1.01
2.06
1.11
1.00
1.03
1.06
1.11
3.13
1.10
1.04
3.51


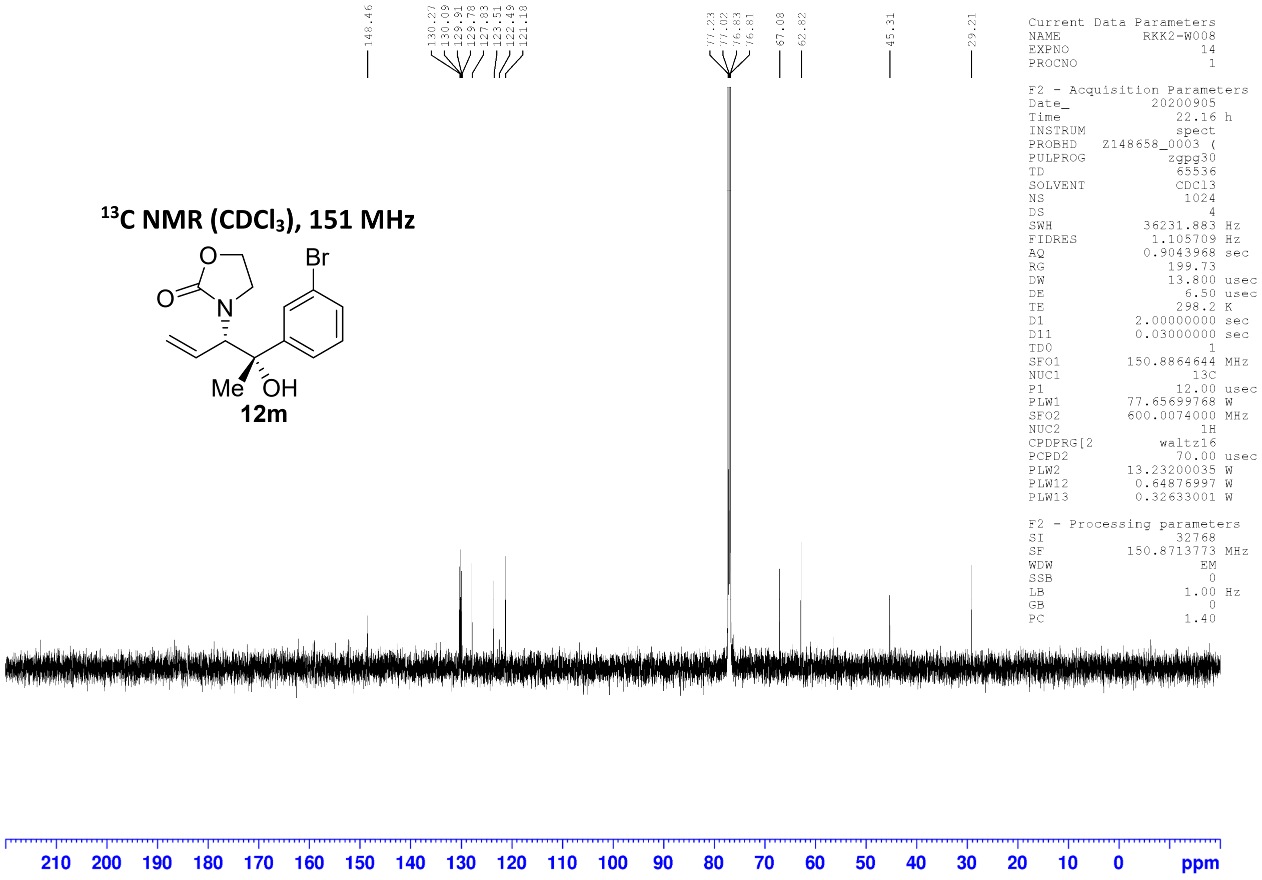

148.46
130.27
130.09
129.91
129.78
127.83
123.51
122.49
121.18
77.23
77.02
76.83
76.81
67.08
62.82
45.31
29.21
13C NMR (CDCl3), 151 MHz
O
N
O
Br
Me
OH
12m
Current Data Parameters
NAME RKK2-W008
EXPNO 14
PROCNO 1
F2 - Acquisition Parameters
Date_ 20200905
Time 22.16 h
INSTRUM spect
PROBHD Z148658_0003 (
PULPROG zgpg30
TD 65536
SOLVENT CDCl3
NS 1024
DS 4
SWH 36231.883 Hz
FIDRES 1.105709 Hz
AQ 0.9043968 sec
RG 199.73
DW 13.800 usec
DE 6.50 usec
TE 298.2 K
D1 2.00000000 sec
D11 0.03000000 sec
TD0 1
SFO1 150.8864644 MHz
NUC1 13C
P1 12.00 usec
PLW1 77.65699768 W
SFO2 600.0074000 MHz
NUC2 1H
CPDPRG[2 waltz16
PCPD2 70.00 usec
PLW2 13.23200035 W
PLW12 0.64876997 W
PLW13 0.32633001 W
F2 - Processing parameters
SI 32768
SF 150.8713773 MHz
WDW EM
SSB 0
LB 1.00 Hz
GB 0
PC 1.40
210
200
190
180
170
160
150
140
130
120
110
100
90
80
70
60
50
40
30
20
10
0
ppm

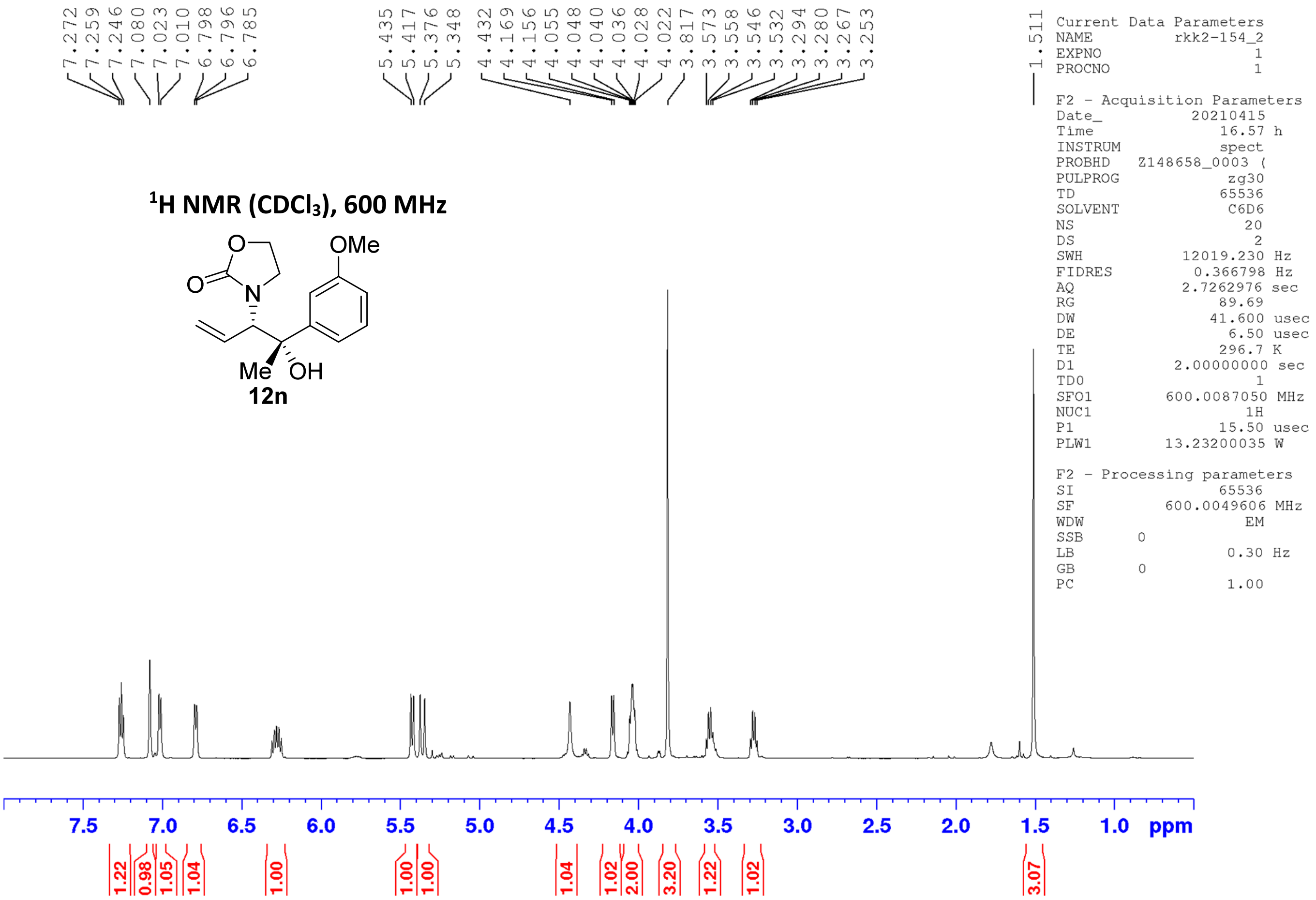
7.272
7.259
7.246
7.080
7.023
7.010
6.798
6.796
6.785
5.435
5.417
5.376
5.348
4.432
4.169
4.156
4.055
4.048
4.040
4.036
4.028
4.022
3.817
3.573
3.558
3.546
3.532
3.294
3.280
3.267
3.253
1.511
Current Data Parameters
NAME rkk2-154_2
EXPNO 1
PROCNO 1
F2 - Acquisition Parameters
Date_ 20210415
Time 16.57 h
INSTRUM spect
PROBHD Z148658_0003 (
PULPROG zg30
TD 65536
SOLVENT C6D6
NS 20
DS 2
SWH 12019.230 Hz
FIDRES 0.366798 Hz
AQ 2.7262976 sec
RG 89.69
DW 41.600 usec
DE 6.50 usec
TE 296.7 K
D1 2.00000000 sec
TD0 1
SFO1 600.0087050 MHz
NUC1 1H
P1 15.50 usec
PLW1 13.23200035 W
F2 - Processing parameters
SI 65536
SF 600.0049606 MHz
WDW EM
SSB 0
LB 0.30 Hz
GB 0
PC 1.00
1H NMR (CDCl3), 600 MHz
OMe
O
N
Me
OH
12n
7.5
7.0
6.5
6.0
5.5
5.0
4.5
4.0
3.5
3.0
2.5
2.0
1.5
1.0
ppm
1.22
0.98
1.05
1.04
1.00
1.00
1.00
1.04
1.02
2.00
3.20
1.22
1.02
3.07

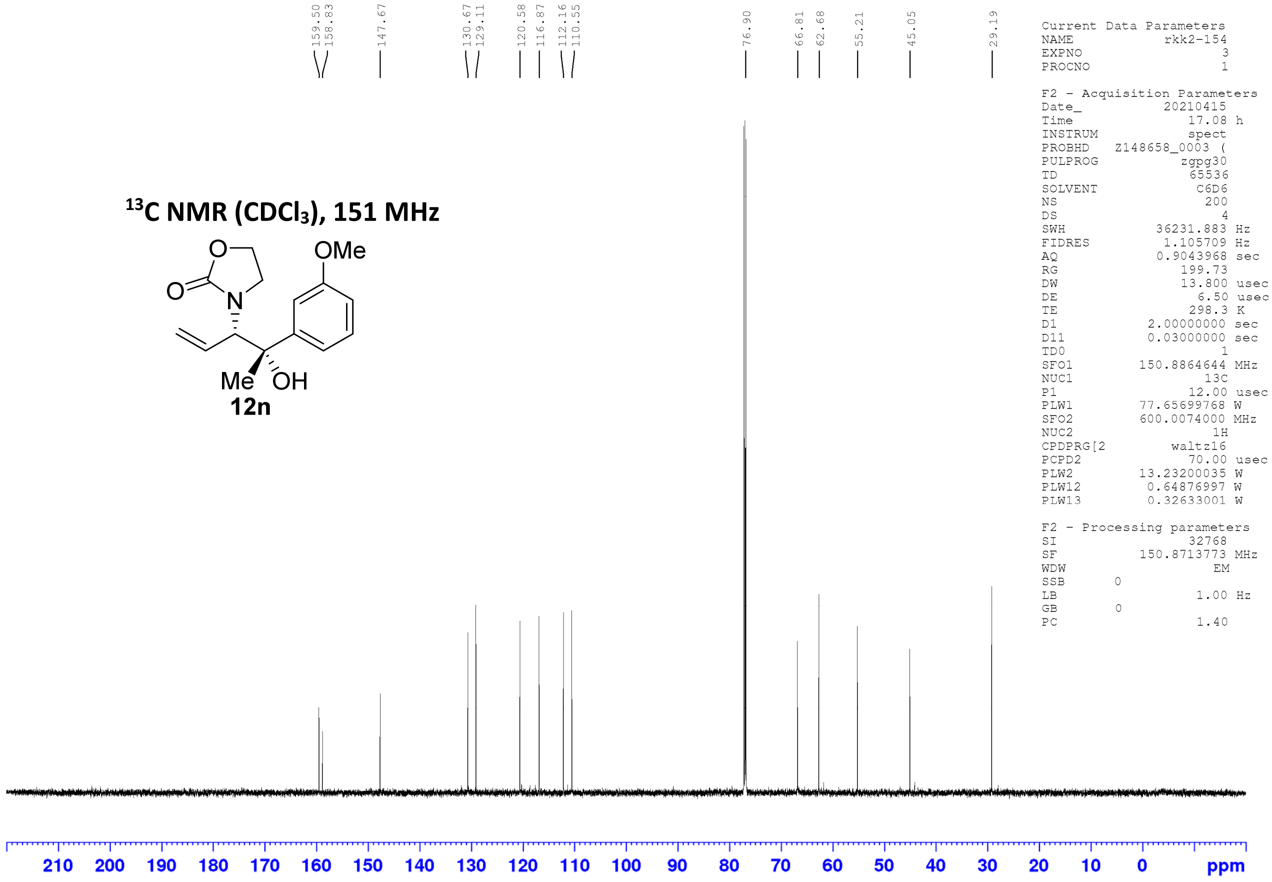
159.50
158.83
147.67
130.67
129.11
120.58
116.87
112.16
110.55
76.90
66.81
62.68
55.21
45.05
29.19
Current Data Parameters
NAME rkk2-154
EXPNO 3
PROCNO 1
F2 - Acquisition Parameters
Date_ 20210415
Time 17.08 h
INSTRUM spect
PROBHD Z148658_0003 (
PULPROG zgpg30
TD 65536
SOLVENT C6D6
NS 200
DS 4
SWH 36231.883 Hz
FIDRES 1.105709 Hz
AQ 0.9043968 sec
RG 199.73
DW 13.800 usec
DE 6.50 usec
TE 298.3 K
D1 2.00000000 sec
D11 0.03000000 sec
TD0 1
SFO1 150.8864644 MHz
NUC1 13C
P1 12.00 usec
PLW1 77.65699768 W
SFO2 600.0074000 MHz
NUC2 1H
CPDPRG[2 waltz16
PCPD2 70.00 usec
PLW2 13.23200035 W
PLW12 0.64876997 W
PLW13 0.32633001 W
F2 - Processing parameters
SI 32768
SF 150.8713773 MHz
WDW EM
SSB 0
LB 1.00 Hz
GB 0
PC 1.40
13C NMR (CDCl3), 151 MHz
OMe
O
N
Me
OH
12n
210
200
190
180
170
160
150
140
130
120
110
100
90
80
70
60
50
40
30
20
10
0
ppm

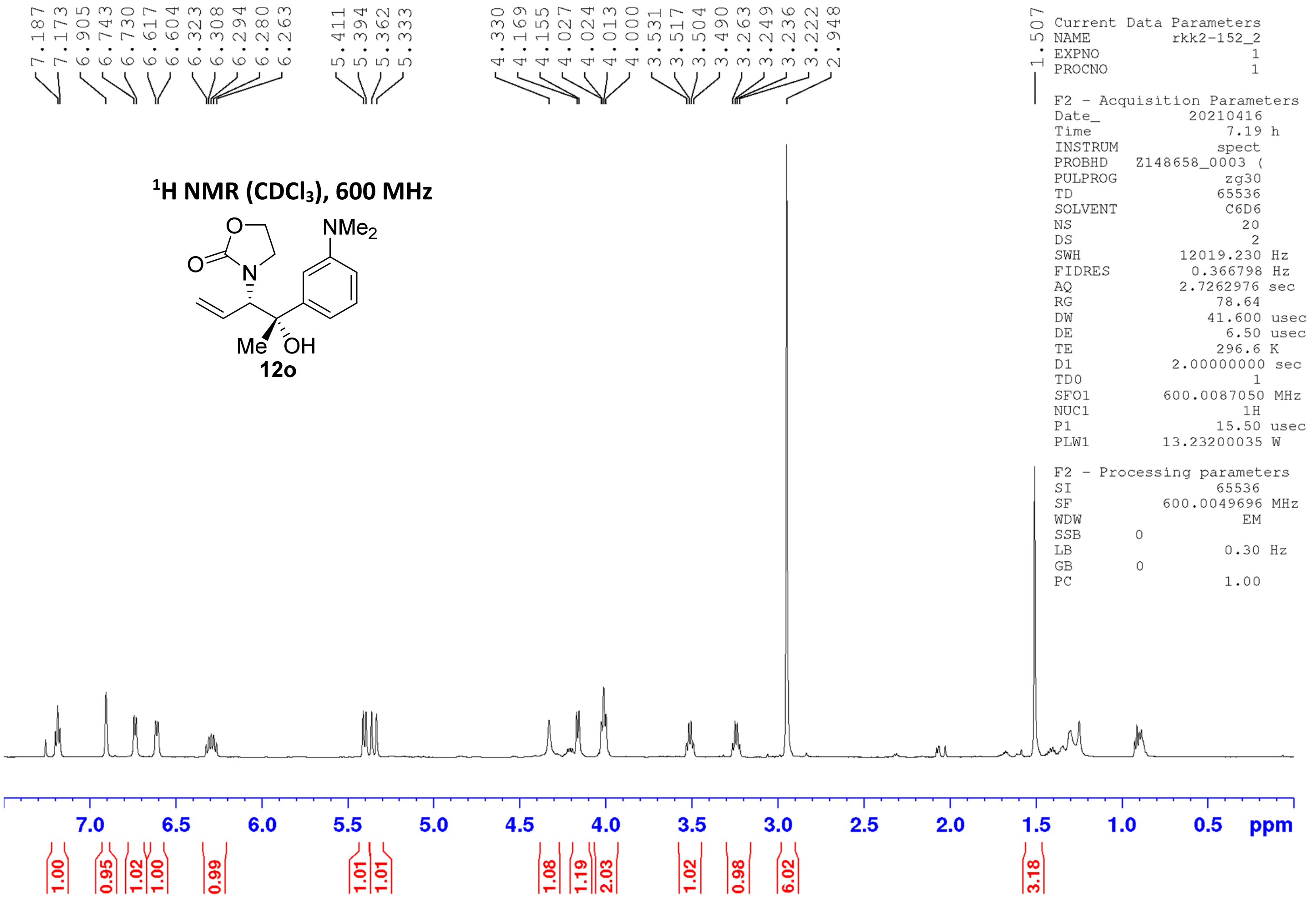

1H NMR (CDCl3), 600 MHz
NMe2
O
N
OH
Me
12o
7.187
7.173
6.905
6.743
6.730
6.617
6.604
6.323
6.308
6.294
6.280
6.263
5.411
5.394
5.362
5.333
4.330
4.169
4.155
4.027
4.024
4.013
4.000
3.531
3.517
3.504
3.490
3.263
3.249
3.236
3.222
2.948
1.507
Current Data Parameters
NAME rkk2-152_2
EXPNO 1
PROCNO 1
F2 - Acquisition Parameters
Date_ 20210416
Time 7.19 h
INSTRUM spect
PROBHD Z148658_0003 (
PULPROG zg30
TD 65536
SOLVENT C6D6
NS 20
DS 2
SWH 12019.230 Hz
FIDRES 0.366798 Hz
AQ 2.7262976 sec
RG 78.64
DW 41.600 usec
DE 6.50 usec
TE 296.6 K
D1 2.00000000 sec
TD0 1
SFO1 600.0087050 MHz
NUC1 1H
P1 15.50 usec
PLW1 13.23200035 W
F2 - Processing parameters
SI 65536
SF 600.0049696 MHz
WDW EM
SSB 0
LB 0.30 Hz
GB 0
PC 1.00
7.0
6.5
6.0
5.5
5.0
4.5
4.0
3.5
3.0
2.5
2.0
1.5
1.0
0.5
ppm
1.00
0.95
1.02
1.00
0.99
1.01
1.01
1.08
1.19
2.03
1.02
0.98
6.02
3.18


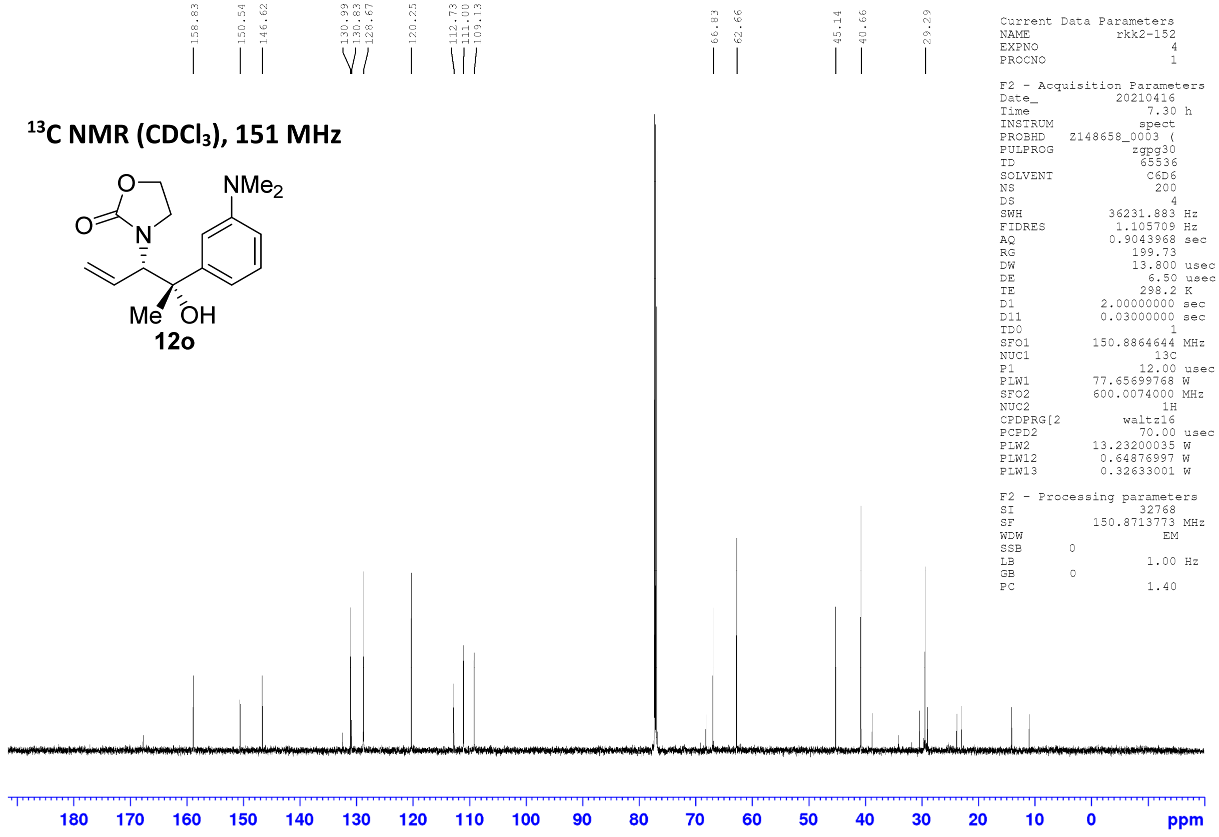

13C NMR (CDCl3), 151 MHz
NMe2
O
N
OH
Me
12o
158.83
150.54
146.62
130.99
130.83
128.67
120.25
112.73
111.00
109.13
66.83
62.66
45.14
40.66
29.29
Current Data Parameters
NAME rkk2-152
EXPNO 4
PROCNO 1
F2 - Acquisition Parameters
Date_ 20210416
Time 7.30 h
INSTRUM spect
PROBHD Z148658_0003 (
PULPROG zgpg30
TD 65536
SOLVENT C6D6
NS 200
DS 4
SWH 36231.883 Hz
FIDRES 1.105709 Hz
AQ 0.9043968 sec
RG 199.73
DW 13.800 usec
DE 6.50 usec
TE 298.2 K
D1 2.00000000 sec
D11 0.03000000 sec
TD0 1
SFO1 150.8864644 MHz
NUC1 13C
P1 12.00 usec
PLW1 77.65699768 W
SFO2 600.0074000 MHz
NUC2 1H
CPDPRG[2 waltz16
PCPD2 70.00 usec
PLW2 13.23200035 W
PLW12 0.64876997 W
PLW13 0.32633001 W
F2 - Processing parameters
SI 32768
SF 150.8713773 MHz
WDW EM
SSB 0
LB 1.00 Hz
GB 0
PC 1.40
180
170
160
150
140
130
120
110
100
90
80
70
60
50
40
30
20
10
0
ppm

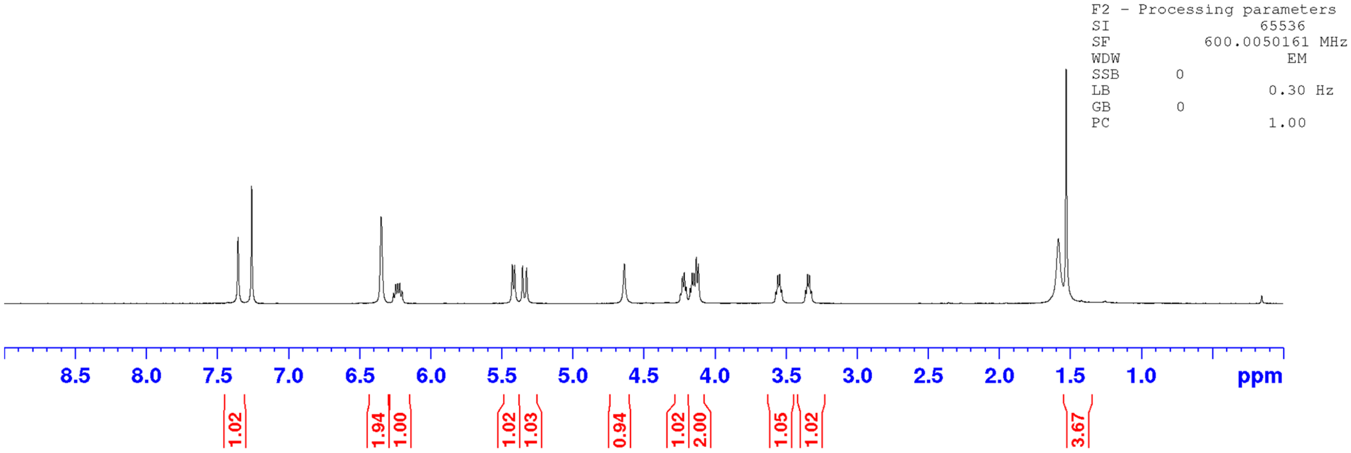


Current Data Parameters
NAME RKK2-W008
EXPNO 11
PROCNO 1

F2 - Acquisition Parameters
Date_ 20200904
Time 15.24 h
INSTRUM spect
PROBHD Z148658_0003 (
PULPROG zg30
TD 65536
SOLVENT CDCl3
NS 20
DS 2
SWH 12019.230 Hz
FIDRES 0.366798 Hz
AQ 2.7262976 sec
RG 199.73
DW 41.600 usec
DE 6.50 usec
TE 296.1 K
D1 1.00000000 sec
TD0 1
SFO1 600.0087050 MHz
NUC1 1H
P1 15.50 usec
PLW1 13.23200035 W

F2 - Processing parameters
SI 65536
SF 600.0050161 MHz
WDW EM
SSB 0
LB 0.30 Hz
GB 0
PC 1.00

**$^{13}$C NMR ($CDCl_3$), 151 MHz**

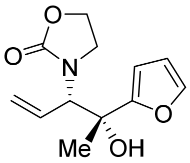


**12p**

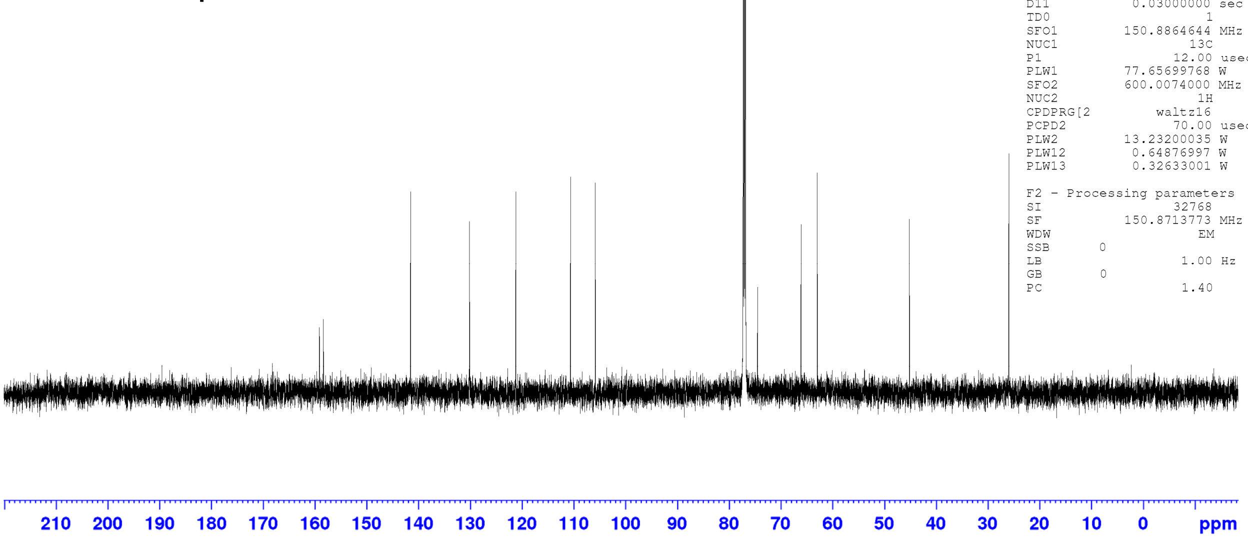


Current Data Parameters
NAME RKK2-W008
EXPNO 15
PROCNO 1

F2 - Acquisition Parameters
Date_ 20200905
Time 21.21 h
INSTRUM spect
PROBHD Z148658_0003 (
PULPROG zgpg30
TD 65536
SOLVENT CDCl3
NS 1024
DS 4
SWH 36231.883 Hz
FIDRES 1.105709 Hz
AQ 0.9043968 sec
RG 199.73
DW 13.800 usec
DE 6.50 usec
TE 298.1 K
D1 2.00000000 sec
D11 0.03000000 sec
TD0 1
SFO1 150.8864644 MHz
NUC1 13C
P1 12.00 usec
PLW1 77.65699768 W
SFO2 600.0074000 MHz
NUC2 1H
CPDPRG[2 waltz16
PCPD2 70.00 usec
PLW2 13.23200035 W
PLW12 0.64876997 W
PLW13 0.32633001 W

F2 - Processing parameters
SI 32768
SF 150.8713773 MHz
WDW EM
SSB 0
LB 1.00 Hz
GB 0
PC 1.40

**$^{1}$H NMR ($CDCl_3$), 600 MHz**

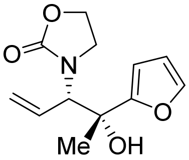


**12p**

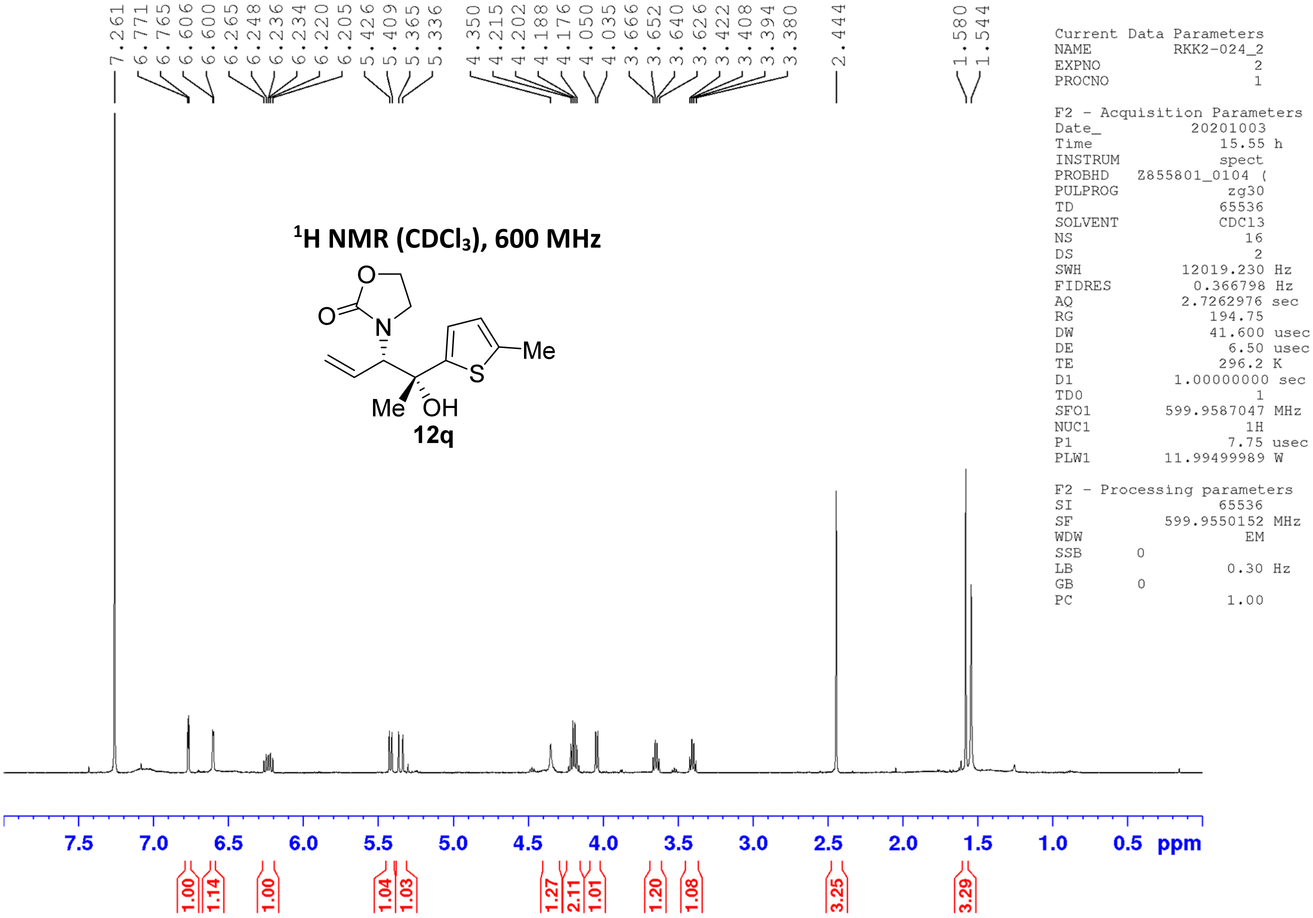


```
Current Data Parameters
NAME           RKK2-024_2
EXPNO                   2
PROCNO                  1

F2 - Acquisition Parameters
Date_            20201003
Time              15.55 h
INSTRUM             spect
PROBHD   Z855801_0104 (
PULPROG              zg30
TD                  65536
SOLVENT             CDCl3
NS                     16
DS                      2
SWH            12019.230 Hz
FIDRES          0.366798 Hz
AQ             2.7262976 sec
RG                 194.75
DW                 41.600 usec
DE                   6.50 usec
TE                  296.2 K
D1            1.00000000 sec
TD0                     1
SFO1         599.9587047 MHz
NUC1                   1H
P1                   7.75 usec
PLW1         11.99499989 W

F2 - Processing parameters
SI                  65536
SF           599.9550152 MHz
WDW                    EM
SSB       0
LB                   0.30 Hz
GB        0
PC                   1.00
```

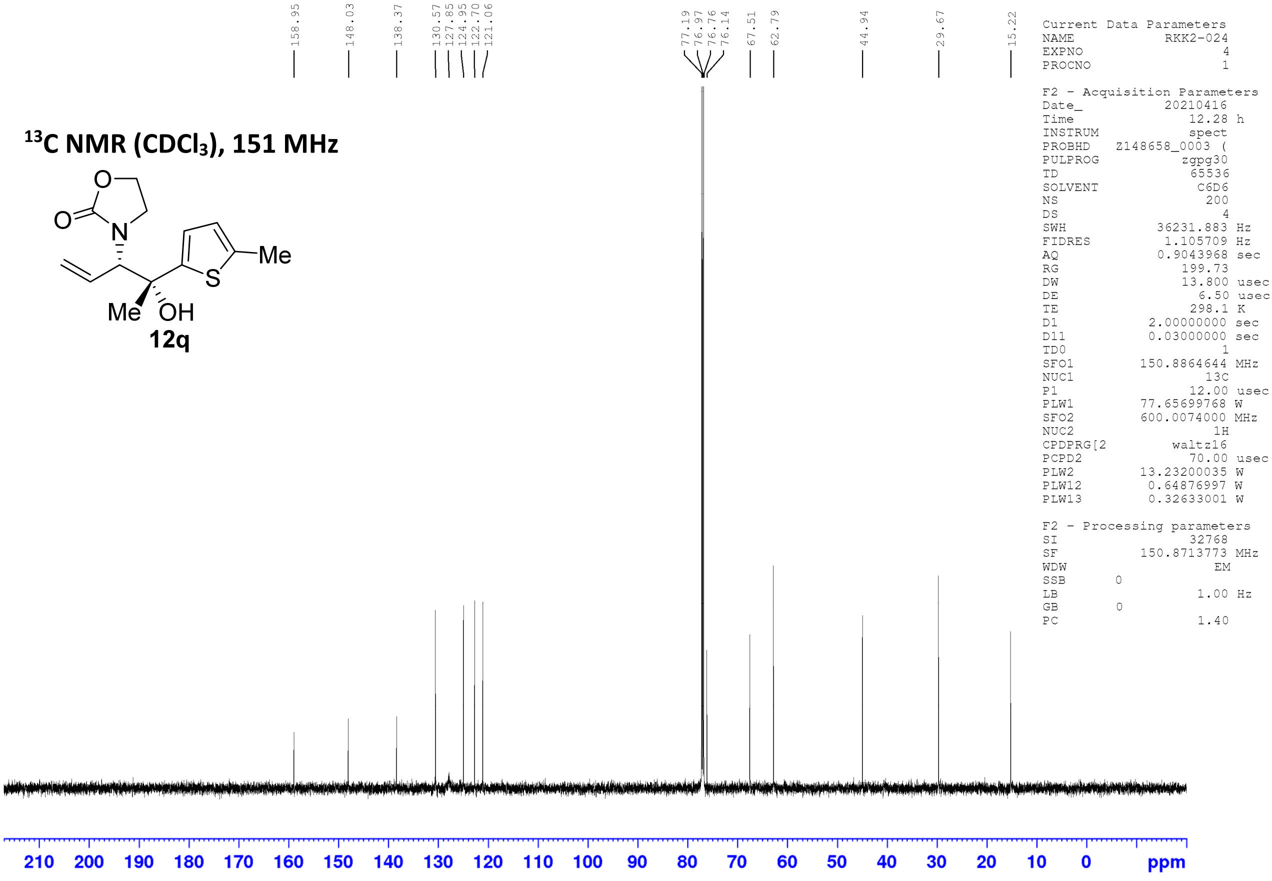


```
Current Data Parameters
NAME             RKK2-024
EXPNO                   4
PROCNO                  1

F2 - Acquisition Parameters
Date_            20210416
Time              12.28 h
INSTRUM             spect
PROBHD   Z148658_0003 (
PULPROG            zgpg30
TD                  65536
SOLVENT              C6D6
NS                    200
DS                      4
SWH            36231.883 Hz
FIDRES          1.105709 Hz
AQ             0.9043968 sec
RG                 199.73
DW                 13.800 usec
DE                   6.50 usec
TE                  298.1 K
D1            2.00000000 sec
D11           0.03000000 sec
TD0                     1
SFO1         150.8864644 MHz
NUC1                  13C
P1                  12.00 usec
PLW1         77.65699768 W
SFO2         600.0074000 MHz
NUC2                   1H
CPDPRG[2          waltz16
PCPD2               70.00 usec
PLW2         13.23200035 W
PLW12         0.64876997 W
PLW13         0.32633001 W

F2 - Processing parameters
SI                  32768
SF           150.8713773 MHz
WDW                    EM
SSB       0
LB                   1.00 Hz
GB        0
PC                   1.40
```

**$^{1}H$ NMR ($CDCl_3$), 600 MHz**

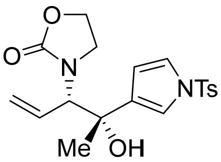


**12r**

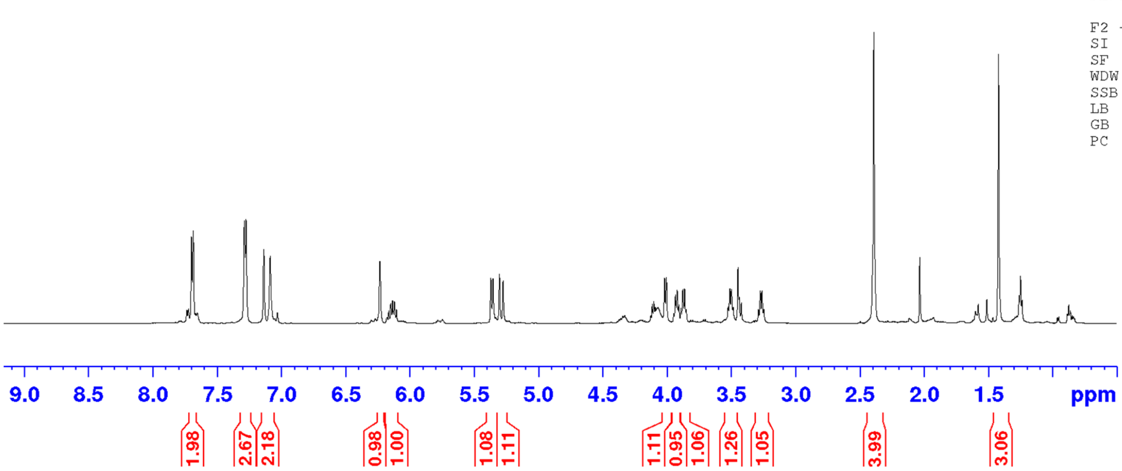


Current Data Parameters
NAME RKK2-027_2
EXPNO 4
PROCNO 1

F2 - Acquisition Parameters
Date_ 20210416
Time 7.35 h
INSTRUM spect
PROBHD Z148658_0003 (
PULPROG zg30
TD 65536
SOLVENT C6D6
NS 20
DS 2
SWH 12019.230 Hz
FIDRES 0.366798 Hz
AQ 2.7262976 sec
RG 55.05
DW 41.600 usec
DE 6.50 usec
TE 296.7 K
D1 2.00000000 sec
TD0 1
SFO1 600.0087050 MHz
NUC1 1H
P1 15.50 usec
PLW1 13.23200035 W

F2 - Processing parameters
SI 65536
SF 600.0049607 MHz
WDW EM
SSB 0
LB 0.30 Hz
GB 0
PC 1.00

**$^{13}C$ NMR ($CDCl_3$), 151 MHz**

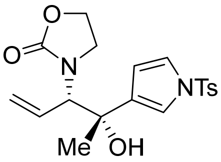


**12r**

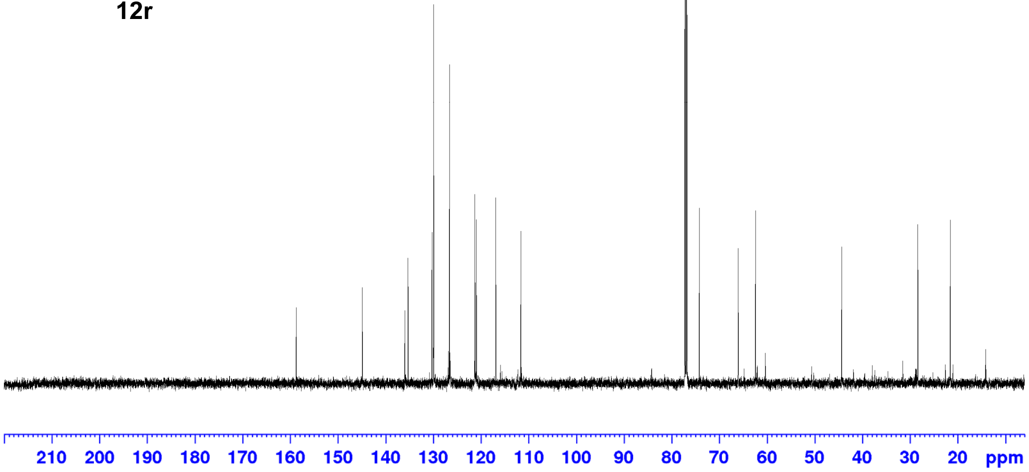


Current Data Parameters
NAME RKK2-027_2
EXPNO 5
PROCNO 1

F2 - Acquisition Parameters
Date_ 20210416
Time 7.41 h
INSTRUM spect
PROBHD Z148658_0003 (
PULPROG zgpg30
TD 65536
SOLVENT C6D6
NS 100
DS 4
SWH 36231.883 Hz
FIDRES 1.105709 Hz
AQ 0.9043968 sec
RG 199.73
DW 13.800 usec
DE 6.50 usec
TE 298.1 K
D1 2.00000000 sec
D11 0.03000000 sec
TD0 1
SFO1 150.8864644 MHz
NUC1 13C
P1 12.00 usec
PLW1 77.65699768 W
SFO2 600.0074000 MHz
NUC2 1H
CPDPRG[2 waltz16
PCPD2 70.00 usec
PLW2 13.23200035 W
PLW12 0.64876997 W
PLW13 0.32633001 W

F2 - Processing parameters
SI 32768
SF 150.8713773 MHz
WDW EM
SSB 0
LB 1.00 Hz
GB 0
PC 1.40

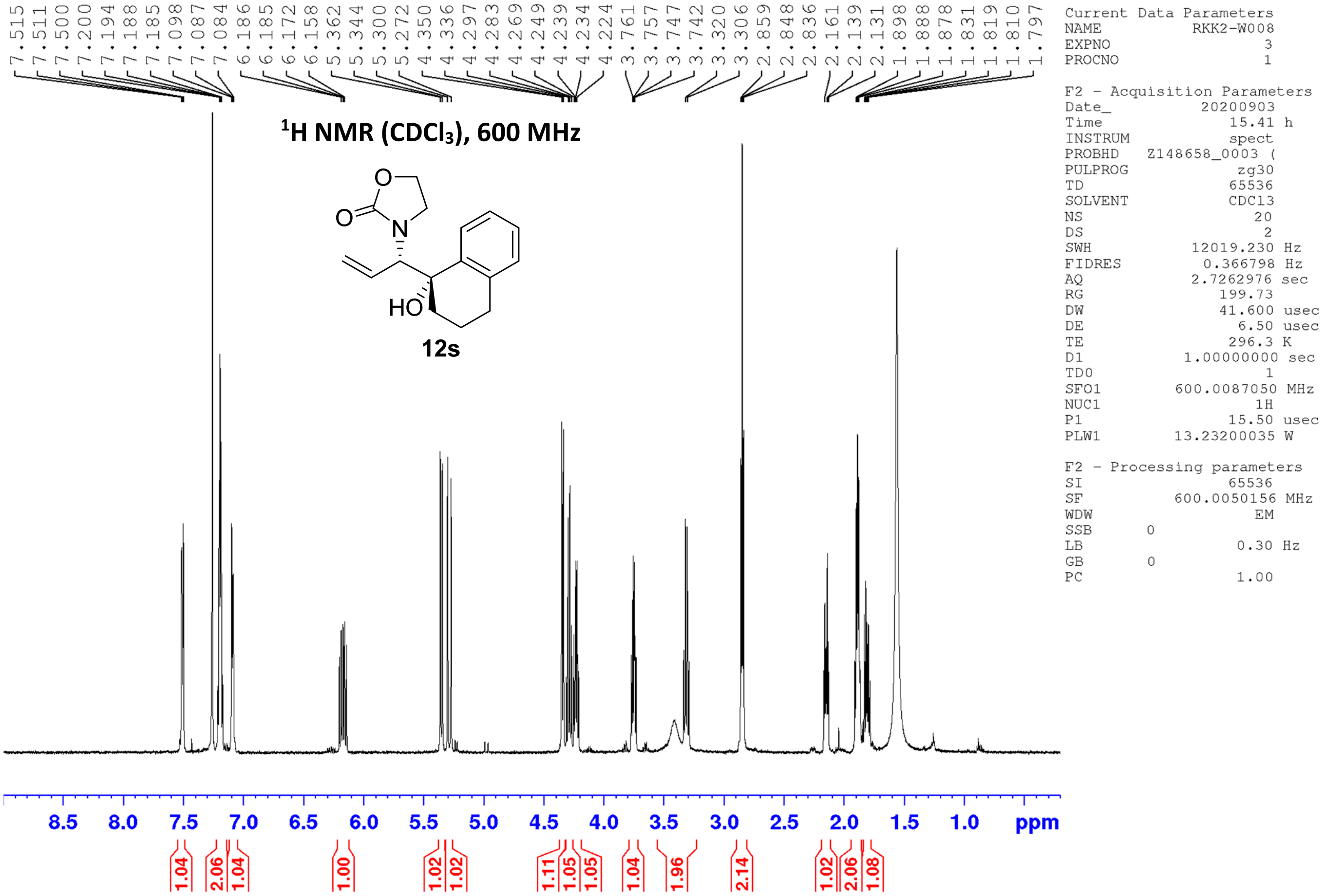

7.515 7.511 7.500 7.200 7.194 7.188 7.185 7.098 7.087 7.084 6.186 6.185 6.172 6.158 5.362 5.344 5.300 5.272 4.350 4.336 4.297 4.283 4.269 4.249 4.239 4.234 4.224 3.761 3.757 3.747 3.742 3.320 3.306 2.859 2.848 2.836 2.161 2.139 2.131 1.898 1.888 1.878 1.831 1.819 1.810 1.797
1H NMR (CDCl3), 600 MHz
O
O
N
HO
12s
Current Data Parameters
NAME RKK2-W008
EXPNO 3
PROCNO 1
F2 - Acquisition Parameters
Date_ 20200903
Time 15.41 h
INSTRUM spect
PROBHD Z148658_0003 (
PULPROG zg30
TD 65536
SOLVENT CDCl3
NS 20
DS 2
SWH 12019.230 Hz
FIDRES 0.366798 Hz
AQ 2.7262976 sec
RG 199.73
DW 41.600 usec
DE 6.50 usec
TE 296.3 K
D1 1.00000000 sec
TD0 1
SFO1 600.0087050 MHz
NUC1 1H
P1 15.50 usec
PLW1 13.23200035 W
F2 - Processing parameters
SI 65536
SF 600.0050156 MHz
WDW EM
SSB 0
LB 0.30 Hz
GB 0
PC 1.00
8.5 8.0 7.5 7.0 6.5 6.0 5.5 5.0 4.5 4.0 3.5 3.0 2.5 2.0 1.5 1.0 ppm
1.04 2.06 1.04 1.00 1.02 1.02 1.11 1.05 1.05 1.04 1.96 2.14 1.02 2.06 1.08


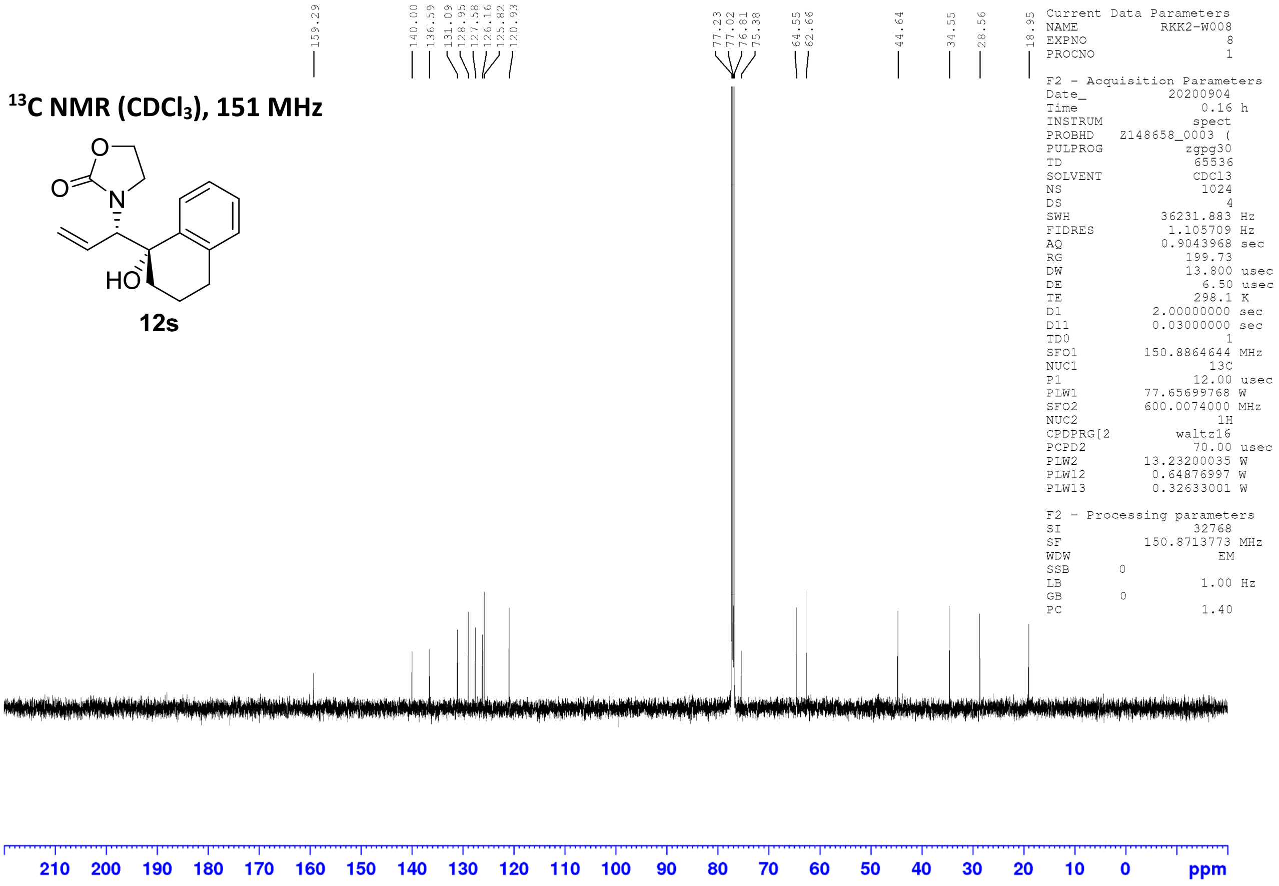

159.29 140.00 136.59 131.09 128.95 127.58 126.16 125.82 120.93 77.23 77.02 76.81 75.38 64.55 62.66 44.64 34.55 28.56 18.95
13C NMR (CDCl3), 151 MHz
O
O
N
HO
12s
Current Data Parameters
NAME RKK2-W008
EXPNO 8
PROCNO 1
F2 - Acquisition Parameters
Date_ 20200904
Time 0.16 h
INSTRUM spect
PROBHD Z148658_0003 (
PULPROG zgpg30
TD 65536
SOLVENT CDCl3
NS 1024
DS 4
SWH 36231.883 Hz
FIDRES 1.105709 Hz
AQ 0.9043968 sec
RG 199.73
DW 13.800 usec
DE 6.50 usec
TE 298.1 K
D1 2.00000000 sec
D11 0.03000000 sec
TD0 1
SFO1 150.8864644 MHz
NUC1 13C
P1 12.00 usec
PLW1 77.65699768 W
SFO2 600.0074000 MHz
NUC2 1H
CPDPRG[2 waltz16
PCPD2 70.00 usec
PLW2 13.23200035 W
PLW12 0.64876997 W
PLW13 0.32633001 W
F2 - Processing parameters
SI 32768
SF 150.8713773 MHz
WDW EM
SSB 0
LB 1.00 Hz
GB 0
PC 1.40
210 200 190 180 170 160 150 140 130 120 110 100 90 80 70 60 50 40 30 20 10 0 ppm

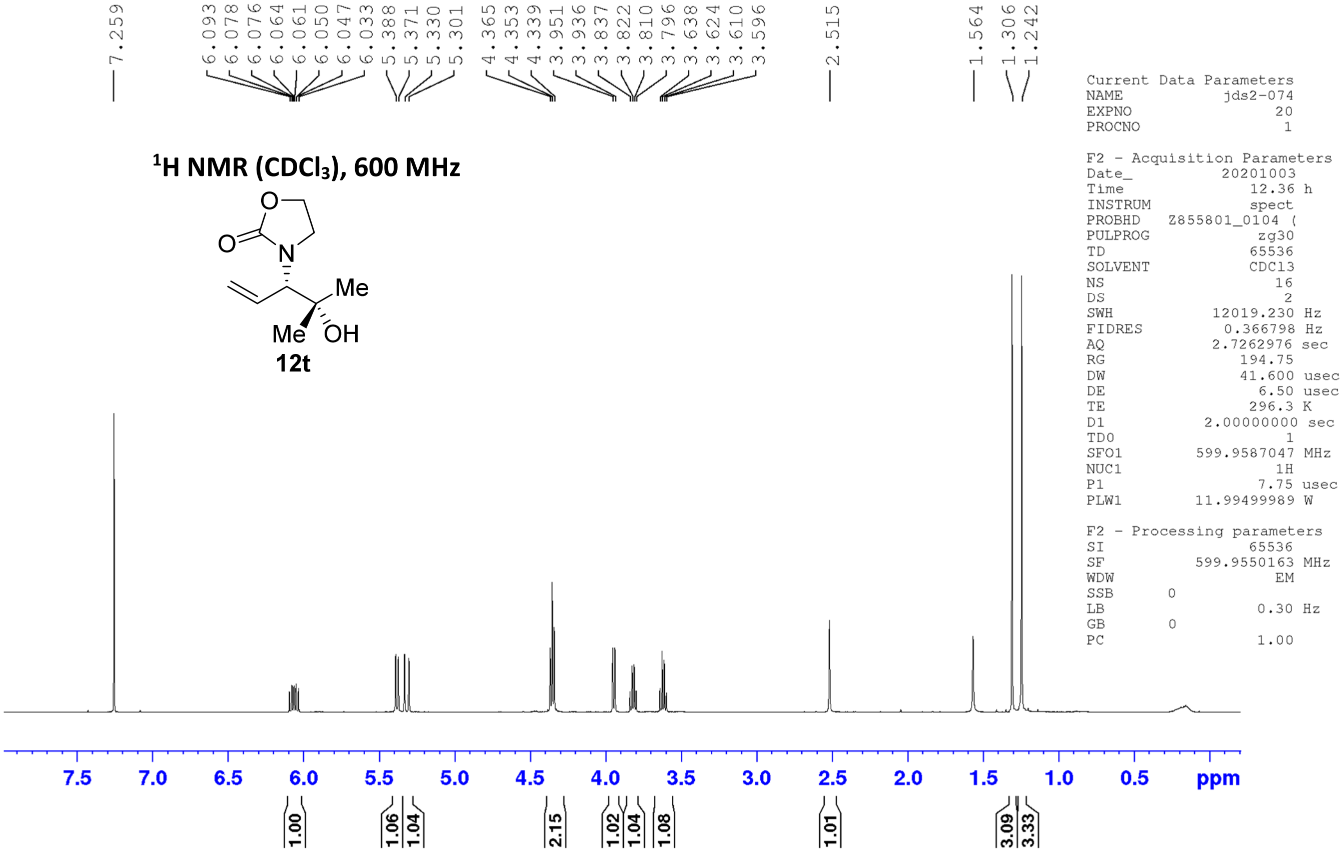

1H NMR (CDCl3), 600 MHz
12t
Me
OH
7.259
6.093
6.078
6.076
6.064
6.061
6.050
6.047
6.033
5.388
5.371
5.330
5.301
4.365
4.353
4.339
3.951
3.936
3.837
3.822
3.810
3.796
3.638
3.624
3.610
3.596
2.515
1.564
1.306
1.242
1.00
1.06
1.04
2.15
1.02
1.04
1.08
1.01
3.09
3.33
ppm
Current Data Parameters
NAME jds2-074
EXPNO 20
PROCNO 1
F2 - Acquisition Parameters
Date_ 20201003
Time 12.36 h
INSTRUM spect
PROBHD Z855801_0104 (
PULPROG zg30
TD 65536
SOLVENT CDCl3
NS 16
DS 2
SWH 12019.230 Hz
FIDRES 0.366798 Hz
AQ 2.7262976 sec
RG 194.75
DW 41.600 usec
DE 6.50 usec
TE 296.3 K
D1 2.00000000 sec
TD0 1
SFO1 599.9587047 MHz
NUC1 1H
P1 7.75 usec
PLW1 11.99499989 W
F2 - Processing parameters
SI 65536
SF 599.9550163 MHz
WDW EM
SSB 0
LB 0.30 Hz
GB 0
PC 1.00


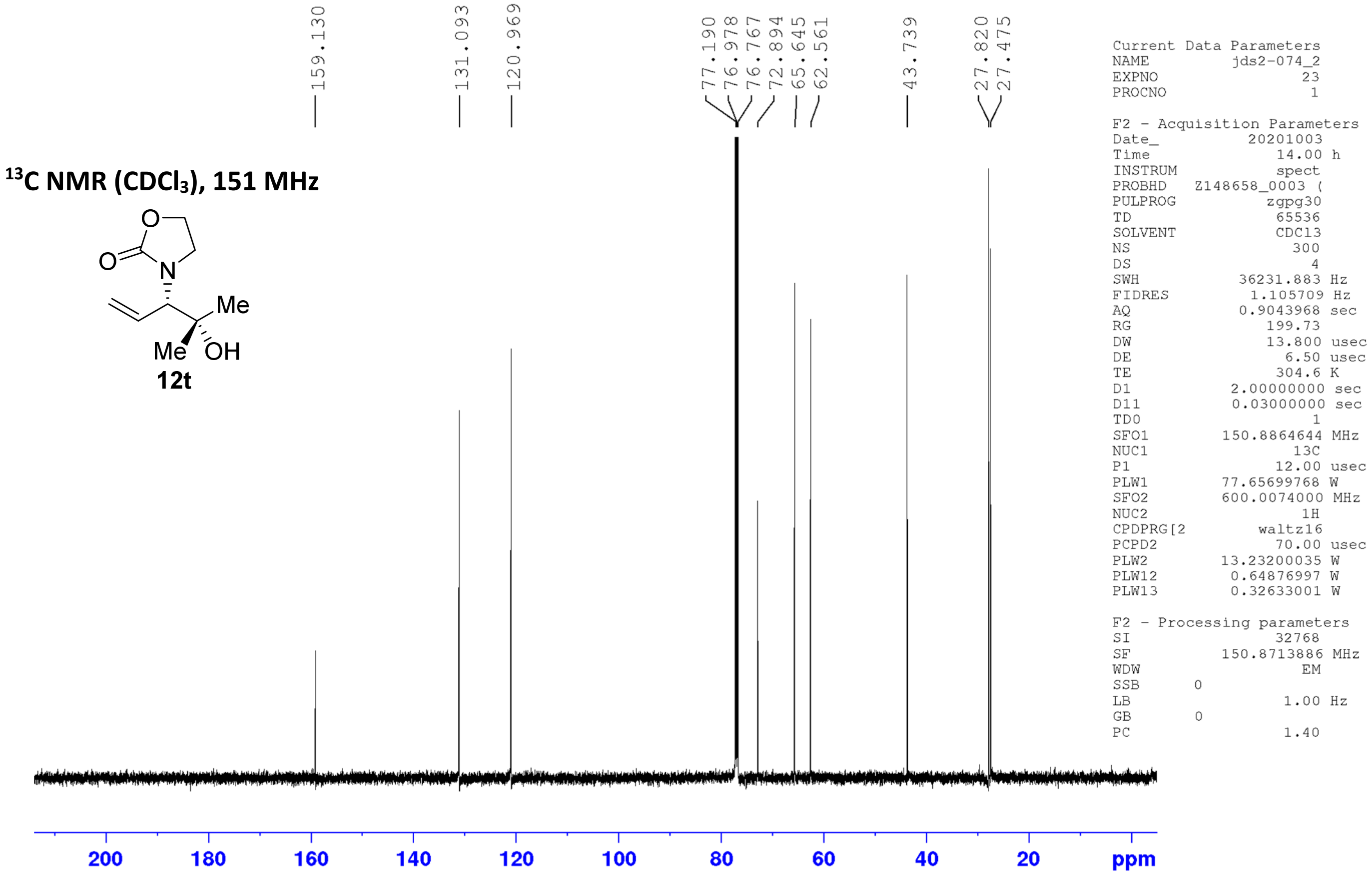

13C NMR (CDCl3), 151 MHz
12t
Me
OH
159.130
131.093
120.969
77.190
76.978
76.767
72.894
65.645
62.561
43.739
27.820
27.475
ppm
Current Data Parameters
NAME jds2-074_2
EXPNO 23
PROCNO 1
F2 - Acquisition Parameters
Date_ 20201003
Time 14.00 h
INSTRUM spect
PROBHD Z148658_0003 (
PULPROG zgpg30
TD 65536
SOLVENT CDCl3
NS 300
DS 4
SWH 36231.883 Hz
FIDRES 1.105709 Hz
AQ 0.9043968 sec
RG 199.73
DW 13.800 usec
DE 6.50 usec
TE 304.6 K
D1 2.00000000 sec
D11 0.03000000 sec
TD0 1
SFO1 150.8864644 MHz
NUC1 13C
P1 12.00 usec
PLW1 77.65699768 W
SFO2 600.0074000 MHz
NUC2 1H
CPDPRG[2 waltz16
PCPD2 70.00 usec
PLW2 13.23200035 W
PLW12 0.64876997 W
PLW13 0.32633001 W
F2 - Processing parameters
SI 32768
SF 150.8713886 MHz
WDW EM
SSB 0
LB 1.00 Hz
GB 0
PC 1.40

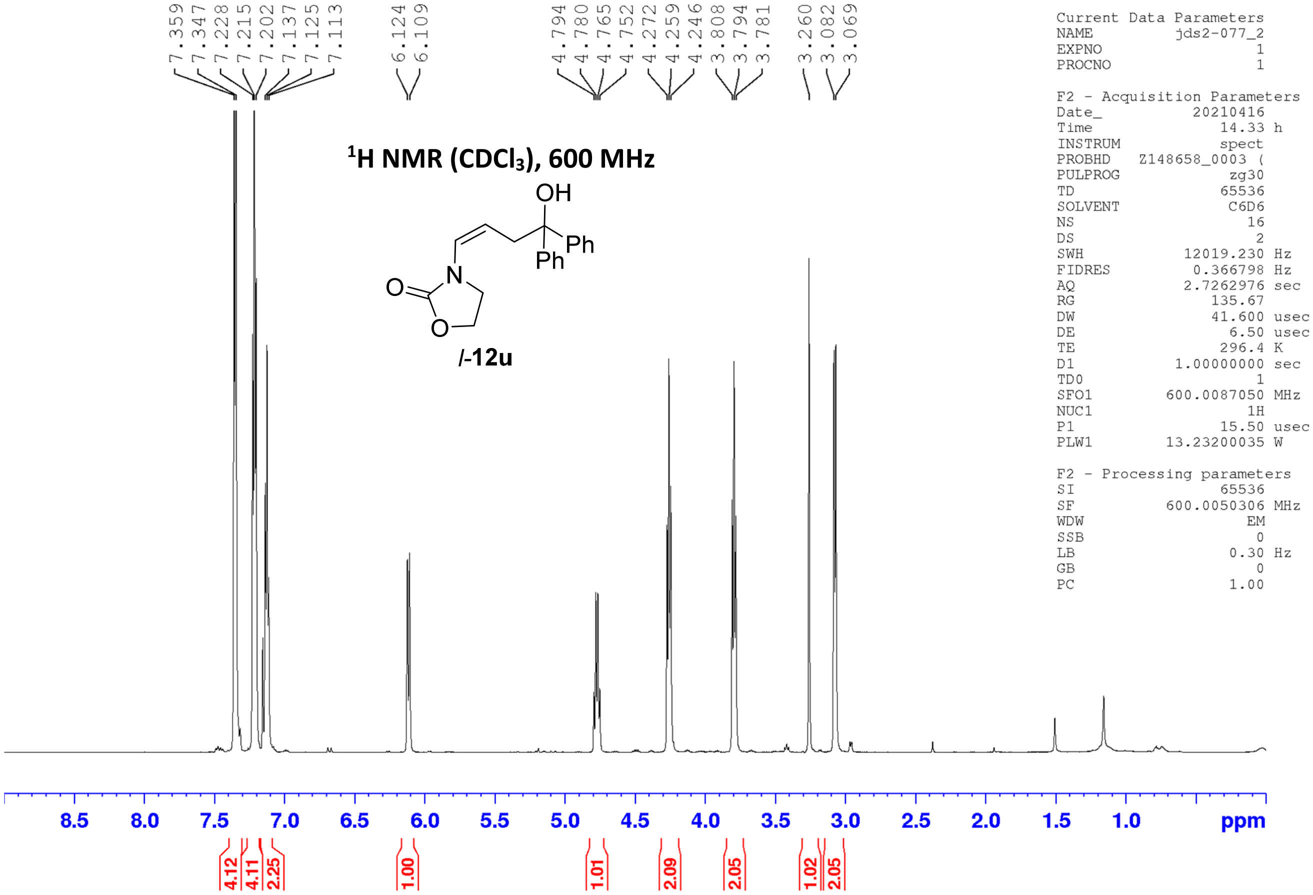


```
Current Data Parameters
NAME            jds2-077_2
EXPNO                    1
PROCNO                   1

F2 - Acquisition Parameters
Date_             20210416
Time                 14.33 h
INSTRUM              spect
PROBHD     Z148658_0003 (
PULPROG               zg30
TD                   65536
SOLVENT               C6D6
NS                      16
DS                       2
SWH              12019.230 Hz
FIDRES            0.366798 Hz
AQ               2.7262976 sec
RG                  135.67
DW                  41.600 usec
DE                    6.50 usec
TE                   296.4 K
D1              1.00000000 sec
TD0                      1
SFO1           600.0087050 MHz
NUC1                    1H
P1                   15.50 usec
PLW1           13.23200035 W

F2 - Processing parameters
SI                   65536
SF             600.0050306 MHz
WDW                     EM
SSB                      0
LB                    0.30 Hz
GB                       0
PC                    1.00
```

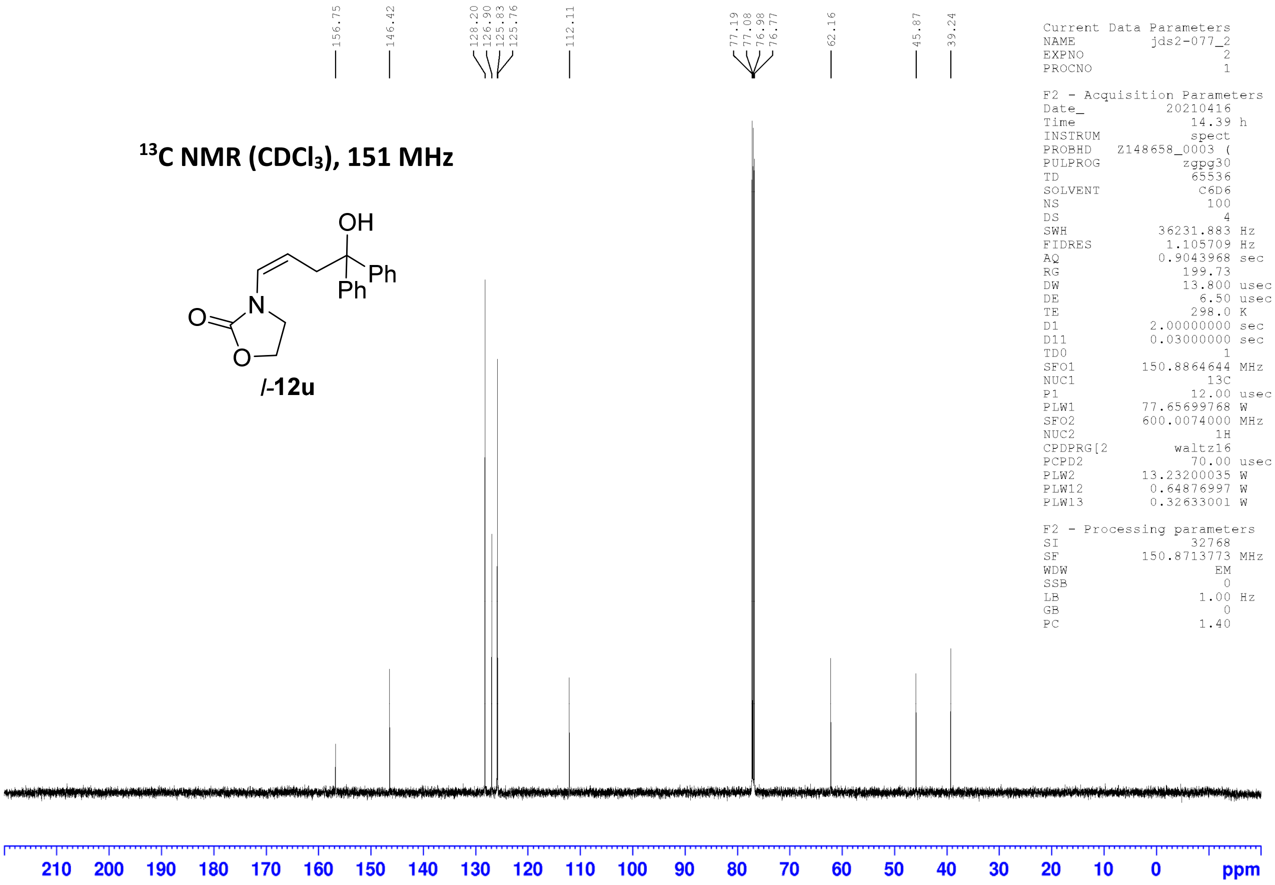


```
Current Data Parameters
NAME            jds2-077_2
EXPNO                    2
PROCNO                   1

F2 - Acquisition Parameters
Date_             20210416
Time                 14.39 h
INSTRUM              spect
PROBHD     Z148658_0003 (
PULPROG             zgpg30
TD                   65536
SOLVENT               C6D6
NS                     100
DS                       4
SWH              36231.883 Hz
FIDRES            1.105709 Hz
AQ               0.9043968 sec
RG                  199.73
DW                  13.800 usec
DE                    6.50 usec
TE                   298.0 K
D1              2.00000000 sec
D11             0.03000000 sec
TD0                      1
SFO1           150.8864644 MHz
NUC1                   13C
P1                   12.00 usec
PLW1           77.65699768 W
SFO2           600.0074000 MHz
NUC2                    1H
CPDPRG[2           waltz16
PCPD2                70.00 usec
PLW2           13.23200035 W
PLW12           0.64876997 W
PLW13           0.32633001 W

F2 - Processing parameters
SI                   32768
SF             150.8713773 MHz
WDW                     EM
SSB                      0
LB                    1.00 Hz
GB                       0
PC                    1.40
```

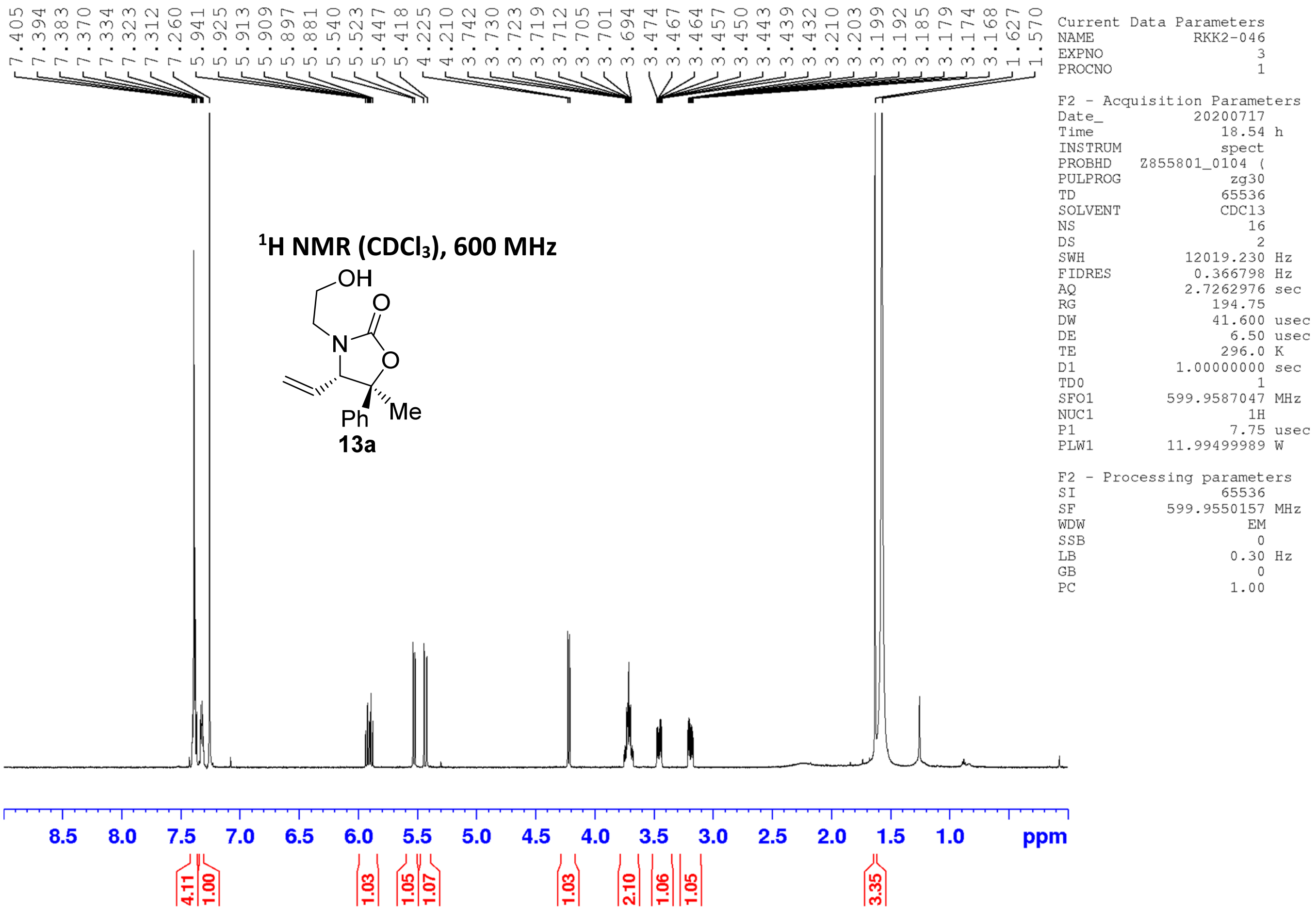

7.405 7.394 7.383 7.370 7.334 7.323 7.312 7.260 5.941 5.925 5.913 5.909 5.897 5.881 5.540 5.523 5.447 5.418 4.225 4.210 3.742 3.730 3.723 3.719 3.712 3.705 3.701 3.694 3.474 3.467 3.464 3.457 3.450 3.443 3.439 3.432 3.210 3.203 3.199 3.192 3.185 3.179 3.174 3.168 1.627 1.570
Current Data Parameters
NAME RKK2-046
EXPNO 3
PROCNO 1
F2 - Acquisition Parameters
Date_ 20200717
Time 18.54 h
INSTRUM spect
PROBHD Z855801_0104 (
PULPROG zg30
TD 65536
SOLVENT CDCl3
NS 16
DS 2
SWH 12019.230 Hz
FIDRES 0.366798 Hz
AQ 2.7262976 sec
RG 194.75
DW 41.600 usec
DE 6.50 usec
TE 296.0 K
D1 1.00000000 sec
TD0 1
SFO1 599.9587047 MHz
NUC1 1H
P1 7.75 usec
PLW1 11.99499989 W
F2 - Processing parameters
SI 65536
SF 599.9550157 MHz
WDW EM
SSB 0
LB 0.30 Hz
GB 0
PC 1.00
1H NMR (CDCl3), 600 MHz
OH
O
N
O
Ph
Me
13a
8.5 8.0 7.5 7.0 6.5 6.0 5.5 5.0 4.5 4.0 3.5 3.0 2.5 2.0 1.5 1.0 ppm
4.11 1.00 1.03 1.05 1.07 1.03 2.10 1.06 1.05 3.35


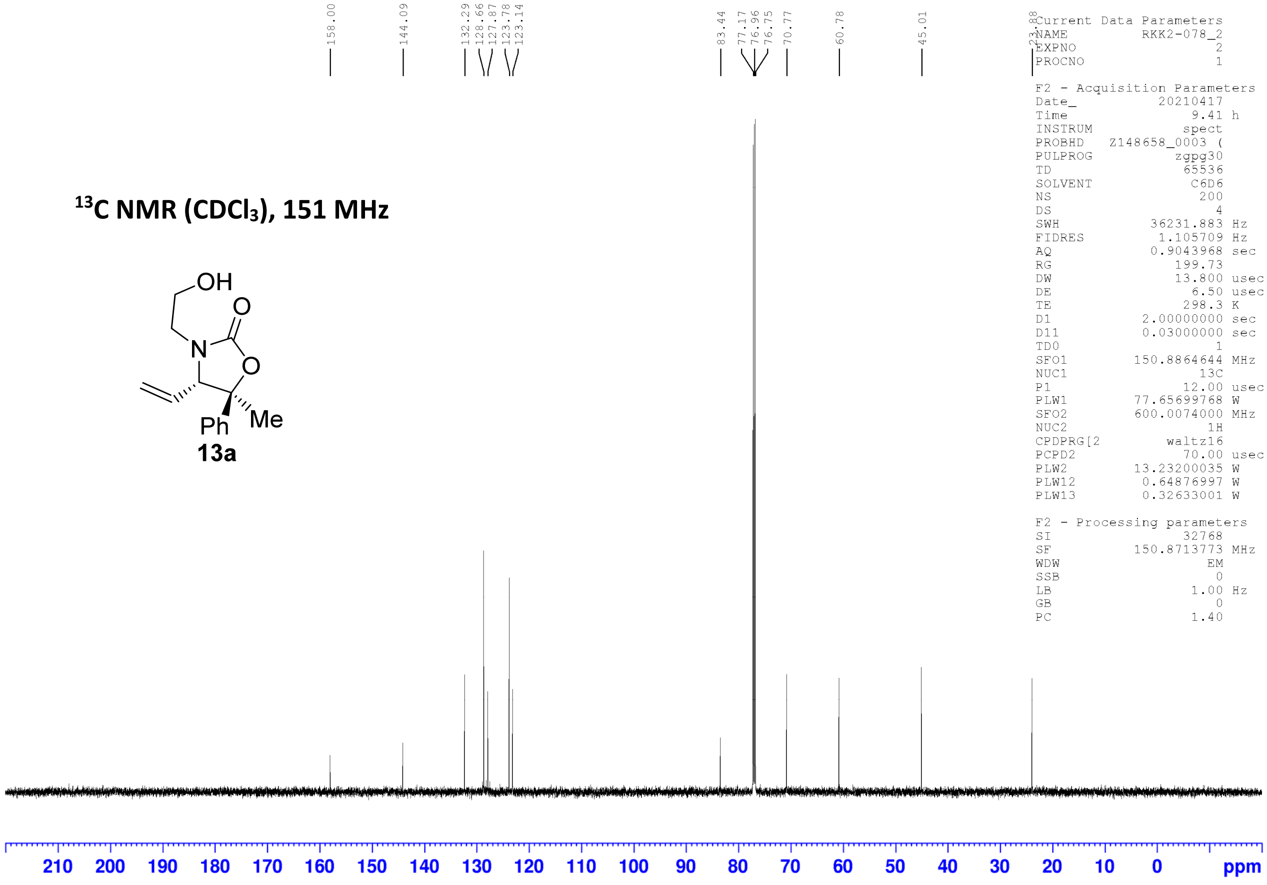

158.00 144.09 132.29 128.66 127.87 123.78 123.14 83.44 77.17 76.96 76.75 70.77 60.78 45.01 23.88
Current Data Parameters
NAME RKK2-078_2
EXPNO 2
PROCNO 1
F2 - Acquisition Parameters
Date_ 20210417
Time 9.41 h
INSTRUM spect
PROBHD Z148658_0003 (
PULPROG zgpg30
TD 65536
SOLVENT C6D6
NS 200
DS 4
SWH 36231.883 Hz
FIDRES 1.105709 Hz
AQ 0.9043968 sec
RG 199.73
DW 13.800 usec
DE 6.50 usec
TE 298.3 K
D1 2.00000000 sec
D11 0.03000000 sec
TD0 1
SFO1 150.8864644 MHz
NUC1 13C
P1 12.00 usec
PLW1 77.65699768 W
SFO2 600.0074000 MHz
NUC2 1H
CPDPRG[2 waltz16
PCPD2 70.00 usec
PLW2 13.23200035 W
PLW12 0.64876997 W
PLW13 0.32633001 W
F2 - Processing parameters
SI 32768
SF 150.8713773 MHz
WDW EM
SSB 0
LB 1.00 Hz
GB 0
PC 1.40
13C NMR (CDCl3), 151 MHz
OH
O
N
O
Ph
Me
13a
210 200 190 180 170 160 150 140 130 120 110 100 90 80 70 60 50 40 30 20 10 0 ppm

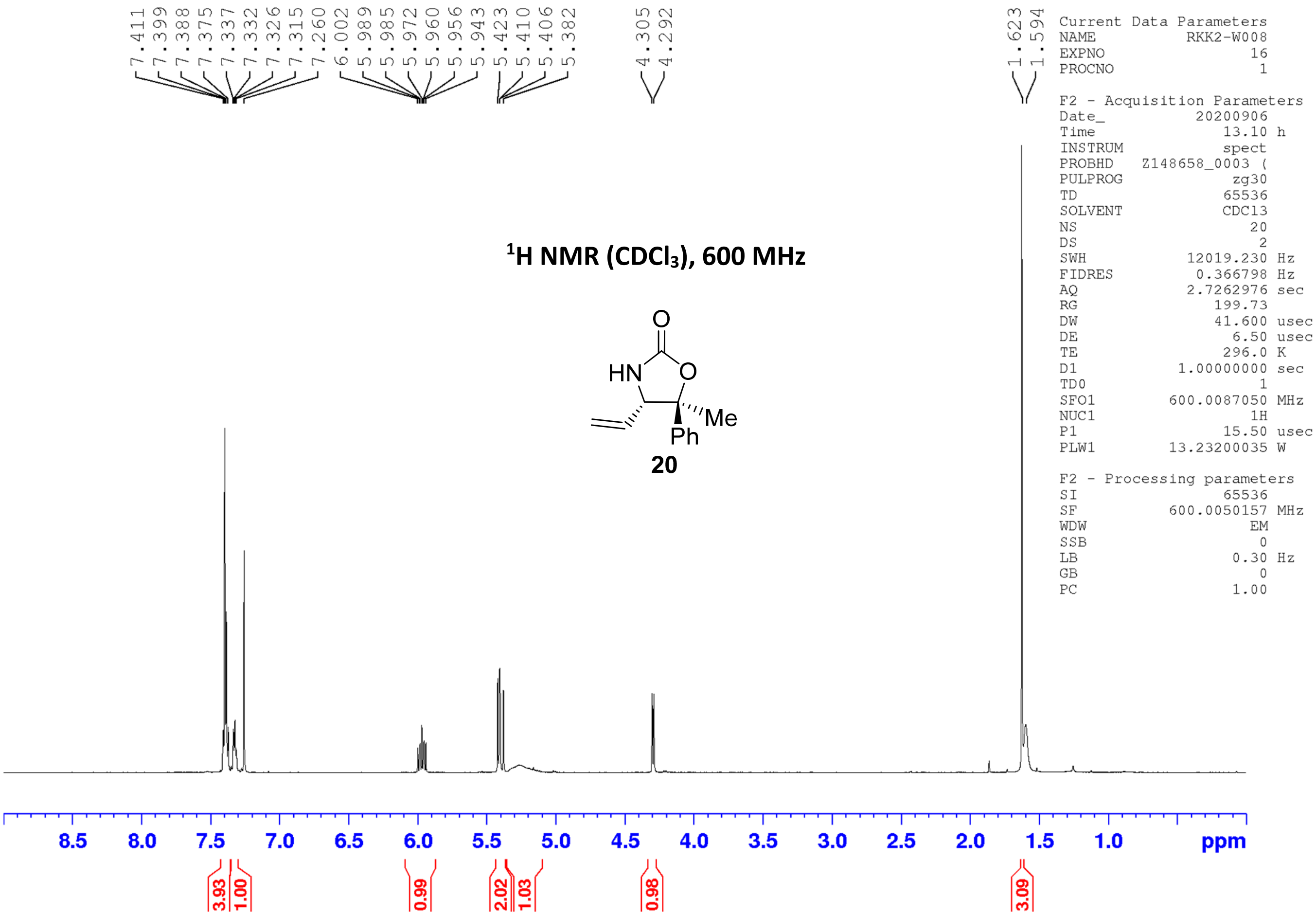
7.411 7.399 7.388 7.375 7.337 7.332 7.326 7.315 7.260 6.002 5.989 5.985 5.972 5.960 5.956 5.943 5.423 5.410 5.406 5.382
4.305 4.292
1.623 1.594
1H NMR (CDCl3), 600 MHz
HN
O
O
Me
Ph
20
Current Data Parameters
NAME RKK2-W008
EXPNO 16
PROCNO 1
F2 - Acquisition Parameters
Date_ 20200906
Time 13.10 h
INSTRUM spect
PROBHD Z148658_0003 (
PULPROG zg30
TD 65536
SOLVENT CDCl3
NS 20
DS 2
SWH 12019.230 Hz
FIDRES 0.366798 Hz
AQ 2.7262976 sec
RG 199.73
DW 41.600 usec
DE 6.50 usec
TE 296.0 K
D1 1.00000000 sec
TD0 1
SFO1 600.0087050 MHz
NUC1 1H
P1 15.50 usec
PLW1 13.23200035 W
F2 - Processing parameters
SI 65536
SF 600.0050157 MHz
WDW EM
SSB 0
LB 0.30 Hz
GB 0
PC 1.00
8.5 8.0 7.5 7.0 6.5 6.0 5.5 5.0 4.5 4.0 3.5 3.0 2.5 2.0 1.5 1.0 ppm
3.93 1.00 0.99 2.02 1.03 0.98 3.09

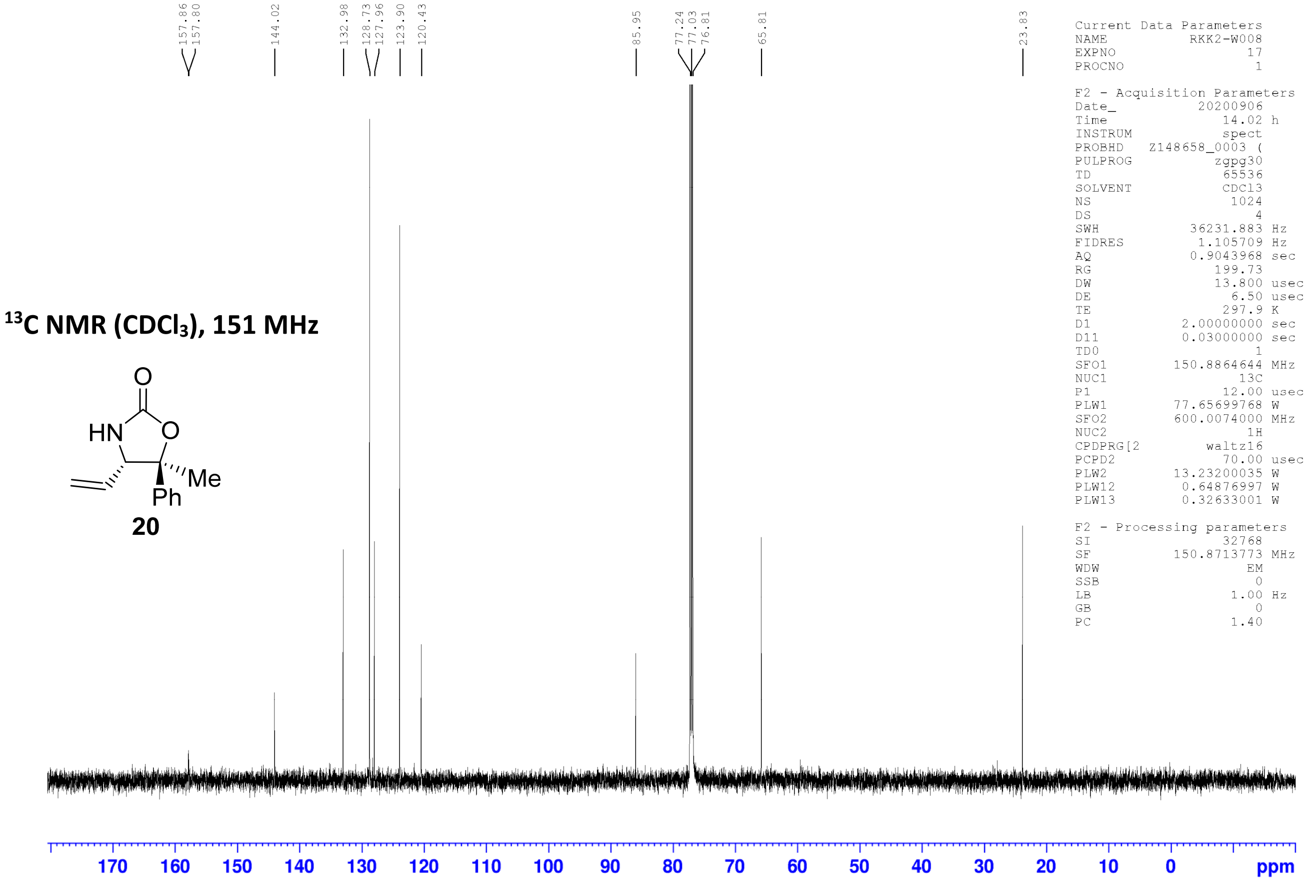
157.86 157.80 144.02 132.98 128.73 127.96 123.90 120.43 85.95 77.24 77.03 76.81 65.81 23.83
13C NMR (CDCl3), 151 MHz
HN
O
O
Me
Ph
20
Current Data Parameters
NAME RKK2-W008
EXPNO 17
PROCNO 1
F2 - Acquisition Parameters
Date_ 20200906
Time 14.02 h
INSTRUM spect
PROBHD Z148658_0003 (
PULPROG zgpg30
TD 65536
SOLVENT CDCl3
NS 1024
DS 4
SWH 36231.883 Hz
FIDRES 1.105709 Hz
AQ 0.9043968 sec
RG 199.73
DW 13.800 usec
DE 6.50 usec
TE 297.9 K
D1 2.00000000 sec
D11 0.03000000 sec
TD0 1
SFO1 150.8864644 MHz
NUC1 13C
P1 12.00 usec
PLW1 77.65699768 W
SFO2 600.0074000 MHz
NUC2 1H
CPDPRG[2 waltz16
PCPD2 70.00 usec
PLW2 13.23200035 W
PLW12 0.64876997 W
PLW13 0.32633001 W
F2 - Processing parameters
SI 32768
SF 150.8713773 MHz
WDW EM
SSB 0
LB 1.00 Hz
GB 0
PC 1.40
170 160 150 140 130 120 110 100 90 80 70 60 50 40 30 20 10 0 ppm

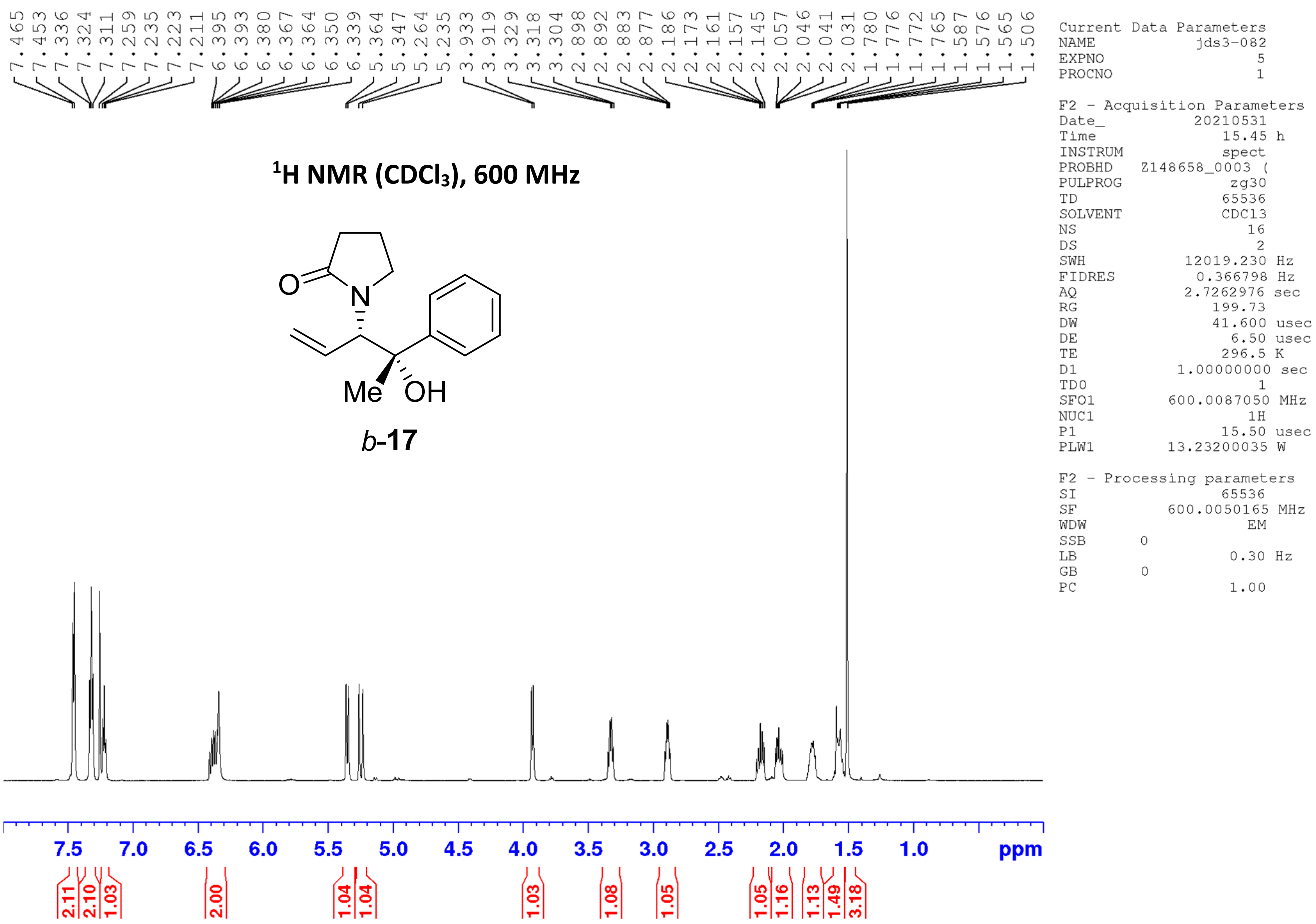
7.465 7.453 7.336 7.324 7.311 7.259 7.235 7.223 7.211 6.395 6.393 6.380 6.367 6.364 6.350 6.339 5.364 5.347 5.264 5.235 3.933 3.919 3.329 3.318 3.304 2.898 2.892 2.883 2.877 2.186 2.173 2.161 2.157 2.145 2.057 2.046 2.041 2.031 1.780 1.776 1.772 1.765 1.587 1.576 1.565 1.506
¹H NMR (CDCl₃), 600 MHz
O
N
Me
OH
b-17
Current Data Parameters
NAME jds3-082
EXPNO 5
PROCNO 1
F2 - Acquisition Parameters
Date_ 20210531
Time 15.45 h
INSTRUM spect
PROBHD Z148658_0003 (
PULPROG zg30
TD 65536
SOLVENT CDCl3
NS 16
DS 2
SWH 12019.230 Hz
FIDRES 0.366798 Hz
AQ 2.7262976 sec
RG 199.73
DW 41.600 usec
DE 6.50 usec
TE 296.5 K
D1 1.00000000 sec
TD0 1
SFO1 600.0087050 MHz
NUC1 1H
P1 15.50 usec
PLW1 13.23200035 W
F2 - Processing parameters
SI 65536
SF 600.0050165 MHz
WDW EM
SSB 0
LB 0.30 Hz
GB 0
PC 1.00
7.5 7.0 6.5 6.0 5.5 5.0 4.5 4.0 3.5 3.0 2.5 2.0 1.5 1.0 ppm
2.11 2.10 1.03 2.00 1.04 1.04 1.03 1.08 1.05 1.05 1.16 1.13 1.49 3.18

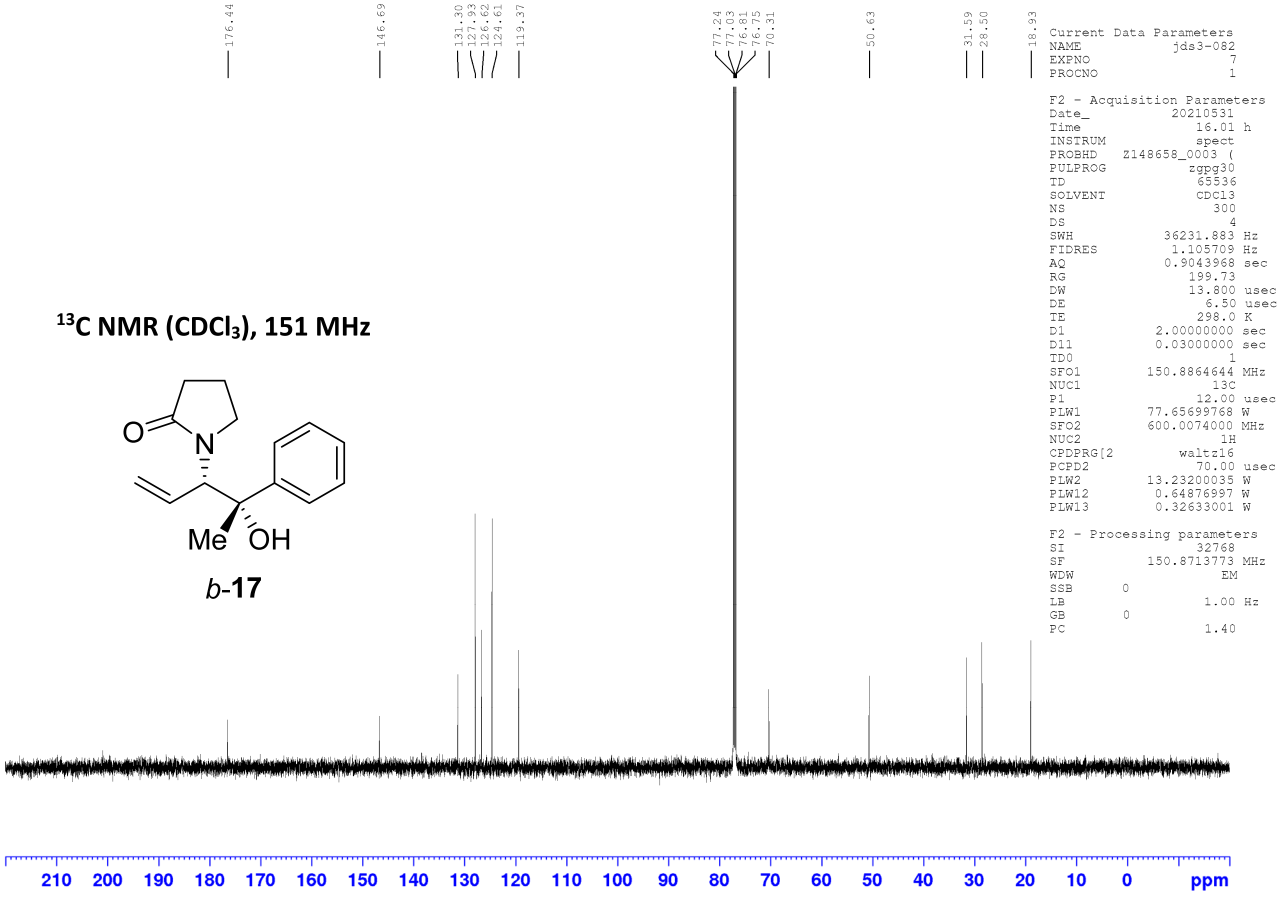
176.44 146.69 131.30 127.93 126.62 124.61 119.37 77.24 77.03 76.81 76.75 70.31 50.63 31.59 28.50 18.93
¹³C NMR (CDCl₃), 151 MHz
O
N
Me
OH
b-17
Current Data Parameters
NAME jds3-082
EXPNO 7
PROCNO 1
F2 - Acquisition Parameters
Date_ 20210531
Time 16.01 h
INSTRUM spect
PROBHD Z148658_0003 (
PULPROG zgpg30
TD 65536
SOLVENT CDCl3
NS 300
DS 4
SWH 36231.883 Hz
FIDRES 1.105709 Hz
AQ 0.9043968 sec
RG 199.73
DW 13.800 usec
DE 6.50 usec
TE 298.0 K
D1 2.00000000 sec
D11 0.03000000 sec
TD0 1
SFO1 150.8864644 MHz
NUC1 13C
P1 12.00 usec
PLW1 77.65699768 W
SFO2 600.0074000 MHz
NUC2 1H
CPDPRG[2 waltz16
PCPD2 70.00 usec
PLW2 13.23200035 W
PLW12 0.64876997 W
PLW13 0.32633001 W
F2 - Processing parameters
SI 32768
SF 150.8713773 MHz
WDW EM
SSB 0
LB 1.00 Hz
GB 0
PC 1.40
210 200 190 180 170 160 150 140 130 120 110 100 90 80 70 60 50 40 30 20 10 0 ppm

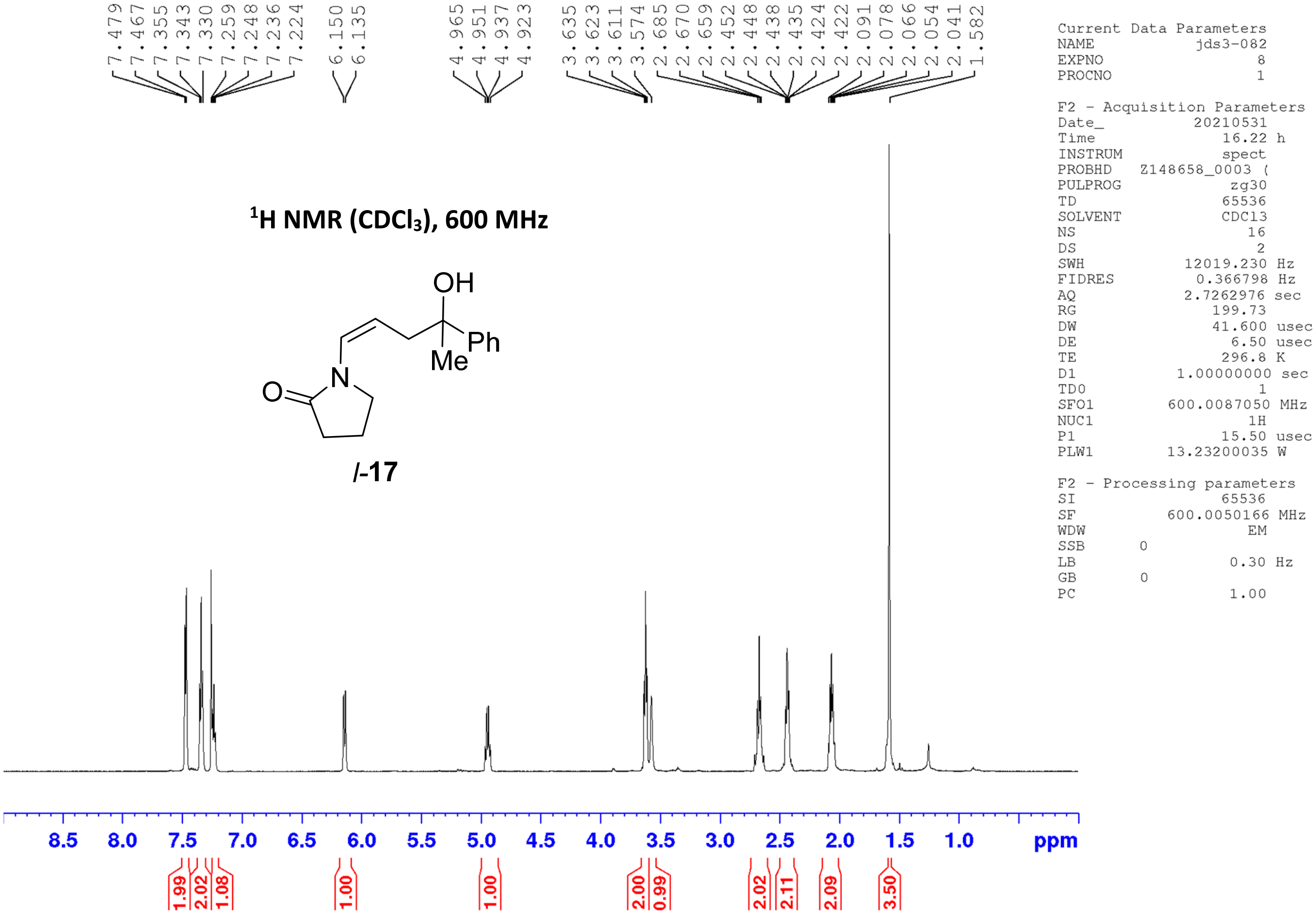
7.479 7.467 7.355 7.343 7.330 7.259 7.248 7.236 7.224 6.150 6.135 4.965 4.951 4.937 4.923 3.635 3.623 3.611 3.574 2.685 2.670 2.659 2.452 2.448 2.438 2.435 2.424 2.422 2.091 2.078 2.066 2.054 2.041 1.582
$^1$H NMR ($CDCl_3$), 600 MHz
OH
Ph
Me
N
O
l-17
Current Data Parameters
NAME jds3-082
EXPNO 8
PROCNO 1
F2 - Acquisition Parameters
Date_ 20210531
Time 16.22 h
INSTRUM spect
PROBHD Z148658_0003 (
PULPROG zg30
TD 65536
SOLVENT CDCl3
NS 16
DS 2
SWH 12019.230 Hz
FIDRES 0.366798 Hz
AQ 2.7262976 sec
RG 199.73
DW 41.600 usec
DE 6.50 usec
TE 296.8 K
D1 1.00000000 sec
TD0 1
SFO1 600.0087050 MHz
NUC1 1H
P1 15.50 usec
PLW1 13.23200035 W
F2 - Processing parameters
SI 65536
SF 600.0050166 MHz
WDW EM
SSB 0
LB 0.30 Hz
GB 0
PC 1.00
8.5 8.0 7.5 7.0 6.5 6.0 5.5 5.0 4.5 4.0 3.5 3.0 2.5 2.0 1.5 1.0 ppm
1.99 2.02 1.08 1.00 1.00 2.00 0.99 2.02 2.11 2.09 3.50

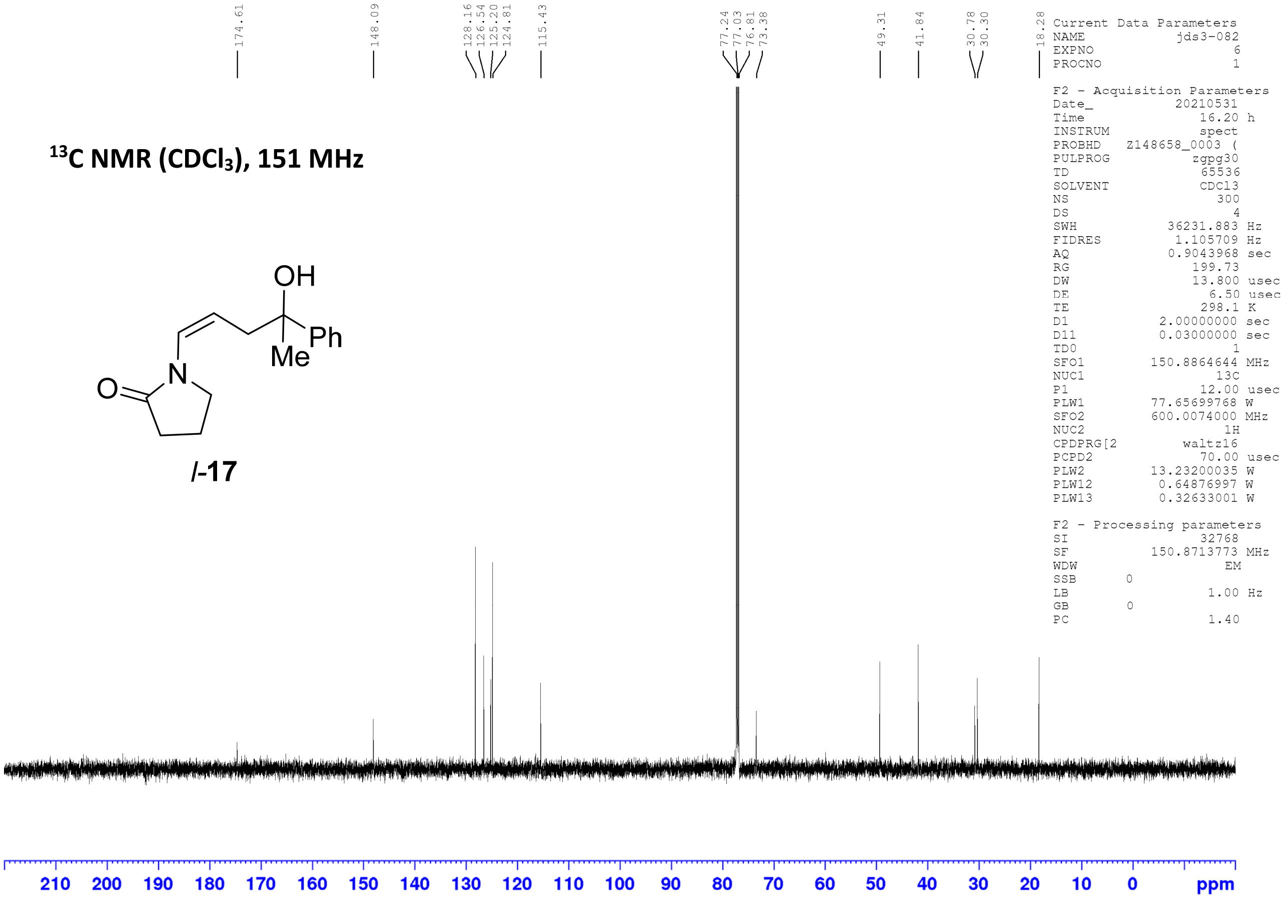
174.61 148.09 128.16 126.54 125.20 124.81 115.43 77.24 77.03 76.81 73.38 49.31 41.84 30.78 30.30 18.28
$^{13}$C NMR ($CDCl_3$), 151 MHz
OH
Ph
Me
N
O
l-17
Current Data Parameters
NAME jds3-082
EXPNO 6
PROCNO 1
F2 - Acquisition Parameters
Date_ 20210531
Time 16.20 h
INSTRUM spect
PROBHD Z148658_0003 (
PULPROG zgpg30
TD 65536
SOLVENT CDCl3
NS 300
DS 4
SWH 36231.883 Hz
FIDRES 1.105709 Hz
AQ 0.9043968 sec
RG 199.73
DW 13.800 usec
DE 6.50 usec
TE 298.1 K
D1 2.00000000 sec
D11 0.03000000 sec
TD0 1
SFO1 150.8864644 MHz
NUC1 13C
P1 12.00 usec
PLW1 77.65699768 W
SFO2 600.0074000 MHz
NUC2 1H
CPDPRG[2 waltz16
PCPD2 70.00 usec
PLW2 13.23200035 W
PLW12 0.64876997 W
PLW13 0.32633001 W
F2 - Processing parameters
SI 32768
SF 150.8713773 MHz
WDW EM
SSB 0
LB 1.00 Hz
GB 0
PC 1.40
210 200 190 180 170 160 150 140 130 120 110 100 90 80 70 60 50 40 30 20 10 0 ppm
